# Arylboration of Enecarbamates for the Synthesis of Borylated Saturated N‐Heterocycles

**DOI:** 10.1002/anie.202212117

**Published:** 2022-10-17

**Authors:** Grace L. Trammel, Prashansa B. Kannangara, Dmytro Vasko, Oleksandr Datsenko, Pavel Mykhailiuk, M. Kevin Brown

**Affiliations:** ^1^ Department of Chemistry Indiana University 800 E. Kirkwood Ave. Bloomington IN, 47401 USA; ^2^ Enamine Ltd. Chervonotkatska 60 02094 Kyiv Ukraine; ^3^ Taras Shevchenko National University of Kyiv Chemistry Department Volodymyrska 64 01601 Kyiv Ukraine

**Keywords:** Alkene, Arylation, Boron, Cross Coupling, Heterocycles

## Abstract

Two catalytic systems have been developed for the arylboration of endocyclic enecarbamates to deliver synthetically versatile borylated saturated N‐heterocycles in good regio‐ and diastereoselectivities. A Cu/Pd dual catalytic reaction enables the synthesis of borylated, α‐arylated azetidines, while a Ni‐catalysed arylboration reaction efficiently functionalizes 5‐, 6‐, and 7‐membered enecarbamates. In the case of the Cu/Pd‐system, a remarkable additive effect was identified that allowed for broader scope. The products are synthetically useful, as demonstrated by manipulations of the boronic ester to access biologically active compounds.

## Graphical abstract

Saturated N‐heterocycles are important motifs found in many biologically active molecules. In this work, difunctionalization of 4–7‐membered enecarbamates is achieved using both Cu/Pd and Ni‐catalysed arylboration to deliver synthetically versatile borylated saturated N‐heterocycles in good selectivity. The products are synthetically useful, as demonstrated by manipulations of the boronic ester to access biologically active compounds.

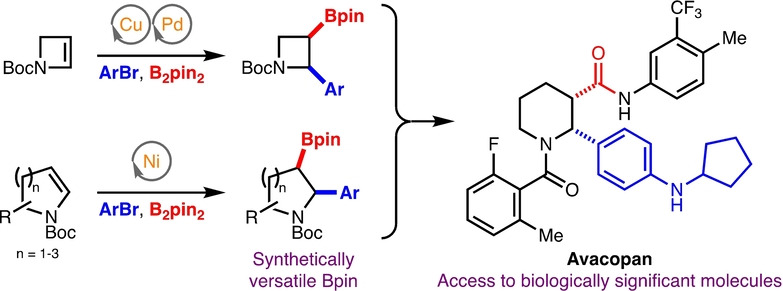

Saturated N‐heterocycles are among the most prominent motifs in biologically active molecules, appearing in pharmaceuticals, agrochemicals and natural products.[^1^, ^2^, ^3^] 59 % of FDA approved pharmaceuticals contain an aza‐heterocycle, with 4 out of the top 5 most prevalent heterocycles being saturated rings (Scheme 1a).^[1]^ Specifically, piperidine is the number one most prominent heterocycle and pyrrolidine the fifth most prevalent N‐heterocycle in FDA‐approved drugs.^[1]^ Azetidines are less prominent than their larger ring counterparts, though interest in incorporating azetidines into pharmaceuticals is rising due to a variety of desirable attributes including polarity, rigidity and basicity that can lead to improved pharmacokinetic properties, such as solubility, lipophilicity, and metabolic stability.^[4]^ Azetidines have also served as replacements for larger rings such as piperidine,^[5]^ pyrrolidine,^[6]^ and others,^[4]^ resulting in favourable improvements.

**Scheme 1:**
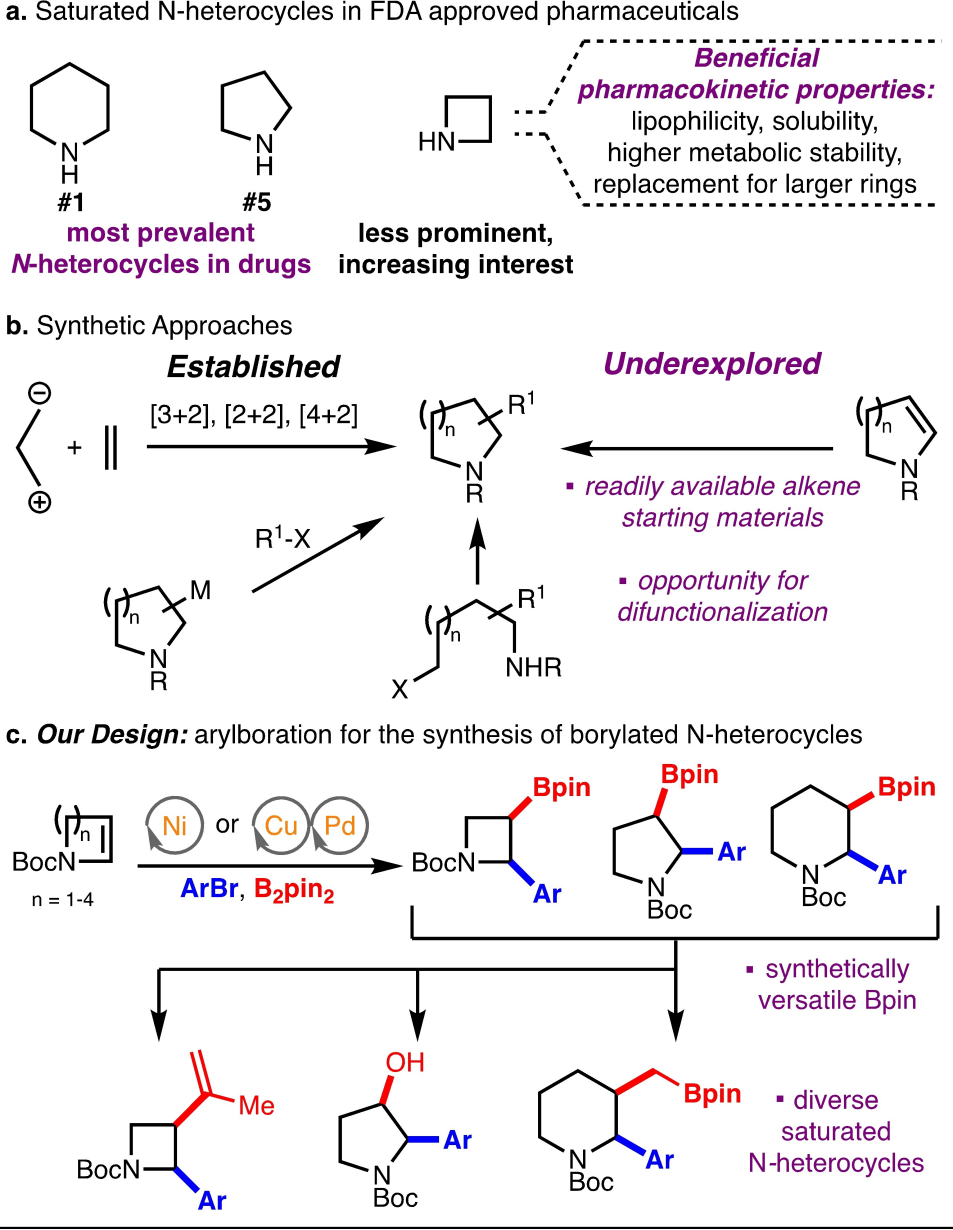
Occurrence and synthesis of saturated N‐heterocycles.

Functionalized saturated N‐heterocycles, including azetidines,^[7]^ pyrrolidines,^[8]^ and piperidines^[9]^ are commonly accessed through cycloadditions,^[10]^ cyclization from linear precursors, and C−H functionalization (Scheme 1b).[^11^, ^12^, ^13^, ^14^] While these approaches are useful for accessing complex scaffolds, an alternative, though underexplored, approach to saturated N‐heterocycles is functionalization of endocyclic enecarbamates. Enecarbamate difunctionalization is attractive, as simple and readily available alkene precursors can allow for rapid build‐up of molecular complexity.^[15]^

Previously reported monofunctionalizations of enecarbamates include Heck reactions followed by hydrogenation to access α‐arylated saturated N‐heterocycles.^[16]^ Additionally, hydrofunctionalizations^[17]^ of enecarbamates including hydroarylation,^[18]^ hydrosilylation,^[19]^ hydroformylation,^[20]^ and hydroboration^[21]^ have also been reported. Difunctionalizations of enecarbamates, which access greater molecular complexity in a single synthetic operation, have been explored and include: Pd‐catalysed alkyl‐alkoxylation,^[22]^ diheterofunctionalization,^[23]^ Cu‐catalysed trifluoromethylalkoxylation,^[24]^ Ni‐catalysed aryldifluoroalkylation^[25]^ and alkylboration,^[26]^ and radical‐mediated functionalizations.^[27]^

We envisioned that arylboration of endocyclic enecarbamates could allow for the synthesis of α‐arylated saturated N‐heterocycles, which are a privileged motif in pharmaceuticals,[^12a^, ^12b^, ^12c^] with simultaneous installation of a synthetically useful C−B bond, which could be transformed to various functional groups to access wide chemical space (Scheme 1c).[^28^, ^29^] In this work, we have achieved the arylboration of endocyclic enecarbamates for the synthesis of borylated saturated N‐heterocycles of various ring sizes (4–7) and substitutions using two catalytic systems: a) a Cu/Pd dual catalytic reaction^[30]^ for arylboration of N‐Boc‐2‐azetine to access azetidines, and b) a Ni‐catalysed system^[31]^ for arylboration of 5–7‐membered endocyclic enecarbamates to access pyrrolidines, piperidines, and azepanes (Scheme 1c). The reaction generates useful building blocks to access broad chemical space, as demonstrated through functionalization of the Bpin handle for the synthesis of a variety of saturated N‐heterocyclic derivatives as well as biologically active molecules Avacopan^[2]^ and an hNK1 antagonist.^[32]^

Our group has developed a Cu/Pd dual catalytic system for the arylboration of activated alkenes, including strained substrates such as spirocyclic cyclobutenes.[^30^, ^33^] Additionally, we and others, have previously reported Cu‐catalysed borylation of cyclobutenes.^[34]^ Based on this precedence, we hypothesized that borylcupration of N*‐*Boc‐2‐azetine (**1**) might proceed selectively to deliver borylated azetidine product (**2**). Subjecting N*‐*Boc‐2‐azetine (**1**) to a variety of Cu/Pd catalysed conditions, it was eventually found that RuPhos‐Pd G3 precatalyst^[35]^ and a pyridylidene‐CuCl catalyst previously developed by our lab^[36]^ were superior to other catalysts evaluated, such as the more common SIMes‐CuCl or IPr‐CuCl (Scheme 2a, See Supporting Information for more details). However, while we did note that the Pyridylidene‐CuCl catalyst was superior, the yield was variable for unknown reasons. Mechanistically, we propose that the reaction proceeds through boryl‐cupration of N‐Boc‐2‐azetine to form alkyl‐Cu intermediate **III**, which undergoes stereoretentive transmetallation with Ar‐Pd‐X **V** to generate alkyl‐Pd intermediate **VI** (Scheme 2b).^[30]^ Finally, reductive elimination delivers the arylboration product and regenerates the Pd^0^ catalyst **IV**.

**Scheme 2:**
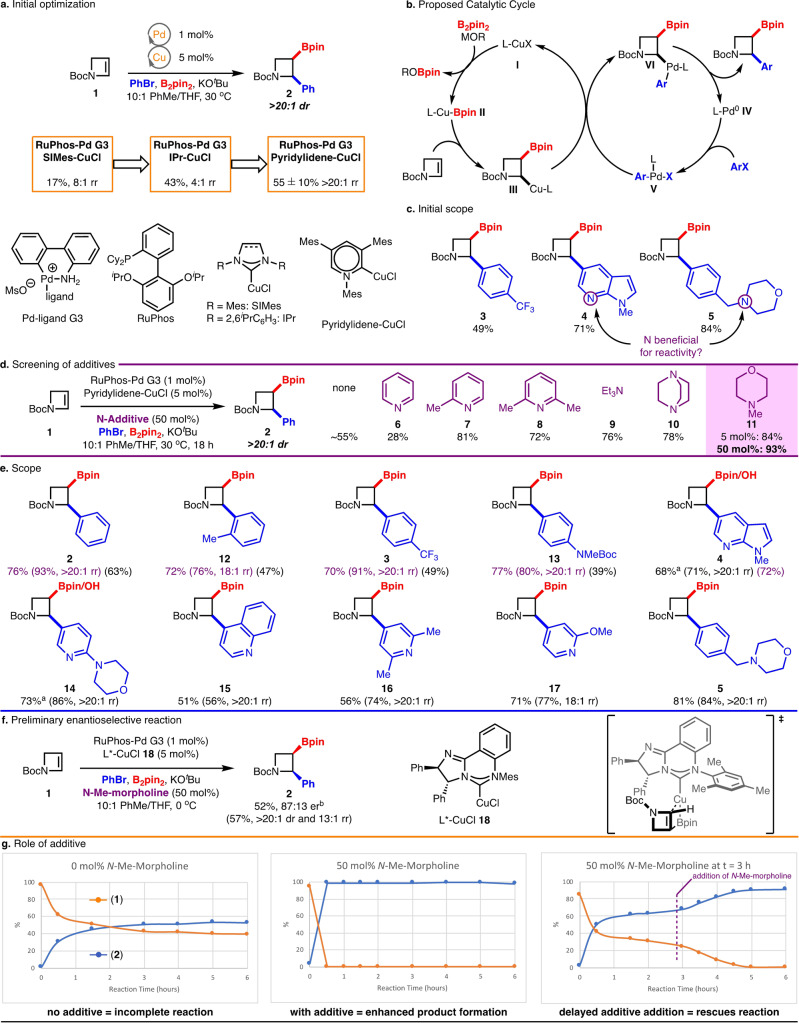
Cu/Pd‐catalysed synthesis of azetidines. a,c,e) Crude yields & dr's determined by ^1^H NMR relative to mesitylene. e.) Yields are reported as the average of two isolated experiments and products were isolated in >20 : 1 dr & rr. Crude yields, dr's, and rr's are reported as the average of two experiments in parentheses and were determined through ^1^H NMR analysis of the crude reaction mixture relative to an internal standard. Yields for reactions with N−Me‐morpholine are in purple. ^a^ Isolated as the alcohol after Bpin oxidation. ^b^ Absolute configuration has not been determined, but is predicted based on the model provided in f.

With these conditions in hand, scope exploration commenced. As a variety of arylbromide partners were subjected to the reaction conditions, it became apparent that substrates containing basic nitrogen functionality (e.g. tertiary amine, pyridine, **4**, **5**) performed better than arylbromides lacking a basic nitrogen (**3**) (Scheme 2c). We hypothesized that a nitrogen additive could be employed to enhance the yield of the reactions of arylbromides without a basic nitrogen.

Addition of 50 mol% (chosen for ease and accuracy of reaction setup) of a variety of pyridine derivatives (**6**–**8**) revealed that the yield could be improved, and that the reaction is sensitive to the steric environment around the nitrogen (Scheme 2d). 2‐picoline (**7**) was superior to strongly binding pyridine (**6**), and also performed better than 2,6‐lutidine (**8**), presumably because the lutidine nitrogen is sterically shielded by two methyl groups. Tertiary alkyl amines were also screened, and it was found that triethylamine (**9**) and DABCO (**10**) could also enhance reactivity. N‐methyl‐morpholine (**11**) was found to be the optimal nitrogen‐based additive and could produce similar yield enhancement at loadings as low as 5 mol%. It is also important to note that upon inclusion of N‐methyl‐morpholine (**11**) the reactions were now reproducible.

Gratifyingly, it was found that addition of 50 mol% N‐methyl‐morpholine could enhance the yields of arylbromide partners lacking basic nitrogen functionality besides bromobenzene, including sterically hindered (product **12**), electron‐deficient (product **3**), and electron‐rich (product **13**) substrates (Scheme 2e). However, the yield was not significantly improved with the addition of N‐methyl‐morpholine to the reaction with a heterocyclic arylbromide (product **4**). Additional heterocyclic arylbromides functioned in the reaction, including quinoline and various pyridine derivatives (products **14**–**17**). Preliminary investigation into the enantioselective version of the reaction was explored with a chiral NHC‐CuCl complex (**18**) (Scheme 2f).[^37^, ^38^] It was found that under the standard reaction conditions at 0 °C, azetidine product **2** could be formed in 87 : 13 er and moderate yield. A model for enantioselective migratory insertion is provided based on prior work with complex **18**, in which the smaller side of the azetine (H versus N‐Boc) approaches towards the mesitylene ring.^[39]^ Overall, the reaction provides access to diverse α‐arylated azetidines in synthetically useful yields, which are otherwise difficult to prepare.

In order to probe the role of N*‐*methyl‐morpholine, the reaction was monitored over time in both the presence and absence of 50 mol% N*‐*methyl‐morpholine (**11**) (Scheme 2g). An appreciable enhancement in product formation was observed for the reaction with 50 mol% N*‐*methyl‐morpholine (**11**), compared to the reaction with no additive, resulting in full conversion of N‐Boc‐2‐azetine (**1**) to desired product **2** in 30 minutes. In the absence of additive, the reaction stalled at ≈60 % conversion around 3 hours. However, when 50 mol% N*‐*methyl‐morpholine (**11**) was added to the reaction at 3 hours, the reactivity was restored, and the reaction reached >98 % conversion and 90 % yield within an additional 3 hours after addition of N−Me‐morpholine (**11**). The ability of the N‐additive to rescue the reaction could indicate that one or both catalysts are becoming inactive by forming off‐cycle species that can be re‐converted to the active catalysts by coordination to an amine ligand.[^40^, ^41^] It is known that CuBpin complexes can aggregate, thus a potential role for N−Me‐morpholine is to disrupt the formation of these species.^[42]^ Under the optimal reaction conditions (with 50 mol% N−Me‐morpholine (**11**) or amine containing substrates), the enhanced reaction conversion may be attributed to mitigation of catalyst inactivation resulting in higher concentration of active catalyst, leading to improved reaction outcomes.

Due to the prevalence of 5‐ and 6‐membered saturated N*‐*heterocycles in pharmaceuticals,^[1]^ we sought to achieve the arylboration of enecarbamates of larger ring sizes to access these borylated heterocycles. Unfortunately, under Cu/Pd‐catalysed arylboration conditions, N*‐*Boc dihydropyrrole (**19**) afforded arylboration product in low yield (<30 %). However, when (**19**) was subjected to Ni‐catalysed arylboration conditions,^31b^ the arylboration product was formed in 88 % crude yield, >10 : 1 rr and >20 : 1 dr (Scheme 3a). The major regioisomer was confirmed to be the C2 arylated product (**20**) by X‐ray crystallography after Bpin oxidation.^[43]^ Mechanistically, the process likely proceeds through a Ni(I/III) cycle in which initial formation of [Ni]^I^‐Bpin complex **VIII** is followed by regioselective addition across the alkene, perhaps due to electronic factors as well as stabilization of the resulting alkyl‐[Ni] complex **IX** by the adjacent carbamate (Scheme 3a).[^19^, ^31b^] Capture of **IX** by the arylbromide electrophile through an oxidative addition/reductive elimination sequence delivers the arylboration product and regenerates the Ni^I^ catalyst **VII**.

**Scheme 3:**
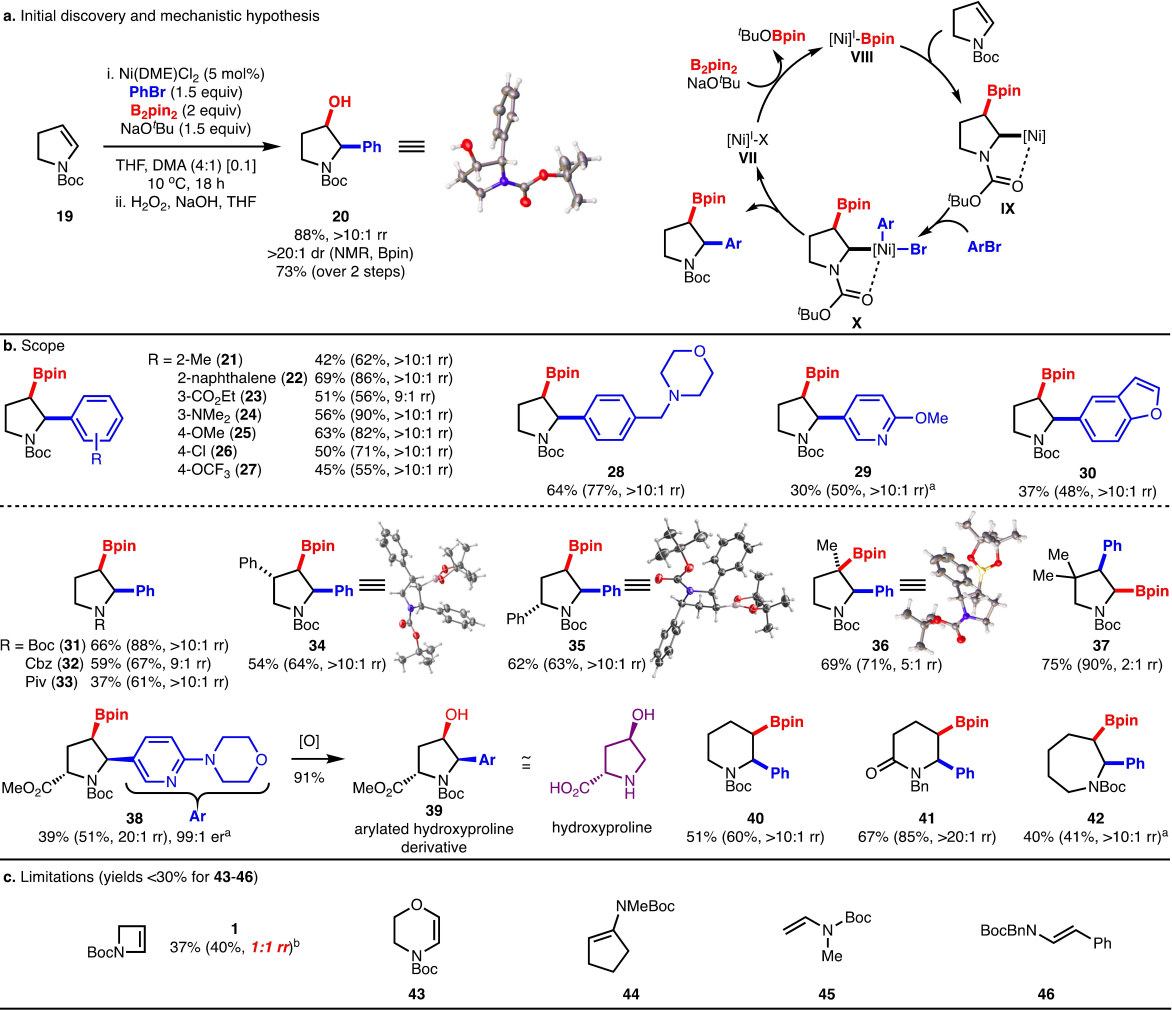
Ni‐catalysed arylboration of enecarbamates. a,b) Yields are reported as the average of two isolated experiments, and products were isolated as single diastereomers. Crude yields, dr's, and rr's are reported as the average of two experiments in parentheses and were determined through ^1^H NMR analysis of the crude reaction mixture relative to an internal standard. All products formed in >20 : 1 crude dr. Note: where rr could not be accurately determined due to rotamers, but a major regioisomer was clearly formed, the rr was assigned as >10 : 1 rr. ^a^ Run at 30 °C. ^b^ Run under standard Ni‐catalysed arylboration conditions, but with 1 : 1 THF/DMA, 3 equiv PhBr, and at 30 °C.

Scope exploration of this process was next undertaken (Scheme 3b). The arylbromide scope proved to be broad, with electron‐deficient (products **23**, **26**,**27**), electron‐rich (products **24**,**25**), sterically‐hindered (products **21**–**22**), and select heterocyclic arylbromides (products **29**,**30**) functioning in the reaction. Additionally, functionality such as tertiary amines (product **28**), esters (product **23**), and halides (product **26**) were tolerated. In addition to Boc, Cbz‐ and pivalate N‐protecting groups functioned in the reaction (products **32** and **33**). A variety of substitutions could be made on the pyrrolidine ring, with high diastereoselectivity observed with respect to existing stereocenters in all cases (products **34**, **35**, **38**). Most notably, an arylated hydroxyproline derivative **39** can be synthesized in high dr while maintaining the integrity of the existing stereocenter. Additionally, a sterically‐hindered trisubstituted alkene reacted to form the tertiary boronic ester product (**36**) demonstrating that the electronics and/or carbamate stabilization is the major driver for regioselectivity.[^31a^, ^31b^] However, an adjacent quaternary center reverses the regioselectivity to yield C2‐borylated pyrrolidine (**37**), albeit in low selectivity (2 : 1 rr). In this case, it is likely that the sterically demanding Bpin unit is positioned distal to the geminal dimethyl group to avoid steric repulsion. Gratifyingly, the Ni‐catalysed arylboration was amenable to the synthesis of piperidine (**40**), piperidone (**41**), and azepane (**42**) products. Interestingly, piperidone product (**41**) was formed in high selectivity and demonstrates that electronics alone are sufficient to control regioselectivity in the absence of a potential directing group. Notably, azetine (**1**) underwent reaction in low yield and regioselectivity under the Ni‐catalysed arylboration conditions (40 %, 1 : 1 rr, Scheme 3c) demonstrating the complementarity of Cu/Pd versus Ni‐catalysed arylboration. Additionally, it was found that cyclic substrates **43**–**44**, and acyclic substrates **45**–**46** did not undergo productive reactions under either Ni or Cu/Pd catalysis.

The synthetic utility of the arylboration reactions was demonstrated by the gram‐scale synthesis of azetidine **14** (Scheme 4a). The C−B bond of **14** underwent oxidation (product **48**), Zweifel olefination (product **50**), and Matteson homologation (product **47**) smoothly. Alcohol **48** could be transformed to fluorinated azetidine **49** in moderate yield.^[44]^ In collaboration with Enamine, the standard Ni‐catalysed arylboration of N‐Boc*‐*dihydropyrrole **19** was performed on 30 g scale using standard Schlenk techniques (Scheme 4b). After Bpin oxidation, **20** could readily be transformed to hNK1 antagonist **51**.^[32]^ Furthermore, piperidine **54** could also be synthesized in gram quantities. Elaboration through synthetic manipulation of the Bpin via homologation, oxidation and amidation allowed for preparation of a known intermediate in the synthesis FDA‐approved pharmaceutical Avacopan (**56**) (Scheme 4c).^[2]^ Our arylboration followed by Bpin functionalization strategy allows for access to diverse saturated N‐heterocyclic motifs, and could be useful in medicinal chemistry settings due to the opportunity for divergent synthesis of many products through transformations of the Bpin handle to a variety of functional groups.

**Scheme 4:**
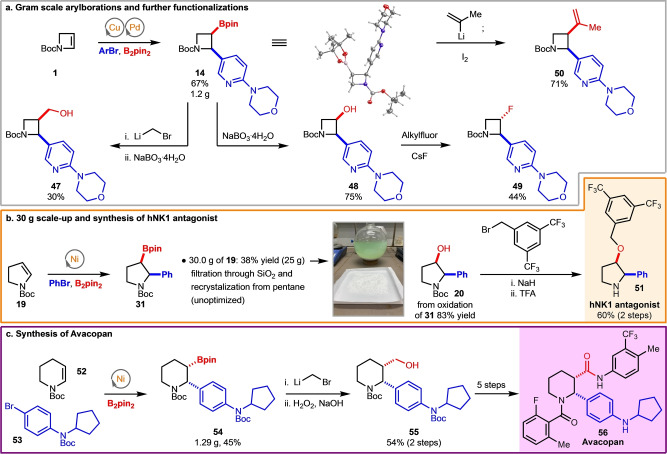
Large scale arylborations, further functionalizations and applications in synthesis.

In conclusion, we have developed a dual catalytic Cu/Pd arylboration of N*‐*Boc‐2‐azetine for the selective synthesis of borylated, α‐arylated azetidines, which are difficult to access by other methods. Key to development of this method was the identification of N−Me‐morpholine as an additive to improve the yield of the reaction. Additionally, Ni‐catalysed arylboration was employed for the functionalization of endocyclic enecarbamates of ring sizes 5–7 of various substitutions. Both reactions were amenable to scaleup, and the synthetic versatility of the arylboration products was also demonstrated.

## Conflict of interest

The authors declare no conflict of interest.

## Supporting information

As a service to our authors and readers, this journal provides supporting information supplied by the authors. Such materials are peer reviewed and may be re‐organized for online delivery, but are not copy‐edited or typeset. Technical support issues arising from supporting information (other than missing files) should be addressed to the authors.

Supporting Information

Supporting Information

Supporting Information

Supporting Information

Supporting Information

Supporting Information

## Data Availability

The data that support the findings of this study are available in the supplementary material of this article.
