# Supplementary material for: Arylboration of Enecarbamates for the Synthesis of Borylated Saturated N‐Heterocycles
Source: Angew Chem Int Ed Engl. 2022 Oct 17;61(46):e202212117. doi: 10.1002/anie.202212117 (PMC9643676; doi:10.1002/anie.202212117)
Supplement: Supplementary file 6 — Supporting Information [file ANIE-61-0-s005.pdf]

Angewandte Chemie
Eine Zeitschrift der Gesellschaft Deutscher Chemiker

Supporting Information

Arylboration of Enecarbamates for the Synthesis of Borylated Saturated N-Heterocycles

*G. L. Trammel, P. B. Kannangara, D. Vasko, O. Datsenko, P. Mykhailiuk, M. K. Brown**

Arylboration of Enecarbamates for the Synthesis of Borylated Saturated N-Heterocycles

Grace L. Trammel,[a] Prashansa B. Kannangara,[a] Dmytro Vasko,[b] Oleksandr Datsenko,[b] Pavel Mykhailiuk,[b] and M. Kevin Brown[a]*

[a]Department of Chemistry, Indiana University, 800 E. Kirkwood Ave, Bloomington, IN 47405
[b]Enamine Ltd., Chervonotkatska 60, 02094 Kyiv (Ukraine)

Table of Contents

1. General Considerations……………………………………………………………...S2

2. List of Reagents and Catalysts………………………………………………….....S2

3. Substrate Synthesis……………………………………………………………….S5

4. Cu/Pd Optimization Studies……………………………………………………..S12

5. General Procedure A: Cu/Pd-Catalyzed Arylboration of Enecarbamates………………S13

6. General Procedure B: Ni-Catalyzed Arylboration of Enecarbamates……………......S14

7. General Procedure C: Oxidation of pinacol boronic esters……………………………….S14

8. Characterization Data……………………………………………………………...S14

9. Gram-scale arylborations………………………………………………………….S39

10. Further functionalizations………………………………………………………...S42

11. Synthetic Applications……………………………………………………………S45

12. Experiments to determine role of N-methyl-morpholine………………………………..S50

13. References…………………………………………………………………………S55

14. HPLC Traces………………………………………………………………………S57

15. X-Ray Crystallography Data…………………………………………………...S65

16. NMR Spectra………………………………………………………………………S76

1. General Considerations:

Infrared (IR) spectra were recorded on a Bruker Tensor II FT- IR Spectrometer, ν_{max} in cm^{-1}. Bands are characterized as broad (br), strong (s), medium (m), and weak (w). ^1H NMR spectra were recorded at room temperature unless otherwise noted on a Varian I400 (400 MHz), Varian VXR400 (400 MHz), Varian I500 (500 MHz), or a Varian I600 (600 MHz) spectrometer. Chemical shifts are reported in ppm from tetramethylsilane with the residual solvent resonance as the internal standard ($CHCl_3$: δ 7.26 ppm or C_7H_9: δ 2.08 ppm). Data are reported as follows: chemical shift, multiplicity (s = singlet, d = doublet, t = triplet, q = quartet, br = broad, m = multiplet), coupling constants (Hz), and integration. ^{13}C NMR spectra were recorded on a Varian I400 (100 MHz), Varian VXR400 (100 MHz), Varian I500 (125 MHz), or a Varian I600 (150 MHz) spectrometer with complete proton decoupling. Chemical shifts are reported in ppm from tetramethylsilane with the solvent resonance as the internal standard ($CDCl_3$: δ 77.16 ppm or C_7D_9: δ20.43). ^{19}F NMR spectra were recorded on Varian VXR400 (375 MHz) spectrometer. High-resolution mass spectrometry (HRMS) was performed on either a Waters/Micromass LCT Classic (ESI-TOF) or a Thermo Electron Corporation MAT 95XP-Trap (GC/MS). Melting points were obtained on a Thomas Hoover capillary melting point apparatus without correction. The diastereomeric and regioisomeric ratios were determined using NMR analysis of unpurified reaction mixtures. GC analyses were performed by means of Agilent 6850 Gas Chromatograph equipped with Agilent 19091Z-413E, 30 m x 320 μm x 0.25 μm column. Helium was used as the GC carrier gas and maintained at a constant flow rate of 25.0 mL/min. The capillary column was held for 1.0 minutes at the initial temperature (60 °C) and subsequently ramped at a rate of 25 °C /min to a final temperature of 300 °C. Total run time was 9.60 min. Unless otherwise noted, all reactions have been carried out with distilled and degassed solvents under an atmosphere of dry N_2 in oven-(135 °C) and flame-dried glassware with standard vacuum-line techniques. Tetrahydrofuran and N,N-Dimethylformamide were purified under a positive pressure of dry argon by passage through two columns of activated alumina. Toluene was purified under a positive pressure of dry argon by passage through columns of activated alumina and Q5 (Grubbs apparatus). All work-up and purification procedures were carried out with reagent grade solvents (purchased from Sigma-Aldrich) in air. Standard column chromatography techniques using ZEOprep 60/40-63 μm silica gel or a CombiFlash Rf 150 with pre-packed silica cartridges were used for purification.

2. Reagents and Catalysts:

((2*R*,3*R*)-6-mesityl-2,3-diphenyl-2,6-dihydroimidazo[1,2-*c*]quinazolin-5(3*H*)-ylidene)copper(III) chloride (chiral NHC-CuCl) was synthesized according to literature procedure.[1]

1-(bromomethyl)-3,5-bis(trifluoromethyl)benzene was purchased from Oakwood and used as received.

1-(*tert*-butyl) 2-methyl (*R*)-5-oxopyrrolidine-1,2-dicarboxylate was purchased from Combi-Blocks and used as received.

1-(*tert*-butyl) 2-methyl (*S*)-5-oxopyrrolidine-1,2-dicarboxylate was purchased from Ambeed and used as received.

1-benzyl-3,4-dihydropyridin-2(1*H*)-one was synthesized according to literature procedure.[2]

1-bromo-2-methylbenzene was purchased from Oakwood and purified *via* neat filtration through a 2 cm pad of dry silica in a 5.75-inch pipet prior to use.
1-bromo-4-(trifluoromethoxy)benzene was purchased from CombiBlocks and purified *via* neat filtration through a 2 cm pad of dry silica in a 5.75-inch pipet prior to use.
1-bromo-4-(trifluoromethyl)benzene was purchased from Oakwood and purified *via* neat filtration through a 2 cm pad of dry silica in a 5.75-inch pipet prior to use.
1-bromo-4-chlorobenzene was purchased from Sigma Aldrich and purified *via* recrystallization from pentane prior to use.
1-bromo-4-fluorobenzene was purchased from TCI America and purified *via* neat filtration through a 2 cm pad of dry silica in a 5.75-inch pipet prior to use.
1-bromonaphthalene was purchased from Oakwood and purified *via* neat filtration through a 2 cm pad of dry silica in a 5.75-inch pipet prior to use.
1-methylimidazole was purchased from Sigma Aldrich and distilled over CaH_2 before use.
2-bromoprop-1-ene was purchased from Oakwood and used as received.
2-methoxypyridine was purchased from Oakwood and distilled over CaH_2 before use.
2-methyl-2-butene was purchased from Oakwood and used as received.
2-picoline was purchased from Oakwood and distilled over CaH_2 before use.
2,5-dihydro-1*H*-pyrrole 2,2,2-trifluoroacetate was synthesized according to literature procedure.[3]
2,6-lutidine was purchased from Oakwood and distilled over CaH_2 before use.
3-bromo-N,N-dimethylaniline was purchased from VWR and purified *via* neat filtration through a 2 cm pad of dry silica in a 5.75-inch pipet prior to use.
4-(4-bromobenzyl)morpholine was synthesized according to literature procedure.[4]
4-(5-bromopyridin-2-yl)morpholine was purchased from CombiBlocks and used as received.
4-bromo-2-methoxypyridine was purchased from Ambeed and purified *via* neat filtration through a 2 cm pad of dry silica in a 5.75-inch pipet prior to use.
4-bromo-2,6-dimethylpyridine was purchased from CombiBlocks and purified *via* neat filtration through a 2 cm pad of dry silica in a 5.75-inch pipet prior to use.
4-bromoaniline was purchased from TCI America and used as received.
4-bromoanisole was purchased from CombiBlocks and purified *via* neat filtration through a 2 cm pad of dry silica in a 5.75-inch pipet prior to use.
4-bromoquinoline was purchased from Ambeed and used as received.
4-Dimethylaminopyridine (DMAP) was purchased from Oakwood and used as received.
4-methyl-3-(trifluoromethyl)aniline was purchased from Matrix Scientific and used as received.
4-phenylpyrrolidin-2-one was purchased from Combi-Blocks and used as received.
4,4-dimethylpyrrolidin-2-one was purchased from Ambeed and used as received.
5-bromo-1-methyl-1*H*-pyrrolo[2,3-*b*]pyridine was synthesized according to literature procedure.[5]
5-bromo-2-methoxypyridine was purchased from CombiBlocks and purified *via* neat filtration through a 2 cm pad of dry silica in a 5.75-inch pipet prior to use.
5-bromobenzofuran was purchased from CombiBlocks and purified *via* neat filtration through a 2 cm pad of dry silica in a 5.75-inch pipet prior to use.
Acetic acid was purchased from EMD and used as received.
Alkylfluor was purchased from Sigma Aldrich and used as received.
Ammonium acetate was purchased from Mallinckrodt, rinsed with acetone, gravity filtered, and dried on vacuum prior to use.

APhos Pd G3 was synthesized according to literature procedure.[6]
benzyl 2,3-dihydro-1*H*-pyrrole-1-carboxylate was synthesized according to literature procedure.[7]
bis(pinacolato)diboron was purchased from Oakwood and recrystallized from pentane before use.
Boc_2O was purchased from Oakwood and used as received.
bromobenzene was purchased from Aldrich and purified *via* neat filtration through a 2 cm pad of dry silica in a 5.75-inch pipet prior to use.
Carbonylchlorohydridotris(triphenylphosphine)ruthenium(II) was purchased from Oakwood and used as received.
Cesium fluoride was purchased from Strem and used as received.
CuCl(1,3-dimesityltetrahydropyrimini-2-ylidene) was synthesized according to literature procedure.[8]
Cyclopentanone was purchased from Oakwood and used as received.
DABCO was purchased from TCI America and used as received.
Dess-Martin Periodinane was purchased from Synthonix and used as received.
Dibromomethane was purchased from Oakwood and used as received.
Ethyl 3-bromobenzoate was purchased from Sigma Aldrich and purified *via* neat filtration through a 2 cm pad of dry silica in a 5.75-inch pipet prior to use.
HATU was purchased from CombiBlocks and used as received.
Hydrogen peroxide (30% aqueous) was purchased from Macron and used as received.
IMes-CuCl was synthesized according to literature procedure.[9]
Iodine was purchased from Alfa Aesar and used as received.
IPr-CuCl was synthesized according to literature procedure.[9]
Lithium bis(trimethylsilyl)amide (1.0 M solution in THF) was purchased from Sigma-Aldrich and used as received.
Lithium *tert*-butoxide was purchased from Strem and used as received.
Lithium Triethylborohydride (1.0 M solution in THF) was purchased from Sigma-Aldrich and used as received.
Methyl 4-oxo-4-phenylbutanoate was purchased from Combi-Blocks and used as received.
***n*-butyllithium** was purchased from Aldrich and titrated before use.
N-methylmorpholine was purchased from Aldrich and distilled over CaH_2 before use.
N,N-diisopropylethylamine was purchased from Oakwood and distilled over CaH_2 before use.
N,N-dimethylacetamide was purchased from Aldrich and used as received.
Nickel(II) chloride ethylene glycol dimethyl ether complex was purchased from Strem and used as received.
Pivaloyl chloride was purchased from Sigma Aldrich and used as received.
Potassium *tert*-butoxide was purchased from Strem and used as received.
Pyridine was purchased from Macron and distilled over CaH_2 before use.
Pyridylidene Cu-Cl was synthesized according to literature procedure.[10]
Quinoline was purchased from Acros and distilled over CaH_2 before use.
RuPhos Pd G3 was synthesized according to literature procedure.[11]
SIMes-CuCl was synthesized according to literature procedure.[12]
SIPr-CuCl was synthesized according to literature procedure.[12]
Sodium cyanoborohydride was purchased from Oakwood and used as received.
Sodium hydride (60% in mineral oil) was purchased from Sigma Aldrich and used as received.

Sodium hydroxide was purchased from Macron and used as received.
Sodium perborate tetrahydrate was purchased from Merck and used as received.
Sodium *tert*-butoxide was purchased from Strem and used as received.
***tert*-butanol** was purchased from VWR and distilled over CaH_2 before use.
***tert*-butyl (4-bromophenyl)(methyl)carbamate** was synthesized according to literature procedure.[13]
***tert*-butyl 2,3-dihydro-1*H*-pyrrole-1-carboxylate** was synthesized according to literature procedure.[2]
***tert*-butyl 2,3,4,5-tetrahydro-1*H*-azepine-1-carboxylate** was synthesized according to literature procedure.[2]
***tert*-butyl 3,4-dihydropyridine-1(2*H*)-carboxylate** was synthesized according to literature procedure.[2]
***tert*-butyl 4-methyl-2,3-dihydro-1*H*-pyrrole-1-carboxylate** was synthesized according to literature procedure.[14]
***tert*-butyl azete-1(2*H*)-carboxylate** was synthesized according to literature procedure.[15]
***tert*-butyllithium** was purchased from Aldrich and titrated before use.
Triethylamine was purchased from EMD and distilled over CaH_2 before use.
Trifluoroacetic acid was purchased from Oakwood and used as received.
Trifluoroacetic acid was purchased from Oakwood and used as received.
Trifluoroacetic anhydride was purchased from Chem Impex and used as received.
Triphenylphosphine was purchased from Oakwood and used as received.
XPhos Pd G3 was synthesized according to literature procedure.[11]

3. Substrate Synthesis

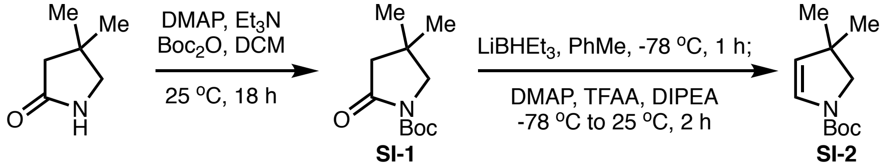

***tert*-butyl 3,3-dimethyl-2,3-dihydro-1*H*-pyrrole-1-carboxylate (SI-2)** was synthesized according to the following modified literature procedures[2,16]: A flame-dried round bottom flask equipped with a stir bar was charged with 4,4-dimethylpyrrolidin-2-one (1.13 g, 10.0 mmol, 1.00 equiv) and evacuated/backfilled with N_2 X 3. DCM (20.0 mL) was then added *via* syringe, followed by triethylamine (1.40 mL, 10.0 mmol, 1.00 equiv.) *via* syringe. DMAP (1.22 g, 10.0 mmol, 1.00 equiv.) and Boc_2O (4.40 g, 20.0 mmol, 2.00 equiv.) were then added portionwise. The reaction was stirred at room temperature for 18 h, and then concentrated. Purification by silica gel chromatography (20% acetone/hexanes) yielded **SI-1** as a white solid, 1.94 g, 91%.

^{1}H NMR (500 MHz, $CDCl_3$) δ 3.45 (s, 2H), 2.32 (s, 2H), 1.52 (s, 9H), 1.16 (s, 6H). **^{13}C NMR (126 MHz, $CDCl_3$)** δ 173.9, 150.4, 82.9, 59.2, 47.9, 31.6, 28.2, 27.3. **IR (neat):** 2965 (w), 1778 (m), 1752 (m), 1710 (m), 1361 (m), 1311 (s), 1255 (m), 1149 (s), 777 (m) cm^{-1}. **HRMS (ESI+):** Calculated for $C_{11}H_{19}O_3NNa$ $[M+Na]^+$: 236.1257, Found: 236.1259.

A flame-dried round bottom flask equipped with a stir bar was charged with *tert*-butyl 4,4-dimethyl-2-oxopyrrolidine-1-carboxylate **SI-1** and evacuated/backfilled with N_2 X 3. Toluene (12.0 mL) was added *via* syringe, and the flask was cooled to -78 °C in a dry ice/acetone bath.

Lithium triethylborohydride (8.80 mL, 1.00 M, 1.10 equiv., 8.80 mmol) was added dropwise *via* syringe. The reaction was stirred at -78 °C for 1 h. N,N-diisopropylethylamine (7.80 mL, 5.70 equiv., 45.6 mmol), and trifluoroacetic anhydride (1.33 mL, 1.20 equiv, 9.60 mmol), were added sequentially *via* syringe, followed by DMAP (9.80 mg, 0.0100 equiv., 0.0800 mmol) as a solid in one portion at -78 °C. The reaction was warmed to room temperature and stirred for 2 h. The reaction was quenched with H_2O (10 mL) and transferred to a separatory funnel. The organic layer was washed 3 X H_2O (10 mL), 1 X brine (10 mL), and was then dried over $MgSO_4$, gravity filtered, and concentrated *in vacuo*. Purification by silica gel chromatography (5% ethyl acetate/hexanes) followed by recrystallization from hexanes yielded **SI-2** as a white solid, 545 mg, 34%.

^{1}H NMR (500 MHz, $CDCl_3$, *1.3:1 mixture of rotamers*) δ 6.49 (appt. s, 0.4H, *minor rotamer*), 6.35 (d, J = 4.2 Hz, 0.6H, *major rotamer*), 4.90 (appt. s, 0.4H, *minor rotamer*), 4.85 (d, J = 4.1 Hz, 0.6H, *major rotamer*), 3.43 (s, 1.2H, *major rotamer*), 3.38 (s, 0.8H, *minor rotamer*), 1.48 (d, J = 1.0 Hz, 9H), 1.13 (s, 6H). **^{13}C NMR (126 MHz, $CDCl_3$, *mixture of rotamers*)** δ 151.7, 127.5, 118.8, 80.2, 80.1, 59.6, 58.9, 43.4, 42.3, 28.8, 28.6. **IR (neat):** 3117 (w), 3004 (w), 1695 (s), 1612 (m), 1402 (s), 1383 (s), 1362 (s), 1350 (s), 1168 (m), 1118 (s), 888 (m), 762 (m) cm^{-1}. **HRMS (EI+):** Calculated for $C_{11}H_{19}NO_2$ $[M]^+$: 197.1416, Found: 197.1416.

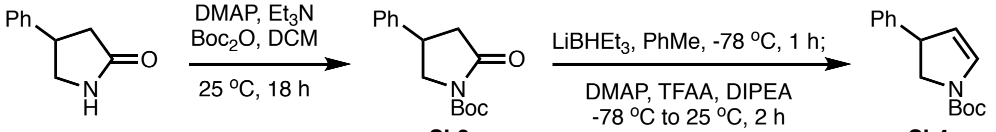

SI-3 **SI-4**

***tert*-butyl 3-phenyl-2,3-dihydro-1*H*-pyrrole-1-carboxylate (SI-4)** was synthesized according to the following modified literature procedures[2,16]: A flame-dried round bottom flask equipped with a stir bar was charged with 4-phenylpyrrolidin-2-one (1.61 g, 10.0 mmol, 1.00 equiv.) and evacuated/backfilled with N_2 X 3. DCM (20.0 mL) was then added *via* syringe, followed by triethylamine (1.40 mL, 10.0 mmol, 1.00 equiv.) *via* syringe. DMAP (1.22 g, 10.0 mmol, 1.00 equiv) and Boc_2O (4.40 g, 20.0 mmol, 2.00 equiv.) were then added portionwise. The reaction was stirred at room temperature for 18 h, and then concentrated. Purification by silica gel chromatography (20% acetone/hexanes) yielded **SI-3** as a white solid, 2.33 g, 89%.

^{1}H NMR (400 MHz, $CDCl_3$) δ 7.41 – 7.22 (m, 5H), 4.16 (dd, J = 10.8, 8.2 Hz, 1H), 3.70 (dd, J = 10.8, 8.5 Hz, 1H), 3.54 (p, J = 8.6 Hz, 1H), 2.90 (dd, J = 17.3, 8.5 Hz, 1H), 2.72 (dd, J = 17.3, 10.0 Hz, 1H), 1.54 (s, 9H). **^{13}C NMR (126 MHz, $CDCl_3$)** δ 173.2, 150.1, 140.7, 129.2, 127.6, 126.9, 83.2, 53.3, 40.5, 36.6, 28.2. **IR (neat):** 3058 (w), 2973 (w), 1775 (s), 1692 (w), 1359 (m), 1310 (s), 1150 (s), 1019 (m), 795 (m), 697 (s) cm^{-1}. **HRMS (ESI+):** Calculated for $C_{15}H_{19}O_3NNa$ $[M+Na]^+$: 284.1257, Found: 284.1260.

A flame-dried round bottom flask equipped with a stir bar was charged with *tert*-butyl 2-oxo-4-phenylpyrrolidine-1-carboxylate **SI-3** and evacuated/backfilled with N_2 X 3. Toluene (11.0 mL) was added *via* syringe, and the flask was cooled to -78 °C in a dry ice/acetone bath. Lithium triethylborohydride (8.40 mL, 1.00 M, 1.10 equiv., 8.40 mmol) was added dropwise *via* syringe. The reaction was stirred at -78 °C for 1 h. N,N-diisopropylethylamine (7.60 mL, 5.70 equiv., 43.9 mmol), and trifluoroacetic anhydride (1.30 mL, 1.20 equiv., 9.20 mmol), were added sequentially *via* syringe, followed by DMAP (9.00 mg, 0.0100 equiv., 0.0770 mmol) as a solid in one portion at -78 °C. The reaction was warmed to room temperature and stirred for 2 h. The reaction was

quenched with H_2O (10 mL) and transferred to a separatory funnel. The organic layer was washed 3 X H_2O (10 mL), 1 X brine (10 mL), and was then dried over $MgSO_4$, gravity filtered, and concentrated *in vacuo*. Purification by silica gel chromatography (5% ethyl acetate/hexanes) yielded **SI-4** as a white solid, 1.41 g, 75%.

^{1}H NMR (600 MHz, $CDCl_3$, *1.1:1 mixture of rotamers*) δ 7.38-7.28 (m, 2H), 7.25 – 7.18 (m, 3H), 6.80 (s, 0.5H, *rotamer A*), 6.64 (s, 0.5H, *rotamer B*), 5.12 (s, 0.5H, *rotamer A*), 5.07 (s, 0.5H, *rotamer B*), 4.27 – 4.04 (m, 2H), 3.75 – 3.61 (m, 0.5H, *rotamer A*), 3.61-3.49 (m, 0.5H, *rotamer B*), 1.51 (s, 5H, *rotamer A*), 1.48 (s, 4H, *rotamer B*). **^{13}C NMR (126 MHz, $CDCl_3$, *mixture of rotamers*)** δ 150.1, 144.3, 130.7, 130.4, 128.8, 127.4, 127.0, 111.5, 111.3, 80.5, 80.4, 54.4, 53.7, 48.5, 47.3, 28.6. **IR (neat):** 3101 (w), 2976 (w), 1698 (s), 1614 (m), 1408 (s), 1353 (s), 1134 (s), 887 (m), 760 (s), 702 (s) cm^{-1}. **HRMS (EI+):** Calculated for $C_{15}H_{19}NO_2$ $[M]^+$: 245.1416, Found: 245.1413.

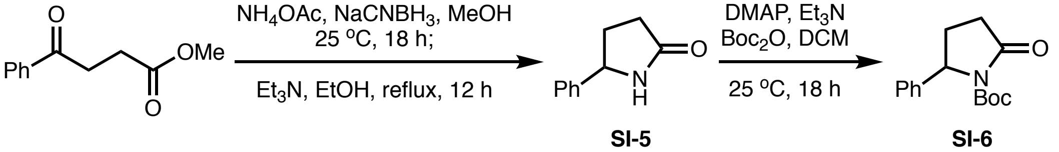

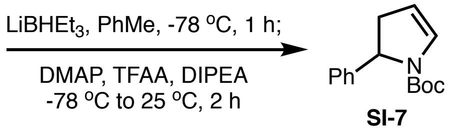

***tert*-butyl 2-phenyl-2,3-dihydro-1*H*-pyrrole-1-carboxylate (SI-7)** was synthesized according to the following modified literature procedures[17,18,16,2]: A flame-dried round bottom flask equipped with a stir bar under N_2 atmosphere was charged with methyl 4-oxo-4-phenylbutanoate (0.96 g, 5.00 mmol, 1.00 equiv.) and MeOH (19.0 mL) sequentially *via* syringe. NH_4OAc (3.85 g, 50.0 mmol, 10.0 equiv.) was added in one portion, followed by $NaCNBH_3$ (628 mg, 20.0 mmol, 2.00 equiv.) in one portion, and the reaction was stirred at room temperature for 18 hours. The reaction was quenched with H_2O (2 mL) and basified to pH 11 with sat. aq. Na_2CO_3. The aqueous layer was extracted with Et_2O (3 X 20 mL), and the combined organic layers were dried over Na_2SO_4, gravity filtered, and concentrated *in vacuo*. The crude material was then lactamized by dissolving in EtOH (5.00 mL) and adding Et_3N (3.50 mL, 25.0 mmol, 5.00 equiv.) *via* syringe. A reflux condenser was attached and the mixture was stirred at reflux for 12 h. The reaction was cooled to room temperature and concentrated *in vacuo*. The material was then diluted with Et_2O (10 mL) and 1 M HCl (10 mL) and the aqueous layer was extracted Et_2O (3 X 10 mL). The combined organic layers were dried over $MgSO_4$, gravity filtered, and concentrated *in vacuo*. Purification *via* MPLC (gradient: 0-2% MeOH/DCM) yielded 206 mg **SI-5** as a white solid, 26% (a second run of this reaction on 4.76 mmol scale yielded 197 mg white solid, 26%).

^{1}H NMR (500 MHz, $CDCl_3$) δ 7.35 – 7.29 (m, 2H), 7.28 – 7.22 (m, 3H), 6.67 (s, 1H), 4.71 (t, J = 7.1 Hz, 1H), 2.57 – 2.47 (m, 1H), 2.47 – 2.29 (m, 2H), 1.97 – 1.86 (m, 1H). **^{13}C NMR (126 MHz, $CDCl_3$)** δ 178.8, 142.7, 129.0, 127.9, 125.7, 58.2, 31.4, 30.4.

^{1}H and ^{13}C NMR data matched that reported in the literature.[17]

A flame-dried round bottom flask equipped with a stir bar was charged with 5-phenylpyrrolidin-2-one **SI-5** (206 mg, 1.28 mmol, 1.00 equiv.) and evacuated/backfilled with N_2 X 3. DCM (3.00 mL) was then added *via* syringe, followed by triethylamine (0.180 mL, 1.28 mmol, 1.00 equiv.) *via* syringe. DMAP (156 mg, 1.28 mmol, 1.00 equiv.) and Boc_2O (559 mg, 2.56 mmol, 2.00 equiv.) were then added portionwise. The reaction was stirred at room temperature for 18 h, and then concentrated. Purification by silica gel chromatography (20% acetone/hexanes) yielded **SI-6** as a white solid, 303 mg, 90% (a second run of this reaction on 1.22 mmol scale yielded 294 mg white solid, 92%).

^{1}H NMR (500 MHz, $CDCl_3$) δ 7.35 (t, *J* = 7.9 Hz, 2H), 7.31 – 7.25 (m, 1H), 7.21 (d, *J* = 8.5 Hz, 2H), 5.13 (dd, *J* = 8.3, 4.0 Hz, 1H), 2.73 – 2.60 (m, 1H), 2.58 – 2.41 (m, 2H), 1.95 – 1.83 (m, 1H), 1.26 (s, 9H). **^{13}C NMR (126 MHz, $CDCl_3$)** δ 174.9, 149.7, 142.7, 128.9, 127.7, 125.2, 83.0, 61.8, 31.4, 27.8, 27.6

^{1}H and ^{13}C NMR data matched that reported in the literature.[19]

A flame-dried round bottom flask equipped with a stir bar was charged with *tert*-butyl 2-oxo-5-phenylpyrrolidine-1-carboxylate **SI-6** (597 mg, 2.28 mmol, 1.00 equiv.) and evacuated/backfilled with N_2 X 3. Toluene (3.50 mL) was added *via* syringe, and the flask was cooled to -78 °C in a dry ice/acetone bath. Lithium triethylborohydride (2.51 mL, 1.00 M, 1.10 equiv., 2.51 mmol) was added dropwise *via* syringe. The reaction was stirred at -78 °C for 1 h. N,N-diisopropylethylamine (2.27 mL, 5.70 equiv., 13.0 mmol), and trifluoroacetic anhydride (387 μL, 1.20 equiv, 2.74 mmol), were added sequentially *via* syringe, followed by DMAP (2.79 mg, 0.0100 equiv., 0.0228 mmol) as a solid in one portion at -78 °C. The reaction was warmed to room temperature and stirred for 2 h. The reaction was quenched with H_2O (4 mL) and transferred to a separatory funnel. The organic layer was washed 3 X H_2O (4 mL), 1 X brine (4 mL), and was then dried over $MgSO_4$, gravity filtered, and concentrated *in vacuo*. Purification by MPLC (gradient: 0-5% ethyl acetate/hexanes) yielded 358 mg **SI-7** as a white solid, 64%.

^{1}H NMR (500 MHz, $CDCl_3$, *1.8:1 mixture of rotamers*) δ 7.23 (t, *J* = 7.5 Hz, 2H), 7.20-7.12 (m, 3H), 6.72 (s, 0.6H, *major rotamer*), 6.57 (s, 0.4H, *minor rotamer*), 5.08 (d, *J* = 11.2 Hz, 0.4H, *minor rotamer*), 4.99 – 4.81 (m, 1.6H, *contains major rotamer*), 3.16 (t, *J* = 14.0 Hz, 1H), 2.48 (d, *J* = 16.9 Hz, 0.6H, *major rotamer*), 2.39 (d, *J* = 17.3 Hz, 0.4H, *minor rotamer*), 1.37 (s, 3H, *minor rotamer*), 1.11 (s, 6H, *major rotamer*). **^{13}C NMR (126 MHz, $CDCl_3$, *mixture of rotamers*)** δ 152.4, 151.7, 145.1, 144.3, 130.4, 128.7, 128.5, 127.1, 125.6, 125.5, 105.6, 80.4, 80.2, 60.8, 60.2, 40.7, 39.8, 28.5, 28.1. **IR (neat):** 3090 (w), 2978 (w), 1686 (s), 1619 (w), 1399 (s), 1365 (m), 1329 (m), 1435 (s), 758 (s), 721 (m), 698 (s), 444 (m) cm^{-1}. **HRMS (ESI+):** Calculated for $C_{15}H_{19}O_2NNa$ $[M+Na]^+$: 268.1308, Found: 268.1310.

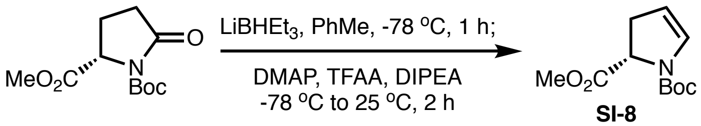

SI-8

1-(*tert*-butyl) 2-methyl (*S*)-2,3-dihydro-1*H*-pyrrole-1,2-dicarboxylate (SI-8) was synthesized according to the following modified literature procedure[2]: A flame-dried round bottom flask equipped with a stir bar was charged with 1-(*tert*-butyl) 2-methyl (*S*)-5-oxopyrrolidine-1,2-dicarboxylate (2.43 g, 10.0 mmol, 1.00 equiv.) and evacuated/backfilled with N_2 X 3. Toluene

(14.0 mL) was added *via* syringe, and the flask was cooled to -78 °C in a dry ice/acetone bath. Lithium triethylborohydride (11.0 mL, 1.00 M, 1.10 equiv., 11.0 mmol) was added dropwise *via* syringe. The reaction was stirred at -78 °C for 1 h. N,N-Diisopropylethylamine (9.90 mL, 5.70 equiv., 57.0 mmol), and trifluoroacetic anhydride (1.67 mL, 1.20 equiv, 12.0 mmol), were added sequentially *via* syringe, followed by DMAP (12 mg, 0.0100 equiv., 0.0100 mmol) as a solid in one portion at -78 °C. The reaction was warmed to room temperature and stirred for 2 h. The reaction was quenched with H_2O (15 mL) and transferred to a separatory funnel. The organic layer was washed 3 X H_2O (15 mL), 1 X brine (15 mL), and was then dried over $MgSO_4$, gravity filtered, and concentrated *in vacuo*. Purification by silica gel chromatography (10% acetone/hexanes + 1% Et_3N), followed by MPLC (gradient: 0-10% ethyl acetate/hexanes) yielded 763 mg **SI-8** as a colorless oil, 34%. The enantiomeric purity was established *via* HPLC analysis using a chiral column. A racemic sample was obtained by preparing a 1:1 mixture of **SI-8** and **SI-9**.

^{1}H NMR (500 MHz, $CDCl_3$, *1.1:1 mixture of rotamers*) δ 6.65 (s, 0.5H, *rotamer A*), 6.51 (s, 0.5H, *rotamer B*), 4.95 (s, 0.5H, *rotamer A*), 4.90 (s, 0.5H, *rotamer B*), 4.66 (dd, *J* = 12.1, 5.1 Hz, 0.5H, *rotamer A*), 4.58 (dd, *J* = 12.0, 5.4 Hz, 0.5H, *rotamer B*), 3.75 (s, 3H), 3.13 – 2.97 (m, 1H), 2.75 – 2.57 (m, 1H), 1.48 (s, 4H, *rotamer A*), 1.43 (s, 5H, *rotamer B*). **^{13}C NMR (126 MHz, $CDCl_3$)** δ 172.6, 172.4, 151.6, 130.2, 130.1, 105.2, 105.1, 81.2, 81.00, 58.5, 57.9, 52.5, 52.3, 35.6, 34.4, 28.5, 28.3. **IR (neat):** 3117 (w), 2977 (w), 1754 (m), 1699 (s), 1393 (s), 1365 (m), 1202 (m), 1177 (m), 1136 (s), 905 (m), 762 (m), 701 (m) cm^{-1}. **HRMS (ESI+):** Calculated for $C_{11}H_{17}O_4NNa$ $[M+Na]^+$: 250.1050, Found: 250.1051. **HPLC:** Lux 3u amylose-2, 22 °C. 0.5 mL/min, Hex/IPA 95:5, 254 nm, t_R = 20.039 min, t_s = 23.206 min, >99:1 er. See Supporting Information part 14 for HPLC chromatographs.

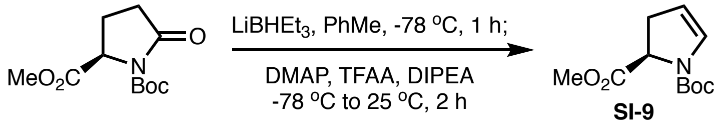

SI-9

1-(*tert*-butyl) 2-methyl (*R*)-2,3-dihydro-1*H*-pyrrole-1,2-dicarboxylate (SI-9) was synthesized according to the following modified literature procedure[2]: A flame-dried round bottom flask equipped with a stir bar was charged with 1-(*tert*-butyl) 2-methyl (*R*)-5-oxopyrrolidine-1,2-dicarboxylate (1.20 g, 5.00 mmol, 1.00 equiv.) and evacuated/backfilled with N_2 X 3. Toluene (7.50 mL) was added *via* syringe, and the flask was cooled to -78 °C in a dry ice/acetone bath. Lithium triethylborohydride (5.50 mL, 1.00 M, 1.10 equiv., 5.50 mmol) was added dropwise *via* syringe. The reaction was stirred at -78 °C for 1 h. N,N-Diisopropylethylamine (5.00 mL, 5.70 equiv., 29.0 mmol), and trifluoroacetic anhydride (0.850 mL, 1.20 equiv, 6.00 mmol), were added sequentially *via* syringe, followed by DMAP (6.10 mg, 0.0100 equiv., 0.00500 mmol) as a solid in one portion at -78 °C. The reaction was warmed to room temperature and stirred for 2 h. The reaction was quenched with H_2O (8 mL) and transferred to a separatory funnel. The organic layer was washed 3 X H_2O (8 mL), 1 X brine (8 mL), and was then dried over $MgSO_4$, gravity filtered, and concentrated *in vacuo*. Purification by MPLC (gradient: 0-10% ethyl acetate/hexanes) yielded 566 mg **SI-9** as a colorless oil, 50%. The enantiomeric purity was established *via* HPLC analysis using a chiral column. A racemic sample was obtained by preparing a 1:1 mixture of **SI-9** and **SI-8**.

^{1}H NMR (500 MHz, $CDCl_3$, *1.1:1 mixture of rotamers*) δ 6.65 (s, 0.5H, *rotamer A*), 6.52 (s, 0.5H, *rotamer B*), 4.96 (s, 0.5H, *rotamer A*), 4.91 (s, 0.5H, *rotamer B*), 4.66 (dd, J = 11.8, 5.0 Hz, 0.5H, *rotamer A*), 4.59 (dd, J = 12.1, 5.4 Hz, 0.5H, *rotamer B*), 3.76 (s, 3H), 3.16 – 2.98 (m, 1H), 2.77 – 2.57 (m, 1H), 1.49 (s, 4H, *rotamer A*), 1.44 (s, 5H, *rotamer B*). **^{13}C NMR** (126 MHz, $CDCl_3$) δ 172.5, 172.3, 151.5, 130.1, 130.0, 105.2, 105.1, 81.1, 80.9, 58.4, 57.9, 52.4, 52.2, 35.5, 34.4, 28.4, 28.3. **HPLC:** Lux 3u amylose-2, 22 °C. 0.5 mL/min, Hex/IPA 95:5, 254 nm, t_R = 20.039 min, t_s = 23.206 min, >99:1 er. See Supporting Information part 14 for HPLC chromatographs.

^{1}H and ^{13}C NMR data matches that from **SI-8**.

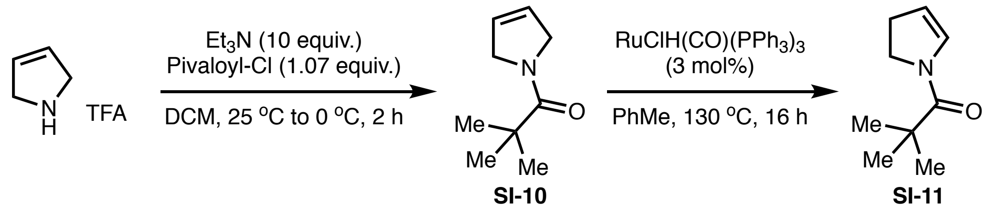

1-(2,3-dihydro-1*H*-pyrrol-1-yl)-2,2-dimethylpropan-1-one (SI-11) was synthesized according to the following modified literature procedures[3,20]: An oven-dried round bottom flask equipped with a stir bar was charged with 2,5-dihydro-1*H*-pyrrole 2,2,2-trifluoroacetate (1.83 g, 1.00 equiv., 10.0 mmol), followed by DCM (77.0 mL) and triethylamine (13.9 mL, 10.0 equiv., 100 mmol) sequentially *via* syringes. The reaction was stirred at room temperature for 1 h, then cooled to 0 °C in an ice water bath. Pivaloyl-Cl was then added dropwise as a solution in 8.00 mL of DCM *via* syringe, and the reaction was stirred at 0 °C for 1 h. The reaction was quenched with saturated aqueous $NaHCO_3$ (50 mL), the layers were separated, and the aqueous layer was extracted with DCM (3 X 50 mL). The combined organic layers were dried over $MgSO_4$, gravity filtered, and concentrated *in vacuo*. Purification by silica gel chromatography (20% acetone in hexanes) yielded 1.08 g **SI-10** as a light brown oil, 71%.

^{1}H NMR (500 MHz, $CDCl_3$, *mixture of rotamers*) δ 5.92 – 5.70 (br m, 2H), 4.41 (br s, 2H), 4.29 (br s, 2H), 1.27 (s, 9H). **^{13}C NMR (126 MHz, $CDCl_3$, *mixture of rotamers*)** δ 176.4, 125.8, 125.4, 56.0, 53.9, 39.1, 27.5. **IR (neat):** 2967 (w), 2865 (w), 2237 (w), 1638 (m), 1607 (s), 1405 (s), 1380 (s), 1186 (m), 720 (s), 671 (s) cm^{-1}. **HRMS (EI+):** Calculated for $C_9H_{15}NO$ [M^+]: 153.1146, found: 153.1145.

An oven-dried reaction tube equipped with a stir bar was brought into an Ar-filled glovebox and charged with carbonylchlorohydridotris(triphenylphosphine)ruthenium(II) (241 mg, 3.00 mol%, 0.253 mmol) and sealed with a septum, then removed from the glovebox. 1-(2,5-dihydro-1*H*-pyrrol-1-yl)-2,2-dimethylpropan-1-one **SI-10** (1.29 g, 1.00 equiv., 8.42 mmol) was added to the vial as a solution in 14.0 mL toluene *via* syringe. The septum was quickly replaced with a screw cap and the reaction was stirred at 130 °C for 16 h. The reaction was cooled to room temperature, filtered through celite, rinsing with ethyl acetate, and the solution was then concentrated *in vacuo*. Purification *via* MPLC (gradient: 0-10% ethyl acetate in hexanes) yielded a yellow solid, which was recrystallized from hexanes to yield 486 mg white crystalline solid **SI-11**, 38%.

^{1}H NMR (400 MHz, $CDCl_3$) δ 6.84 (br s, 1H), 5.19 (dt, J = 4.4, 2.7 Hz, 1H), 3.88 (app. t, J = 8.9 Hz, 2H), 2.56 (br s, 2H), 1.27 (s, 9H). **^{13}C NMR (126 MHz, $CDCl_3$)** δ 174.3, 129.8, 111.0, 47.2,

39.0, 27.8. **IR (neat):** 3128 (w), 2951 (w), 1605 (s), 1409 (s), 1198 (m), 840 (m), 704 (s), 470 (m) cm^{-1}. **HRMS (ESI+):** Calculated for $C_9H_{16}ON$ $[M+H]^+$: 154.1226, found: 176.1045.

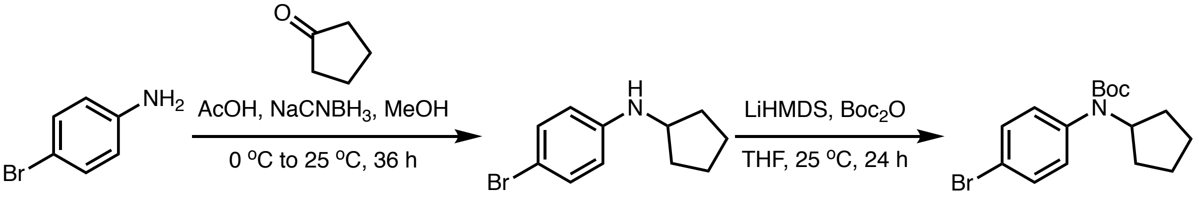

SI-12 53

***tert*-butyl (4-bromophenyl)(cyclopentyl)carbamate (53)** was synthesized according to the following modified literature procedures[21,22]: An oven-dried round bottom flask equipped with a stir bar was charged with 4-bromoaniline (3.44 g, 1.00 equiv., 20.0 mmol) and evacuated/backfilled with N_2 X 3. Added methanol (30.0 mL), acetic acid (572 µL, 0.500 equiv., 10.0 mmol), and cyclopentanone (17.7 mL, 10.0 equiv., 200 mmol) sequentially *via* syringes. The reaction was cooled to 0 °C in an ice bath, and then sodium cyanoborohydride (1.89 g, 1.50 equiv., 30.0 mmol) was added in small portions over 5 minutes. The reaction was warmed to room temperature and stirred under N_2 atmosphere overnight. After 18 hours, TLC showed remaining aniline starting material. The reaction was re-cooled to 0 °C and sodium cyanoborohydride (628 mg, 0.500 equiv., 10.0 mmol) was added in small portions. The reaction was warmed to rt and stirred for another 18 h. The reaction was concentrated *in vacuo*, then the crude material was dissolved in ethyl acetate (30 mL). The organic layer was washed 1 X H_2O (30 mL), 1 X brine (30 mL), dried over Na_2SO_4, gravity filtered, and concentrated *in vacuo*. Purification by silica gel chromatography (5% ethyl acetate/hexanes) yielded 3.75 g **SI-12** as a colorless oil, 78%.

^{1}H NMR (500 MHz, $CDCl_3$) δ 7.13 (d, J = 8.6 Hz, 2H), 6.37 (d, J = 8.1 Hz, 2H), 3.63 (p, J = 6.3 Hz, 1H), 3.57 (s, 1H), 1.91 (dq, J = 13.0, 6.4 Hz, 2H), 1.69 – 1.58 (m, 2H), 1.58-1.48 (m, 2H), 1.35 (dq, J = 12.9, 6.3 Hz, 2H). **^{13}C NMR** (126 MHz, $CDCl_3$) δ 147.1, 131.9, 114.8, 108.4, 54.7, 33.5, 24.1. **IR (neat):** 3408 (w), 3020 (w), 2953 (m), 2865 (w), 1591 (m), 1492 (s), 1311 (m), 1176 (m), 1072 (w), 800 (s), 502 (m) cm^{-1}. **HRMS (ESI+):** Calculated for $C_{11}H_{15}NBr$ $[M+H]^+$: 240.0382, found: 240.0383.

An oven-dried round bottom flask was charged with 4-bromo-N-cyclopentylaniline **SI-12** (3.46 g, 1.00 equiv., 14.4 mmol) and evacuated/backfilled with N_2 X 3. THF (85.0 mL) was added *via* syringe, followed by lithium bis(trimethylsilyl)amide (20.2 mL, 1.00 molar, 1.40 equiv., 20.2 mmol) dropwise at room temperature *via* syringe. The reaction was stirred for 30 minutes at room temperature, then Boc_2O (7.86 g, 2.50 equiv., 36.0 mmol) was added dropwise as a solution in 10.0 mL THF *via* syringe. The reaction was stirred at room temperature for 24 h, and then concentrated *in vacuo*. Purification by silica gel chromatography (gradient: 2-5% Et_2O/hexanes), followed by a second purification *via* MPLC (gradient: 0-5% ethyl acetate/hexanes) yielded 4.1 g **53** as a white solid, 84%.

^{1}H NMR (500 MHz, $CDCl_3$) δ 7.45 (d, J = 7.4 Hz, 2H), 6.95 (d, J = 7.4 Hz, 2H), 4.44 (p, J = 8.8 Hz, 1H), 1.94-1.81 (m, 2H), 1.59 – 1.45 (m, 4H), 1.42 – 1.30 (m, 11H). **^{13}C NMR (126 MHz, $CDCl_3$)** δ 155.0, 139.4, 131.9, 131.7, 120.7, 80.2, 59.1, 30.2, 28.5, 23.0. **IR (neat):** 3005 (w), 2970 (w), 2872 (w), 1687(s), 1488 (m), 1393 (m), 1337 (s), 1312 (s), 1161 (s), 1005 (s), 767 (s) cm^{-1}. **HRMS (ESI+):** Calculated for $C_{16}H_{22}O_2NBrNa$ $[M+Na]^+$: 362.0762, found: 362.0728.

4. Cu/Pd Optimization Studies

General Procedure: In an N_2-filled glovebox, to an oven-dried 13 x 100 mm screw-capped reaction vial equipped with a stir bar was added L-Pd G3 (0.00200 mmol, 1.00 mol%), L-CuCl (0.0100 mmol, 5.00 mol%), MO^{t}Bu (0.300 mmol, 1.50 equiv.), and bis(pinacolato)diboron, (81.3 mg, 0.320 mmol, 1.60 equiv.). The reaction vial was sealed with a septum and removed from the glovebox. A solution of *tert*-butyl azete-1(2*H*)-carboxylate (36.0 µL, 0.210 mmol, 1.02 equiv.), bromobenzene (34 µL, 0.315 mmol, 1.58 equiv.), and additive (0.105 mmol, 0.525 equiv.) (if applicable) in solvent (2.10 mL) was prepared in a separate oven-dried 3-dram vial under N_2. 2.00 mL (0.200 mmol, 1.00 equiv. enecarbamate; 0.300 mmol, 1.50 equiv. PhBr, 0.100 mmol, 0.500 equiv. N-Me-morpholine) of this solution was added to the reaction vial *via* syringe, the septum was sealed with electrical tape, and the reaction was stirred at 30 °C in an aluminum block for 18 h. The reaction was quenched with H_2O (3 mL) and the mixture was extracted with EtOAc (3 X 3 mL). The combined organic layers were dried over $MgSO_4$, gravity filtered, and concentrated *in vacuo*. Mesitylene was added as an internal standard, and a small aliquot of the reaction mixture was analyzed *via* ^{1}H NMR.

Discussion of results: The optimization table for the Cu/Pd-catalyzed arylboration of N-Boc-2-azetine is presented in **Table S1**. It was found that the combination of RuPhos-Pd G3 and Pyridylidene-CuCl were superior to other Pd and Cu catalysts screened (**Table S1**, entries 2-8). Additionally, the 10:1 PhMe/THF solvent mixture performed better than PhMe alone (**Table S1**, entry 9), and NaO^{t}Bu was found to be comparable to KO^{t}Bu, while LiO^{t}Bu was inferior (**Table S1**, entries 10-11).

Table S1. Change from standard conditions table

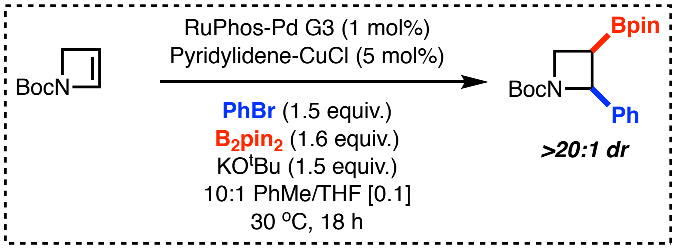

Entry	Change from above	% pdt	rr
1	none	63	>20:1
2	SIMesCuCl	17	8:1
3	IMesCuCl	10	9:1
4	SIPrCuCl	38	4:1
5	IPrCuCl	43	4:1
6	"6-NHC"-CuCl	26	12:1
7	APhos	12	11:1
8	XPhos	38	12:1
9	PhMe only	16	15:1
10	NaOtBu	63	>20:1
11	LiOtBu	36	35:1

Reactions run on 0.2 mmol scale. Crude yields & dr's determined by ^{1}H NMR relative to mesitylene

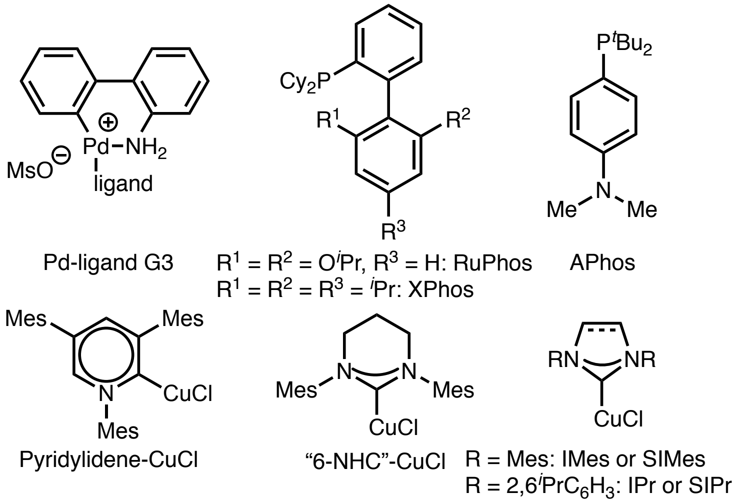

Additional additives were also screened during the optimization of the Cu/Pd-catalyzed arylboration of N-Boc-2-azetine (**Table S2**). It was found that a variety of substituted pyridines and quinoline (**7-8**, **SI-13-14**) performed similarly to one another, while pyridine **6** and DMAP **SI-15** resulted in diminished yield. Additionally, N-Me-imidazole **SI-16** lowered the yield and triphenylphosphine **SI-17** resulted in no reaction at both 5 and 50 mol% loading. While triethylamine **9** and DABCO **10** performed similarly to each other as well as **7-8**, and **SI-13-14**, N-methyl-morpholine **11** was found to be the optimal N-additive, outperforming the others at both 5 and 50 mol% loading. 50 mol% was chosen for ease and accuracy of reaction setup.

Table S2. Screening of additional additives

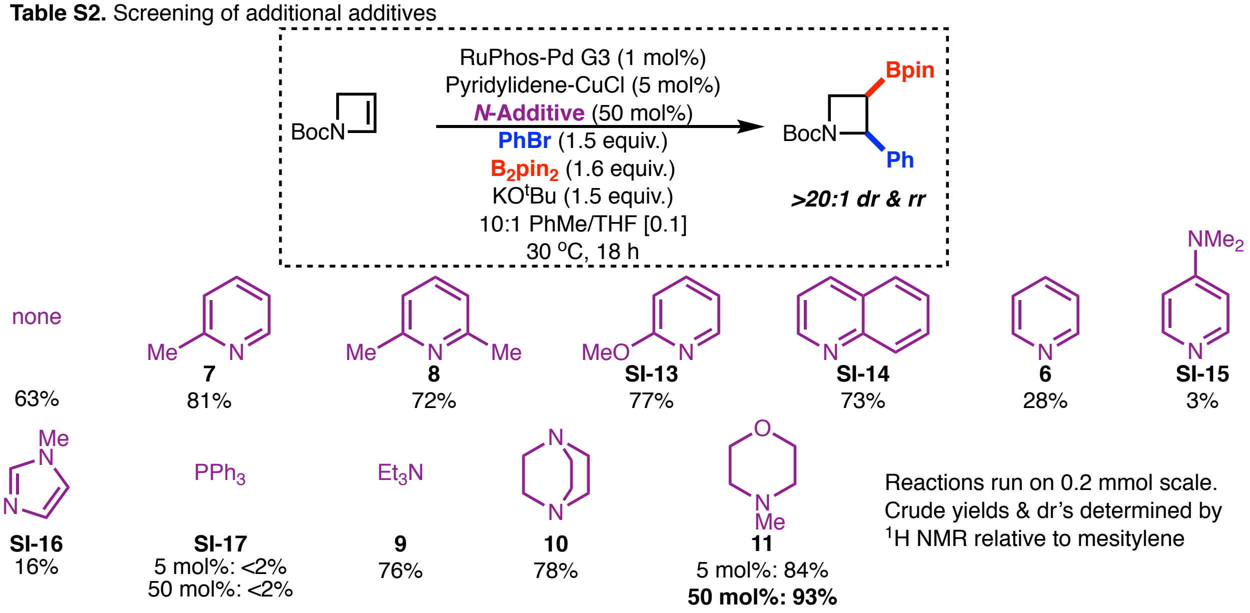

5. General Procedure A: Cu/Pd-Catalyzed Arylboration of Enecarbamates

In an N_2-filled glovebox, to an oven-dried 16 x 100 mm screw-capped reaction vial equipped with a stir bar was added RuPhosPd G3 (4.20 mg, 0.00500 mmol, 1.00 mol%), Pyridylidene-CuCl (13.3 mg, 0.025 mmol, 5.00 mol%), potassium *tert*-butoxide (84.2 mg, 0.750 mmol, 1.50 equiv.), and bis(pinacolato)diboron, (203 mg, 0.800 mmol, 1.60 equiv.). The reaction vial was sealed with a septum and removed from the glovebox. A solution of *tert*-butyl azete-1(2*H*)-carboxylate (88.0 μL, 0.510 mmol, 1.02 equiv.), arylbromide (0.765 mmol, 1.53 equiv.), and N-Me-morpholine (29.0 μL, 0.255 mmol, 0.510 equiv.) (if ArBr did not contain a pyridine or tertiary aliphatic nitrogen atom) in 10:1 Toluene/THF (5.10 mL) was prepared in a separate oven-dried 3-dram vial under N_2. 5.00 mL (0.500 mmol, 1.00 equiv. enecarbamate; 0.75 mmol, 1.50 equiv. ArBr, 0.250 mmol, 0.500 equiv. N-Me-morpholine) of this solution was added to the reaction vial *via* syringe, the septum was sealed with electrical tape, and the reaction was stirred at 30 °C in an aluminum block for 18 h. The reaction was quenched with H_2O (4 mL) and the mixture was extracted with EtOAc (3 X 6 mL). The combined organic layers were dried over $MgSO_4$, gravity filtered, and concentrated *in vacuo*. Mesitylene was added as an internal standard, and a small aliquot of the reaction mixture was analyzed *via* ^{1}H NMR. The crude material was either oxidized *via* General Procedure C, or purified directly *via* silica gel chromatography or MPLC.

6. General Procedure B: Ni-Catalyzed Arylboration of Enecarbamates

In an N_2-filled glovebox, to an oven-dried 16 x 100 mm screw-capped reaction vial equipped with a stir bar was added bis(pinacolato)diboron, (254 mg, 1.00 mmol, 2.00 equiv.), and NaO^{t}Bu (72.0 mg, 0.750 mmol, 1.50 equiv.). Note: if arylbromide (0.750 mmol, 1.50 equiv.) or enecarbamate (0.500 mmol, 1.00 equiv.) was a solid, it was added to the reaction vial in the glovebox. To a separate oven-dried 2-dram vial, Ni(DME)Cl_2 (6.9 mg, 0.031 mmol, 0.06 equiv.) was added. Both vials were sealed with septa and removed from the glovebox. THF (4.00 mL) was added to the reaction vial, followed by arylbromide (0.750 mmol, 1.50 equiv.), and enecarbamate (0.500 mmol, 1.00 equiv.) (if liquids) sequentially *via* syringes. The reaction was then cooled to 0 °C in an ice-water bath. DMA (1.25 mL) was added to the vial containing Ni(DME)Cl_2 (0.0250 M in Ni(DME)Cl_2) to prepare the catalyst solution. 1.00 mL of the catalyst solution was then added to the reaction vial (5 mol% catalyst loading) at 0 °C. The septum on the reaction vial was then quickly replaced by a Teflon-lined screw cap and the reaction was stirred at 2-10 °C for 18 h in a temperature-controlled cold room. The reaction was quenched upon the addition of H_2O (4 mL) and the mixture was extracted with EtOAc (3 X 6 mL). The combined organic layers were dried over $MgSO_4$, gravity filtered, and concentrated *in vacuo*. Mesitylene or 1,3,5-trimethoxybenzene was added as an internal standard, and a small aliquot of the reaction mixture was analyzed *via* ^{1}H NMR. The crude material was then re-dissolved in 1:1 EtOAc/hexanes (4 mL) and washed with 1 M KOH (3 X 4 mL), dried over $MgSO_4$, gravity filtered, and concentrated *in vacuo.* The crude material was then purified *via* silica gel chromatography or MPLC.

7. General Procedure C: Sodium Perborate Oxidation of Pinacol Boronic Esters

To a 25-mL round bottom flask containing the crude arylboration reaction mixture (0.500 mmol) was added THF (2.50 mL) and H_2O (2.50 mL) sequentially *via* syringe. $NaBO_3{\cdot}4H_2O$ (462 mg, 3.00 mmol, 6.00 equiv.) was then added to the mixture in one portion. The reaction was stirred at 25 °C for 18 h. The reaction was quenched with sat. aq. $Na_2S_2O_3$ (1 mL) and further diluted with H_2O (1 mL). The organic layer was separated, and the aqueous layer was extracted with DCM or ethyl acetate (3 X 5 mL). The combined organic layers were dried over $MgSO_4$ gravity filtered, and concentrated *in vacuo*. The crude material was purified *via* silica gel chromatography or MPLC.

8. Characterization Data

In ^{13}C NMR spectra, carbons directly bonded to boron were not detected because of quadrupole relaxation.

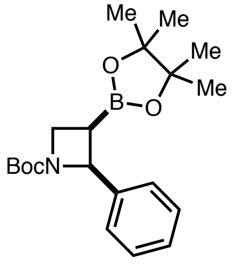

***tert*-butyl-2-phenyl-3-(4,4,5,5-tetramethyl-1,3,2-dioxaborolan-2-yl)azetidine-1-carboxylate (2):** The title compound was prepared according to General Procedure A with 50 mol% N-Me-

morpholine as additive. Crude yield was determined by ^{1}H NMR of the unpurified reaction mixture using mesitylene as an NMR standard. Purification by MPLC (gradient: 0-5% ethyl acetate in hexanes) yielded **2** as a colorless oil. Relative stereochemistry and regiochemistry for **2** were assigned by analogy to the crystal structure of **14**. Average yield over 2 runs: 93% NMR yield, >20:1 crude dr and rr. 76% isolated yield, 137 mg.

^{1}H NMR (500 MHz, $CDCl_3$) δ 7.34 – 7.25 (m, 4H), 7.18 (t, *J* = 7.1 Hz, 1H), 5.36 (br s, 1H), 4.11 (app. t, *J* = 7.3 Hz, 1H), 4.02 (dd, *J* = 9.7, 7.8 Hz, 1H), 2.54 (td, *J* = 9.9, 6.7 Hz, 1H), 1.57 – 1.09 (br m, 9H), 0.93 (s, 6H), 0.82 (s, 6H). **^{13}C NMR (126 MHz, $CDCl_3$, *mixture of rotamers*)** δ 156.1, 141.3, 128.2, 127.2, 126.2, 83.6, 79.3, 65.6, 64.7, 48.8, 47.7, 28.4, 24.9, 24.6 (signal of carbon directly bonded to boron was not detected because of quadrupolar relaxation). **IR (neat):** 2977 (w), 2931 (w), 2161 (w), 1700 (m), 1364 (s), 1128 (s), 857 (m), 692 (m) cm^{-1}. **HRMS (ESI+):** Calculated for: $C_{20}H_{30}O_4NBNa$ $[M+Na]^+$: 382.2160, found: 382.2166.

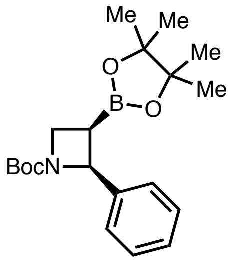

***tert*-butyl (2*S*,3*R*)-2-phenyl-3-(4,4,5,5-tetramethyl-1,3,2-dioxaborolan-2-yl)azetidine-1-carboxylate (2)** was prepared according to the following procedure: In an N_2-filled glovebox, to an oven-dried 13 x 100 mm screw-capped reaction vial equipped with a stir bar was added RuPhosPd G3 (1.67 mg, 0.00200 mmol, 1.00 mol%), Chiral NHC-CuCl **18** (5.41 mg, 0.010 mmol, 5.00 mol%), potassium *tert*-butoxide (33.7 mg, 0.300 mmol, 1.50 equiv.), and bis(pinacolato)diboron, (81.3 mg, 0.320 mmol, 1.60 equiv.). The reaction vial was sealed with a septum and removed from the glovebox. A solution of *tert*-butyl azete-1(2*H*)-carboxylate (37.0 µL, 0.220 mmol, 1.10 equiv.), bromobenzene (33.0 µL, 0.330 mmol, 1.65 equiv.), and N-Me-morpholine (11.5 µL, 0.110 mmol, 0.550 equiv.) in 10:1 Toluene/THF (2.10 mL) was prepared in a separate oven-dried 3-dram vial under N_2 and cooled to 0 °C in a dry ice/acetone bath. The reaction vial containing solids was also cooled to 0 °C, and 2.00 mL (0.200 mmol, 1.00 equiv. enecarbamate; 0.300 mmol, 1.50 equiv. ArBr, 0.100 mmol, 0.500 equiv. N-Me-morpholine) of the cold substrate solution was added to the reaction vial *via* syringe while stirring. The septum on the reaction vial was sealed with electrical tape, and the reaction was stirred at 0 °C in a fridge for 18 h. The reaction was quenched with H_2O (2 mL) and the mixture was extracted with EtOAc (3 X 2 mL). The combined organic layers were dried over $MgSO_4$, gravity filtered, and concentrated *in vacuo*. Mesitylene was added as an internal standard, and a small aliquot of the reaction mixture was analyzed *via* ^{1}H NMR. The crude material was purified *via* MPLC (gradient: 0-10% ethyl acetate in hexanes) to yield **2** as a colorless oil. Relative stereochemistry and regiochemistry for **2** were assigned by analogy to the crystal structure of **14**. 57% NMR yield, >20:1 crude dr, 13:1 rr. 52% isolated yield, 38 mg (isolated as a 14:1 mixture of regioisomers, characterization data for the major regioisomer is given. Because the sample contained 7% of the minor regioisomer, optical rotation data was not obtained). The enantiomeric purity was established *via* HPLC analysis using a chiral column. A racemic sample was prepared *via* General Procedure A. Note: absolute

configuration of this compound has not been determined, and the stereochemistry is drawn based on the model proposed in Scheme 2f.[1,23]

^{1}H NMR (400 MHz, CDCl$_3$) δ 7.36 – 7.24 (m, 4H), 7.19 (t, *J* = 7.2 Hz, 1H), 5.38 (br s, 1H), 4.12 (q, *J* = 7.2 Hz, 1H), 4.04 (dd, *J* = 9.7, 7.8 Hz, 1H), 2.56 (td, *J* = 10.0, 6.7 Hz, 1H), 1.50 – 1.14 (br m, 9H), 0.95 (s, 6H), 0.84 (s, 6H). **HPLC:** CHIRALPAK-IA, 22 °C. 0.5 mL/min, Hex/IPA 95:5, 220 nm, t_{minor} = 9.331 min, t_{major} = 11.017 min, 87:13 er. See Supporting Information part 14 for HPLC chromatographs.

^{1}H NMR data matched that for racemic **2**.

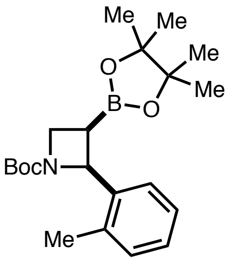

***tert*-butyl-3-(4,4,5,5-tetramethyl-1,3,2-dioxaborolan-2-yl)-2-(*o*-tolyl)azetidine-1-carboxylate (12):** The title compound was prepared according to General Procedure A with 50 mol% N-Me-morpholine as additive. Crude yield was determined by ^{1}H NMR of the unpurified reaction mixture using mesitylene as an NMR standard. Purification by MPLC (gradient: 0-5% ethyl acetate in hexanes) yielded **12** as a colorless oil. Relative stereochemistry and regiochemistry for **12** were assigned by analogy to the crystal structure of **14**. Average yield over 2 runs: 76% NMR yield, >20:1 crude dr and rr. 72% isolated yield, 134 mg.

^{1}H NMR (500 MHz, CDCl$_3$) δ 7.43 (d, *J* = 7.7 Hz, 1H), 7.14 (t, *J* = 7.5 Hz, 1H), 7.07 (t, *J* = 7.3 Hz, 1H), 7.03 (d, *J* = 8.1 Hz, 1H), 5.52 (d, *J* = 10.2 Hz, 1H), 4.10 – 3.98 (m, 2H), 2.52 (td, *J* = 9.7, 6.4 Hz, 1H), 2.23 (s, 3H), 1.64 – 1.12 (br m, 9H), 0.86 (s, 6H), 0.81 (s, 6H). **^{13}C NMR (126 MHz, CDCl$_3$)** δ 156.7, 138.8, 134.7, 129.8, 126.9, 125.7, 125.4, 83.5, 79.4, 62.8, 47.5, 28.5, 24.8, 24.5, 18.9. (signal of carbon directly bonded to boron was not detected because of quadrupolar relaxation). **IR (neat):** 2977 (w), 2931 (w), 2160 (w), 2037 (w), 1700 (m), 1361 (s), 1130 (s), 859 (m) cm^{-1}. **HRMS (ESI+):** Calculated for: $C_{21}H_{32}O_4NBNa$ [M+Na]$^+$: 396.2317, found: 396.2321.

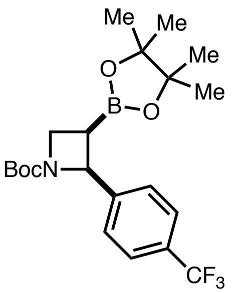

***tert*-butyl-3-(4,4,5,5-tetramethyl-1,3,2-dioxaborolan-2-yl)-2-(4-(trifluoromethyl)phenyl)azetidine-1-carboxylate (3):** The title compound was prepared according to General Procedure A with 50 mol% N-Me-morpholine as additive. Crude yield was determined by ^{1}H NMR of the unpurified reaction mixture using mesitylene as an NMR standard. Purification by MPLC (gradient: 0-10% ethyl acetate in hexanes) and a second MPLC (gradient:

0-10% ethyl acetate in hexanes) yielded **3** as a white solid. Relative stereochemistry and regiochemistry for **3** were assigned by analogy to the crystal structure of **14**. Average yield over 2 runs: 91% NMR yield, >20:1 crude dr and rr. 70% isolated yield, 149 mg.

^{1}H NMR (500 MHz, CDCl$_3$) δ 7.54 (d, J = 8.0 Hz, 2H), 7.43 (d, J = 8.0 Hz, 2H), 5.41 (d, J = 10.3 Hz, 1H), 4.11 – 3.98 (m, 2H), 2.54 (td, J = 9.8, 6.5 Hz, 1H), 1.59 – 1.11 (m, 9H), 0.90 (s, 6H), 0.80 (s, 6H). **^{13}C NMR (126 MHz, CDCl$_3$, *mixture of rotamers*)** δ 156.1, 145.3, 129.6 (q, J = 32.2 Hz), 126.5, 125.2, 124.3 (q, J = 271.8 Hz), 83.7, 79.7, 64.9, 64.1, 48.8, 47.7, 28.3, 24.7, 24.5 (signal of carbon directly bonded to boron was not detected because of quadrupolar relaxation). **^{19}F NMR (471 MHz, CDCl$_3$)** δ -62.55. **IR (neat):** 2978 (w), 2930 (w), 1712 (m), 1364 (s), 1322 (s), 1128 (s), 1109 (s), 855 (m) cm^{-1}. **HRMS (ESI+):** Calculated for: $C_{21}H_{29}O_4NBF_3Na$ [M+Na]$^+$: 450.2034, found: 450.2040.

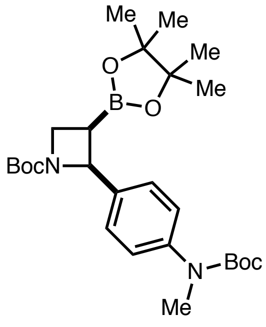

***tert*-butyl-2-(4-((*tert*-butoxycarbonyl)(methyl)amino)phenyl)-3-(4,4,5,5-tetramethyl-1,3,2-dioxaborolan-2-yl)azetidine-1-carboxylate (13):** The title compound was prepared according to General Procedure A with 50 mol% N-Me-morpholine as additive. Crude yield was determined by ^{1}H NMR of the unpurified reaction mixture using mesitylene as an NMR standard. Purification by MPLC (gradient: 0-20% ethyl acetate in hexanes) yielded **13** as a light yellow oil. Relative stereochemistry and regiochemistry for **13** were assigned by analogy to the crystal structure of **14**. Average yield over 2 runs: 80% NMR yield, >20:1 crude dr and rr. 77% isolated yield, 188 mg.

^{1}H NMR (500 MHz, CDCl$_3$) δ 7.20 (d, J = 8.5 Hz, 2H), 7.07 (d, J = 8.2 Hz, 2H), 5.27 (br s, 1H), 4.02 (app. t, J = 7.3 Hz, 1H), 3.94 (dd, J = 9.7, 7.8 Hz, 1H), 3.11 (s, 3H), 2.46 (td, J = 9.9, 6.6 Hz, 1H), 1.50 – 1.05 (br m, 18H), 0.89 (s, 6H), 0.78 (s, 6H). **^{13}C NMR (126 MHz, CDCl$_3$, *mixture of rotamers*)** δ 155.8, 154.5, 142.9, 138.4, 126.3, 125.0, 83.5, 80.0, 79.1, 65.1, 64.3, 48.6, 47.5, 37.4, 28.3, 28.2, 24.8, 24.4 (signal of carbon directly bonded to boron was not detected because of quadrupolar relaxation). **IR (neat):** 2976 (w), 2931 (w), 1696 (s), 1362 (s), 1131 (s), 858 (m), 731 (m) cm^{-1}. **HRMS (ESI+):** Calculated for: $C_{26}H_{41}O_6N_2BNa$ [M+Na]$^+$: 511.2950, found: 511.2954.

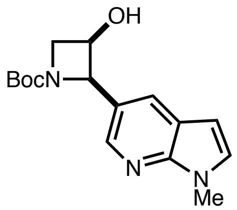

***tert*-butyl-3-hydroxy-2-(1-methyl-1*H*-pyrrolo[2,3-*b*]pyridin-5-yl)azetidine-1-carboxylate (4):** The title compound was prepared according to General Procedure A without 50 mol% N-Me-morpholine as additive. Crude yield of the Bpin product was determined by ^{1}H NMR of the

unpurified reaction mixture using mesitylene as an NMR standard. The crude mixture was then oxidized the alcohol according to General Procedure C. Purification by silica gel chromatography (40% acetone in hexanes) yielded **4** as an off-white foam. Relative stereochemistry and regiochemistry for **4** were assigned by analogy to the crystal structure of **14**. Average yield over 2 runs: 69% NMR yield (Bpin), >20:1 crude dr and rr. 68% isolated yield (alcohol), 103 mg.

^{1}H NMR (500 MHz, $CDCl_3$) δ 8.13 (s, 1H), 7.91 (s, 1H), 7.12 (s, 1H), 6.41 (br d, *J* = 1.4 Hz, 1H), 5.45 (d, *J* = 6.8 Hz, 1H), 4.75 (br s, 1H), 4.31 (dd, *J* = 9.8, 7.1 Hz, 1H), 3.87 (dd, *J* = 10.1, 4.0 Hz, 1H), 3.80 (s, 3H), 2.76 (br s, 1H), 1.32 (s, 9H). **^{13}C NMR (126 MHz, $CDCl_3$)** δ 156.3, 147.5, 142.3, 129.9, 128.4, 122.7, 120.5, 99.4, 80.0, 69.0, 64.2, 57.3, 31.5, 28.4. **IR (neat):** 3190 (br,w), 2970 (w), 1738 (m), 1693 (s), 1391 (s), 1364 (s), 1231 (m), 1137 (s), 727 (s) cm^{-1}. **HRMS (ESI+):** Calculated for: $C_{16}H_{22}O_3N_3$ $[M+H]^+$: 304.1656, found: 304.1657.

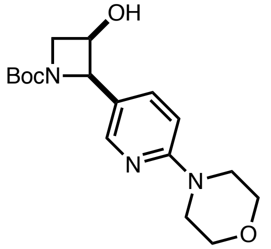

***tert*-butyl-3-hydroxy-2-(6-morpholinopyridin-3-yl)azetidine-1-carboxylate (48):** The title compound was prepared according to General Procedure A without 50 mol% N-Me-morpholine as additive. Crude yield of the Bpin product was determined by ^{1}H NMR of the unpurified reaction mixture using mesitylene as an NMR standard. The crude mixture was then oxidized the alcohol according to General Procedure C. Purification by silica gel chromatography (40% acetone in hexanes) yielded **48** as a white foam. Relative stereochemistry and regiochemistry for **48** were assigned by analogy to the crystal structure of **14**. Average yield over 2 runs: 82% NMR yield (Bpin), >20:1 crude dr and rr. 73% isolated yield (alcohol), 122 mg.

^{1}H NMR (500 MHz, $CDCl_3$) δ 7.96 (s, 1H), 7.51 (d, *J* = 8.4 Hz, 1H), 6.62 (d, *J* = 8.8 Hz, 1H), 5.14 (d, *J* = 6.8 Hz, 1H), 4.63 (br app. dd, *J* = 9.8, 5.4 Hz, 1H), 4.18 (app. t, *J* = 8.4 Hz, 1H), 3.75 (br s, 5H), 3.41 (br s, 4H), 3.28 (br s, 1H), 1.57 – 1.13 (br m, 9H). **^{13}C NMR (126 MHz, $CDCl_3$)** δ 159.4, 156.1, 147.2, 137.6, 121.1, 106.7, 79.9, 68.1, 66.7, 64.0, 57.3, 45.8, 28.4. **IR (neat):** 3409 (w), 2997 (w), 2848 (w), 2160 (w), 2030 (w), 1670 (s), 1606 (m), 1493 (m), 1398 (s), 1250 (m), 1143 (s), 1117 (s), 944 (m), 585 (m) cm^{-1}. **HRMS (ESI+):** Calculated for: $C_{17}H_{26}O_4N_3$ $[M+H]^+$: 336.1918, found: 336.1921.

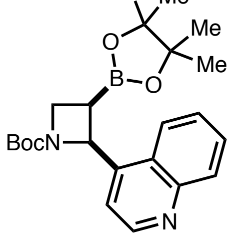

***tert*-butyl-2-(quinolin-4-yl)-3-(4,4,5,5-tetramethyl-1,3,2-dioxaborolan-2-yl)azetidine-1-carboxylate (15):** The title compound was prepared according to General Procedure A without 50 mol% N-Me-morpholine as additive. Crude yield was determined by ^{1}H NMR of the unpurified

reaction mixture using mesitylene as an NMR standard. Purification by MPLC (gradient: 0-20% ethyl acetate in hexanes) and a second MPLC (gradient: 0-20% ethyl acetate in hexanes) yielded **15** as an off-white foam. Relative stereochemistry and regiochemistry for **15** were assigned by analogy to the crystal structure of **14**. Average yield over 2 runs: 54% NMR yield, >20:1 crude dr and rr. 51% isolated yield, 105 mg.

^{1}H NMR (500 MHz, CDCl$_3$) δ 8.86 (s, 1H), 8.05 (d, J = 8.4 Hz, 1H), 7.71 (d, J = 8.4 Hz, 1H), 7.62 (t, J = 7.6 Hz, 1H), 7.55 – 7.45 (m, 2H), 6.01 (d, J = 10.5 Hz, 1H), 4.14 (app. t, J = 8.4 Hz, 1H), 3.99 (app. t, J = 5.9 Hz, 1H), 2.74 (td, J = 10.1, 5.9 Hz, 1H), 1.65 – 1.12 (br m, 9H), 0.57 (s, 6H), 0.45 (s, 6H). **^{13}C NMR (126 MHz, CDCl$_3$)** δ 156.7, 150.3, 148.1, 146.1, 130.0, 129.0, 126.3, 125.3, 123.5, 117.8, 83.4, 80.0, 61.9, 48.9, 28.4, 24.2, 24.1 (signal of carbon directly bonded to boron was not detected because of quadrupolar relaxation). **IR (neat):** 2973 (w), 2892 (w), 1701 (m), 1360 (s), 1129 (s), 856 (m), 768 (m) cm^{-1}. **HRMS (ESI+):** Calculated for: $C_{23}H_{32}O_4N_2B$ $[M+H]^+$: 411.2450, found: 411.2459.

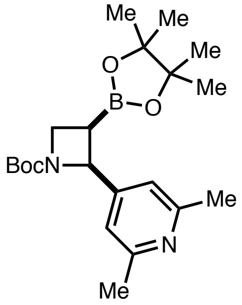

***tert*-butyl-2-(2,6-dimethylpyridin-4-yl)-3-(4,4,5,5-tetramethyl-1,3,2-dioxaborolan-2-yl)azetidine-1-carboxylate (16):** The title compound was prepared according to General Procedure A without 50 mol% N-Me-morpholine as additive. Crude yield was determined by ^{1}H NMR of the unpurified reaction mixture using mesitylene as an NMR standard. Purification by MPLC (gradient: 0-40% ethyl acetate in hexanes) and a second MPLC (gradient: 0-40% ethyl acetate in hexanes) yielded **16** as an off-white solid. Relative stereochemistry and regiochemistry for **16** were assigned by analogy to the crystal structure of **14**. Average yield over 2 runs: 71% NMR yield, >20:1 crude dr and rr. 56% isolated yield, 109 mg.

^{1}H NMR (500 MHz, CDCl$_3$) δ 6.84 (s, 2H), 5.19 (d, J = 10.5 Hz, 1H), 4.03 – 3.91 (m, 2H), 2.45 (td, J = 10.0, 6.6 Hz, 1H), 2.40 (s, 6H), 1.53 – 1.05 (br m, 9H), 0.86 (s, 6H), 0.79 (s, 6H). **^{13}C NMR (126 MHz, CDCl$_3$, *mixture of rotamers*)** δ 157.5, 155.8, 150.3, 117.7, 83.6, 79.6, 64.2, 63.4, 48.8, 47.7, 28.3, 24.7, 24.5, 24.4 (signal of carbon directly bonded to boron was not detected because of quadrupolar relaxation). **IR (neat):** 2977 (w), 2888 (w), 1697 (m), 1363 (s), 1331 (m), 1130 (s), 860 (m) cm^{-1}. **HRMS (ESI+):** Calculated for: $C_{21}H_{34}O_4N_2B$ $[M+H]^+$: 389.2606, found: 389.2615.

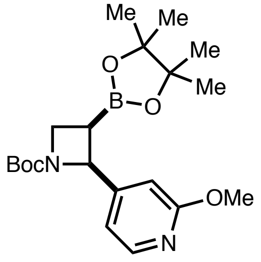

***tert*-butyl-2-(2-methoxypyridin-4-yl)-3-(4,4,5,5-tetramethyl-1,3,2-dioxaborolan-2-yl)azetidine-1-carboxylate (17):** The title compound was prepared according to General Procedure A without 50 mol% N-Me-morpholine as additive. Crude yield was determined by ^{1}H NMR of the unpurified reaction mixture using mesitylene as an NMR standard. Purification by MPLC (gradient: 0-20% ethyl acetate in hexanes) and a second MPLC (gradient: 0-20% ethyl acetate in hexanes) yielded **17** as a white foam. Relative stereochemistry and regiochemistry for **17** were assigned by analogy to the crystal structure of **14**. Average yield over 2 runs: 73% NMR yield, >20:1 crude dr and rr. 71% isolated yield, 139 mg.

^{1}H NMR (500 MHz, CDCl$_3$) δ 8.04 (d, *J* = 5.3 Hz, 1H), 6.79 (d, *J* = 5.4 Hz, 1H), 6.68 (s, 1H), 5.24 (d, *J* = 10.5 Hz, 1H), 4.06 – 3.95 (m, 2H), 3.86 (s, 3H), 2.50 (td, *J* = 10.0, 6.8 Hz, 1H), 1.54 – 1.13 (br m, 9H), 0.93 (s, 6H), 0.86 (s, 6H). **^{13}C NMR (126 MHz, CDCl$_3$, *mixture of rotamers*)** δ 164.6, 156.0, 152.9, 146.7, 114.8, 108.1, 83.8, 79.8, 64.2, 63.3, 53.4, 48.8, 47.7, 28.4, 24.7, 24.6 (signal of carbon directly bonded to boron was not detected because of quadrupolar relaxation). **IR (neat):** 2977 (w), 2692 (w), 1702 (m), 1612 (m), 1363 (s), 1128 (s), 855 (m), 732 (m) cm^{-1}. **HRMS (ESI+):** Calculated for: $C_{20}H_{32}O_5N_2B$ [M+H]$^+$: 391.2399, found: 391.2405.

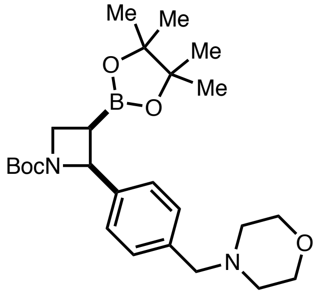

***tert*-butyl-2-(4-(morpholinomethyl)phenyl)-3-(4,4,5,5-tetramethyl-1,3,2-dioxaborolan-2-yl)azetidine-1-carboxylate (5)** The title compound was prepared according to General Procedure A without 50 mol% N-Me-morpholine as additive. Crude yield was determined by ^{1}H NMR of the unpurified reaction mixture using mesitylene as an NMR standard. Purification by MPLC (gradient: 0-30% ethyl acetate in hexanes) yielded **5** as an off-white solid. Relative stereochemistry and regiochemistry for **5** were assigned by analogy to the crystal structure of **14**. Average yield over 2 runs: 82% NMR yield, >20:1 crude dr and rr. 81% isolated yield, 186 mg.

^{1}H NMR (500 MHz, CDCl$_3$) δ 7.24 (d, *J* = 8.2 Hz, 2H), 7.21 (d, *J* = 8.1 Hz, 2H), 5.32 (s, 1H), 4.07 (app. t, *J* = 7.3 Hz, 1H), 3.98 (dd, *J* = 9.7, 7.8 Hz, 1H), 3.63 (app. t, *J* = 4.6 Hz, 4H), 3.41 (s, 2H), 2.50 (td, *J* = 10.0, 6.6 Hz, 1H), 2.37 (br app. t, *J* = 4.6 Hz, 4H), 1.57 – 1.04 (br m, 9H), 0.91 (s, 6H), 0.80 (s, 6H). **^{13}C NMR (126 MHz, CDCl$_3$)** δ 156.0, 140.1, 136.6, 129.0, 126.2, 83.5, 79.2, 67.0, 64.9, 63.1, 53.5, 47.8, 28.3, 24.8, 24.5. **IR (neat):** 2976 (w), 2803 (w), 2364 (m), 2168 (m),

2030 (m), 1697 (s), 1365 (s), 1331 (s), 1110 (s), 865 (m) cm^{-1}. **HRMS (ESI+):** Calculated for: $C_{25}H_{40}O_5N_2B$ $[M+H]^+$: 459.3025, found: 459.3033.

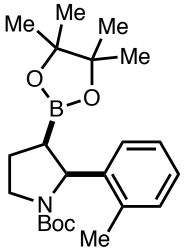

***tert*-butyl-3-(4,4,5,5-tetramethyl-1,3,2-dioxaborolan-2-yl)-2-(*o*-tolyl)pyrrolidine-1-carboxylate (21):** The title compound was prepared according to General Procedure B. Crude yield was determined by ^{1}H NMR of the unpurified reaction mixture using 1,3,5-trimethoxybenzene as an NMR standard. Purification by silica gel chromatography (10% ethyl acetate in hexanes) yielded **21** as a white solid. Relative stereochemistry and regiochemistry of the major regioisomer for **21** were assigned by analogy to the crystal structure of **20**. Minor regioisomer was assigned by analogy to **37B**; note: rotamers complicated NMR analysis, thus crude rr was approximately assigned as >10:1. Average yield over 2 runs: 62% NMR yield, >20:1 crude dr, >10:1 crude rr. 42% isolated yield, 81 mg (isolated as a single regioisomer).

^{1}H NMR (400 MHz, CDCl$_3$, *3.5:1 mixture of rotamers*) δ 7.10 – 6.98 (m, 4H), 5.29 (d, *J* = 8.4 Hz, 0.2H, *minor rotamer*), 5.14 (d, *J* = 9.0 Hz, 0.8H, *major rotamer*), 3.86 (t, *J* = 9.1 Hz, 0.8H, *major rotamer*), 3.74 (t, *J* = 9.5 Hz, 0.2H, *minor rotamer*), 3.41 (td, *J* = 10.7, 6.7 Hz, 1H), 2.47 (s, 0.6H, *minor rotamer*), 2.40 (s, 2.4H, *major rotamer*), 2.39 – 2.23 (m, 1H), 2.11 – 1.93 (m, 2H), 1.39 (s, 2H, *minor rotamer*), 1.11 (s, 7H, *major rotamer*), 0.92 (s, 6H), 0.81 (d, *J* = 3.8 Hz, 6H). **^{13}C NMR (101 MHz, CDCl$_3$, *mixture of rotamers*)** δ 154.3, 143.2, 135.4, 129.7, 126.5, 126.0, 125.9, 83.3, 79.0, 58.2, 47.4, 28.7, 28.2, 27.2, 25.2, 24.9, 24.4, 20.0 (signal of carbon directly bonded to boron was not detected because of quadrupolar relaxation). **IR (neat):** 3019 (w), 2974 (w), 2867 (w), 1686 (s), 1361 (s), 1327 (s), 1166 (m), 1143 (s), 1121 (s), 978 (m), 857 (m), 748 (m), 578 (m) cm^{-1}. **HRMS (ESI+):** Calculated for: $C_{22}H_{34}O_4NBNa$ $[M+Na]^+$: 410.2473, found: 410.2477.

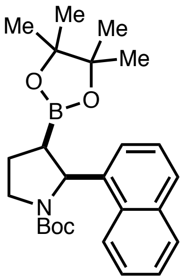

***tert*-butyl-2-(naphthalen-1-yl)-3-(4,4,5,5-tetramethyl-1,3,2-dioxaborolan-2-yl)pyrrolidine-1-carboxylate (22):** The title compound was prepared according to General Procedure B. Crude yield was determined by ^{1}H NMR of the unpurified reaction mixture using 1,3,5-trimethoxybenzene as an NMR standard. Purification by silica gel chromatography (10% ethyl acetate in hexanes) yielded **22** as a white solid. Relative stereochemistry and regiochemistry of the major regioisomer for **22** were assigned by analogy to the crystal structure of **20**. Minor regioisomer was assigned by analogy to **37B**; note: rotamers complicated NMR analysis, thus

crude rr was approximately assigned as >10:1. Average yield over 2 runs: 86% NMR yield, >20:1 crude dr, >10:1 crude rr. 69% isolated yield, 146 mg (isolated as a single regioisomer).

^{1}H NMR (500 MHz, CDCl$_3$, *2.4:1 mixture of rotamers*) δ 8.24 (d, *J* = 8.6 Hz, 0.3H, *minor rotamer*), 8.19 (d, *J* = 8.5 Hz, 0.7H, *major rotamer*), 7.82 – 7.73 (m, 1H), 7.71 – 7.63 (m, 1H), 7.50 – 7.35 (m, 3H), 7.32 – 7.23 (m, 1H), 5.99 (d, *J* = 8.7 Hz, 0.3H, *minor rotamer*), 5.86 (d, *J* = 8.8 Hz, 0.7H, *major rotamer*), 3.95 (t, *J* = 10.1 Hz, 0.7H, *major rotamer*), 3.83 (t, *J* = 9.5 Hz, 0.3H, *minor rotamer*), 3.51 (td, *J* = 10.5, 6.9 Hz, 1H), 2.38 – 2.09 (m, 2H), 2.09 – 1.94 (m, 1H), 1.41 (s, 3H, *minor rotamer*), 0.91 (s, 6H, *major rotamer*), 0.62 (s, 2H, *minor rotamer*), 0.57 (s, 4H, *major rotamer*), 0.49 (s, 4H, *major rotamer*), 0.44 (s, 2H, *minor rotamer*). **^{13}C NMR (126 MHz, CDCl$_3$, *mixture of rotamers*)** δ 154.3, 154.2, 140.7, 139.3, 134.1, 133.6, 131.1, 131.0, 128.4, 128.3, 127.2, 127.0, 125.5, 125.4, 125.3, 124.4, 124.1, 123.1, 122.6, 83.0, 82.9, 79.2, 78.9, 58.5, 58.1, 48.0, 47.4, 28.7, 28.0, 26.5, 25.5, 24.7, 24.6, 23.9 (signal of carbon directly bonded to boron was not detected because of quadrupolar relaxation). **IR (neat):** 3048 (w), 2974 (w), 2928 (w), 2883 (w), 1688 (s), 1360 (s), 1327 (m), 1112 (m), 799 (m), 780 (m) cm^{-1}. **HRMS (ESI+):** Calculated for: $C_{25}H_{34}O_4NBNa$ [M+Na]$^+$: 446.2473, found: 446.2472.

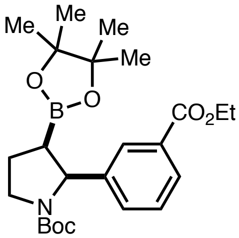

***tert*-butyl-2-(3-(ethoxycarbonyl)phenyl)-3-(4,4,5,5-tetramethyl-1,3,2-dioxaborolan-2-yl)pyrrolidine-1-carboxylate (23):** The title compound was prepared according to General Procedure B. Crude yield was determined by ^{1}H NMR of the unpurified reaction mixture using mesitylene as an NMR standard. Purification by silica gel chromatography (15% ethyl acetate in hexanes) yielded **23** as a colorless oil. Relative stereochemistry and regiochemistry of the major regioisomer for **23** were assigned by analogy to the crystal structure of **20**. Minor regioisomer was assigned by analogy to **37B**; note: rotamers complicated NMR analysis, thus crude rr was approximately assigned as >10:1. Average yield over 2 runs: 56% NMR yield, >20:1 crude dr, >10:1 crude rr. 51% isolated yield, 114 mg (isolated as a ~9:1 regioisomeric mixture, characterization data for the major regioisomer is given).

^{1}H NMR (400 MHz, CDCl$_3$, *2.0:1 mixture of rotamers*) δ 7.95 – 7.85 (m, 2H), 7.46 – 7.27 (m, 2H), 5.11 (d, *J* = 8.1 Hz, 0.3H, *minor rotamer*), 4.99 (d, *J* = 8.4 Hz, 0.7H, *major rotamer*), 4.37 (q, *J* = 7.1 Hz, 2H), 3.84 (t, *J* = 10.1 Hz, 0.7H, *major rotamer*), 3.76 (t, *J* = 9.0 Hz, 0.3H, *minor rotamer*), 3.53 – 3.35 (m, 1H), 2.28 – 2.13 (m, 1H), 2.11 – 1.93 (m, 2H), 1.42 (s, 2H, *minor rotamer*), 1.39 (t, *J* = 7.1 Hz, 3H), 1.16 (s, 7H, *major rotamer*), 0.98 (s, 2H, *minor rotamer*), 0.96 (s, 4H, *major rotamer*), 0.87 (s, 6H). **^{13}C NMR (101 MHz, CDCl$_3$, *mixture of rotamers*)** δ 166.7, 154.2, 144.7, 144.0, 131.3, 130.3, 130.0, 128.4, 128.2, 128.1, 127.9, 83.4, 79.3, 79.2, 62.8, 62.6, 60.9, 60.8, 47.9, 47.3, 28.6, 28.2, 24.8, 24.7, 24.5, 14.4 (signal of carbon directly bonded to boron was not detected because of quadrupolar relaxation). **IR (neat):** 3067 (w), 2977 (w), 2932 (w), 2877 (w), 1718 (m), 1694 (s), 1365 (s), 1287 (m), 1166 (s), 1141 (s), 1115 (s), 856 (m), 756 (m), 730 (m), 698 (m) cm^{-1}. **HRMS (ESI+):** Calculated for: $C_{24}H_{36}O_6NBNa$ [M+Na]$^+$: 468.2528, found: 468.2530.

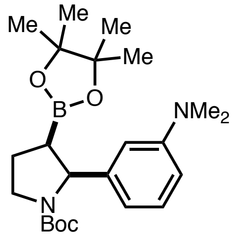

***tert*-butyl-2-(3-(dimethylamino)phenyl)-3-(4,4,5,5-tetramethyl-1,3,2-dioxaborolan-2-yl)pyrrolidine-1-carboxylate (24):** The title compound was prepared according to General Procedure B. Crude yield was determined by ^{1}H NMR of the unpurified reaction mixture using 1,3,5-trimethoxybenzene as an NMR standard. Purification by silica gel chromatography (20% ethyl acetate in hexanes) yielded **24** as a colorless oil. Relative stereochemistry and regiochemistry of the major regioisomer for **24** were assigned by analogy to the crystal structure of **20**. Minor regioisomer was assigned by analogy to **37B**; note: rotamers complicated NMR analysis, thus crude rr was approximately assigned as >10:1. Average yield over 2 runs: 90% NMR yield, >20:1 crude dr, >10:1 crude rr. 56% isolated yield, 117 mg (isolated as a single regioisomer).

^{1}H NMR (500 MHz, $CDCl_3$, *2.1:1 mixture of rotamers*) δ 7.06 (t, *J* = 8.0 Hz, 1H), 6.62 – 6.47 (m, 3H), 4.99 (d, *J* = 8.2 Hz, 0.3H, *minor rotamer*), 4.86 (d, *J* = 8.4 Hz, 0.7H, *major rotamer*), 3.78 (t, *J* = 9.7 Hz, 0.7H, *major rotamer*), 3.68 (t, *J* = 9.6 Hz, 0.3H, *minor rotamer*), 3.46 – 3.30 (m, 1H), 2.88 (s, 6H), 2.32 – 2.15 (m, 1H), 2.05 – 1.88 (m, 2H), 1.40 (s, 3H, *minor rotamer*), 1.16 (s, 6H, *major rotamer*), 1.00 (s, 2H, *minor rotamer*), 0.98 (s, 4H, *major rotamer*), 0.87 (s, 6H). **^{13}C NMR (126 MHz, $CDCl_3$, *mixture of rotamers*)** δ 154.6, 154.1, 150.6, 144.9, 144.2, 128.7, 128.5, 115.8, 115.1, 112.2, 111.7, 83.3, 83.2, 78.9, 63.6, 63.3, 47.9, 47.3, 41.0, 40.8, 28.7, 28.3, 25.8, 25.1, 25.0, 24.6 (signal of carbon directly bonded to boron was not detected because of quadrupolar relaxation). **IR (neat):** 2975 (w), 2930 (w), 2876 (w), 2799 (w), 1691 (s), 1603 (m), 1365 (s), 1324 (s) 1166 (s), 1141 (s), 1118 (s), 851 (m), 775 (m), 732 (m), 698 (m) cm^{-1}. **HRMS (ESI+):** Calculated for: $C_{23}H_{38}O_4N_2B$ $[M+H]^+$: 417.2919, found: 417.2920.

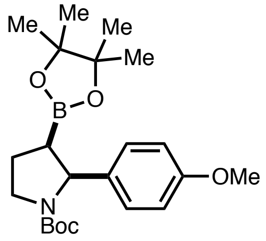

***tert*-butyl-2-(4-methoxyphenyl)-3-(4,4,5,5-tetramethyl-1,3,2-dioxaborolan-2-yl)pyrrolidine-1-carboxylate (25):** The title compound was prepared according to General Procedure B. Crude yield was determined by ^{1}H NMR of the unpurified reaction mixture using 1,3,5-trimethoxybenzene as an NMR standard. Purification by silica gel chromatography (20% ethyl acetate in hexanes) yielded **25** as a white solid. Relative stereochemistry and regiochemistry of the major regioisomer for **25** were assigned by analogy to the crystal structure of **20**. Minor regioisomer was assigned by analogy to **37B**; note: rotamers complicated NMR analysis, thus crude rr was approximately assigned as >10:1. Average yield over 2 runs: 82% NMR yield, >20:1 crude dr, >10:1 crude rr. 62% isolated yield, 125 mg (isolated as a ~13:1 regioisomeric mixture, characterization data for the major regioisomer is given).

^{1}H NMR (400 MHz, CDCl$_3$, *2.2:1 mixture of rotamers*) δ 7.14 – 7.04 (m, 2H), 6.74 (d, *J* = 8.7 Hz, 2H), 4.98 (d, *J* = 8.2 Hz, 0.3H, *minor rotamer*), 4.86 (d, *J* = 8.1 Hz, 0.7H, *major rotamer*), 3.83 – 3.61 (m, 4H), 3.45 – 3.26 (m, 1H), 2.26 – 2.10 (m, 1H), 2.03 – 1.85 (m, 2H), 1.38 (s, 3H, *minor rotamer*), 1.14 (s, 6H, *major rotamer*), 0.97 (s, 6H), 0.87 (d, *J* = 3.3 Hz, 6H). **^{13}C NMR (101 MHz, CDCl$_3$, *mixture of rotamers*)** δ 158.6, 154.4, 136.7, 136.0, 128.1, 127.9, 113.5, 113.1, 83.3, 78.9, 62.5, 62.2, 55.4, 53.7, 47.2, 28.6, 28.3, 24.9, 24.8, 24.6 (carbon directly bonded to boron was not detected because of quadrupole relaxation). **IR (neat):** 3060 (w), 2978 (w), 2930 (w), 2879 (w), 1694 (s), 1513 (m), 1365 (s), 1313 (s), 1246 (s), 1167 (s), 1142 (s), 1112 (s), 828 (m), 536 (m) cm^{-1}. **HRMS (ESI+):** Calculated for: $C_{22}H_{34}O_5NBNa$ [M+Na]$^+$: 426.2422, found: 426.2427.

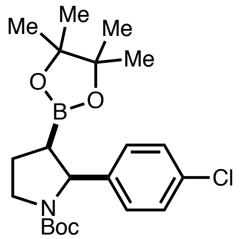

***tert*-butyl-2-(4-chlorophenyl)-3-(4,4,5,5-tetramethyl-1,3,2-dioxaborolan-2-yl)pyrrolidine-1-carboxylate (26):** The title compound was prepared according to General Procedure B. Crude yield was determined by ^{1}H NMR of the unpurified reaction mixture using 1,3,5-trimethoxybenzene as an NMR standard. Purification by silica gel chromatography (10% ethyl acetate in hexanes) yielded **26** as a white foam. Relative stereochemistry and regiochemistry of the major regioisomer for **26** were assigned by analogy to the crystal structure of **20**. Minor regioisomer was assigned by analogy to **37B**; note: rotamers complicated NMR analysis, thus crude rr was approximately assigned as >10:1. Average yield over 2 runs: 71% NMR yield, >20:1 crude dr, >10:1 crude rr. 50% isolated yield, 102 mg (isolated as a ~9:1 regioisomeric mixture, characterization data for the major regioisomer is given).

^{1}H NMR (400 MHz, CDCl$_3$, *1.9:1 mixture of rotamers*) δ 7.21 (d, *J* = 8.3 Hz, 2H), 7.18 – 7.09 (m, 2H), 4.99 (d, *J* = 8.2 Hz, 0.3H, *minor rotamer*), 4.88 (d, *J* = 7.9 Hz, 0.7H, *major rotamer*), 3.78 (t, *J* = 9.5 Hz, 0.6H, *major rotamer*), 3.69 (t, *J* = 10.0 Hz, 0.3H, *minor rotamer*), 3.47 – 3.31 (m, 1H), 2.27 – 2.07 (m, 1H), 2.06 – 1.88 (m, 2H), 1.40 (s, 3H, *minor rotamer*), 1.16 (s, 6H, *major rotamer*), 0.99 (s, 6H), 0.90 (s, 6H). **^{13}C NMR (101 MHz, CDCl$_3$, *mixture of rotamers*)** δ 154.3, 143.0, 132.4, 128.5, 128.4, 128.2, 127.9, 83.5, 79.3, 62.5, 62.3, 47.9, 47.3, 28.6, 28.3, 27.2, 25.8, 24.9, 24.6 (carbon directly bonded to boron was not detected because of quadrupole relaxation). **IR (neat):** 3055 (w), 2974 (w), 2931 (w), 2882 (w), 1694 (s), 1364 (s), 1330 (s), 1169 (s), 1139 (s), 1111 (s), 901 (m), 843 (m), 820 (m) cm^{-1}. **HRMS (ESI+):** Calculated for: $C_{21}H_{31}O_4NBClNa$ [M+Na]$^+$: 430.1927, found: 430.1929.

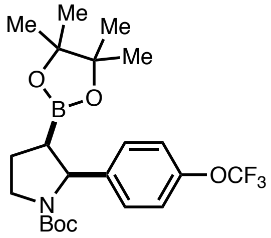

***tert*-butyl-3-(4,4,5,5-tetramethyl-1,3,2-dioxaborolan-2-yl)-2-(4-(trifluoromethoxy)phenyl)pyrrolidine-1-carboxylate (27):** The title compound was prepared according to General Procedure B. Crude yield was determined by ^{1}H NMR of the unpurified reaction mixture using 1,3,5-trimethoxybenzene as an NMR standard. Purification by silica gel chromatography (10% ethyl acetate in hexanes) yielded **27** as a white solid. Relative stereochemistry and regiochemistry of the major regioisomer for **27** were assigned by analogy to the crystal structure of **20**. Minor regioisomer was assigned by analogy to **37B**; note: rotamers complicated NMR analysis, thus crude rr was approximately assigned as >10:1. Average yield over 2 runs: 55% NMR yield, >20:1 crude dr, >10:1 crude rr. 45% isolated yield, 103 mg (isolated as a ~10:1 regioisomeric mixture, characterization data for the major regioisomer is given).

^{1}H NMR (500 MHz, CDCl$_3$, *1.9:1 mixture of rotamers*) δ 7.25 – 7.19 (m, 2H), 7.15 – 7.06 (m, 2H), 5.03 (d, *J* = 8.4 Hz, 0.3H, *minor rotamer*), 4.90 (d, *J* = 8.5 Hz, 0.7H, *major rotamer*), 3.81 (t, *J* = 9.5 Hz, 0.7H, *major rotamer*), 3.70 (t, *J* = 8.8 Hz, 0.3H, *minor rotamer*), 3.45 – 3.33 (m, 1H), 2.25 – 2.10 (m, 1H), 2.08 – 1.91 (m, 2H), 1.40 (s, 3H, *minor rotamer*), 1.14 (s, 6H, *major rotamer*), 0.97 (br d, *J* = 5.7 Hz, 6H, *rotamers*), 0.87 (br d, *J* = 4.6 Hz, 6H, *rotamers*). **^{13}C NMR (126 MHz, CDCl$_3$, *mixture of rotamers*)** δ 154.2, 148.1, 143.5, 142.7, 129.4, 128.5, 128.3, 120.9, 120.7 (q, *J* = 254.8 Hz), 120.6, 83.5, 83.4, 79.5, 79.4, 62.5, 62.3, 47.9, 47.4, 28.6, 28.2, 25.8, 24.9, 24.8, 24.5, 24.4 (signal of carbon directly bonded to boron was not detected because of quadrupolar relaxation). **^{19}F NMR (471 MHz, CDCl$_3$, *mixture of rotamers*)** δ -57.96, -58.13 **IR (neat):** 3073 (w), 2982 (w), 1689 (m), 1363 (s), 1255 (s), 1219 (s), 1155 (s), 1105 (s), 850 (m) cm^{-1}. **HRMS (ESI+):** Calculated for: $C_{22}H_{31}O_5NBF_3Na$ $[M+Na]^+$: 480.2140, found: 480.2139.

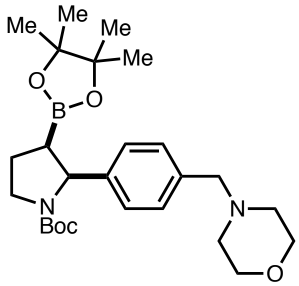

***tert*-butyl-2-(4-(morpholinomethyl)phenyl)-3-(4,4,5,5-tetramethyl-1,3,2-dioxaborolan-2-yl)pyrrolidine-1-carboxylate (28):** The title compound was prepared according to General Procedure B. Crude yield was determined by ^{1}H NMR of the unpurified reaction mixture using 1,3,5-trimethoxybenzene as an NMR standard. Purification by silica gel chromatography (20% acetone in hexanes) yielded **28** as a white solid. Relative stereochemistry and regiochemistry of the major regioisomer for **28** were assigned by analogy to the crystal structure of **20**. Minor regioisomer was assigned by analogy to **37B**; note: rotamers complicated NMR analysis, thus crude rr was approximately assigned as >10:1. Average yield over 2 runs: 77% NMR yield, >20:1

crude dr, >10:1 crude rr. 64% isolated yield, 151 mg (isolated as a ~11:1 regioisomeric mixture, characterization data for the major regioisomer is given).

^{1}H NMR (500 MHz, $CDCl_3$, *2.2:1 mixture of rotamers*) δ 7.25 – 7.15 (m, 4H), 5.09 (d, *J* = 8.1 Hz, 0.3H, *minor rotamer*), 4.96 (d, *J* = 8.3 Hz, 0.7H, *major rotamer*), 3.85 (t, *J* = 9.6 Hz, 0.7H, *major rotamer*), 3.79 – 3.65 (m, 4.3H, *contains minor rotamer*), 3.55 – 3.38 (m, 3H), 2.43 (s, 4H), 2.33 – 2.18 (m, 1H), 2.12 – 1.95 (m, 2H), 1.45 (s, 3H, *minor rotamer*), 1.18 (s, 6H, *major rotamer*), 1.04 (s, 2H, *minor rotamer*), 1.02 (s, 4H, *major rotamer*), 0.93 (s, 2H, *minor rotamer*), 0.91 (s, 4H, *major rotamer*). **^{13}C NMR (126 MHz, $CDCl_3$, *mixture of rotamers*)** δ 154.4, 143.3, 136.1, 129.1, 128.8, 127.1, 126.8, 83.4, 79.1, 67.1, 63.3, 62.9, 53.6, 47.9, 47.3, 28.7, 28.3, 25.0, 24.9, 24.7, 24.6 (signal of carbon directly bonded to boron was not detected because of quadrupolar relaxation). **IR (neat):** 2975 (w), 2802 (w), 1693 (m), 1367 (s), 1330 (m), 1142 (m), 1115 (s), 864 (m), 731 (w) cm^{-1}. **HRMS (ESI+):** Calculated for: $C_{26}H_{42}O_5N_2B$ $[M+H]^+$: 473.3181, found: 473.3190.

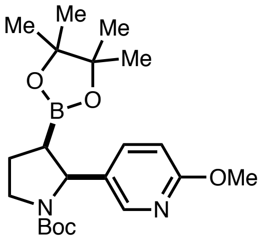

***tert*-butyl-2-(6-methoxypyridin-3-yl)-3-(4,4,5,5-tetramethyl-1,3,2-dioxaborolan-2-yl)pyrrolidine-1-carboxylate (29):** The title compound was prepared according to General Procedure B, but the reaction was run at 30 ºC in an aluminum block. Crude yield was determined by ^{1}H NMR of the unpurified reaction mixture using 1,3,5-trimethoxybenzene as an NMR standard. Purification by silica gel chromatography (20% ethyl acetate in hexanes) yielded **29** as a white solid. Relative stereochemistry and regiochemistry of the major regioisomer for **29** were assigned by analogy to the crystal structure of **20**. Minor regioisomer was assigned by analogy to **37B**; note: rotamers complicated NMR analysis, thus crude rr was approximately assigned as >10:1. Average yield over 2 runs: 50% NMR yield, >20:1 crude dr, >10:1 crude rr. 30% isolated yield, 61 mg (isolated as a ~9:1 regioisomeric mixture, characterization data for the major regioisomer is given).

^{1}H NMR (500 MHz, $CDCl_3$, *1.9:1 mixture of rotamers*) δ 8.05 (s, 0.4H, *minor rotamer*), 8.01 (s, 0.6H, *major rotamer*), 7.45 (d, *J* = 8.7 Hz, 0.3H, *minor rotamer*), 7.42 (dd, *J* = 8.6, 2.5 Hz, 0.7H, *major rotamer*), 6.68 (d, *J* = 8.6 Hz, 0.1H, *minor rotamer*), 6.65 (d, *J* = 8.6 Hz, 0.9H, *major rotamer*), 5.01 (d, *J* = 8.3 Hz, 0.3H, *minor rotamer*), 4.90 (d, *J* = 8.1 Hz, 0.7H, *major rotamer*), 3.90 (s, 2H, *major rotamer*), 3.88 (s, 1H, *minor rotamer*), 3.76 (t, *J* = 9.5 Hz, 0.7H, *major rotamer*), 3.68 (t, *J* = 9.7 Hz, 0.3H, *minor rotamer*), 3.45-3.32 (m, 1H), 2.22-2.08 (m, 1H), 2.07-1.94 (m, 2H), 1.41 (s, 3H, *minor rotamer*), 1.21 (s, 6H, *major rotamer*), 1.01 (s, 2H, *minor rotamer*), 0.99 (s, 4H, *major rotamer*), 0.93 (s, 6H). **^{13}C NMR (126 MHz, $CDCl_3$, *mixture of rotamers*)** δ 163.3, 154.2, 145.7, 145.2, 137.8, 137.6, 132.7, 132.1, 110.1, 109.9, 83.7, 83.5, 79.4, 79.3, 60.3, 60.2, 53.5, 47.8, 47.2, 28.7, 28.6, 28.4, 25.0, 24.9, 24.8, 24.7 (signal of carbon directly bonded to boron was not detected because of quadrupolar relaxation). **IR (neat):** 3043 (w), 2976 (w), 2932 (w), 2853 (w), 1685 (s), 1603 (m), 1492 (m), 1364 (s), 1328 (s), 1286 (s), 1164 (m), 1142 (s), 1124 (s),

1025 (m), 829 (m), 577 (m) cm^{-1}. **HRMS (ESI+):** Calculated for: $C_{21}H_{34}O_5N_2B$ $[M+H]^+$: 405.2555, found: 405.2563.

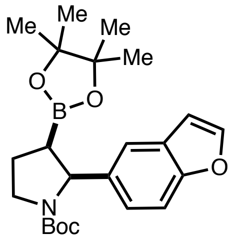

***tert*-butyl-2-(benzofuran-5-yl)-3-(4,4,5,5-tetramethyl-1,3,2-dioxaborolan-2-yl)pyrrolidine-1-carboxylate (30):** The title compound was prepared according to General Procedure B. Crude yield was determined by ^{1}H NMR of the unpurified reaction mixture using 1,3,5-trimethoxybenzene as an NMR standard. Purification by silica gel chromatography (10% ethyl acetate in hexanes) yielded **30** as a white foam. Relative stereochemistry and regiochemistry of the major regioisomer for **30** were assigned by analogy to the crystal structure of **20**. Minor regioisomer was assigned by analogy to **37B**; note: rotamers complicated NMR analysis, thus crude rr was approximately assigned as >10:1. Average yield over 2 runs: 48% NMR yield, >20:1 crude dr, >10:1 crude rr. 37% isolated yield, 76 mg (isolated as a single regioisomer).

^{1}H NMR (500 MHz, $CDCl_3$, *2.1:1 mixture of rotamers*) δ 7.60 – 7.53 (m, 1H), 7.45 (s, 0.3H, *minor rotamer*), 7.41 (s, 0.7H, *major rotamer*), 7.37 (d, *J* = 8.5 Hz, 1H), 7.16 (t, *J* = 10.2 Hz, 1H), 6.73 – 6.66 (m, 1H), 5.17 (d, *J* = 8.0 Hz, 0.3H, *minor rotamer*), 5.06 (d, *J* = 8.3 Hz, 0.7H, *major rotamer*), 3.86 (t, *J* = 9.6 Hz, 0.7H, *major rotamer*), 3.77 (t, *J* = 9.6 Hz, 0.3H, *minor rotamer*), 3.52 – 3.36 (m, 1H), 2.35 – 2.21 (m, 1H), 2.11 – 1.94 (m, 2H), 1.42 (s, 3H, *minor rotamer*), 1.13 (s, 6H, *major rotamer*), 0.93 (s, 6H), 0.79 (s, 6H). **^{13}C NMR (126 MHz, $CDCl_3$, *mixture of rotamers*)** δ 154.5, 154.3, 154.2, 154.1, 145.2, 145.1, 139.1, 138.4, 127.3, 127.0, 123.7, 123.3, 119.4, 119.3, 110.8, 110.4, 106.7, 106.6, 83.3, 83.2, 79.2, 79.1, 63.1, 62.9, 48.0, 47.4, 28.7, 28.3, 25.7, 24.8, 24.6, 24.5 (signal of carbon directly bonded to boron was not detected because of quadrupolar relaxation). **IR (neat):** 2976 (w), 2878 (w), 1687 (m), 1363 (s), 1326 (m), 1121 (m), 857 (m), 732 (m) cm^{-1}. **HRMS (ESI+):** Calculated for: $C_{23}H_{32}O_5NBNa$ $[M+Na]^+$: 436.2266, found: 436.2266.

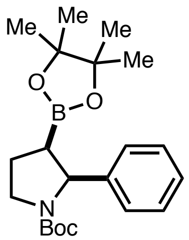

***tert*-butyl-2-phenyl-3-(4,4,5,5-tetramethyl-1,3,2-dioxaborolan-2-yl)pyrrolidine-1-carboxylate (31):** The title compound was prepared according to General Procedure B. Crude yield was determined by ^{1}H NMR of the unpurified reaction mixture using mesitylene as an NMR standard. Purification by MPLC (gradient: 0-10% ethyl acetate in hexanes) yielded **31** as a white solid. Relative stereochemistry and regiochemistry of the major regioisomer for **31** were assigned by analogy to the crystal structure of **20**. Minor regioisomer was assigned by analogy to **37B**; note: rotamers complicated NMR analysis, thus crude rr was approximately assigned as >10:1. Average yield over 2 runs: 88% NMR yield, >20:1 crude dr, >10:1 crude rr. 66% isolated yield, 123 mg

(isolated as a ~11:1 regioisomeric mixture, characterization data for the major regioisomer is given).

^{1}H NMR (400 MHz, CDCl$_3$, *2.6:1 mixture of rotamers*) δ 7.33 – 7.11 (m, 5H), 5.05 (d, *J* = 8.2 Hz, 0.3H, *minor rotamer*), 4.93 (d, *J* = 8.6 Hz, 0.7H, *major rotamer*), 3.83 (t, *J* = 9.3 Hz, 0.7H, *major rotamer*), 3.72 (t, *J* = 9.6 Hz, 0.3H, *minor rotamer*), 3.49-3.32 (m, 1H), 2.32 – 2.13 (m, 1H), 2.11 – 1.90 (m, 2H), 1.41 (s, 3H, *minor rotamer*), 1.15 (s, 6H, *major rotamer*), 0.98 (s, 6H), 0.87 (s, 6H). **^{13}C NMR (101 MHz, CDCl$_3$, *mixture of rotamers*)** δ 154.3, 144.3, 143.6, 128.0, 127.8, 127.1, 126.8, 126.7, 83.3, 79.0, 63.1, 62.8, 47.8, 47.3, 28.6, 28.2, 24.9, 24.8, 24.6 (carbon directly bonded to boron was not detected because of quadrupole relaxation). **IR (neat):** 3026 (w), 2975 (w), 2871 (w), 1685 (s), 1383 (s), 1326 (m), 1312 (m), 1165 (m), 1144 (m), 1123 (m), 754 (m), 697 (m), 573 (m) cm^{-1}. **HRMS (ESI+):** Calculated for: $C_{21}H_{32}O_4NBNa$ [M+Na]$^+$: 396.2317, found: 396.2320.

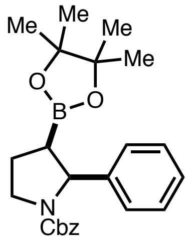

benzyl-2-phenyl-3-(4,4,5,5-tetramethyl-1,3,2-dioxaborolan-2-yl)pyrrolidine-1-carboxylate (32): The title compound was prepared according to General Procedure B. Crude yield was determined by ^{1}H NMR of the unpurified reaction mixture using mesitylene as an NMR standard. Purification by silica gel chromatography (10% ethyl acetate in hexanes) yielded **32** as a white solid. Relative stereochemistry and regiochemistry of the major regioisomer for **32** were assigned by analogy to the crystal structure of **20**. Minor regioisomer was assigned by analogy to **37B**. Average yield over 2 runs: 67% NMR yield, >20:1 crude dr, 9:1 crude rr. 59% isolated yield, 119 mg (isolated as a ~13:1 regioisomeric mixture, characterization data for the major regioisomer is given).

^{1}H NMR (400 MHz, CDCl$_3$) δ 7.46 – 7.10 (m, 9H), 6.85 (d, *J* = 6.6 Hz, 1H), 5.19 – 4.88 (m, 3H), 3.85 (dt, *J* = 17.5, 9.6 Hz, 1H), 3.58 – 3.42 (m, 1H), 2.39 – 2.14 (m, 1H), 2.14 – 1.91 (m, 2H), 1.00 (s, 6H), 0.88 (s, 6H). **^{13}C NMR (101 MHz, CDCl$_3$, *mixture of rotamers*)** δ 154.7, 154.5, 143.6, 143.1, 137.2, 137.0, 128.5, 128.4, 128.3, 128.2, 128.1, 128.0, 127.9, 127.8, 127.7, 127.4, 127.1, 127.0, 126.9, 83.6, 83.4, 66.7, 66.4, 63.5, 63.0, 48.0, 47.6, 27.2, 25.9, 24.9, 24.8, 24.6, 24.5 (carbon directly bonded to boron was not detected because of quadrupole relaxation). **IR (neat):** 3062 (w), 2973 (w), 1699 (s), 1351 (s), 1142 (m), 1111 (s), 700 (s), 570 (m) cm^{-1}. **HRMS (ESI+):** Calculated for: $C_{24}H_{30}NBNa$ [M+Na]$^+$: 430.2160, found: 430.2164.

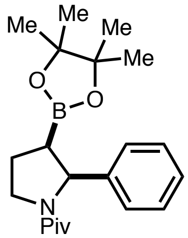

2,2-dimethyl-1-((2*S*,3*R*)-2-phenyl-3-(4,4,5,5-tetramethyl-1,3,2-dioxaborolan-2-yl)pyrrolidin-1-yl)propan-1-one (33): The title compound was prepared according to General Procedure B, except that the reaction was run at 30 ºC, and the crude residue was washed 3 X 1 M NaOAc instead of 1 M KOH. Crude yield was determined by ^{1}H NMR of the unpurified reaction mixture using 1,3,5-trimethoxybenzene as an NMR standard. Purification by silica gel chromatography (20% ethyl acetate in hexanes) yielded **33** as a white solid. Relative stereochemistry and regiochemistry of the major regioisomer for **33** were assigned by analogy to the crystal structure of **20**. Minor regioisomer was assigned by analogy to **37B**; note: rotamers complicated NMR analysis, thus crude rr was approximately assigned as >10:1. Average yield over 2 runs: 61% NMR yield, >20:1 crude dr, >10:1 crude rr. 37% isolated yield, 67 mg (isolated as a >10:1 regioisomeric mixture, characterization data for the major regioisomer is given).

^{1}H NMR (500 MHz, $CDCl_3$, *mixture of rotamers*) δ 7.25 – 7.07 (m, 5H), 5.37 (br s, 1H), 4.05 (br s, 1H), 3.66 (td, J = 10.4, 6.7 Hz, 1H), 2.32 (br s, 1H), 2.04 (br s, 1H), 1.87 (br s, 1H), 1.25 (br s, 2H, *minor rotamer*), 1.23 (s, 7H, *major rotamer*), 1.01 (s, 6H), 0.89 (s, 6H). **^{13}C NMR (126 MHz, $CDCl_3$, *mixture of rotamers*)** δ 175.8, 143.7, 128.1, 126.8, 126.7, 83.6, 83.4, 65.0, 49.1, 39.4, 27.6, 25.1, 25.0, 24.7 (carbon directly bonded to boron was not detected because of quadrupole relaxation). **IR (neat):** 2973 (w), 2876 (w), 1625 (s), 1358 (s), 1319 (s), 1140 (s), 850 (m), 698 (s) cm^{-1}. **HRMS (ESI+):** Calculated for $C_{21}H_{32}O_3NBNa$ $[M+Na]^+$: 380.2367, found: 380.2372.

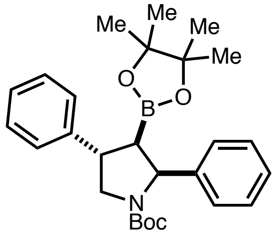

***tert*-butyl-2,4-diphenyl-3-(4,4,5,5-tetramethyl-1,3,2-dioxaborolan-2-yl)pyrrolidine-1-carboxylate (34):** The title compound was prepared according to General Procedure B. Crude yield was determined by ^{1}H NMR of the unpurified reaction mixture using mesitylene as an NMR standard. Purification by silica gel chromatography (5% ethyl acetate in hexanes) yielded **34** as a white solid. Crystals suitable for X-ray crystallography were obtained *via* slow diffusion of pentane into a saturated solution of **34** in diethyl ether (see Supporting Information part 15 for crystallography data). Minor regioisomer was assigned by analogy to **37B**; note: rotamers complicated NMR analysis, thus crude rr was approximately assigned as >10:1. Average yield over 2 runs: 64% NMR yield, >20:1 crude dr, >10:1 crude rr. 54% isolated yield, 121 mg (isolated as a single regioisomer).

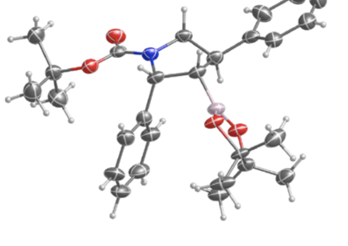

^{1}H NMR (500 MHz, CDCl$_3$, *2.2:1 mixture of rotamers*) δ 7.30 – 7.13 (m, 10H), 5.19 (d, J = 9.0 Hz, 0.3H, *minor rotamer*), 5.05 (d, J = 9.1 Hz, 0.7H, *major rotamer*), 4.14 (dd, J = 10.8, 8.2 Hz, 0.7H, *major rotamer*), 4.03 (app. t, J = 9.4 Hz, 0.3H, *minor rotamer*), 3.83 – 3.69 (m, 1H), 3.49 (t, J = 10.7 Hz, 0.7H, *major rotamer*), 3.40 (t, J = 10.7 Hz, 0.3H, *minor rotamer*), 2.23 (dd, J = 12.8, 9.2 Hz, 1H), 1.38 (s, 3H, *minor rotamer*), 1.13 (s, 6H, *major rotamer*), 0.79 (s, 6H), 0.74 (s, 6H). **^{13}C NMR (126 MHz, CDCl$_3$, *mixture of rotamers*)** δ 154.3, 154.1, 144.2, 143.4, 141.1, 141.0, 128.4, 128.3, 128.1, 128.0, 127.1, 127.0, 126.9, 126.8, 126.6, 83.4, 79.5, 79.4, 64.1, 63.8, 55.2, 54.3, 44.6, 43.8, 28.7, 28.3, 24.8, 24.5 (carbon directly bonded to boron was not detected because of quadrupole relaxation). **IR (neat):** 3028 (w), 2976 (w), 2925 (w), 2869 (w), 1677 (s), 1366 (s), 1329 (m), 1141 (s), 970 (m), 847 (m), 743 (m), 695 (s), 579 (m) cm^{-1}. **HRMS (ESI+):** Calculated for: $C_{27}H_{36}O_4NBNa$ [M+Na]$^+$: 472.2630, found: 472.2628.

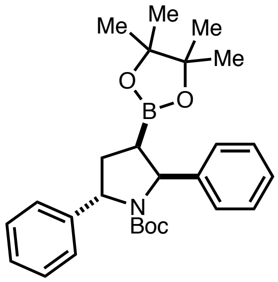

***tert*-butyl-2,5-diphenyl-3-(4,4,5,5-tetramethyl-1,3,2-dioxaborolan-2-yl)pyrrolidine-1-carboxylate (35):** The title compound was prepared according to General Procedure B. Crude yield was determined by ^{1}H NMR of the unpurified reaction mixture using mesitylene as an NMR standard. Purification by silica gel chromatography (10% ethyl acetate in hexanes) yielded **35** as a white solid. Crystals suitable for X-ray crystallography were obtained *via* slow diffusion of pentane into a saturated solution of **35** in diethyl ether (see Supporting Information part 15 for crystallography data). Relative stereochemistry for related products (**38-39**) was assigned by analogy to the crystal structure of **35**. Minor regioisomer was assigned by analogy to **37B**; note: rotamers complicated NMR analysis, thus crude rr was approximately assigned as >10:1. Average yield over 2 runs: 63% NMR yield, >20:1 crude dr, >10:1 crude rr. 62% isolated yield, 139 mg (isolated as a single regioisomer).

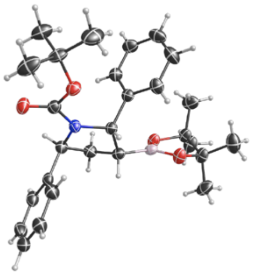

^{1}H NMR (500 MHz, CDCl$_3$, *1.5:1 mixture of rotamers*) δ 7.34 – 7.13 (m, 10H), 5.36 (d, J = 8.4 Hz, 0.6H, *major rotamer*), 5.32 (d, J = 8.9 Hz, 0.4H, *minor rotamer*), 5.17 (app. t, J = 7.9 Hz, 1H), 2.74 (tdd, J = 11.6, 8.4, 2.6 Hz, 1H), 2.20 (ddt, J = 14.9, 9.0, 4.6 Hz, 1H), 1.80 (dd, J = 12.9, 6.2 Hz, 1H), 1.09 (s, 3H, *minor rotamer*), 1.06 (s, 6H, *major rotamer*), 0.94 (s, 2H, *minor rotamer*), 0.93 (s, 4H, *major rotamer*), 0.82 (s, 2H, *minor rotamer*), 0.80 (s, 4H, *major rotamer*). **^{13}C NMR (126 MHz, CDCl$_3$, *mixture of rotamers*)** δ 154.0, 153.8, 145.5, 144.3, 144.2, 143.3, 128.5, 128.3, 128.2, 128.0, 127.1, 126.9, 126.8, 126.7, 126.6, 125.4, 125.3, 83.4, 79.5, 79.4, 64.5, 64.0, 62.6, 61.8, 34.9, 34.3, 28.2, 28.1, 25.0, 24.9, 24.7, 24.6 (carbon directly bonded to boron was not detected because of quadrupole relaxation). **IR (neat):** 3032 (w), 2975 (w), 2877 (w), 1691 (s), 1365 (s), 1333 (m), 1169 (m), 1136 (m), 1122 (m), 849 (w), 754 (w), 690 (m) cm^{-1}. **HRMS (ESI+):** Calculated for: $C_{27}H_{36}O_4NBNa$ [M+Na]$^+$: 472.2630, found: 472.2627.

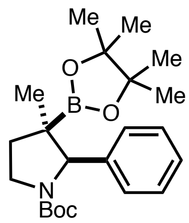

tert-butyl-3-methyl-2-phenyl-3-(4,4,5,5-tetramethyl-1,3,2-dioxaborolan-2-yl)pyrrolidine-1-carboxylate (36): The title compound was prepared according to General Procedure B. Crude yield was determined by ^{1}H NMR of the unpurified reaction mixture using mesitylene as an NMR standard. Purification by silica gel chromatography (10% ethyl acetate in hexanes) yield **36** as a white solid. Crystals suitable for X-ray crystallography were obtained *via* slow diffusion of a saturated solution of **36** in diethyl ether to identity the major regioisomer (see Supporting Information part 15 for crystallography data). Minor regioisomer was assigned by analogy to **37B**; note: rotamers complicated NMR analysis, thus crude rr was approximately assigned as 5:1. Average yield over 2 runs: 71% NMR yield, >20:1 crude dr, 5:1 crude rr. 69% isolated yield, 134 mg (isolated as a ~5:1 regioisomeric mixture, characterization data for the major regioisomer is given).

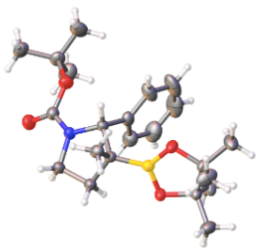

^{1}H NMR (500 MHz, $CDCl_3$, *2.6:1 mixture of rotamers*) δ 7.33 – 7.11 (m, 5H), 4.61 (s, 0.2H, *minor rotamer*), 4.44 (s, 0.8H, *major rotamer*), 3.85 – 3.41 (m, 2H), 2.59 (app. p, *J* = 10.9 Hz, 0.2H, *minor rotamer*), 2.41 (dt, *J* = 13.0, 9.7 Hz, 0.8H, *major rotamer*), 1.95-1.85 (m, 0.2H, *minor rotamer*), 1.67-1.58 (m, 0.8H, *major rotamer*), 1.48 (s, 1H, *minor rotamer*), 1.42 (s, 2H, *major rotamer*), 1.21 (s, 2H, *minor rotamer*), 1.13 (s, 7H, *major rotamer*), 0.98 (s, 2H, *minor rotamer*), 0.96 (s, 4H, *major rotamer*), 0.86 (s, 1H, *minor rotamer*), 0.83 (s, 5H, *major rotamer*). **^{13}C NMR (126 MHz, $CDCl_3$, *mixture of rotamers and regioisomers*)** δ 155.1, 154.8, 154.7, 154.6, 146.2, 145.9, 144.0, 143.3, 128.2, 128.1, 128.0, 127.8, 127.1, 127.0, 126.9, 126.8, 126.7, 126.5, 126.3, 83.4, 83.3, 83.2, 79.3, 79.1, 79.0, 78.9, 71.1, 70.6, 48.4, 47.5, 45.6, 45.2, 45.0, 44.6, 35.3, 34.0, 32.3, 31.6, 31.1, 30.9, 28.7, 28.6, 28.2, 24.9, 24.8, 24.7, 24.6, 24.5, 24.4, 23.3, 23.2 (carbon directly bonded to boron was not detected because of quadrupole relaxation). **IR (neat):** 3062 (w), 2978 (w), 2927 (w), 2874 (w), 1691 (s), 1378 (s), 1364 (s), 1322 (s), 1178 (m), 1145 (m), 1129 (s), 853 (m), 700 (m) cm^{-1}. **HRMS (ESI+):** Calculated for: $C_{22}H_{34}O_4NBNa$ $[M+Na]^+$: 410.2473, found: 410.2474.

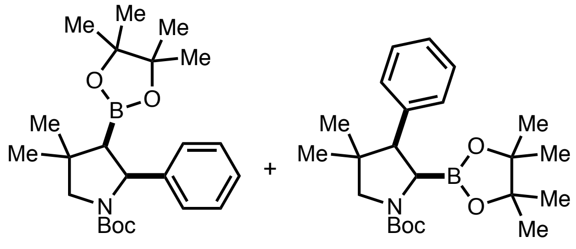

A B

tert-butyl-4,4-dimethyl-2-phenyl-3-(4,4,5,5-tetramethyl-1,3,2-dioxaborolan-2-yl)pyrrolidine-1-carboxylate (A) + _tert_-butyl-4,4-dimethyl-3-phenyl-2-(4,4,5,5-tetramethyl-1,3,2-dioxaborolan-2-yl)pyrrolidine-1-carboxylate (B) (37): The title compound was prepared according to General Procedure B. Crude yield was determined by ^{1}H NMR of the unpurified

reaction mixture using mesitylene as an NMR standard. Purification by silica gel chromatography (10% ethyl acetate in hexanes) yielded **37** as a white solid and regioisomeric mixture. Further purification by MPLC (gradient: 0-5% ethyl acetate in hexanes) allows for separation of the regioisomers. The minor regioisomers for all other related products (**20-39**) were assigned by comparison to regioisomer **37B**. Average yield over 2 runs: 90% NMR yield, >20:1 crude dr, 1:1.6 crude rr (A:B). 75% isolated yield, 151 mg (isolated as 1:1.5 A:B regioisomeric mixture, NMR data for the mixture of regioisomers is given. The mixture was further purified *via* MPLC (gradient: 0-5% ethyl acetate in hexanes) to separate and characterize the regioisomers, and full characterization data for the individual regioisomers is given).

Mixture of Regioisomers A/B: **^{1}H NMR (500 MHz, $CDCl_3$, *1:1.5 mixture of regioisomers A/B and mixture of rotamers*)** δ 7.28 – 7.08 (m, 14H), 5.17 (d, *J* = 8.9 Hz, 0.3H, *minor rotamer*), 5.04 (d, *J* = 8.7 Hz, 0.7H, *major rotamer*), 3.89 (app. t, *J* = 6.6 Hz, 1.5H), 3.64 – 3.26 (m, 5H), 3.02 (app. t, *J* = 8.4 Hz, 1.5H), 1.89 (d, *J* = 9.3 Hz, 0.3H, *minor rotamer*), 1.85 (d, *J* = 8.6 Hz, 0.7H, *major rotamer*), 1.50 (rotameric d, *J* = 2.9 Hz, 14H), 1.20 – 1.10 (m, 17H), 1.04 – 0.92 (m, 16H), 0.88 – 0.78 (m, 15H), 0.64 (s, 2H), 0.59 (s, 2H). **^{13}C NMR (126 MHz, $CDCl_3$, *mixture of regioisomers and rotamers*)** δ 155.6, 155.2, 155.1, 155.0, 143.0, 141.9, 141.5, 141.3, 129.5, 129.3, 128.0, 127.9, 127.7, 127.5, 126.7, 126.6, 126.5, 126.4, 126.3, 126.2, 83.5, 83.4, 83.1, 79.4, 79.2, 79.0, 62.8, 62.5, 60.2, 59.2, 58.4, 58.3, 58.2, 56.8, 43.0, 41.5, 40.2, 39.7, 29.4, 28.9, 28.7, 28.6, 28.5, 28.1, 25.3, 25.2, 25.1, 25.0, 24.9, 24.4, 24.3, 23.9, 23.7, 23.6, 22.7, 22.5 (carbons directly bonded to boron was not detected because of quadrupole relaxation).

***tert*-butyl-4,4-dimethyl-2-phenyl-3-(4,4,5,5-tetramethyl-1,3,2-dioxaborolan-2-yl)pyrrolidine-1-carboxylate (Regioisomer A): ^{1}H NMR (500 MHz, $CDCl_3$, *2.2:1 mixture of rotamers*)** δ 7.30 – 7.06 (m, 5H), 5.16 (d, *J* = 8.9 Hz, 0.3H, *minor rotamer*), 5.04 (d, *J* = 8.7 Hz, 0.7H, *major rotamer*), 3.63 – 3.39 (m, 2H), 1.88 (d, *J* = 8.9 Hz, 0.3H, *minor rotamer*), 1.84 (d, *J* = 8.7 Hz, 0.7H, *major rotamer*), 1.46 (s, 3H), 1.19 – 1.08 (m, 12H), 0.97 (s, 4H, *major rotamer*), 0.93 (s, 2H, *minor rotamer*), 0.80 (s, 6H). **^{13}C NMR (126 MHz, $CDCl_3$, *mixture of rotamers*)** δ 155.3, 155.2, 143.1, 142.0, 128.0, 127.6, 126.8, 126.4, 126.3, 126.2, 83.2, 79.3, 79.1, 62.9, 62.6, 60.2, 59.3, 40.2, 39.7, 28.8, 28.7, 28.1, 25.4, 25.3, 24.5, 24.4, 23.8, 23.7 (carbon directly bonded to boron was not detected because of quadrupole relaxation). **IR (neat):** 3000 (w), 2974 (w), 2881 (w), 1683 (s), 1391 (s), 1366 (s), 1338 (m), 1140 (s), 697 (m) cm^{-1}. **HRMS (ESI+):** Calculated for: $C_{23}H_{36}O_4NBNa$ $[M+Na]^+$: 424.2630, found: 424.2635.

***tert*-butyl-4,4-dimethyl-3-phenyl-2-(4,4,5,5-tetramethyl-1,3,2-dioxaborolan-2-yl)pyrrolidine-1-carboxylate (Regioisomer B): ^{1}H NMR (500 MHz, $CDCl_3$, *1:1 mixture of rotamers*)** δ 7.31 – 7.11 (m, 5H), 3.88 (app. t, *J* = 6.9 Hz, 1H), 3.49 (d, *J* = 11.1 Hz, 0.5H, *rotamer*), 3.37 (dd, *J* = 15.9, 10.7 Hz, 1H), 3.30 (d, *J* = 10.9 Hz, 0.5H, *rotamer*), 3.02 (app. t, *J* = 8.1 Hz, 1H), 1.50 (d, *J* = 2.8 Hz, 9H), 1.17 (s, 1.5H, *rotamer*), 1.16 (s, 1.5H, *rotamer*), 1.00 (s, 3H, *rotamer*), 0.95 (s, 3H, *rotamer*), 0.85 (s, 3H, *rotamer*), 0.83 (s, 3H, *rotamer*), 0.64 (s, 1.5H, *rotamer*), 0.59 (s, 1.5H, *rotamer*). **^{13}C NMR (126 MHz, $CDCl_3$, mixture of rotamers)** δ 155.7, 155.1, 141.6, 141.4, 129.6, 129.3, 128.0, 127.8, 126.7, 126.5, 83.6, 83.5, 79.4, 79.0, 58.5, 58.4, 58.3, 56.8, 43.1, 41.5, 29.4, 29.0, 28.7, 25.2, 25.0, 24.9, 23.9, 22.8, 22.5 (carbon directly bonded to boron was not detected because of quadrupole relaxation). **IR (neat):** 2974 (w), 2874 (w), 1666 (m), 1407 (m), 1378 (s), 1340 (m), 1145 (s), 698 (m) cm^{-1}. **HRMS (ESI+):** Calculated for: $C_{23}H_{36}O_4NBNa$ $[M+Na]^+$: 424.2630, found: 424.2634.

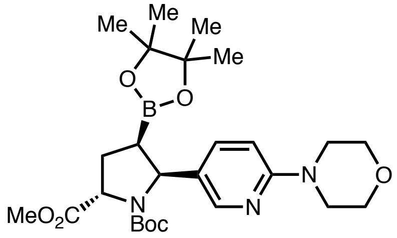

1-(*tert*-butyl) 2-methyl (2*S*,4*R*,5*S*)-5-(6-morpholinopyridin-3-yl)-4-(4,4,5,5-tetramethyl-1,3,2-dioxaborolan-2-yl)pyrrolidine-1,2-dicarboxylate ((*S*)-38): The title compound was prepared according to General Procedure B, but the reaction was run at 30 °C in an aluminum block, the alkene was added as a solution in THF, and the crude mixture was washed with 1 M NaOAc instead of 1 M KOH. Crude yield was determined by ^{1}H NMR of the unpurified reaction mixture using mesitylene as an NMR standard. Purification by MPLC (gradient: 0-40% ethyl acetate in hexanes) yielded **(*S*)-38** as a white foam. Relative stereochemistry and regiochemistry of the major regioisomer for **(*S*)-38** were assigned by analogy to the crystal structure of **35**. Minor regioisomer was assigned by analogy to **37B**; note: rotamers complicated NMR analysis, thus crude rr was approximately assigned as >10:1. Average yield over 2 runs: 51% NMR yield, >20:1 crude dr, >10:1 crude rr. 39% isolated yield, 101 mg (isolated with 5% B_2pin_2 and as a single regioisomer).

^{1}H NMR (500 MHz, $CDCl_3$, *1.2:1 mixture of rotamers*) δ 8.07 (d, *J* = 2.4 Hz, 0.5H, *minor rotamer*), 8.05 (d, *J* = 2.4 Hz, 0.5H, *major rotamer*), 7.38 (dd, *J* = 8.7, 2.5 Hz, 0.4H, *minor rotamer*), 7.32 (dd, *J* = 8.7, 2.5 Hz, 0.6H, *major rotamer*), 6.55 (d, *J* = 3.0 Hz, 0.6H, *major rotamer*), 6.53 (d, *J* = 3.0 Hz, 0.4H, *minor rotamer*), 5.12 (d, *J* = 8.8 Hz, 0.4H, *minor rotamer*), 5.01 (d, *J* = 8.9 Hz, 0.6H, *major rotamer*), 4.60 (d, *J* = 8.9 Hz, 0.6H, *major rotamer*), 4.49 (d, *J* = 9.0 Hz, 0.4H, *minor rotamer*), 3.78 (dt, *J* = 10.2, 4.9 Hz, 4H), 3.72 (s, 1.6H, *major rotamer*), 3.71 (s, 1.4H, *minor rotamer*), 3.48 – 3.34 (m, 4H), 2.59 – 2.44 (m, 1H), 2.29 – 2.14 (m, 1H), 2.04 (dd, *J* = 13.6, 6.6 Hz, 1H), 1.33 (s, 4H, *minor rotamer*), 1.20 (s, 5H, *major rotamer*), 0.97 (s, 2.8H, *minor rotamer*), 0.95 (s, 3.2H, *major rotamer*), 0.90 (s, 2.8H, *minor rotamer*), 0.88 (s, 3.2H, *major rotamer*). **^{13}C NMR (126 MHz, $CDCl_3$, *mixture of rotamers*)** δ 173.7, 173.3, 159.3, 159.2, 154.1, 153.3, 146.8, 146.3, 136.8, 136.2, 129.3, 128.6, 106.7, 106.5, 83.6, 83.5, 80.2, 80.1, 66.8, 66.7, 61.0, 60.9, 60.5, 60.0, 52.3, 52.1, 46.3, 46.2, 30.9, 29.9, 28.4, 28.3, 24.8, 24.7, 24.6, 24.5 (carbon directly bonded to boron was not detected because of quadrupole relaxation). **IR (neat):** 2982 (w), 1692 (m), 1604 (m), 1486 (m), 1363 (s), 1169 (m), 1120 (s), 943 (m) cm^{-1}. **HRMS (ESI+):** Calculated for: $C_{26}H_{41}O_7N_3B$ [M+H]$^+$: 518.3032, found: 518.3040.

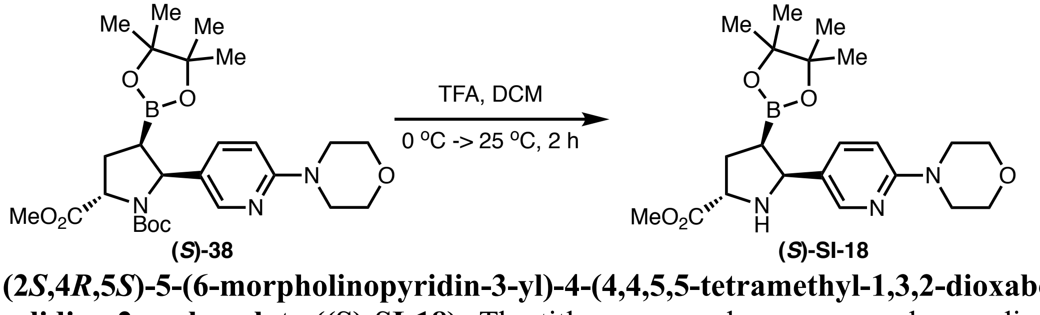

methyl (2*S*,4*R*,5*S*)-5-(6-morpholinopyridin-3-yl)-4-(4,4,5,5-tetramethyl-1,3,2-dioxaborolan-2-yl)pyrrolidine-2-carboxylate ((S)-SI-18): The title compound was prepared according to the following literature procedure[24]: An oven-dried 1-dram vial equipped with a stir bar is charged with 1-(*tert*-butyl) 2-methyl (2*S*,4*R*,5*S*)-5-(6-morpholinopyridin-3-yl)-4-(4,4,5,5-tetramethyl-

1,3,2-dioxaborolan-2-yl)pyrrolidine-1,2-dicarboxylate **(*S*)-38** (51.7 mg, 0.100 mmol, 1.00 equiv.) and evacuated/backfilled with N_2 X 3. DCM (0.300 mL) was added *via* syringe, and the mixture was cooled to 0 °C in an ice water bath. TFA (154 µL, 2.00 mmol, 20.0 equiv.) was added dropwise *via* syringe, and then the reaction was warmed to room temperature and stirred for 2 hours. The reaction was concentrated *in vacuo*, and the crude residue was re-dissolved in ethyl acetate (1 mL) and slowly neutralized with sat. aq. $NaHCO_3$ (1 mL). The aqueous layer was extracted with ethyl acetate (3 X 1 mL). The combined organic layers were dried over $MgSO_4$, gravity filtered, and concentrated *in vacuo*. Purification by silica gel chromatography (30% acetone in hexanes) yielded 9.2 mg **(*S*)-SI-18** as a white solid, 22%. The enantiomeric purity was established via HPLC analysis using a chiral column. A racemic sample was obtained by preparing a 1:1 mixture of **(*S*)-SI-18** and **(*R*)-SI-18**.

^{1}H NMR (500 MHz, $CDCl_3$) δ 8.14 (d, *J* = 2.4 Hz, 1H), 7.52 (dd, *J* = 8.7, 2.5 Hz, 1H), 6.57 (d, *J* = 8.7 Hz, 1H), 4.61 (d, *J* = 8.7 Hz, 1H), 4.12 (dd, *J* = 8.5, 3.1 Hz, 1H), 3.80 (app. t, *J* = 4.9 Hz, 4H), 3.73 (s, 3H), 3.50 – 3.38 (m, 4H), 2.85 – 2.24 (m, 2H), 2.17 (ddd, *J* = 13.0, 7.8, 3.1 Hz, 1H), 1.92 (dt, *J* = 10.2, 8.1 Hz, 1H), 0.97 (s, 6H), 0.96 (s, 6H). **^{13}C NMR (126 MHz, $CDCl_3$)** δ 176.4, 159.3, 146.8, 136.9, 130.5, 106.8, 83.3, 66.9, 60.8, 60.5, 52.3, 46.4, 32.0, 24.9, 24.7 (carbon directly bonded to boron was not detected because of quadrupole relaxation). **IR (neat):** 2954 (w), 2824 (w), 2160 (w), 1741 (m), 1604 (m), 1374 (m), 1139 (s), 1114 (s), 939 (m) cm^{-1}. **HRMS (ESI+):** Calculated for: $C_{21}H_{33}O_5N_3B$ $[M+H]^+$: 418.2508, found: 418.2514. **HPLC:** ChiralPak IA, 22 °C, 0.5 mL/min, 80:20 hexane:isopropanol, 254 nm, t_R = 25.895 min, t_s = 27.862 min, 99:1 er. See Supporting Information part 14 for HPLC chromatographs.

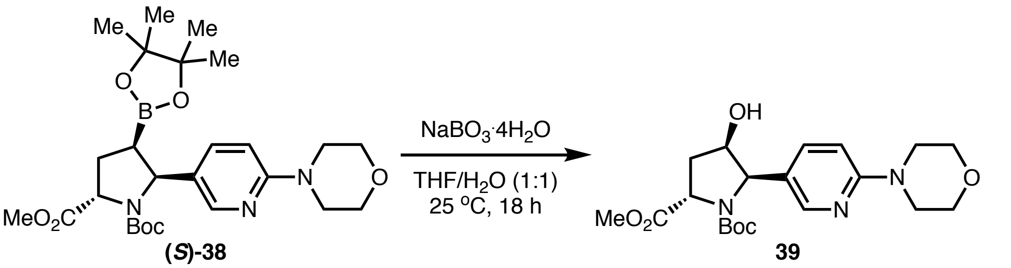

methyl (2*S*,4*R*,5*R*)-4-hydroxy-5-(6-morpholinopyridin-3-yl)pyrrolidine-2-carboxylate (39): The title compound was prepared by oxidizing **(*S*)-38** according to General Procedure C on 0.05 mmol scale. After azeotroping with toluene (3 X 1 mL) to remove pinacol, 18.7 mg of **39** were obtained as a white foam, 91% (isolated with 4% B_2pin_2).

^{1}H NMR (500 MHz, $CDCl_3$, *1.1:1 mixture of rotamers*) δ 8.01 (dd, *J* = 4.3, 2.4 Hz, 1H), 7.33 (ddd, *J* = 12.0, 8.9, 2.5 Hz, 1H), 6.63 (d, *J* = 8.8 Hz, 1H), 5.02 (d, *J* = 7.2 Hz, 0.5H, *major rotamer*), 4.93 (d, *J* = 7.1 Hz, 0.5H, *minor rotamer*), 4.65 – 4.54 (m, 1.5H, *contains minor rotamer*), 4.50 (dd, *J* = 8.9, 2.9 Hz, 0.5H, *major rotamer*), 3.80 (dt, *J* = 10.2, 4.9 Hz, 4H), 3.76 (s, 1.6H, *major rotamer*), 3.76 (s, 1.4H, *minor rotamer*), 3.48 (app. q, *J* = 5.1 Hz, 4H), 2.25 – 2.18 (m, 1H), 2.18 – 2.08 (m, 1H), 1.92 – 1.50 (br s, 1H), 1.36 (s, 4.4H, *minor rotamer*), 1.22 (d, *J* = 1.7 Hz, 4.6H, *major rotamer*). **^{13}C NMR (126 MHz, $CDCl_3$, *mixture of rotamers*)** δ 173.5, 173.2, 159.5, 159.4, 154.2, 153.3, 147.1, 146.6, 137.3, 137.0, 123.0, 122.0, 106.9, 106.6, 80.9, 80.7, 71.2, 70.4, 66.9, 66.8, 62.1, 61.7, 58.1, 57.4, 52.5, 52.4, 45.8, 45.7, 35.9, 35.3, 28.3, 28.2. **IR (neat):** 3316 (br, w), 2969 (w), 2855 (w), 1747 (m), 1692 (m), 1606 (m), 1494 (m), 1381 (s), 1365 (s), 1113 (s) cm^{-1}. **HRMS (ESI+):** Calculated for: $C_{20}H_{30}O_6N_3$ $[M+H]^+$: 408.2129, found: 408.2133.

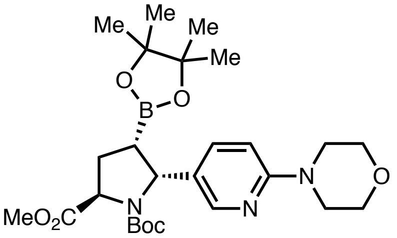

1-(*tert*-butyl) 2-methyl (2*R*,4*S*,5*R*)-5-(6-morpholinopyridin-3-yl)-4-(4,4,5,5-tetramethyl-1,3,2-dioxaborolan-2-yl)pyrrolidine-1,2-dicarboxylate ((*R*)-38): The title compound was prepared according to General Procedure B, but the reaction was run at 30 °C in an aluminum block, the alkene was added as a solution in THF, and the crude mixture was washed with 1 M NaOAc instead of 1 M KOH. Crude yield was determined by ^{1}H NMR of the unpurified reaction mixture using mesitylene as an NMR standard. Purification by MPLC (gradient: 0-40% ethyl acetate in hexanes) yielded **(*R*)-38** as a white foam. Relative stereochemistry and regiochemistry of the major regioisomer for **(*R*)-38** were assigned by analogy to the crystal structure of **35**. Minor regioisomer was assigned by analogy to **37B**; note: rotamers complicated NMR analysis, thus crude rr was approximately assigned as >10:1. Yield: 53% NMR yield, >20:1 crude dr, >10:1 crude rr. 44% isolated yield, 113 mg (isolated as a ~17:1 mixture of regioisomers, characterization data for the major regioisomer is given).

^{1}H NMR (500 MHz, $CDCl_3$, *1.2:1 mixture of rotamers*) δ 8.06 (d, *J* = 2.5 Hz, 0.5H, *minor rotamer*), 8.04 (d, *J* = 2.4 Hz, 0.5H, *major rotamer*), 7.37 (dd, *J* = 8.7, 2.5 Hz, 0.4H, *minor rotamer*), 7.31 (dd, *J* = 8.7, 2.6 Hz, 0.6H, *major rotamer*), 6.54 (d, *J* = 3.2 Hz, 0.5H, *major rotamer*), 6.52 (d, *J* = 3.1 Hz, 0.5H, *minor rotamer*), 5.11 (d, *J* = 8.9 Hz, 0.4H, *minor rotamer*), 5.01 (d, *J* = 8.9 Hz, 0.6H, *major rotamer*), 4.59 (d, *J* = 8.9 Hz, 0.6H, *minor rotamer*), 4.48 (d, *J* = 8.9 Hz, 0.4H, *major rotamer*), 3.77 (dt, *J* = 10.1, 4.9 Hz, 4H), 3.71 (s, 1.7H, *major rotamer*), 3.70 (s, 1.3H, *minor rotamer*), 3.48 – 3.33 (m, 4H), 2.58 – 2.44 (m, 1H), 2.27 – 2.14 (m, 1H), 2.09 – 2.01 (m, 1H), 1.32 (s, 4H, *minor rotamer*), 1.19 (s, 5H, *major rotamer*), 0.96 (s, 2.7H, *minor rotamer*), 0.94 (s, 3.3H, *major rotamer*), 0.89 (s, 2.7H, *minor rotamer*), 0.87 (s, 3.3H, *major rotamer*). **^{13}C NMR (126 MHz, $CDCl_3$, *mixture of rotamers*)** δ 173.7, 173.3, 159.3, 159.2, 154.1, 153.2, 146.8, 146.3, 136.7, 136.2, 129.3, 128.5, 106.7, 106.4, 83.6, 83.5, 80.2, 80.1, 66.8, 66.7, 61.0, 60.9, 60.5, 60.0, 52.2, 52.1, 46.3, 46.2, 30.9, 29.9, 28.4, 28.2, 24.8, 24.7, 24.6, 24.5 (carbon directly bonded to boron was not detected because of quadrupole relaxation).

NMR data matches that for **(*S*)-38**.

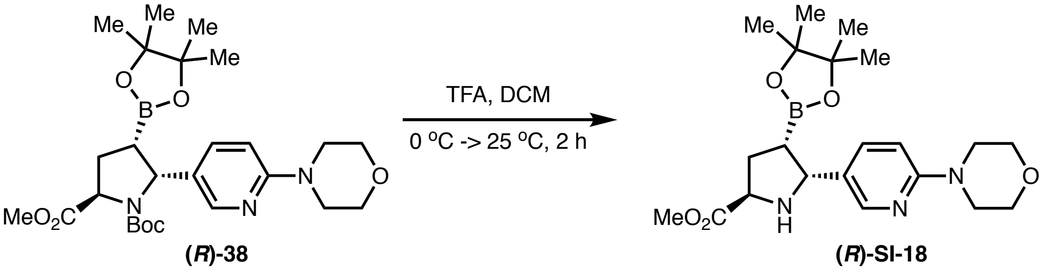

(*R*)-38 **(*R*)-SI-18**

methyl (2*R*,4*S*,5*R*)-5-(6-morpholinopyridin-3-yl)-4-(4,4,5,5-tetramethyl-1,3,2-dioxaborolan-2-yl)pyrrolidine-2-carboxylate ((*R*)-SI-18): The title compound was prepared according to the same procedure used to prepare **(*S*)-SI-18**. Obtained 4.0 mg **(*R*)-SI-18** as a white solid, 9%. The

enantiomeric purity was established via HPLC analysis using a chiral column. A racemic sample was obtained by preparing a 1:1 mixture of **(*S*)-SI-18** and **(*R*)-SI-18**.

^{1}H NMR (500 MHz, CDCl$_3$) δ 8.15 (d, *J* = 2.4 Hz, 1H), 7.53 (dd, *J* = 8.7, 2.5 Hz, 1H), 6.58 (d, *J* = 8.7 Hz, 1H), 4.65 (d, *J* = 8.7 Hz, 1H), 4.18 (dd, *J* = 8.5, 3.1 Hz, 1H), 3.80 (app. t, *J* = 4.9 Hz, 4H), 3.74 (s, 3H), 3.50 – 3.38 (m, 4H), 2.39 (ddd, *J* = 13.1, 10.5, 8.6 Hz, 1H), 2.19 (ddd, *J* = 13.2, 7.8, 3.1 Hz, 1H), 1.94 (dt, *J* = 10.4, 8.2 Hz, 1H), 0.98 (s, 6H), 0.96 (s, 6H). **^{13}C NMR (126 MHz, CDCl$_3$)** δ 176.0, 159.2, 146.7, 137.0, 129.7, 106.8, 83.4, 66.9, 61.0, 60.4, 52.4, 46.3, 31.9, 24.9, 24.7 (carbon directly bonded to boron was not detected because of quadrupole relaxation). **HPLC:** ChiralPak IA, 22 °C, 0.5 mL/min, 80:20 hexane:isopropanol, 254 nm, t_R = 25.412 min, t_s = 27.862 min, >99:1 er. See Supporting Information part 14 for HPLC chromatographs.

NMR data matches that for **(*S*)-SI-18**.

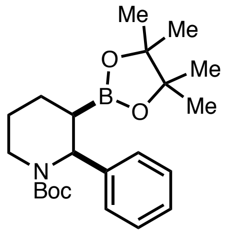

***tert*-butyl-2-phenyl-3-(4,4,5,5-tetramethyl-1,3,2-dioxaborolan-2-yl)piperidine-1-carboxylate (40):** The title compound was prepared according to General Procedure B. Crude yield was determined by ^{1}H NMR of the unpurified reaction mixture using mesitylene as an NMR standard. Purification by MPLC (gradient: 0-10% ethyl acetate in hexanes) yielded **40** as a white foam. Average yield over 2 runs: 60% NMR yield, >20:1 crude dr and > 10:1 crude rr (note: where crude rr could not be determined accurately due to rotamers, but a major regioisomer was clearly formed, rr was assigned as >10:1). 51% isolated yield, 99 mg (isolated as a single regioisomer).

^{1}H NMR (500 MHz, CDCl$_3$) δ 7.36 (d, *J* = 7.6 Hz, 2H), 7.28 (t, *J* = 7.6 Hz, 2H), 7.20 (t, *J* = 7.3 Hz, 1H), 5.91 – 5.32 (br m, 1H), 4.23 – 3.66 (br m, 1H), 2.96 – 2.50 (br m, 1H), 1.97 – 1.81 (m, 2H), 1.78 – 1.59 (m, 2H), 1.49 (s, 10H), 1.15 (s, 6H), 1.10 (s, 6H). **^{13}C NMR (126 MHz, VT 50 °C, CDCl$_3$)** δ 155.3, 141.7, 128.6, 128.0, 126.7, 83.4, 79.4, 55.1, 40.1, 28.7, 26.6, 24.9, 24.8, 21.4 (carbon directly bonded to boron was not detected because of quadrupole relaxation). **IR (neat):** 3060 (w), 2977 (w), 2933 (w), 2666 (w), 1684 (m), 1365 (m), 1320 (m), 1139 (s), 884 (m), 730 (s), 699 (m) cm^{-1}. **HRMS (ESI+):** Calculated for: $C_{22}H_{34}O_4NBNa$ $[M+Na]^+$: 410.2473, found: 410.2477.

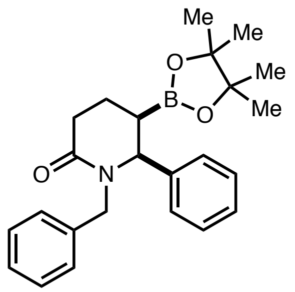

1-benzyl-6-phenyl-5-(4,4,5,5-tetramethyl-1,3,2-dioxaborolan-2-yl)piperidin-2-one (41): The title compound was prepared according to General Procedure B, but the reaction was run at 30 °C in an aluminum block and the crude mixture was washed 3 X with 1 M NaOAc instead of 1 M KOH. Crude yield was determined by ^{1}H NMR of the unpurified reaction mixture using mesitylene as an NMR standard. Purification by silica gel chromatography (40% ethyl acetate in hexanes) yielded **41** as a white solid. Average yield over 2 runs: 85% NMR yield, >20:1 crude dr and crude rr. 67% isolated yield, 131 mg.

^{1}H NMR (500 MHz, CDCl$_3$) δ 7.39 – 7.13 (m, 10H), 5.53 (d, *J* = 15.0 Hz, 1H), 4.70 (d, *J* = 5.2 Hz, 1H), 3.29 (d, *J* = 14.9 Hz, 1H), 2.75 (dd, *J* = 18.6, 5.2 Hz, 1H), 2.66 – 2.49 (m, 1H), 2.01 (qd, *J* = 13.4, 5.7 Hz, 1H), 1.89 – 1.69 (m, 2H), 1.12 (s, 6H), 1.00 (s, 6H). **^{13}C NMR (126 MHz, CDCl$_3$)** δ 170.6, 140.0, 137.6, 128.6, 128.4, 128.3, 128.1, 127.9, 127.3, 83.7, 60.9, 48.0, 32.8, 25.0, 24.7, 17.7 (signal of carbon directly bonded to boron was not detected because of quadrupolar relaxation). **IR (neat):** 2987 (w), 2835 (w), 1639 (s), 1381 (m), 1327 (m), 1139 (m), 698 (s) cm^{-1}. **HRMS (ESI+):** Calculated for: $C_{24}H_{31}O_3NB$ [M+H]$^+$: 392.2392, found: 392.2395.

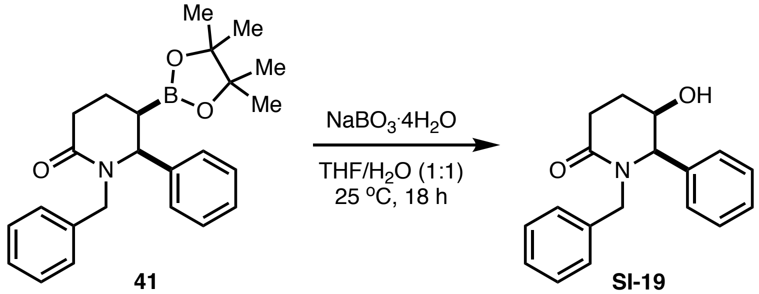

Regiochemistry of 1-benzyl-6-phenyl-5-(4,4,5,5-tetramethyl-1,3,2-dioxaborolan-2-yl)piperidin-2-one 41 was confirmed by oxidation to yield 1-benzyl-5-hydroxy-6-phenylpiperidin-2-one (SI-19): 1-benzyl-6-phenyl-5-(4,4,5,5-tetramethyl-1,3,2-dioxaborolan-2-yl)piperidin-2-one **41** was oxidized following General Procedure C on 0.1 mmol scale. After workup, the crude material was azeotroped with toluene (5 X 2 mL) to remove pinacol and yield 23 mg white solid **SI-19**, 82%.

^{1}H NMR (400 MHz, CDCl$_3$) δ 7.45 – 7.33 (m, 3H), 7.32 – 7.22 (m, 3H), 7.21 – 7.15 (m, 2H), 7.13 – 7.08 (m, 2H), 5.53 (d, *J* = 14.7 Hz, 1H), 4.47 (d, *J* = 5.2 Hz, 1H), 4.04 (dt, *J* = 8.4, 5.6 Hz, 1H), 3.34 (d, *J* = 14.7 Hz, 1H), 2.75 (dt, *J* = 18.1, 5.0 Hz, 1H), 2.58 (dt, *J* = 17.9, 8.6 Hz, 1H), 1.87 – 1.67 (m, 3H). **^{13}C NMR (101 MHz, CDCl$_3$)** δ 170.0, 137.0, 135.4, 128.9, 128.7, 128.7, 128.4, 127.6, 67.3, 63.8, 48.1, 29.6, 25.5 (note: missing 1 aromatic signal due to overlapping peaks). **IR (neat):** 3292 (br, m), 3059 (w), 2909 (w), 1616 (s), 1449 (m) 1260 (m), 955 (m), 702 (s) cm^{-1}. **HRMS (ESI+):** Calculated for: $C_{18}H_{20}O_2N$ [M+H]$^+$: 282.1489, found: 282.1490.

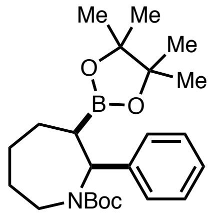

***tert*-butyl-2-phenyl-3-(4,4,5,5-tetramethyl-1,3,2-dioxaborolan-2-yl)azepane-1-carboxylate (42):** The title compound was prepared according to General Procedure B, but the reaction was run at 30 °C in an aluminum block. Crude yield was determined by ^{1}H NMR of the unpurified reaction mixture using mesitylene as an NMR standard. Purification by silica gel chromatography (5% ethyl acetate in hexanes) yielded **42** as a white foam. Average yield over 2 runs: 41% NMR yield, >20:1 crude dr, >10:1 crude rr (note: where crude rr could not be determined accurately due to rotamers, but a major regioisomer was clearly formed, rr was assigned as >10:1). 40% isolated yield, 80 mg (isolated as a single regioisomer).

^{1}H NMR (500 MHz, CDCl$_3$, *1:1 mixture of rotamers*) δ 7.32 – 7.07 (m, 5H), 5.65 (d, *J* = 6.7 Hz, 0.5H, *rotamer A*), 5.35 (d, *J* = 6.0 Hz, 0.5H, *rotamer B*), 3.86 (d, *J* = 14.6 Hz, 0.5H, *rotamer A*), 3.65 (d, *J* = 14.6 Hz, 0.5H, *rotamer B*), 3.54 (t, *J* = 12.4 Hz, 0.5H, *rotamer A*), 3.17 (t, *J* = 12.8 Hz, 0.5H, *rotamer B*), 2.10-1.95 (m, 1H), 1.95 – 1.52 (m, 6H), 1.49 (s, 5H, *rotamer A*), 1.29 (s, 4H, *rotamer B*), 1.08 (s, 3H, *rotamer A*), 1.06-0.98 (m, 9H, contains *rotamer B*). **^{13}C NMR (126 MHz, CDCl$_3$, *mixture of rotamers*)** δ 156.3, 143.7, 142.6, 128.1, 127.9, 127.0, 126.7, 126.2, 126.0, 83.3, 79.3, 79.2, 60.9, 58.8, 43.2, 42.8, 29.0, 28.7, 28.4, 28.3, 28.1, 27.8, 27.1, 26.2, 24.9, 24.8, 24.6, 24.5 (carbon directly bonded to boron was not detected because of quadrupole relaxation). **IR (neat):** 3027 (w), 2978 (w), 2924 (w), 1680 (s), 1449 (m), 1294 (m), 1217 (m), 1143 (s), 978 (m), 873 (m), 698 (m) cm^{-1}. **HRMS (ESI+):** Calculated for: $C_{23}H_{36}O_4NBNa$ [M+Na]$^+$: 424.2630, found: 424.2633.

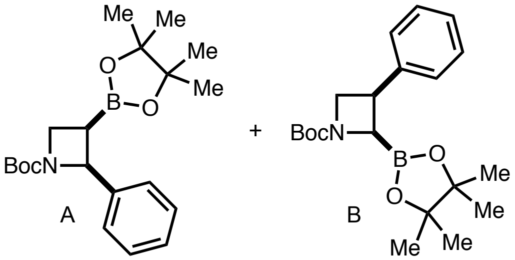

***tert*-butyl-2-phenyl-3-(4,4,5,5-tetramethyl-1,3,2-dioxaborolan-2-yl)azetidine-1-carboxylate (A) + *tert*-butyl-3-phenyl-2-(4,4,5,5-tetramethyl-1,3,2-dioxaborolan-2-yl)azetidine-1-carboxylate (B) (2):** The title compound was prepared according to General Procedure B with the following modifications: 1:1 THF/DMA (5 mL total) was used as solvent, 3 equiv. of PhBr was used, and the reactions were run at 30 °C. Crude yield was determined by ^{1}H NMR of the unpurified reaction mixture using mesitylene as an NMR standard. Purification by MPLC (gradient: 0-10% ethyl acetate in hexanes) yielded **2** as a colorless oil. Average yield over 2 runs: 40% NMR yield, >20:1 crude dr, 1:1 crude rr. 37% isolated yield, 66 mg (isolated as a 1:1 mixture of regioisomers, data for both regioisomers is given).

^{1}H NMR (500 MHz, CDCl$_3$, *1:1 mixture of regioisomers A/B*, overlapping peaks**)** δ 7.45 – 7.26 (m, 8H), 7.26 – 7.15 (m, 2H), 5.38 (br s, 1H), 4.46 – 4.20 (m, 3H), 4.13 (app. t, *J* = 7.3 Hz, 1H), 4.04 (dd, *J* = 9.7, 7.8 Hz, 1H), 3.90 (br s, 1H), 2.56 (td, *J* = 10.0, 6.7 Hz, 1H), 1.55 – 1.15 (m,

18H), 1.00 (s, 6H), 0.95 (s, 6H), 0.87 (br s, 6H), 0.84 (s, 6H). **^{13}C NMR (126 MHz, $CDCl_3$, *mixture of regioisomers A/B, each regioisomer has two rotameric peaks*)** δ 156.8, 156.1, 141.3, 140.7, 128.5, 128.2, 128.0, 127.3, 127.1, 126.3, 83.9, 83.6, 79.3, 65.8, 64.9, 55.8, 54.4, 53.9, 48.8, 47.6, 35.2, 34.7, 28.6, 28.4, 25.1, 24.9, 24.6, 24.5 (signal of carbon directly bonded to boron was not detected because of quadrupolar relaxation). **IR (neat):** 2977 (w), 2929 (w), 1698 (s), 1365 (s), 1128 (s), 856 (m), 694 (m) cm^{-1}. **HRMS (APCI+):** Calculated for: $C_{20}H_{30}O_4NBNa$ $[M+Na]^+$: 382.2160, found: 382.2160.

9. Gram-scale arylborations

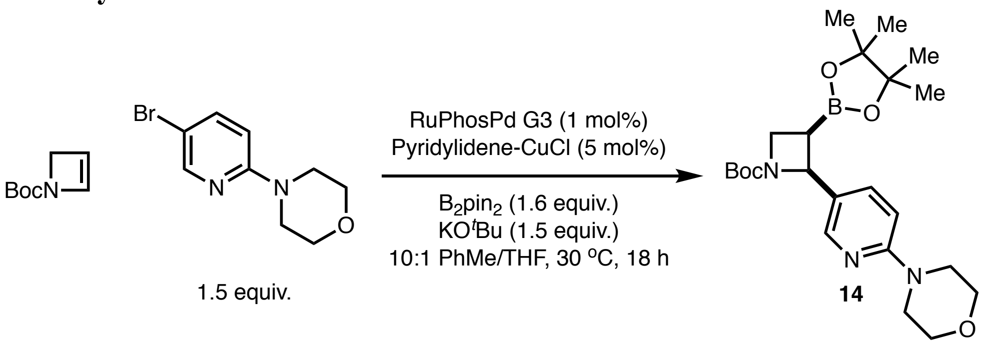

***tert*-butyl-2-(6-morpholinopyridin-3-yl)-3-(4,4,5,5-tetramethyl-1,3,2-dioxaborolan-2-yl)azetidine-1-carboxylate (14):** An oven-dried 100-mL round bottom flask equipped with a stir bar was brought into a N_2-filled glovebox and charged with RuPhosPd G3 (33.5 mg, 0.0400 mmol, 0.0100 equiv.), Pyridylidene-CuCl (107 mg, 0.200 mmol, 0.0500 equiv.), bis(pinacolato)diboron (1.63 g, 6.40 mmol, 1.60 equiv.), and KO^{t}Bu (673 mg, 6.00 mmol, 1.50 equiv.). The flask was sealed with a septum and removed from the glovebox. A separate oven-dried 50-mL round bottom flask was charged with 4-(5-bromopyridin-2-yl)morpholine (1.46 g, 6.00 mmol, 1.50 equiv.) and evacuated/backfilled with N_2 X 3. 50 mL 10:1 PhMe/THF was added to the 50-mL round bottom flask *via* syringe, followed by *tert*-butyl azete-1(2*H*)-carboxylate (684 µL, 4.00 mmol, 1.00 equiv.) *via* syringe. This solution was transferred to the 100-mL round bottom flask while stirring *via* syringe. The reaction was placed into a 30 °C water bath and stirred overnight. The reaction was quenched with H_2O (60 mL), and diluted with ethyl acetate (20 mL). The layers were separated and the aqueous layer was extracted with ethyl acetate (3 X 60 mL). The combined organic layers were dried over $MgSO_4$, gravity filtered, and concentrated. Purification *via* three sequential MPLC's (gradient: 0-40% ethyl acetate in hexanes) yielded *tert*-butyl-2-(6-morpholinopyridin-3-yl)-3-(4,4,5,5-tetramethyl-1,3,2-dioxaborolan-2-yl)azetidine-1-carboxylate **14** as an off-white solid, 1.19 g, 67%. Crystals suitable for X-ray crystallography were obtained *via* slow diffusion of pentane into a saturated solution of **14** in diethyl ether (see Supporting Information part 15 for crystallography data). Relative stereochemistry and regioselectivity for related products (**2-5**, **12-17**) were assigned by analogy to the crystal structure of **14**.

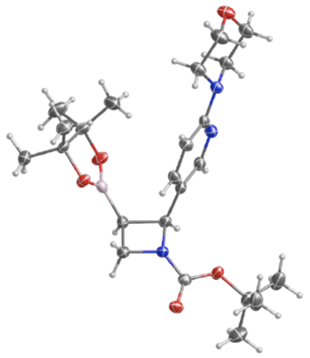

^{1}H NMR (500 MHz, $CDCl_3$) δ 8.16 (s, 1H), 7.53 (dd, J = 8.4, 2.4 Hz, 1H), 6.61 (d, J = 8.7 Hz, 1H), 5.30 (br s, 1H), 4.09 (dd, J = 7.9, 6.7 Hz, 1H), 4.02 (dd, J = 9.7, 7.9 Hz, 1H), 3.81 (app. t, J = 4.8 Hz, 4H), 3.44 (app. t, J = 9.8 Hz, 4H), 2.53 (td, J = 9.9, 6.7 Hz, 1H), 1.52 – 1.17 (br m, 9H), 1.01 (s, 6H), 0.90 (s, 6H). **^{13}C NMR (126 MHz, VT 50 °C, $CDCl_3$)** δ 159.6, 156.1, 146.5, 136.1,

127.0, 106.5, 83.8, 79.5, 66.9, 63.3, 46.5, 45.9, 28.6, 25.1, 24.7 (signal of carbon directly bonded to boron was not detected because of quadrupolar relaxation). **IR (neat):** 2974 (w), 2856 (w), 2363 (w), 2162 (w), 1977 (w), 1682 (m), 1364 (s), 1120 (s) cm^{-1}. **HRMS (ESI+):** Calculated for: $C_{23}H_{37}O_5N_3B$ $[M+H]^+$: 446.2825, found: 446.2825.

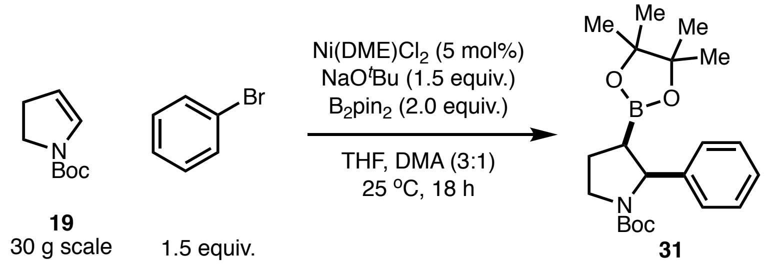

***tert*-butyl-2-phenyl-3-(4,4,5,5-tetramethyl-1,3,2-dioxaborolan-2-yl)pyrrolidine-1-carboxylate (31):** In a 2L three-neck flask equipped with a magnetic stirrer under Ar atmosphere (no glovebox) was added NaO*t*Bu (25.56 g, 0.2660 mol, 1.500 eq.) and B_2Pin_2 (90.00 g, 0.3546 mol, 2.000 eq.). Under vigorous stirring, dry THF (1.200 L) was added. The reaction mixture was allowed to stir under Ar atmosphere for 30 min at room temperature. After that PhBr (41.75 g, 0.2660 mol, 1.500 eq.) and *tert*-butyl 2,3-dihydro-1*H*-pyrrole-1-carboxylate (**19**) (30.000 g, 0.177 mol, 1.000 eq.) were added consequently. The reaction mixture was quickly vacuumed and then re-filled with Ar and allowed to cool down to 0 °C in an external ice bath under stirring.

$Ni(DME)Cl_2$ (1.950 g, 8.860 mmol, 0.05000 eq.) was added to dry DMA (400.0 mL). This mixture was bubbled with a stream of Ar for 5 min, and was subsequently added to the main reaction mixture. The combined reaction mixture was left to stir overnight at a room temperature.

Isolation: MeO*t*Bu (1L) and 1M HCl (100 mL) were added to the reaction mixture. The reaction mixture was transferred to a separating funnel. The mixture was vigorously shacked, and the lower aqueous layer was separated and discarded. The organic layer was washed with water (3 x 500 mL), brine (1 x 500 mL); dried over Na_2SO_4 and evaporated.

Purification: The obtained crude product was dissolved in hexane/EtOAc=6/1 (100 mL) and filtered through a plug of silica gel (100 g) on a glass Schott filter (diameter 7.0 cm, high 8.5 cm) using hexane/EtOAc=6/1 (1.5-2L) as an eluent until the constant mass of the material obtained after evaporation of the organic phase. The obtained solution (R_f=0.4 in hexane/EtOAc=6/1) was evaporated under vacuum. White solid (32 g) with ca. 90% purity according to NMR.

To the obtained material was added pentane (250 mL). The formed suspension was heated to a reflux under stirring. Afterwards, the suspension was left to cool down to room temperature for 1 h. The white precipitate was filtered off, and was air dried. White solid **31** was obtained (25.1 g, 0.067 mol, 38% yield; single stereoisomer).

^{1}H NMR (500 MHz, $CDCl_3$, *7:3 mixture of rotamers*) δ 7.25 – 7.11 (m, 5H), 5.04 (d, *J* = 8.3 Hz, 0.3H, *minor rotamer*), 4.92 (d, *J* = 8.6 Hz, 0.7H, *major rotamer*), 3.83 (t, *J* = 9.4 Hz, 0.7H, *major rotamer*), 3.72 (t, *J* = 9.5 Hz, 0.3H, *minor rotamer*), 3.47 – 3.34 (m, 1H), 2.29 – 2.15 (m, 1H), 2.06 – 1.91 (m, 2H), 1.41 (s, 3H, *minor rotamer*), 1.14 (s, 6H, *major rotamer*), 0.99-0.97 (m, 6H, *rotamers*), 0.87-0.86 (m, 6H, *rotamers*). **^{13}C NMR (151 MHz, $CDCl_3$, *mixture of rotamers*)** δ 154.4, 154.2, 144.3, 143.7, 128.1, 127.8, 127.1, 126.9, 126.8, 126.7, 83.4, 79.2, 79.1, 63.2, 62.9, 47.9, 47.4, 30.5 (br s), 28.7, 28.3, 24.9, 24.8, 24.7, 24.6 (carbon directly bonded to boron was not

detected because of quadrupole relaxation). **HRMS (ESI-TOF):** Calculated for: $C_{21}H_{33}BNO_4$ [M + H]$^+$: 374.2503; found 374.2491. **M.P.** = 86-87 °C.

NMR data matched that from compound **31** in the substrate scope.

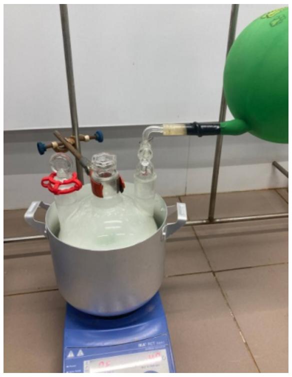

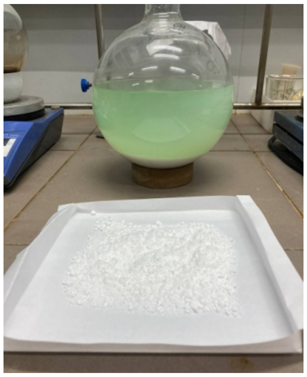

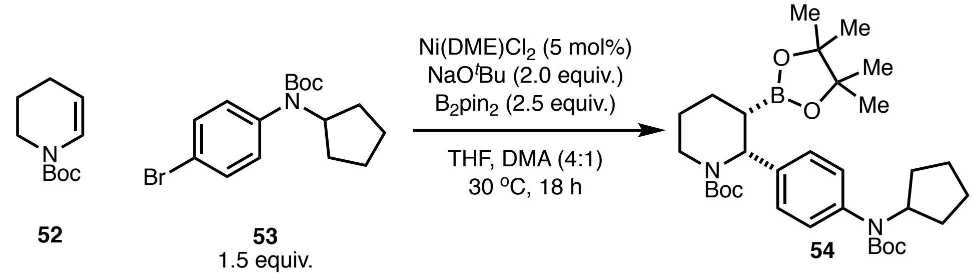

***tert*-butyl-2-(4-((*tert*-butoxycarbonyl)(cyclopentyl)amino)phenyl)-3-(4,4,5,5-tetramethyl-1,3,2-dioxaborolan-2-yl)piperidine-1-carboxylate (54):** An oven dried 100-mL round bottom flask equipped with a stir bar and an oven dried 25-mL round bottom flask were brought into a N_2-filled glovebox. The 100-mL flask was charged with bis(pinacolato)diboron (3.17 g, 12.5 mmol, 2.50 equiv), *tert*-butyl (4-bromophenyl)(cyclopentyl)carbamate **53** (2.55 g, 7.50 mmol, 1.50 equiv.), and NaO^{t}Bu (960 mg, 10.0 mmol, 2.00 equiv). The 25-mL flask was charged with Ni(DME)Cl_2 (55.0 mg, 0.25 mmol, 0.0500 equiv). Both flasks were sealed with septa, removed from the glovebox, and placed under N_2 atmosphere. THF (40.0 mL) was added to the 100-mL flask, followed by *tert*-butyl 3,4-dihydropyridine-1(2H)-carboxylate **52** (916 mg, 927 µL, 5.00 mmol, 1.00 equiv.) sequentially *via* syringes. The reaction was then cooled to 0 ºC in an ice-water bath for 10 minutes. The 25-mL round bottom flask was charged with 10.0 mL of DMA to prepare the catalyst solution (0.025 M Ni(DME)Cl_2 in DMA), and then 10.0 mL of this catalyst solution was added to the reaction vial (5 mol% catalyst loading) *via* syringe. The reaction was warmed to room temperature and then stirred at 30º C for 18 h. The reaction was quenched upon the addition of H_2O (50 mL) and the mixture was extracted with EtOAc (3 X 40 mL). The combined organic layers were diluted with hexanes (100 mL) and then washed with 1 M KOH (3 X 150 mL). The organic layer was dried over $MgSO_4$, gravity filtered, and concentrated *in vacuo*. Purification *via* silica gel chromatography (10% ethyl acetate in hexanes), followed by a second purification by MPLC (gradient: 0-10% ethyl acetate in hexanes) yielded **54** as a white solid (1.29 g, 45%).

^{1}H NMR (500 MHz, $CDCl_3$) δ 7.29 (d, J = 8.0 Hz, 2H), 6.95 (d, J = 8.4 Hz, 2H), 5.86 – 5.30 (br m, 1H), 4.45 (p, J = 8.5 Hz, 1H), 4.08-3.67 (br m, 1H), 2.94-2.49 (br m, 1H), 1.93-1.78 (m, 4H), 1.74-1.55 (m, 2H), 1.53 – 1.41 (m, 14H), 1.33 (s, 11H), 1.13 (s, 6H), 1.07 (s, 6H). **^{13}C NMR (126**

MHz, VT 50 °C, $CDCl_3$) δ 155.3, 155.2, 140.3, 138.9, 129.3, 128.8, 83.4, 79.6, 79.5, 59.3, 40.1, 30.3, 30.2, 28.7, 28.6, 26.6, 24.9, 24.8, 23.3, 21.3 (signal of carbon directly bonded to boron was not detected because of quadrupolar relaxation). **IR (neat):** 3035 (w), 2975 (w), 2873 (w), 1687 (s), 1399 (m), 1366 (m), 1336 (m), 1309 (m), 1165 (m), 1141 (m), 1111 (m), 850 (m) cm^{-1}. **HRMS (ESI+):** Calculated for: $C_{32}H_{51}O_6N_2BNa$ $[M+Na]^+$: 593.3732, found: 593.3745.

10. Further functionalizations

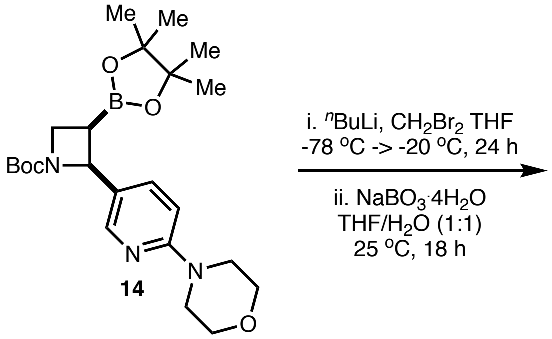

***tert*-butyl-3-(hydroxymethyl)-2-(6-morpholinopyridin-3-yl)azetidine-1-carboxylate (47)** was prepared according to the following literature procedure[25]: An oven-dried 13X100 mm reaction vial equipped with a stir bar was charged with *tert*-butyl-2-(6-morpholinopyridin-3-yl)-3-(4,4,5,5-tetramethyl-1,3,2-dioxaborolan-2-yl)azetidine-1-carboxylate **14** (44.5 mg, 0.100 mmol, 1.00 equiv.) and evacuated/backfilled with N_2 X 3. THF (1.00 mL) was added followed by dibromomethane (22.3 μL, 0.320 mmol, 3.20 equiv.) sequentially *via* syringes. The mixture was cooled to -78 °C in a dry ice/acetone bath. *n*-Butyllithium (110 μL, 0.220 mmol, 2.20 equiv., 2.00 M in hexanes) was added dropwise *via* syringe, and then the reaction was stirred at -78 °C for 1 h. The reaction was then warmed to -20 °C in a freezer and left to sit without stirring for 23 hours. The reaction was quenched with H_2O (3 mL) and extracted with ethyl acetate (3 X 3 mL). The combined organic layers were dried over $MgSO_4$, gravity filtered, and concentrated *in vacuo*. The crude mixture was oxidized according to General Procedure C on 0.1 mmol scale. Purification *via* MPLC (gradient: 0-100% ethyl acetate in hexanes) yielded 10.5 mg **47** as a white foam, 30% (2 steps).

^{1}H NMR (500 MHz, $CDCl_3$) δ 8.12 (s, 1H), 7.48 (dd, *J* = 8.7, 2.4 Hz, 1H), 6.64 (d, *J* = 8.8 Hz, 1H), 5.30 (d, *J* = 8.5 Hz, 1H), 4.07 (app. t, *J* = 8.6 Hz, 1H), 3.82 (app. t, *J* = 4.7 Hz, 4H), 3.64 (dd, *J* = 8.7, 5.0 Hz, 1H), 3.57 – 3.44 (m, 6H), 2.96 (pd, *J* = 8.0, 5.2 Hz, 1H), 1.35 (br s, 9H). **^{13}C NMR (126 MHz, $CDCl_3$)** δ 159.1, 156.6, 146.2, 136.3, 122.9, 106.6, 80.0, 66.9, 63.2, 62.3, 49.7, 45.8, 35.8, 28.4. **IR (neat):** 3306 (br, w), 2976 (w), 1678 (m), 1607 (m), 1365 (m), 1120 (s), 941 (m) cm^{-1}. **HRMS (ESI+):** Calculated for: $C_{18}H_{28}O_4N_3$ $[M+H]^+$: 350.2074, found: 350.2077.

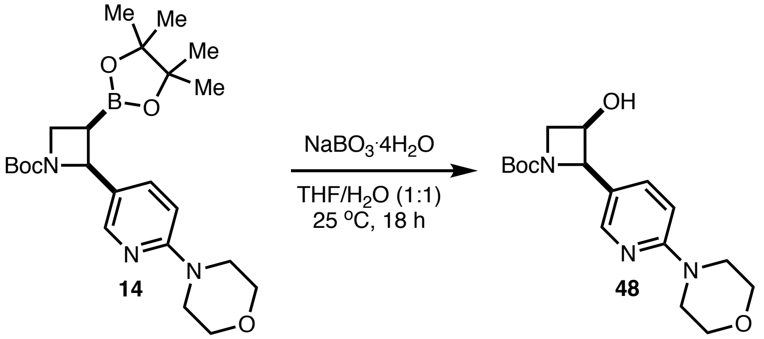

***tert*-butyl-3-hydroxy-2-(6-morpholinopyridin-3-yl)azetidine-1-carboxylate (48)** was prepared according to General Procedure C on 0.1 mmol scale. Purification *via* silica gel chromatography (40% acetone in hexanes) yielded 25 mg white solid **48**, 75%.

^{1}H NMR (500 MHz, $CDCl_3$) δ 8.04 (s, 1H), 7.52 (dd, J = 8.8, 2.4 Hz, 1H), 6.65 (d, J = 8.8 Hz, 1H), 5.20 (d, J = 6.9 Hz, 1H), 4.66 (td, J = 6.9, 4.1 Hz, 1H), 4.23 (dd, J = 9.8, 6.9 Hz, 1H), 3.78 (app. t, J = 4.8 Hz, 5H), 3.46 (app. t, J = 4.9 Hz, 4H), 2.55 (br s, 1H), 1.33 (s, 9H). **^{13}C NMR (126 MHz, $CDCl_3$)** δ 159.5, 156.1, 147.2, 137.5, 120.8, 106.7, 80.0, 68.1, 66.8, 64.1, 57.2, 45.8, 28.4.

NMR data matches that for the same compound **48** prepared in the substrate scope.

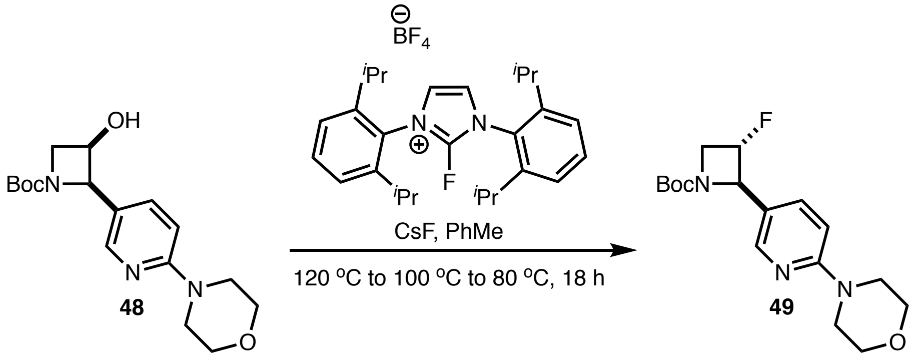

***tert*-butyl-3-fluoro-2-(6-morpholinopyridin-3-yl)azetidine-1-carboxylate (49)** was prepared according to literature procedure[26]: An oven-dried 13X100 mm reaction vial equipped with a stir bar was brought into an N_2-filled glovebox and charged with CsF (76.0 mg, 0.500 mmol, 5.00 equiv.) and Alkylfluor (59.3 mg, 0.120 mmol, 1.20 equiv.). The vial was sealed with a septum and removed from the glovebox. The solids were heated to 120 °C under vacuum with stirring for 1 h. The vial was then backfilled with N_2, and toluene (0.750 mL) was added via syringe. The mixture was then heated to 100 °C with stirring for 1.5 hours. A separate oven-dried 2-dram vial was charged with *tert*-butyl-3-hydroxy-2-(6-morpholinopyridin-3-yl)azetidine-1-carboxylate **48** (33.5 mg, 0.100 mmol, 1.00 equiv.) and evacuated/backfilled with N_2 X 3. **48** was dissolved in toluene (2.00 mL) and then transferred to the reaction vial *via* syringe. The vial was quickly capped and the reaction was stirred at 80 °C overnight. The reaction was cooled to room temperature and filtered through a celite plug, washing with DCM. The eluent was concentrated. Purification *via* silica gel chromatography (40% ethyl acetate in hexanes) yielded 15 mg **49** as a yellow oil, 44%.

^{1}H NMR (400 MHz, $CDCl_3$) δ 8.15 (s, 1H), 7.46 (dd, J = 8.8, 2.5 Hz, 1H), 6.64 (d, J = 8.8 Hz, 1H), 5.10 (dd, J = 23.0, 3.0 Hz, 1H), 4.94 (dq, J = 56.1, 4.4 Hz, 1H), 4.19 (ddd, J = 17.4, 10.1, 6.2 Hz, 1H), 4.03 (ddd, J = 24.5, 10.1, 3.9 Hz, 1H), 3.81 (app. t, J = 4.8 Hz, 4H), 3.51 (app. t, J = 4.9

Hz, 4H), 1.36 (s, 9H). **^{13}C NMR (101 MHz, $CDCl_3$)** δ 159.7, 157.0, 146.5, 135.9, 123.5, 106.9, 89.5, 87.4, 80.7, 66.9, 54.4 (d, J = 21.9 Hz), 45.8, 28.5. **^{19}F NMR (376 MHz, $CDCl_3$)** δ -177.81 (dtd, J = 56.1, 23.7, 17.8 Hz). **IR (neat):** 2969 (w), 2849 (w), 2364 (w), 2155 (w), 2033 (w), 1699 (s), 1607 (m), 1496 (m), 1366 (s), 1245 (s), 1117 (s), 942 (m) cm^{-1}. **HRMS (ESI+):** Calculated for: $C_{17}H_{25}O_3N_3F$ $[M+H]^+$: 338.1874, found: 338.1877.

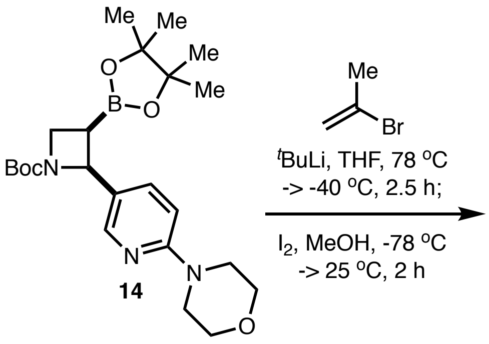

***tert*-butyl-2-(6-morpholinopyridin-3-yl)-3-(prop-1-en-2-yl)azetidine-1-carboxylate (50)** was prepared according to the following literature procedure[27]: An oven-dried round bottom flask equipped with a stir bar under N_2 was charged with 2-bromoprop-1-ene (73.4 µL, 0.820 mmol, 4.10 equiv.) and THF (2.00 mL) sequentially *via* syringes. The mixture was cooled to -78 °C in a dry ice/acetone bath. *tert*-Butyllithium (656 µL, 1.22 molar in pentane, 0.800 mmol, 4.00 equiv.) was added dropwise *via* syringe and the reaction was stirred at -78 °C for 30 minutes. A solution of *tert*-butyl-2-(6-morpholinopyridin-3-yl)-3-(4,4,5,5-tetramethyl-1,3,2-dioxaborolan-2-yl)azetidine-1-carboxylate **14** (89.1 mg, 0.200 mmol, 1.00 equiv.) dissolved in THF (0.800 mL) was added to the reaction dropwise *via* syringe at -78 °C. The reaction was stirred at -78 °C for 1 h, then warmed to -40 °C in a dry ice/MeCN bath and stirred for 1 h. The reaction was then cooled to -78 °C in a dry ice/acetone bath and a solution of I_2 (203 mg, 0.800 mmol, 4.00 equiv.) in MeOH (3.20 mL) was added dropwise *via* syringe. The reaction was stirred at -78 °C for 45 min, then warmed to room temperature and stirred for 1 h. The reaction was quenched with sat. aq. $Na_2S_2O_3$ (4 mL) and stirred for 1 h. The reaction was diluted with H_2O (2 mL) and the aqueous layer was extracted with ethyl acetate (3 X 6 mL). The combined organic layers were washed with brine (1 X 20 mL), dried over $MgSO_4$, gravity filtered, and concentrated *in vacuo.* Purification *via* MPLC (gradient: 0-35% ethyl acetate in hexanes) yielded 51 mg *tert*-butyl-2-(6-morpholinopyridin-3-yl)-3-(prop-1-en-2-yl)azetidine-1-carboxylate **50** as a white solid, 71%.

^{1}H NMR (500 MHz, $CDCl_3$) δ 8.04 (s, 1H), 7.41 (dd, J = 8.7, 2.5 Hz, 1H), 6.55 (d, J = 8.8 Hz, 1H), 5.26 (d, J = 9.0 Hz, 1H), 4.88 (s, 1H), 4.85 (s, 1H), 4.10 (dd, J = 8.9, 6.5 Hz, 1H), 4.04 (app. t, J = 8.8 Hz, 1H), 3.80 (app. t, J = 4.9 Hz, 4H), 3.53 – 3.41 (m, 5H), 1.48 – 1.20 (m, 12H). **^{13}C NMR (126 MHz, $CDCl_3$, *mixture of rotamers*)** δ 159.2, 156.1, 147.0, 141.4, 136.6, 123.8, 113.3, 106.1, 82.9, 79.7, 66.8, 65.1, 49.4, 45.8, 41.1, 28.4, 24.7, 22.2. **IR (neat):** 2973 (w), 2846 (w), 1703 (s), 1605 (m), 1411 (s), 1244 (s), 1120 (s), 944 (m) cm^{-1}. **HRMS (APCI+):** Calculated for: $C_{20}H_{30}O_3N_3$ $[M+H]^+$: 360.2282, found: 360.2285.

11. Synthetic Applications

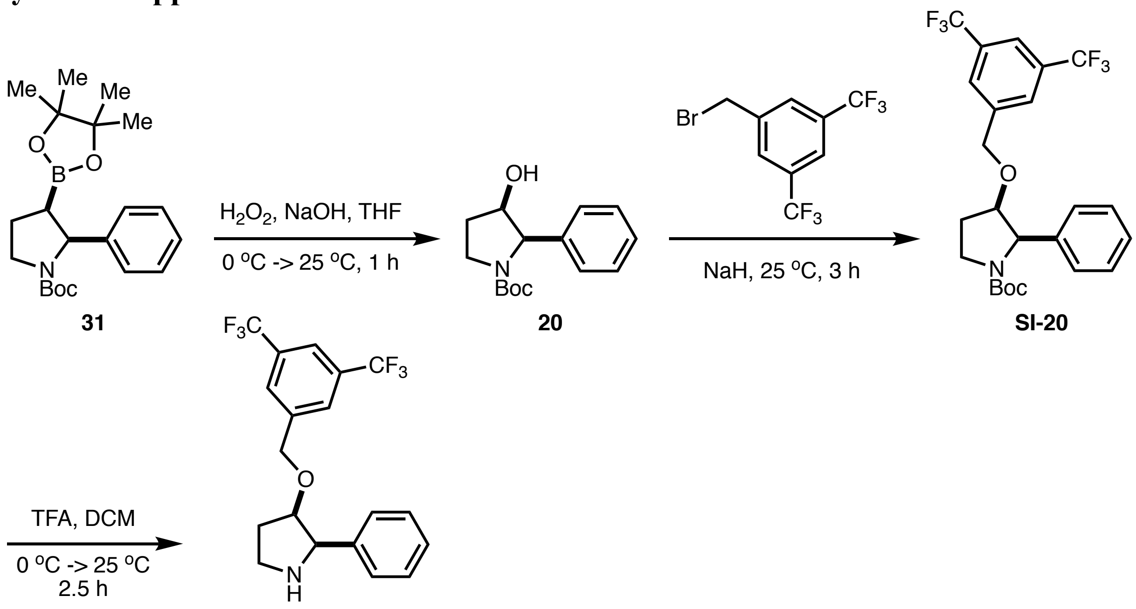

31 **20** **SI-20** **51**

***tert*-butyl-3-hydroxy-2-phenylpyrrolidine-1-carboxylate (20)** was synthesized according to the following literature procedure[31]: A round bottom flask equipped with a stir bar was charged with *tert*-butyl-2-phenyl-3-(4,4,5,5-tetramethyl-1,3,2-dioxaborolan-2-yl)pyrrolidine-1-carboxylate **31** (119 mg, 0.319 mmol, 1.00 equiv.) and THF (3.20 mL) *via* syringe. The mixture was cooled to 0 °C in an ice water bath. Sodium hydroxide (1.59 mL, 2.00 molar, 3.19 mmol, 10.0 equiv.) and hydrogen peroxide (98.0 µL, 30 wt%, 0.956 mmol, 3.00 equiv.) were added sequentially *via* syringe. The reaction was warmed to 25 °C and stirred for 1 hour. The reaction was slowly quenched with sat. aq. $Na_2S_2O_3$ (3 mL), the layers were separated, and the aqueous layer was extracted with ethyl acetate (3 X 3 mL). The combined organic layers were dried over $MgSO_4$, gravity filtered, and concentrated *in vacuo*. Purification *via* MPLC (gradient: 0-30% ethyl acetate in hexanes) yielded *tert*-butyl-3-hydroxy-2-phenylpyrrolidine-1-carboxylate **20** as a white solid (69 mg, 83%). Crystals suitable for X-ray crystallography were obtained *via* slow diffusion of pentane into a saturated solution of **20** in diethyl ether (see Supporting Information part 15 for crystallography data). Relative stereochemistry and regioselectivity for related Bpin products (**21**-**33**, **37**) were assigned by analogy to the crystal structure of **20**.

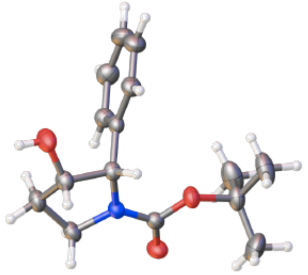

^{1}H NMR (500 MHz, $CDCl_3$, *2.4:1 mixture of rotamers*) δ 7.35 (t, J = 7.4 Hz, 2H), 7.28 (t, J = 7.4 Hz, 1H), 7.20 (d, J = 7.5 Hz, 2H), 4.94 (s, 0.3H, *minor rotamer*), 4.82 (d, J = 5.7 Hz, 0.7H, *major rotamer*), 4.41 (q, J = 5.5 Hz, 1H), 3.83 – 3.56 (br m, 2H), 2.10 (td, J = 12.9, 7.6 Hz, 1H), 1.94 (br s, 1H), 1.44 (s, 3H, *minor rotamer*), 1.28 – 1.03 (m, 7H, contains *major rotamer*). **^{13}C NMR (126 MHz, $CDCl_3$, *mixture of rotamers*)** δ 154.7, 138.4, 128.8, 128.6, 127.8, 127.4, 127.2, 79.7, 79.6, 73.4, 72.7, 65.9, 65.0, 45.1, 44.7, 31.9, 28.6, 28.2. **IR (neat):** 3339 (m, br), 2983 (w), 2880 (w), 1651 (s), 1417 (s), 1166 (m), 1118 (s), 700 (s) cm^{-1}. **HRMS (ESI+):** Calculated for: $C_{15}H_{21}O_3NNa$ [M+Na]$^+$: 286.1414, found: 286.1414.

***tert*-butyl-3-((3,5-bis(trifluoromethyl)benzyl)oxy)-2-phenylpyrrolidine-1-carboxylate (SI-20)** was synthesized according to the following modified literature procedure[28]: A flame-dried reaction via equipped with a stir bar was charged with *tert*-butyl-3-hydroxy-2-phenylpyrrolidine-1-carboxylate **20** (26.3 mg, 0.100 mmol, 1.00 equiv.) and placed under N_2 atmosphere by evacuating/backfilling with N_2 X 3. DMF (0.500 mL) was added *via* syringe, followed by sodium hydride (8.00 mg, 60 wt%, 0.200 mmol, 2.00 equiv.) in a single portion. The mixture was stirred for 10 minutes and then 1-(bromomethyl)-3,5-bis(trifluoromethyl)benzene (28.0 μL, 0.150 mmol, 1.50 equiv.) was added *via* syringe. The reaction was stirred for 4 hours, and then quenched with 1 M HCl (0.5 mL) and diluted with diethyl ether (0.5 mL). The aqueous layer was extracted with diethyl ether (2 X 0.5 mL), the combined organic layers were dried over $MgSO_4$, gravity filtered, and concentrated *in vacuo*. Purification *via* MPLC (gradient: 0-20% ethyl acetate in hexanes) yielded *tert*-butyl-3-((3,5-bis(trifluoromethyl)benzyl)oxy)-2-phenylpyrrolidine-1-carboxylate **SI-20** as a white solid (32 mg, 64%).

^{1}H NMR (400 MHz, $CDCl_3$, *2.3:1 mixture of rotamers*) δ 6.96 – 6.75 (m, 8H), 4.64 (s, 0.3H, *minor rotamer*), 4.50 (s, 0.7H, *major rotamer*), 4.03 (d, *J* = 12.4 Hz, 1H), 3.93 – 3.77 (m, 2H), 3.39 – 3.10 (br m, 2H), 1.70 (qd, *J* = 7.3, 2.2 Hz, 2H), 0.99 (br s, 2.7H, *minor rotamer*), 0.71 (br s, 6.3H, *major rotamer*). **^{13}C NMR (126 MHz, $CDCl_3$, *mixture of rotamers*)** δ 154.6, 140.8, 139.5, 131.5 (q, *J* = 33.2 Hz), 128.0, 127.5, 127.4, 127.1, 124.5, 122.3, 121.4 (app. p, *J* = 3.9 Hz), 120.1, 81.1, 79.8, 70.2, 64.6, 44.46, 29.6, 28.2. **^{19}F NMR (376 MHz, $CDCl_3$)** δ -62.89. **IR (neat):** 2979 (w), 1693 (m), 1392 (m), 1275 (m), 1165 (m), 1125 (s), 703 (m) cm^{-1}. **HRMS (ESI+):** Calculated for: $C_{24}H_{25}O_3NF_6Na$ $[M+Na]^+$: 512.1631, found: 512.1631.

3-((3,5-bis(trifluoromethyl)benzyl)oxy)-2-phenylpyrrolidine (51) was synthesized according to the following literature procedure[29]: An oven-dried reaction vial equipped with a stir bar was charged with *tert*-butyl-3-((3,5-bis(trifluoromethyl)benzyl)oxy)-2-phenylpyrrolidine-1-carboxylate **SI-20** (10.6 mg, 0.022 mmol, 1.00 equiv.) and evacuated/backfilled with N_2 X 3. DCM (0.200 mL) was added *via* syringe and the mixture was cooled to 0 °C in an ice water bath. Trifluoroacetic acid (33.4 μL, 0.433 mmol, 20 equiv.) was added dropwise *via* syringe. The reaction was warmed to 25 °C and stirred for 2.5 hours (until complete by TLC). The reaction was concentrated *in vacuo*, and the crude residue was diluted with ethyl acetate (1 mL). The organic layer was washed with sat. aq. $NaHCO_3$ (1 X 1 mL), dried over $MgSO_4$, gravity filtered, and concentrated *in vacuo* to yield 3-((3,5-bis(trifluoromethyl)benzyl)oxy)-2-phenylpyrrolidine **51** as a yellow oil (7.8 mg, 93%). Characterization data is not given in the literature report[30] of this compound, so full characterization data is given here.

^{1}H NMR (500 MHz, $CDCl_3$) δ 7.07 – 6.81 (m, 8H), 3.99 (d, *J* = 12.6 Hz, 1H), 3.76 – 3.62 (m, 3H), 2.95 (ddd, *J* = 9.8, 8.3, 5.4 Hz, 1H), 2.68 – 2.55 (m, 1H), 1.87 (br s, 1H), 1.81 – 1.72 (m, 1H), 1.73 – 1.64 (m, 1H). **^{13}C NMR (126 MHz, $CDCl_3$)** δ 141.2, 138.4, 131.5 (q, *J* = 33.5 Hz), 128.3, 128.0, 127.6, 127.3, 123.4 (q, *J* = 272.1 Hz), 121.3 (q, *J* = 4.0 Hz), 82.2, 70.0, 67.6, 44.8, 32.6. **^{19}F NMR (376 MHz, $CDCl_3$)** δ -62.88. **IR (neat):** 2933 (w), 2026 (w), 1374 (w), 1275 (s), 1168 (m), 1124 (s), 886 (w), 701 (m), 681 (m) cm^{-1}. **HRMS (ESI+):** Calculated for: $C_{19}H_{18}ONF_6$ $[M+H]^+$: 390.1287, found: 390.1288.

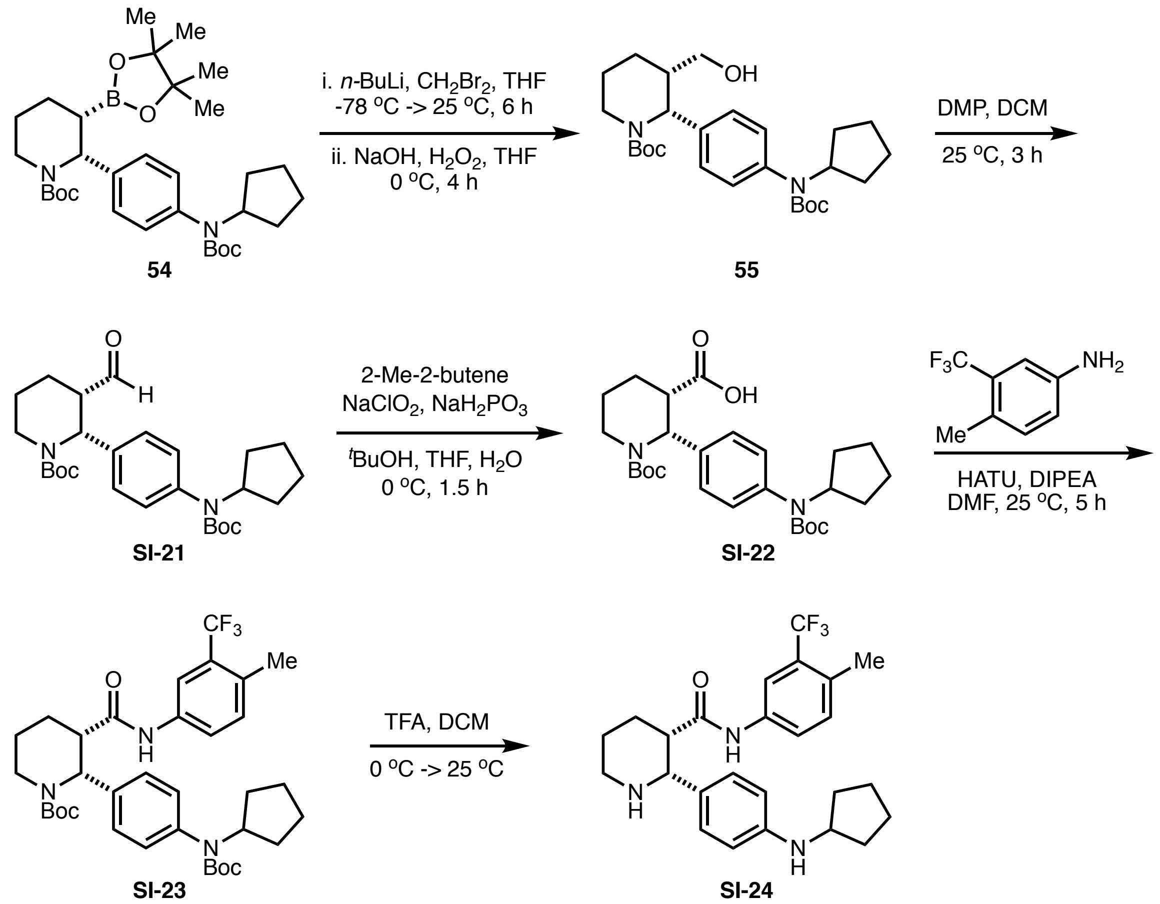

***tert*-butyl-2-(4-((*tert*-butoxycarbonyl)(cyclopentyl)amino)phenyl)-3-(hydroxymethyl)piperidine-1-carboxylate (55)** was synthesized according to the following literature procedures[1,31]: An oven dried round bottom flask equipped with a stir bar was charged with *tert*-butyl-2-(4-((*tert*-butoxycarbonyl)(cyclopentyl)amino)phenyl)-3-(4,4,5,5-tetramethyl-1,3,2-dioxaborolan-2-yl)piperidine-1-carboxylate **54** (850 mg, 1.49 mmol, 1.00 equiv.) and evacuated/backfilled with N_2 X 3. THF (15.0 mL) was added, followed by dibromomethane (647 mg, 260 µL, 3.72 mmol, 2.50 equiv.) sequentially *via* syringes. The reaction was cooled to -78 °C in a dry ice/acetone bath. *n*-Butyllithium (1.62 mL, 2.02 molar, 3.28 mmol, 2.20 equiv.) was added dropwise *via* syringe. The reaction was stirred at -78 °C for 1 h and then warmed to 25 °C and stirred for 5 h. The reaction was quenched with H_2O (20 mL), the layers were separated and the aqueous layer was extracted with ethyl acetate (3 X 20 mL). The combined organic layers were dried over $MgSO_4$, gravity filtered, and concentrated *in vacuo*. The crude *tert*-butyl-2-(4-((*tert*-butoxycarbonyl)(cyclopentyl)amino)phenyl)-3-((4,4,5,5-tetramethyl-1,3,2-dioxaborolan-2-yl)methyl)piperidine-1-carboxylate was oxidized directly.

tert-butyl-2-(4-((tert-butoxycarbonyl)(cyclopentyl)amino)phenyl)-3-((4,4,5,5-tetramethyl-1,3,2-dioxaborolan-2-yl)methyl)piperidine-1-carboxylate (871 mg, 1.49 mmol, 1.00 equiv.) was dissolved in THF (9.00 mL) in a round bottom flask equipped with a stir bar and the mixture was cooled to 0 °C in an ice water bath. Sodium hydroxide (9.00 mL, 2.00 molar, 20.0 mmol, 10.0 equiv.) was added *via* syringe, followed by hydrogen peroxide (9.00 mL, 30.0% wt, 90.0 mmol, 60.0 equiv.) *via* syringe. The reaction was stirred at 0 °C for 4 h, then carefully quenched with sat. aqueous $Na_2S_2O_3$ at 0 °C. The aqueous layer was extracted with ethyl acetate (3 X 30 mL), the combined organic layers were dried over MgSO4, gravity filtered, and

concentrated *in vacuo*. Purification by MPLC (gradient: 0-30% ethyl acetate in hexanes) yielded 382 mg *tert*-butyl-2-(4-((*tert*-butoxycarbonyl)(cyclopentyl)amino)phenyl)-3-(hydroxymethyl)piperidine-1-carboxylate **55** as a white solid, 54% (2 steps).

^{1}H NMR (500 MHz, $CDCl_3$) δ 7.33 (d, *J* = 7.9 Hz, 2H), 6.97 (d, *J* = 7.0 Hz, 2H), 5.24 (br s, 1H), 4.42 (p, *J* = 8.6 Hz, 1H), 4.01 (d, *J* = 10.8 Hz, 1H), 3.32-3.16 (m, 3H), 2.21 – 2.08 (m, 1H), 1.91 (br d, *J* = 12.6 Hz, 1H), 1.88 – 1.71 (m, 3H), 1.71-1.56 (m, 2H), 1.55-1.40 (m, 5H), 1.38 – 1.31 (m, 11H), 1.30 (s, 9H). **^{13}C NMR (126 MHz, $CDCl_3$)** δ 155.6, 155.3, 139.0, 129.5, 129.1, 79.8, 79.7, 64.8, 59.0, 56.4, 42.3, 40.8, 30.2, 28.4, 28.3, 24.7, 23.1, 21.6 (note: missing 1 aromatic peak due to overlap). **IR (neat):** 3453 (br, w), 2973 (w), 2933 (w), 2871 (w), 1672 (s), 1391 (m), 1364 (m), 1167 (s), 1149 (s), 730 (s) cm^{-1}. **HRMS (ESI):** Calculated for $C_{27}H_{42}O_5N_2Na$ [M+Na]$^+$: 497.2986, found: 497.2986.

***tert*-butyl-2-(4-((*tert*-butoxycarbonyl)(cyclopentyl)amino)phenyl)-3-formylpiperidine-1-carboxylate (SI-21)** was synthesized according to the following literature procedures[31]: A flame-dried reaction vial equipped with a stir bar was charged with *tert*-butyl-2-(4-((*tert*-butoxycarbonyl)(cyclopentyl)amino)phenyl)-3-(hydroxymethyl)piperidine-1-carboxylate **55** (177 mg, 0.373 mmol, 1.00 equiv.) and Dess-Martin Periodinane (237 mg, 0.559 mmol, 1.50 equiv.) and then evacuated/backfilled with N_2 X 3. DCM (1.90 mL) was added *via* syringe and the reaction was stirred at 25 °C for 3 h (until complete by TLC). The reaction mixture was quenched with sat. aq. $NaHCO_3$ (2 mL). The layers were separated, and the aqueous layer was extracted 3 X DCM (2 mL). The combined organic layers were dried over $MgSO_4$, gravity filtered, and concentrated *in vacuo.* The crude *tert*-butyl-2-(4-((*tert*-butoxycarbonyl)(cyclopentyl)amino)phenyl)-3-formylpiperidine-1-carboxylate **SI-21** was obtained as a white solid (119 mg, 67%) that was used directly in the next step without further purification.

^{1}H NMR (500 MHz, $CDCl_3$) δ 9.72 (s, 1H), 7.14 (d, *J* = 6.9 Hz, 2H), 6.98 (d, *J* = 7.1 Hz, 2H), 5.95 (br s, 1H), 4.37 (p, *J* = 8.8 Hz, 1H), 3.93 (br s, 1H), 2.87 – 2.74 (m, 1H), 2.67 (t, *J* = 13.7 Hz, 1H), 2.11 (d, *J* = 13.9 Hz, 1H), 1.96 – 1.70 (m, 4H), 1.59 – 1.40 (m, 15H), 1.33 (s, 10H). **^{13}C NMR (126 MHz, $CDCl_3$)** δ 201.8, 155.1, 154.9, 139.5, 136.9, 129.9, 128.3, 80.4, 79.9, 59.1, 52.2, 39.9, 30.2, 30.1, 28.5, 28.4, 24.5, 23.0, 19.8.

1-(*tert*-butoxycarbonyl)-2-(4-((*tert*-butoxycarbonyl)(cyclopentyl)amino)phenyl)piperidine-3-carboxylic acid (SI-22) was synthesized according to the following literature procedure[32]: A reaction vial equipped with a stir bar was charged with *tert*-butyl-2-(4-((*tert*-butoxycarbonyl)(cyclopentyl)amino)phenyl)-3-formylpiperidine-1-carboxylate **SI-21** (119 mg, 0.251 mmol, 1.00 equiv.), *tert*-butanol (1.60 mL) *via syringe*, and 2-methyl-2-butene (0.880 mL, 2.0 molar in THF, 1.76 mmol, 7.00 equiv.) *via syringe*. The mixture was cooled to 0 °C in an ice water bath and a mixture of sodium chlorite (113 mg, 1.25 mmol, 5.00 equiv) and sodium phosphate monobasic monohydrate (242 mg, 1.76 mmol, 7.00 equiv.) dissolved in 0.85 mL H_2O was added *via* syringe. The reaction was stirred for 1.5 h at 0 °C and then concentrated *in vacuo.* The crude residue was diluted with H_2O (2 mL) and the aqueous layer was extracted with ethyl acetate (3 X 2 mL). The combined organic layers were dried over Na_2SO_4, gravity filtered, and concentrated *in vacuo* to obtain the crude 1-(*tert*-butoxycarbonyl)-2-(4-((*tert*-butoxycarbonyl)(cyclopentyl)amino)phenyl)piperidine-3-carboxylic acid **SI-22** as a white foam (118 mg, 96%) that was used in the next step without further purification.

^{1}H NMR (500 MHz, CDCl$_3$) δ 9.47 (br s, 1H), 7.21 (d, *J* = 8.0 Hz, 2H), 6.96 (d, *J* = 7.1 Hz, 2H), 6.13 – 5.55 (br m, 1H), 4.37 (p, *J* = 8.6 Hz, 1H), 4.13 – 3.66 (br m, 1H), 3.01 – 2.79 (m, 1H), 2.60 (br s, 1H), 2.11 – 1.87 (m, 2H), 1.87 – 1.63 (m, 4H), 1.57 – 1.39 (m, 14H), 1.34 (s, 10H). **^{13}C NMR (101 MHz, CDCl$_3$, VT 50 ^{o}C)** δ 176.5, 155.4, 155.1, 139.3, 137.4, 129.6, 128.3, 80.4, 80.0, 60.7, 59.4, 44.7, 39.6, 30.3, 28.5, 28.4, 25.0, 23.2, 21.3.

***tert*-butyl-2-(4-((*tert*-butoxycarbonyl)(cyclopentyl)amino)phenyl)-3-((4-methyl-3-(trifluoromethyl)phenyl)carbamoyl)piperidine-1-carboxylate (SI-23)** was synthesized according to the following literature procedure[33]: An oven-dried reaction vial equipped with a stir bar was charged with 1-(*tert*-butoxycarbonyl)-2-(4-((*tert*-butoxycarbonyl)(cyclopentyl)amino)phenyl)piperidine-3-carboxylic acid **SI-22** (118 mg, 0.242 mmol, 1.20 equiv.), DMF (2.00 mL) *via* syringe, 4-methyl-3-(trifluoromethyl)aniline (29.0 μL, 0.202 mmol, 1.00 equiv) *via* syringe, N,N-Diisopropylethylamine (70.4 μL, 0.404 mmol, 2.00 equiv.) *via* syringe, and HATU (92.2 mg, 0.242 mmol, 1.20 equiv.) in one portion. The reaction was stirred at 25 ^{o}C for 6 h (until complete by TLC). The mixture was then diluted with ethyl acetate (2 mL) and the organic layer was washed with H_2O (1 X 3 mL) and brine (1 X 3 mL). The organic layer was dried over $MgSO_4$, gravity filtered, and concentrated *in vacuo.* Purification by MPLC (gradient: 0-20% ethyl acetate in hexanes) and a second MPLC (gradient: 0-50% diethyl ether in hexanes) yielded *tert*-butyl-2-(4-((*tert*-butoxycarbonyl)(cyclopentyl)amino)phenyl)-3-((4-methyl-3-(trifluoromethyl)phenyl)carbamoyl)piperidine-1-carboxylate **SI-23** as a white foam (54 mg, 42%).

^{1}H NMR (500 MHz, CDCl$_3$) δ 7.92 (br s, 1H), 7.63 (s, 1H), 7.39 (d, *J* = 8.4 Hz, 1H), 7.32 (d, *J* = 8.0 Hz, 2H), 7.11 (d, *J* = 8.4 Hz, 1H), 6.95 (d, *J* = 7.1 Hz, 2H), 5.86 (br s, 1H), 4.36 (p, *J* = 8.7 Hz, 1H), 3.98 (d, *J* = 13.7 Hz, 1H), 3.05 – 2.94 (m, 1H), 2.87 (t, *J* = 13.3 Hz, 1H), 2.38 (s, 3H), 2.19 – 1.98 (m, 2H), 1.92 – 1.72 (m, 3H), 1.68 – 1.50 (m, 1H), 1.43 (app. d, 13H), 1.31 (app. s, 11H). **^{13}C NMR (126 MHz, CDCl$_3$)** δ 170.6, 155.4, 155.2, 139.4, 137.0, 135.6, 132.4, 132.3, 129.8, 129.2 (q, *J* = 30.2 Hz), 128.4, 124.2 (q, *J* = 273.7 Hz), 123.2, 117.7 (q, *J* = 5.5 Hz), 80.5, 79.9, 59.2, 55.3, 47.2, 40.2, 30.1, 28.5, 28.4, 24.9, 23.0, 21.3, 18.8. **^{19}F NMR** (471 MHz, CDCl$_3$) δ -61.93. **IR (neat):** 2974 (w), 2941 (w), 1692 (m), 1664 (m), 1320 (m), 1164 (s), 1118 (s), 889 (w) cm^{-1}. **HRMS (ESI+):** Calculated for: $C_{35}H_{46}O_5N_3F_3Na$ $[M+Na]^+$: 668.3282, found: 668.3286.

2-(4-(cyclopentylamino)phenyl)-N-(4-methyl-3-(trifluoromethyl)phenyl)piperidine-3-carboxamide (SI-24) was synthesized in accordance with the following literature procedure[24]: An oven-dried reaction vial equipped with a stir bar was charged with *tert*-butyl-2-(4-((*tert*-butoxycarbonyl)(cyclopentyl)amino)phenyl)-3-((4-methyl-3-(trifluoromethyl)phenyl)carbamoyl)piperidine-1-carboxylate **SI-23** (53 mg, 0.082 mmol, 1.0 equiv.) and evacuated/backfilled with N_2 X 3. DCM (1.6 mL) was added to the reaction vial *via* syringe and the mixture was cooled to 0 ^{o}C in an ice water bath. Trifluoroacetic acid (0.13 mL, 1.6 mmol, 20 equiv.) was added dropwise *via* syringe, and then the reaction was warmed to 25 ^{o}C and stirred for 5 h (until complete by TLC). The mixture was concentrated *in vacuo*, and the crude residue was dissolved in ethyl acetate (2 mL) and washed with sat. aq. $NaHCO_3$ (1 X 2 mL), brine (1 X 2 mL), the organic layer was dried over $MgSO_4$, gravity filtered, and concentrated *in vacuo* to obtain 2-(4-(cyclopentylamino)phenyl)-N-(4-methyl-3-(trifluoromethyl)phenyl)piperidine-3-carboxamide **SI-24** as an off-white solid (26 mg, 72%).

^{1}H NMR (500 MHz, CD_3OD_SPE) δ 7.73 (s, 1H), 7.45 (d, *J* = 8.3 Hz, 1H), 7.24 (d, *J* = 8.3 Hz, 1H), 7.10 (d, *J* = 7.2 Hz, 2H), 6.60 (d, *J* = 6.7 Hz, 2H), 3.88 (d, *J* = 3.3 Hz, 1H), 3.71 (p, *J* = 6.5 Hz, 1H), 3.39 – 3.28 (m, 1H), 2.89 – 2.77 (m, 2H), 2.40 (s, 3H), 2.14 (d, *J* = 10.0 Hz, 1H), 2.09 – 1.98 (m, 2H), 1.98 – 1.85 (m, 2H), 1.70 (q, *J* = 6.1 Hz, 2H), 1.65 – 1.52 (m, 3H), 1.49 – 1.36 (m, 2H). **^{13}C NMR (126 MHz, CD_3OD_SPE)** δ 175.4, 149.0, 137.6, 133.5, 132.8, 131.1, 129.9 (q, *J* = 30.0 Hz), 129.3, 127.8, 124.4, 118.5 (q, *J* = 5.8 Hz), 114.7, 62.6, 56.0, 48.1, 48.0, 33.9, 29.3, 25.0, 22.3, 18.8. **^{19}F NMR (471 MHz, CD_3OD_SPE)** δ -63.17.

^{1}H NMR data matches that reported in the literature.[34]

12. Experiments to determine role of N-methyl-morpholine

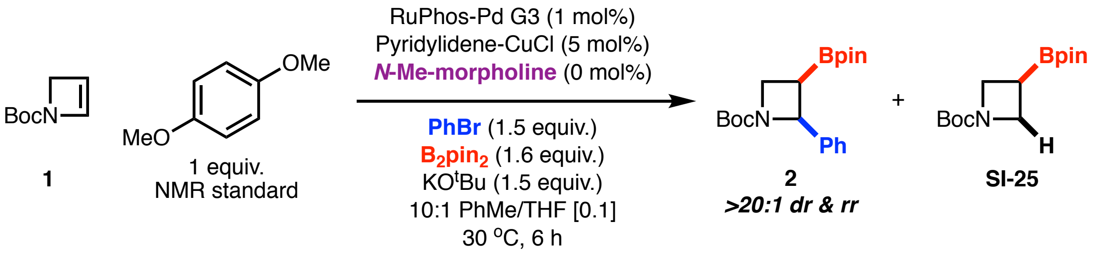

An oven-dried 100-mL round bottom flask equipped with a stir bar was brought into an N_2-filled glovebox and charged with RuPhos-Pd G3 (16.7 mg, 0.0200 mmol, 0.0100 equiv.), Pyridylidene-CuCl (53.3 mg, 0.100 mmol, 0.0500 equiv.), B_2pin_2 (813 mg, 3.20 mmol, 1.60 equiv.), and KO^{t}Bu (337 mg, 3.00 mmol, 1.50 equiv.). The flask was sealed with a septum and removed from the glovebox. A separate oven-dried 50-mL round bottom flask was charged with 1,4-dimethoxybenzene (276 mg, 2.00 mmol, 1.00 equiv.) and evacuated/backfilled with N_2 X 3. To the 50-mL flask was next added: 20.0 mL 10:1 PhMe/THF, N-Boc-2-azetine **1** (342 µL, 2.00 mmol, 1.00 equiv.), and bromobenzene (316 µL, 3.00 mmol, 1.50 equiv.) sequentially *via* syringes. This solution was transferred to the 100-mL round bottom flask *via* syringe while stirring, and a 0.2 mL aliquot of the reaction mixture was immediately quenched with 1 M HCl and the aqueous layer was extracted with ethyl acetate (3 X 1 mL) (Note: the alkene ring opened to *tert*-butyl (3-oxopropyl)carbamate and the aldehyde peak of this compound was used to calculate % remaining alkene in the ^{1}H NMR). The combined organic layers were dried over $MgSO_4$, gravity filtered, and concentrated *in vacuo*. An NMR was taken of the mixture and the remaining alkene starting material **1**, arylboration product **2** formation, and protoboration byproduct **SI-25** formation were calculated relative to 1,4-dimethoxybenzene as an internal standard. Similar aliquots were taken periodically over a 6 hour time period. Two outliers (t = 1 h and t = 2 h) were removed from the raw data. The mass balance of the remaining data points was calculated by adding the alkene **1**, arylboration product **2**, and protoboration byproduct **SI-25**. The data for each time point for both alkene **1** and arylboration product **2** was then normalized to 100% by dividing by the total mass balance for each time point and then multiplying x 100 to generate Plot **1**.

0 mol% N-Me-morpholine, raw data:

Reaction time (hours)	% alkene **1**	% arylboration **2**	% protoboration **SI-25**	Mass balance
0	109	1	2	112
0.5	79	40	9	128
1	18	44	4	66
1.5	54	48	4	106
2	14	55	7	76
3	44	53	7	104
4	42	52	7	101
5	40	54	7	101
6	40	54	8	102

0 mol% N-Me-morpholine, normalized data with outliers removed:

Reaction time (hours)	% alkene **1**	% arylboration **2**
0	97	1
0.5	62	31
1.5	51	45
3	42	51
4	42	51
5	40	53
6	39	53

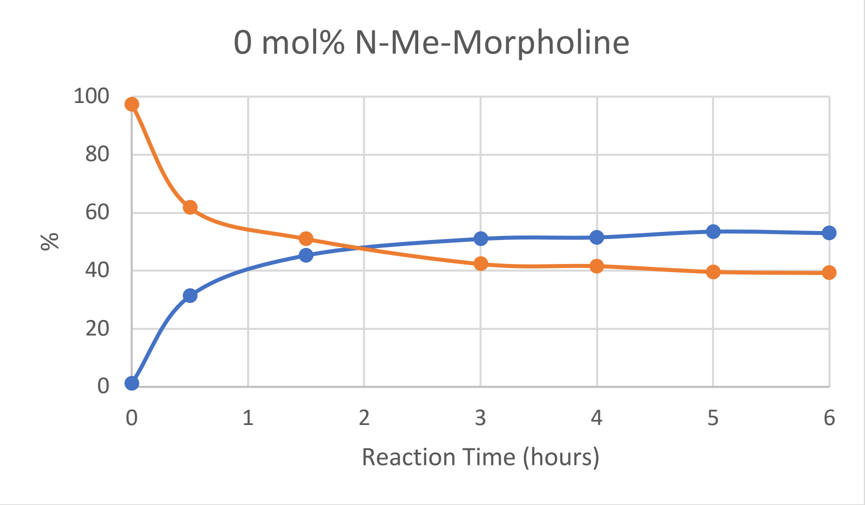

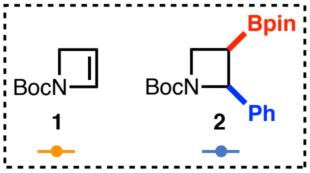

Plot **1**

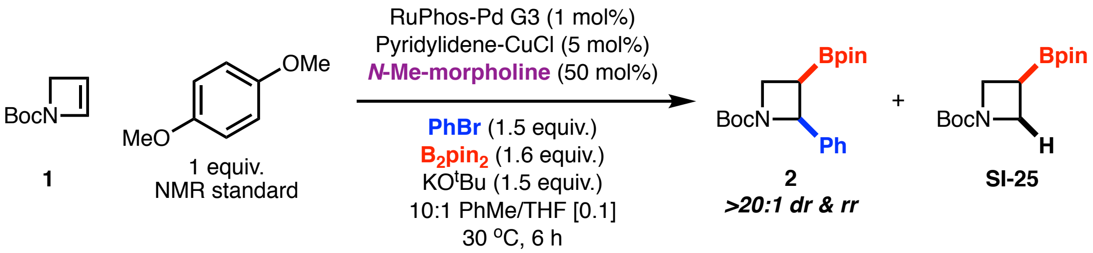

An oven-dried 100-mL round bottom flask equipped with a stir bar was brought into an N_2-filled glovebox and charged with RuPhos-Pd G3 (16.7 mg, 0.0200 mmol, 0.0100 equiv.), Pyridylidene-CuCl (53.3 mg, 0.100 mmol, 0.0500 equiv.), B_2pin_2 (813 mg, 3.20 mmol, 1.60 equiv.), and KO^{t}Bu (337 mg, 3.00 mmol, 1.50 equiv.). The flask was sealed with a septum and removed from the glovebox. A separate oven-dried 50-mL round bottom flask was charged with 1,4-dimethoxybenzene (276 mg, 2.00 mmol, 1.00 equiv.) and evacuated/backfilled with N_2 X 3. To the 50-mL flask was next added: 20.0 mL 10:1 PhMe/THF, *N*-Boc-2-azetine **1** (342 µL, 2.00 mmol, 1.00 equiv.), bromobenzene (316 µL, 3.00 mmol, 1.50 equiv.), and *N*-Me-morpholine (110 µL, 1.00 mmol, 0.500 equiv.) sequentially *via* syringes. This solution was transferred to the 100-mL round bottom flask *via* syringe while stirring, and a 0.2 mL aliquot of the reaction mixture was immediately quenched with 1 M HCl and the aqueous layer was extracted with ethyl acetate (3 X 1 mL)) (Note: the alkene ring opened to *tert*-butyl (3-oxopropyl)carbamate and the aldehyde peak of this compound was used to calculate % remaining alkene in the ^{1}H NMR). The combined organic layers were dried over $MgSO_4$, gravity filtered, and concentrated *in vacuo*. An NMR was taken of the mixture and the remaining alkene starting material **1**, arylboration product **2** formation, and protoboration byproduct **SI-25** formation were calculated relative to 1,4-dimethoxybenzene as an internal standard. Similar aliquots were taken periodically over a 6 hour time period. From the raw data, the mass balance of the data points was calculated by adding the alkene **1**, arylboration product **2**, and protoboration byproduct **SI-25**. The data for each time point for both alkene **1** and arylboration product **2** was then normalized to 100% by dividing by the total mass balance and then multiplying by 100 for each time point to generate Plot **2**.

50 mol% N-Me-morpholine, raw data:

Reaction time (hours)	% alkene **1**	% arylboration **2**	% protoboration **SI-25**	Mass balance
0	96	4	2	102
0.5	0	128	1	129
1	0	124	1	125
1.5	0	124	1	125
2	0	125	1	126
3	0	124	1	125
4	0	136	1	137
5	0	132	1	133
6	0	144	3	147

50 mol% N-Me-morpholine, normalized data:

Reaction time (hours)	% alkene **1**	% arylboration **2**
0	94	4
0.5	0	99

1	0	99
1.5	0	99
2	0	99
3	0	99
4	0	99
5	0	99
6	0	98

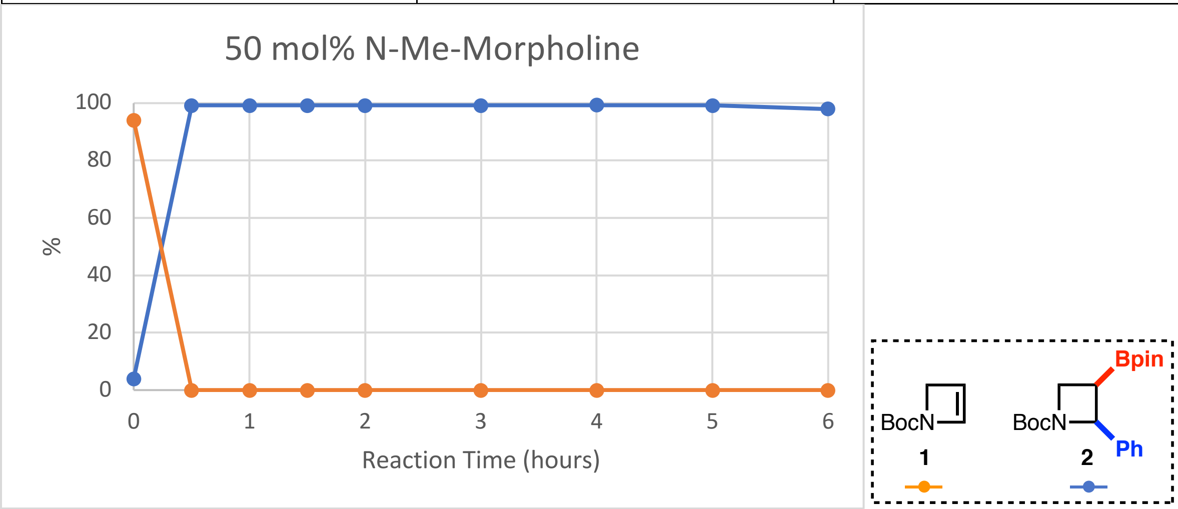

Plot **2**

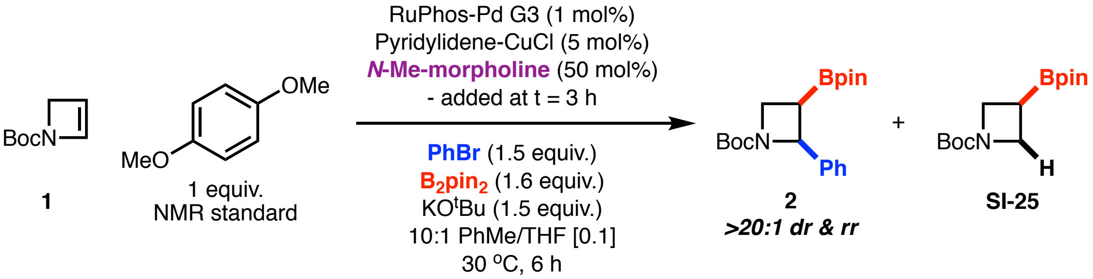

An oven-dried 100-mL round bottom flask equipped with a stir bar was brought into an N_2-filled glovebox and charged with RuPhos-Pd G3 (16.7 mg, 0.0200 mmol, 0.0100 equiv.), Pyridylidene-CuCl (53.3 mg, 0.100 mmol, 0.0500 equiv.), B_2pin_2 (813 mg, 3.20 mmol, 1.60 equiv.), and KO^{t}Bu (337 mg, 3.00 mmol, 1.50 equiv.). The flask was sealed with a septum and removed from the glovebox. A separate oven-dried 50-mL round bottom flask was charged with 1,4-dimethoxybenzene (276 mg, 2.00 mmol, 1.00 equiv.) and evacuated/backfilled with N_2 X 3. To the 50-mL flask was next added: 20.0 mL 10:1 PhMe/THF, *N*-Boc-2-azetine **1** (342 µL, 2.00 mmol, 1.00 equiv.), and bromobenzene (316 µL, 3.00 mmol, 1.50 equiv.) sequentially *via* syringes. This solution was transferred to the 100-mL round bottom flask *via* syringe while stirring, and a 0.2 mL aliquot of the reaction mixture was immediately quenched with 1 M HCl and the aqueous layer was extracted with ethyl acetate (3 X 1 mL)) (Note: the alkene ring opened to *tert*-butyl (3-oxopropyl)carbamate and the aldehyde peak of this compound was used to calculate % remaining alkene in the ^{1}H NMR). The combined organic layers were dried over $MgSO_4$, gravity filtered, and concentrated *in vacuo*. An NMR was taken of the mixture and the remaining alkene starting material **1**, arylboration product **2** formation, and protoboration byproduct **SI-25** formation were calculated relative to 1,4-dimethoxybenzene as an internal standard. Similar aliquots were taken

periodically over a 6 hour time period. Just before the aliquot at t = 3 h was taken, N-Me-morpholine (110 µL, 1.00 mmol, 0.500 equiv.) was added to the reaction *via* syringe and then the t = 3 h aliquot was immediately taken. One outlier (t = 1 h) was removed from the raw data. From the raw data, the mass balance of the data points was calculated by adding the alkene **1**, arylboration product **2**, and protoboration byproduct **SI-25**. The data for each time point for both alkene **1** and arylboration product **2** was then normalized to 100% by dividing by the total mass balance and then multiplying by 100 for each time point to generate Plot **3**.

50 mol% N-Me-morpholine at t = 3 h, raw data:

Reaction time (hours)	% alkene **1**	% arylboration **2**	% protoboration **SI-25**	Mass balance
0	91	3	13	107
0.5	34	41	6	81
1	42	50	4	96
1.5	32	59	4	95
2	30	61	5	96
3 (note: taken after adding N-Me-morpholine)	22	60	6	88
3.5	16	68	6	90
4	11	95	9	115
4.5	4	90	8	102
5	1	94	9	104
6	1	82	7	90

50 mol% N-Me-morpholine at t = 3 h, normalized data with outlier removed:

Reaction time (hours)	% alkene **1**	% arylboration **2**
0	85	3
0.5	42	51
1.5	34	62
2	31	64
3	25	68
3.5	18	76
4	10	83
4.5	4	88
5	1	90
6	1	91

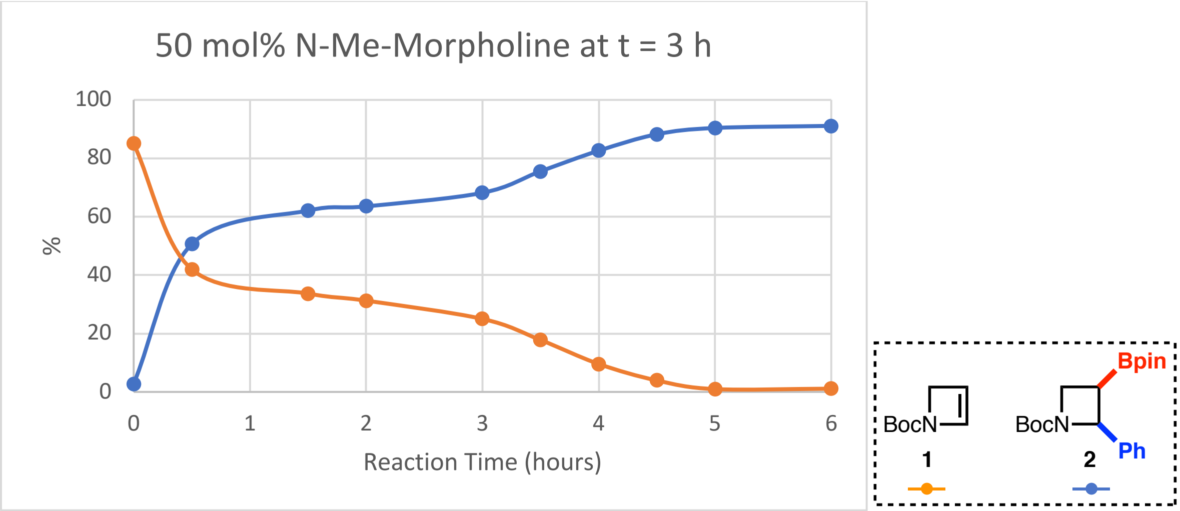

Plot **3**

13. References

[1] K. M. Logan, M. K. Brown. *Angew. Chem. Int. Ed.* **2017**, *56*, 851-855.
[2] J. Yu, V. Truc, P. Riebel, E. Hierl, B. Mudryk. *Tetrahedron Letters* **2005**, *46*, 4011-4013.
[3] D. M. Hodgson, T. J. Miles, J. Witherington. *Tetrahedron* **2003**, *59*, 9729-9742.
[4] J. Han, Y. Chen, C. Yang, T. Liu, M. Wand, H. Xu, L. Chang, C. Zheng, Y. Song, J. Zhu. *Eur. J. Med. Chem.* **2016**, *122*, 684-701.
[5] P. Kannaboina, K. Anilkumar, S. Aravinda, R. A. Vishwakarma, P. Das. *Org. Lett.* **2013**, *15*, 5718-5721.
[6] J. H. Clotet, L. O. Viturro, S. R. Ropero, P. D. Barjoan, WO 2018115362, June 28, 2018
[7] W. Marais, C. W. Holzapfel. *Synthetic Communication.* **1998**, *28*, 3681-3691.
[8] G. M. Pawar, B. Bantu, J. Weckesser, S. Blecher, K. Wurst, M. R. Buchmeiser. *Dalton Trans.* **2009**, *41*, 9043-9051.
[9] S. Díez-González, E. C. Escudero-Adán, J. Benet-Buchholz, E. D. Stevens, A. M. Z. Slawin, S. P. Nolan. *Dalton Trans.* **2010**, *39*, 7595-7606.
[10] Y. Huang, M. K. Brown. *Angew. Chem. Int. Ed.* **2019**, *58*, 6048-6052.
[11] N. C. Bruno, M. T. Tudge, S. L. Buchwald. *Chem. Sci.* **2013**, *4*, 916-920.
[12] G. Dubinima, J. Ogikubo, D. Vicic. *Organometallics*. **2008**, *27*, 6233-6235.
[13] M. Chen, Z.-H. Ren, Y.-Y. Wang, Z.-H. Guan. *J. Org. Chem.* **2015**, *80*, 1258-1263.
[14] D. F. Oliveira, P. C. M. L. Miranda, C. R. D. Correia. *J. Org. Chem.* **1999**, *64*, 6646-6652.
[15] R. I. McDonald, G. W. Wong, R. P. Neupane, S. S. Stahl, C. R. Landis. *J. Am. Chem. Soc.* **2010**, *132*, 14027-14029.
[16] W. Delong, W. Lanying, W. Yongling, W. Shuang, F. Juntao, Z. Xing. *Eur. J. Med. Chem.* **2017**, *130*, 286-407.
[17] À. Mourelle-Insua, L. A. Zampieri, I. Lavandera, V. Gotor-Fernández. *Adv. Synth. Catal.* **2018**, *360*, 686-695.
[18] V. Gasparik, H. Greney, S. Schann, J. Feldman, L. Fellmann, J.-D. Ehrhardt, P. Bousquet. *J. Med. Chem.* **2015**, *58*, 878-887.
[19] A. O. Maldaner, R. A. Pilli. *Tetrahedron* **1999**, *55*, 13321-13332.
[20] E. M. Boyd, J. Sperry. *Tetrahedron Letters* **2012**, *53*, 3623-3626.
[21] P. Sarma, S. Kumara, V. Acharya, S. R. Kasibhatla, A. Tiwari, V. N. Reddy, A. Bischoff. U.S. Patent WO 2010127208, April 30, 2010.
[22] Z.-B. Zhang, C.-L. Ji, C. Yang, J. Chen, X. Hong, J.-B. Xia. *Org. Lett.* **2019**, *21*, 1226-1231.
[23] A. M. Bergmann, S. K. Dorn, K. B. Smith, K. M. Logan, M. K. Brown. *Angew. Chem. Int. Ed.* **2019**, *58*, 1719-1723.
[24] F. Lafzi, H. Kilic, G. Tanriver, Ö. N. Avci, S. Catak, N. Saracoglu. *Synthetic Communications* **2019**, *49*, 3510-3527.
[25] G. L. Trammel, R. Kuniyil, P. F. Crook, P. Liu, M. K. Brown. *J. Am. Chem. Soc.* **2021**, *143*, 16502-16511.
[26] N. W. Goldberg, X. Shen, J. Li, T. Ritter. *Org. Lett.* **2016**, *18*, 6102-6104.

[27] A. K. Simlandy, M.-Y. Lyu, M. K. Brown. *ACS Catal.* **2021**, *11*, 12815-12820.
[28] P. E. Finke, L. C. Meurer, S. G. Mills, M. Maccoss, H. Qi. U.S. Patent WO 2002024629, March 28, 2002.
[29] C. Quinet, L. Sampoux, I. E. Markó. *Eur. J. Org. Chem.* **2009**, *11*, 1806-1811.
[30] T. Harrison, B. J. Williams, C. J. Swain, R. G. Ball. *Bioorganic & Medicinal Chemistry Letters* **1994**, *4*, 2545-2550.
[31] S. R. Sardini, A. L. Lambright, G. L. Trammel, H. M. Omer, P. Liu, M. K. Brown. *J. Am. Chem. Soc.* **2019**, *141*, 9391-9400.
[32] J. Fan, J. Kalisiak, R. M. Lui, V. R. Mali, J. P. Mcmahon, J. P. Powers, H. Tanaka, Y. Zeng, P. Zhang. U.S. Patent WO 2016187393, November 24, 2016.
[33] S. Oh, D. Y. Kwon, I. Choi, Y. M. Kim, J. Y. Lee, J. Ryu, H. Jeong, M. J. Kim, R. Song. *ACS Med. Chem. Lett.* **2021**, *12*, 563-571.
[34] P. Fan, J. Kalisiak, A. Krasinski, R. Lui, J. Powers, S. Punna, H. Tanaka, P. Zhang. U.S. Patent WO 20160090357, March 31, 2016.

Data File C:\CHEM32\2\DATA\GLT\GLT-VI-15RAC001.D
Sample Name: GLT-VI-15RAC

===

Acq. Operator : SYSTEM   Seq. Line : 1
Acq. Instrument : 1220 HPLC   Location : Vial 31
Injection Date : 5/1/2022 12:35:35 PM   Inj : 1
Inj Volume : 10.000 µl
Acq. Method : C:\CHEM32\2\METHODS\MLC_VARIABLE.M
Last changed : 5/1/2022 12:29:30 PM by SYSTEM
Analysis Method : C:\CHEM32\2\METHODS\DEF_LC.M
Last changed : 7/15/2022 1:18:30 PM by SYSTEM
Sample Info : LUX 3U AMYLOSE-2, HEX:IPA=95:5, 0.5ml/min, 254nm

Additional Info : Peak(s) manually integrated

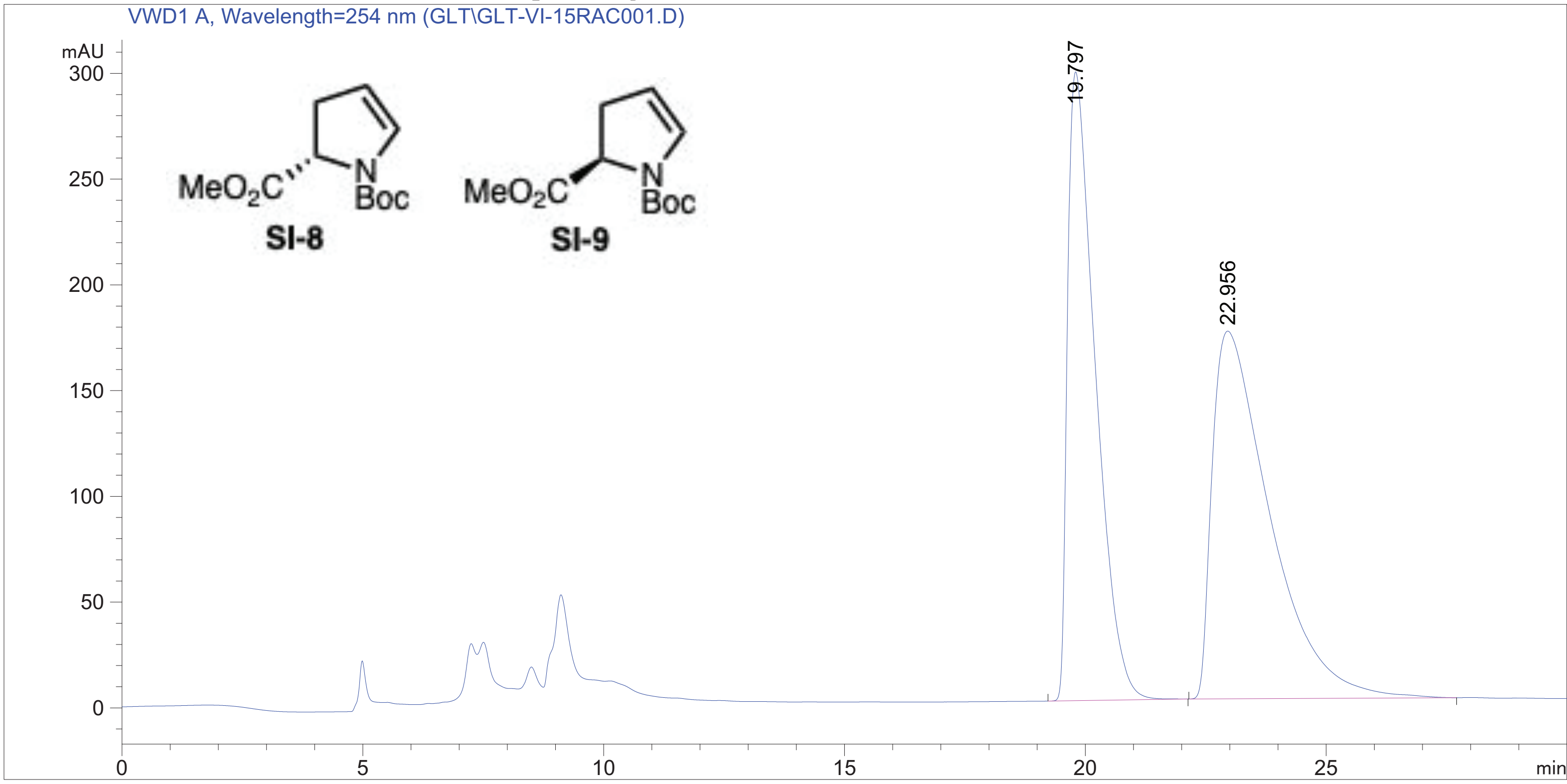

===

Area Percent Report

===

Sorted By : Signal
Multiplier: : 1.0000
Dilution: : 1.0000
Use Multiplier & Dilution Factor with ISTDs

Signal 1: VWD1 A, Wavelength=254 nm

Peak #	RetTime [min]	Type	Width [min]	Area [mAU*s]	Height [mAU]	Area %
1	19.797	BB	0.6065	1.19140e4	297.34070	45.1287
2	22.956	BB	1.2330	1.44861e4	173.86546	54.8713

Totals : 2.64001e4 471.20616

===

*** End of Report ***

Data File C:\CHEM32\2\DATA\GLT\GLT-VI-15000001.D
Sample Name: GLT-VI-15

===

Acq. Operator : SYSTEM Seq. Line : 1
Acq. Instrument : 1220 HPLC Location : Vial 31
Injection Date : 5/1/2022 1:26:14 PM Inj : 1
Inj Volume : 10.000 µl
Acq. Method : C:\CHEM32\2\METHODS\MLC_VARIABLE.M
Last changed : 5/1/2022 12:29:30 PM by SYSTEM
Analysis Method : C:\CHEM32\2\METHODS\DEF_LC.M
Last changed : 7/15/2022 1:18:30 PM by SYSTEM
Sample Info : LUX 3U AMYLOSE-2, HEX:IPA=95:5, 0.5ml/min, 254nm

Additional Info : Peak(s) manually integrated

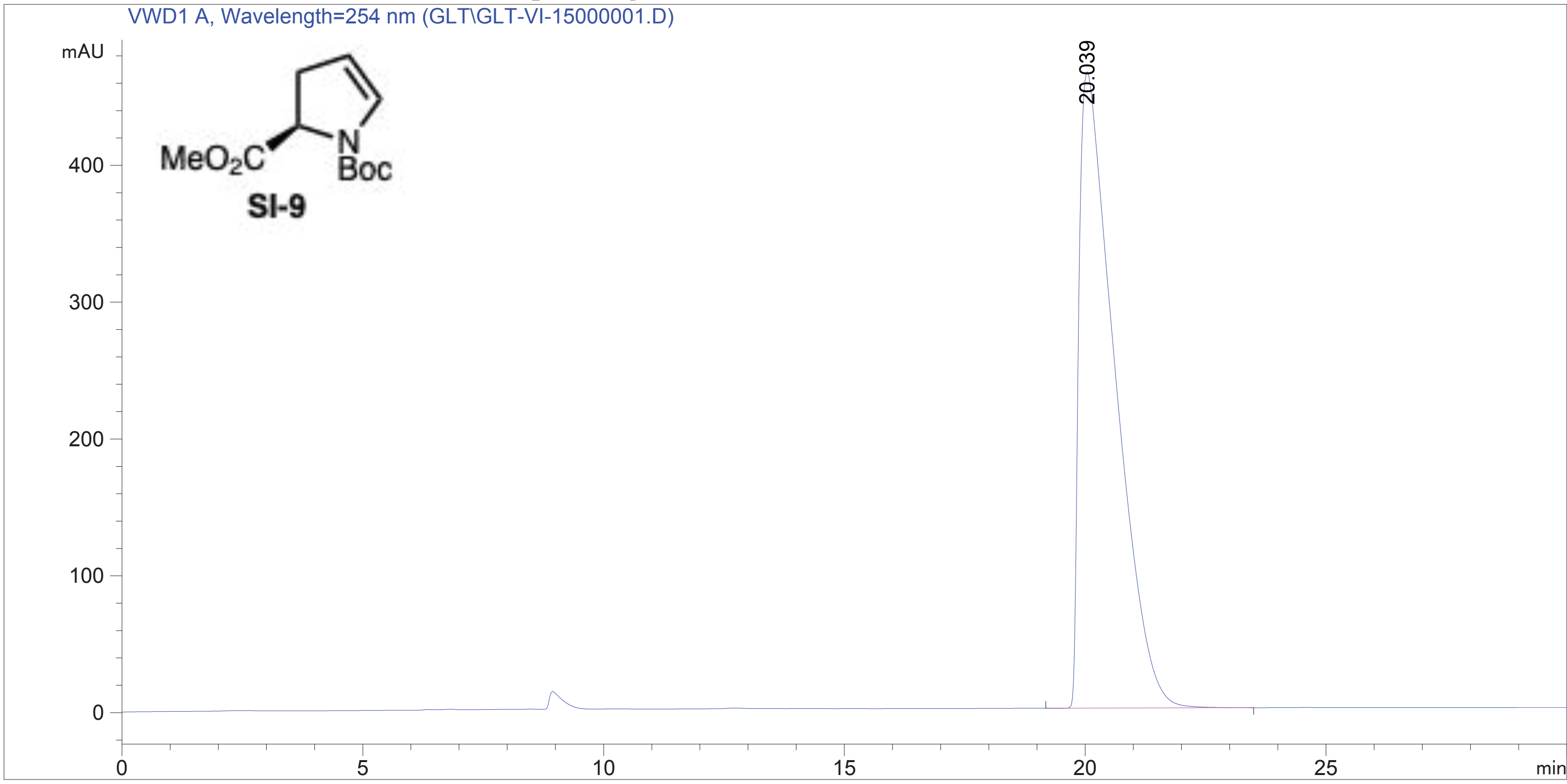

===

Area Percent Report

===

Sorted By : Signal
Multiplier: : 1.0000
Dilution: : 1.0000
Use Multiplier & Dilution Factor with ISTDs

Signal 1: VWD1 A, Wavelength=254 nm

Peak #	RetTime [min]	Type	Width [min]	Area [mAU*s]	Height [mAU]	Area %
1	20.039	BB	0.7554	2.40497e4	465.01044	100.0000

Totals : 2.40497e4 465.01044

===

*** End of Report ***

Data File C:\CHEM32\2\DATA\GLT\GLT-V-482000001.D
Sample Name: GLT-V-482

===

Acq. Operator : SYSTEM   Seq. Line : 1
Acq. Instrument : 1220 HPLC   Location : Vial 31
Injection Date : 5/1/2022 2:37:32 PM   Inj : 1
Inj Volume : 10.000 µl
Acq. Method : C:\CHEM32\2\METHODS\MLC_VARIABLE.M
Last changed : 5/1/2022 12:29:30 PM by SYSTEM
Analysis Method : C:\CHEM32\2\METHODS\DEF_LC.M
Last changed : 7/15/2022 1:18:30 PM by SYSTEM
Sample Info : LUX 3U AMYLOSE-2, HEX:IPA=95:5, 0.5ml/min, 254nm

Additional Info : Peak(s) manually integrated

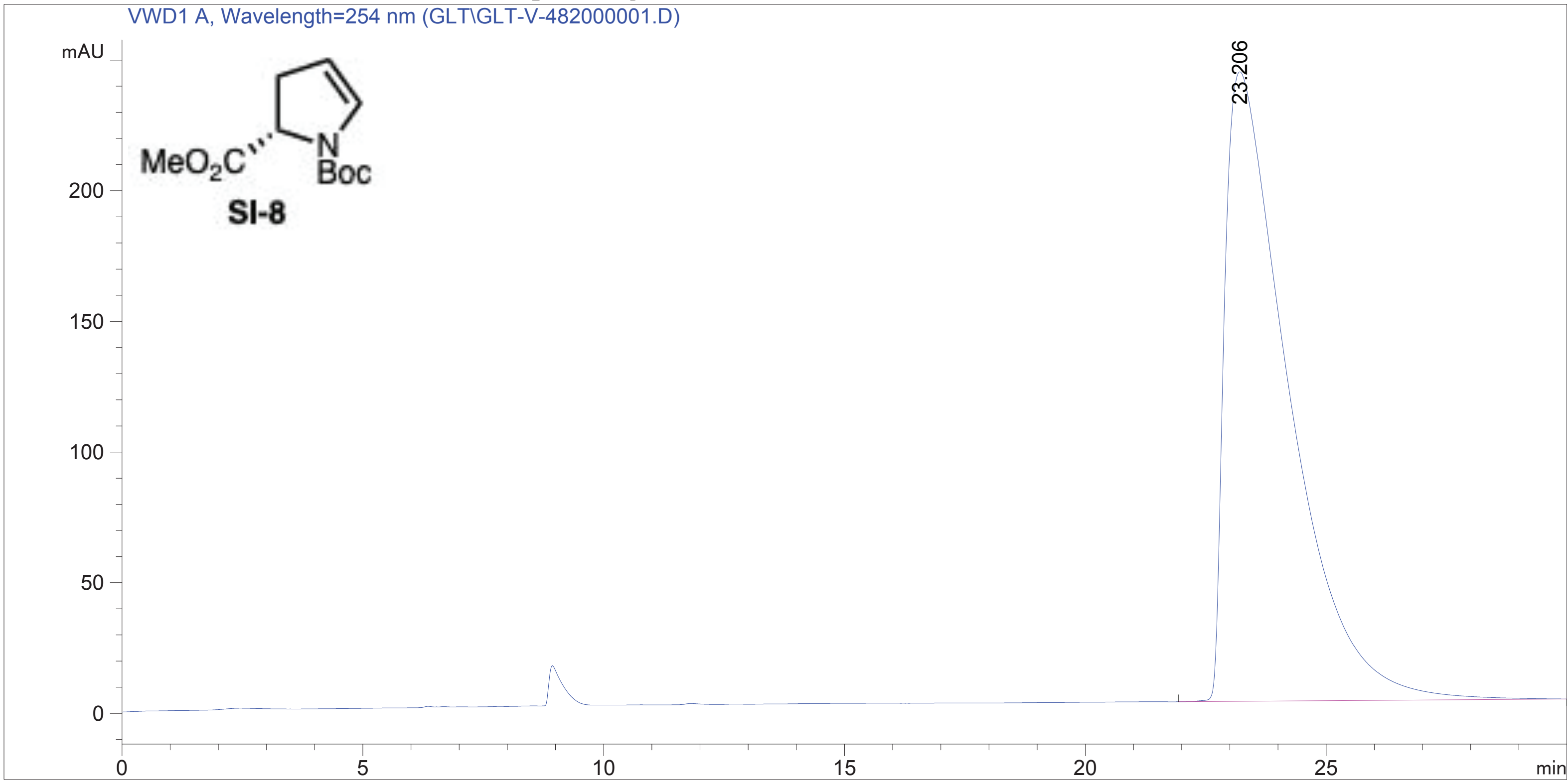

===
Area Percent Report
===

Sorted By : Signal
Multiplier: : 1.0000
Dilution: : 1.0000
Use Multiplier & Dilution Factor with ISTDs

Signal 1: VWD1 A, Wavelength=254 nm

Peak #	RetTime [min]	Type	Width [min]	Area [mAU*s]	Height [mAU]	Area %
1	23.206	BBA	1.3503	2.22628e4	240.87943	100.0000

Totals : 2.22628e4 240.87943

===
*** End of Report ***

Data File C:\CHEM32\2\DATA\PK\GLT-VI-48700002.D
Sample Name: GLT-VI-487-2

===

Acq. Operator : SYSTEM Seq. Line : 2
Acq. Instrument : 1220 HPLC Location : Vial 31
Injection Date : 9/15/2022 12:50:18 PM Inj : 1
Inj Volume : 10.000 µl
Acq. Method : C:\CHEM32\2\METHODS\MLC_VARIABLE.M
Last changed : 9/10/2022 4:55:36 PM by SYSTEM
Analysis Method : C:\CHEM32\2\METHODS\DEF_LC.M
Sample Info : CHIRALPAK-IA 95:5 Hex:IPA, 0.5 mL/min, 220 nm, 20 min

Additional Info : Peak(s) manually integrated

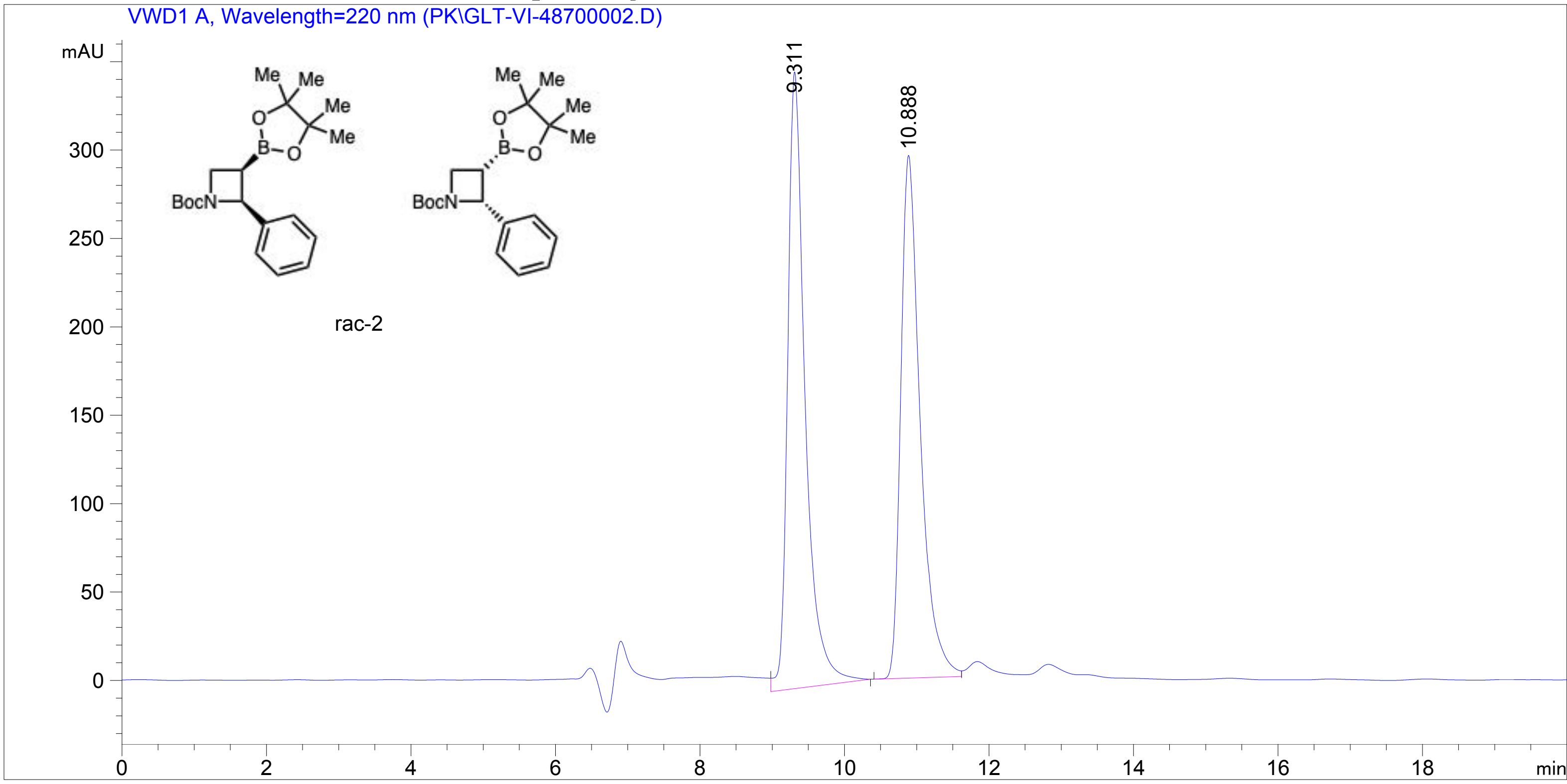

===

Area Percent Report

===

Sorted By : Signal
Multiplier: : 1.0000
Dilution: : 1.0000
Use Multiplier & Dilution Factor with ISTDs

Signal 1: VWD1 A, Wavelength=220 nm

Peak #	RetTime [min]	Type	Width [min]	Area [mAU*s]	Height [mAU]	Area %
1	9.311	VB	0.2500	5858.90576	348.81668	51.4456
2	10.888	BV	0.2812	5529.63721	295.64664	48.5544
Totals :				1.13885e4	644.46332	

===

*** End of Report ***

Data File C:\CHEM32\2\DATA\PK\PK-I-2100000002.D
Sample Name: PK-I-210-2

===

Acq. Operator : SYSTEM Seq. Line : 2
Acq. Instrument : 1220 HPLC Location : Vial 31
Injection Date : 9/12/2022 1:28:04 PM Inj : 1
Inj Volume : 10.000 µl
Acq. Method : C:\CHEM32\2\METHODS\MLC_VARIABLE.M
Last changed : 9/10/2022 4:55:36 PM by SYSTEM
Analysis Method : C:\CHEM32\2\METHODS\DEF_LC.M
Sample Info : CHIRALPAK-IA 95:5 Hex:IPA, 0.5 mL/min, 220 nm, 20 min

Additional Info : Peak(s) manually integrated

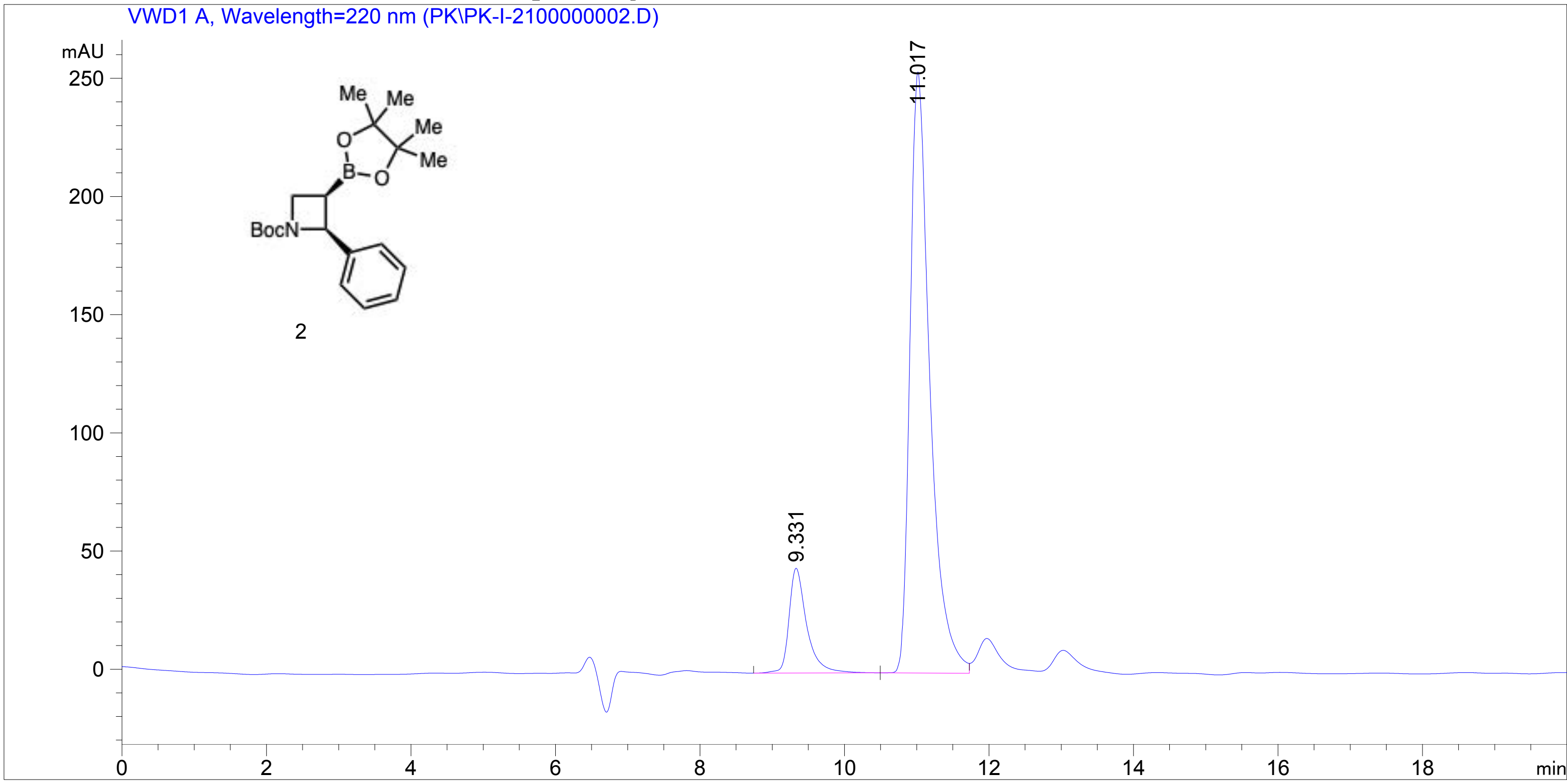

===
Area Percent Report
===

Sorted By : Signal
Multiplier: : 1.0000
Dilution: : 1.0000
Use Multiplier & Dilution Factor with ISTDs

Signal 1: VWD1 A, Wavelength=220 nm

Peak #	RetTime [min]	Type	Width [min]	Area [mAU*s]	Height [mAU]	Area %
1	9.331	BB	0.2521	752.55426	44.32791	13.3826
2	11.017	BV	0.2875	4870.82959	254.27754	86.6174

Totals : 5623.38385 298.60545

===
*** End of Report ***

Data File C:\CHEM32\2\DATA\GLT\GLT-VI-143RAC04.D
Sample Name: GLT-VI-143RAC

===

Acq. Operator : SYSTEM Seq. Line : 1
Acq. Instrument : 1220 HPLC Location : Vial 31
Injection Date : 8/1/2022 7:44:55 AM Inj : 1
Inj Volume : 5.000 µl
Acq. Method : C:\CHEM32\2\METHODS\MLC_VARIABLE.M
Last changed : 8/1/2022 8:16:57 AM by SYSTEM
(modified after loading)
Analysis Method : C:\CHEM32\2\METHODS\DEF_LC.M
Last changed : 8/1/2022 5:38:19 PM by SYSTEM
Sample Info : CHIRALPAK IA, HEX:IPA=80:20, 0.5ml/min, 254nm

Additional Info : Peak(s) manually integrated

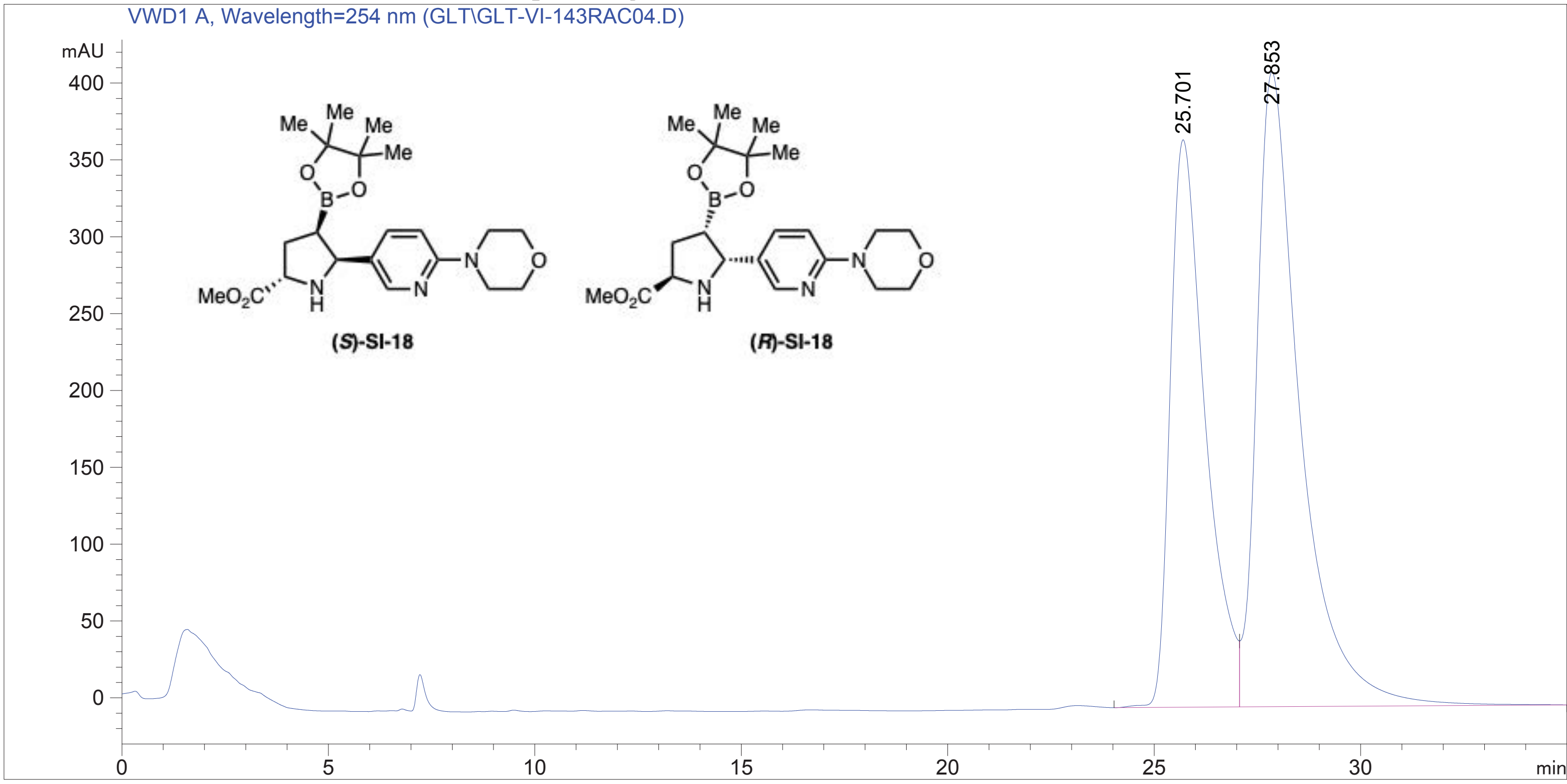

===

Area Percent Report

===

Sorted By : Signal
Multiplier: : 1.0000
Dilution: : 1.0000
Use Multiplier & Dilution Factor with ISTDs

Signal 1: VWD1 A, Wavelength=254 nm

Peak #	RetTime [min]	Type	Width [min]	Area [mAU*s]	Height [mAU]	Area %
1	25.701	BV	0.8890	2.18026e4	369.18289	41.8799
2	27.853	VBA	1.0775	3.02571e4	413.10760	58.1201

Totals : 5.20597e4 782.29050

===

*** End of Report ***

Data File C:\CHEM32\2\DATA\GLT\GLT-VI-14200003.D
Sample Name: GLT-VI-142

===

Acq. Operator : SYSTEM Seq. Line : 1
Acq. Instrument : 1220 HPLC Location : Vial 31
Injection Date : 8/1/2022 9:16:13 AM Inj : 1
Inj Volume : 5.000 µl
Acq. Method : C:\CHEM32\2\METHODS\MLC_VARIABLE.M
Last changed : 8/1/2022 8:26:26 AM by SYSTEM
Analysis Method : C:\CHEM32\2\METHODS\DEF_LC.M
Last changed : 8/1/2022 5:38:19 PM by SYSTEM
Sample Info : CHIRALPAK IA, HEX:IPA=80:20, 0.5ml/min, 254nm

Additional Info : Peak(s) manually integrated

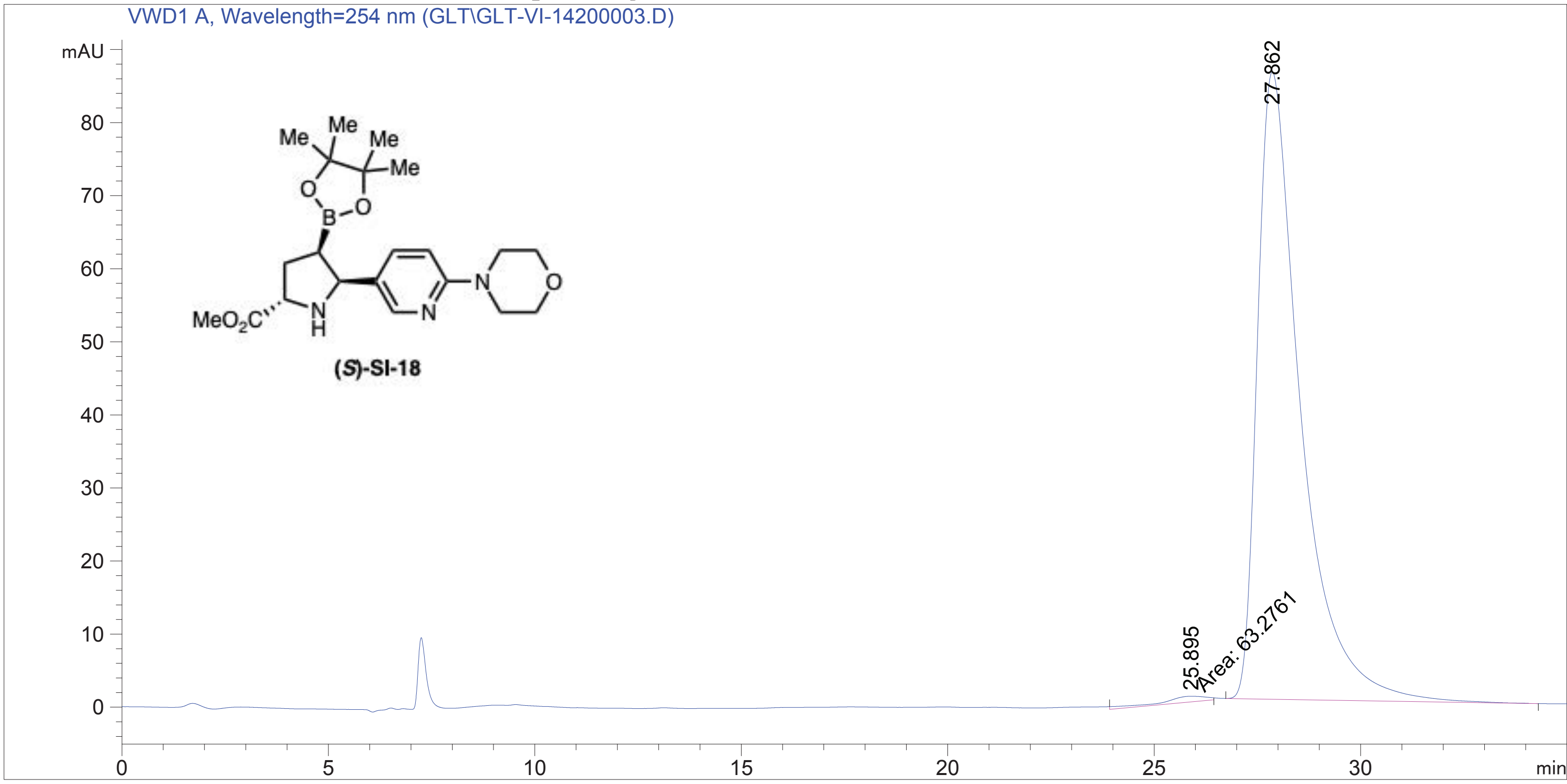

===
Area Percent Report
===

Sorted By : Signal
Multiplier: : 1.0000
Dilution: : 1.0000
Use Multiplier & Dilution Factor with ISTDs

Signal 1: VWD1 A, Wavelength=254 nm

Peak #	RetTime [min]	Type	Width [min]	Area [mAU*s]	Height [mAU]	Area %
1	25.895	MM	0.9642	63.27610	7.78523e-1	1.0030
2	27.862	BB	1.0747	6245.52002	85.85497	98.9970

Totals : 6308.79612 86.63349

===
*** End of Report ***

Data File C:\CHEM32\2\DATA\GLT\GLT-VI-14300006.D
Sample Name: GLT-VI-143

===

Acq. Operator : SYSTEM   Seq. Line : 1
Acq. Instrument : 1220 HPLC   Location : Vial 31
Injection Date : 8/1/2022 8:32:25 AM   Inj : 1
Inj Volume : 5.000 µl
Acq. Method : C:\CHEM32\2\METHODS\MLC_VARIABLE.M
Last changed : 8/1/2022 8:26:26 AM by SYSTEM
Analysis Method : C:\CHEM32\2\METHODS\DEF_LC.M
Last changed : 8/1/2022 5:38:19 PM by SYSTEM
Sample Info : CHIRALPAK IA, HEX:IPA=80:20, 0.5ml/min, 254nm

Additional Info : Peak(s) manually integrated

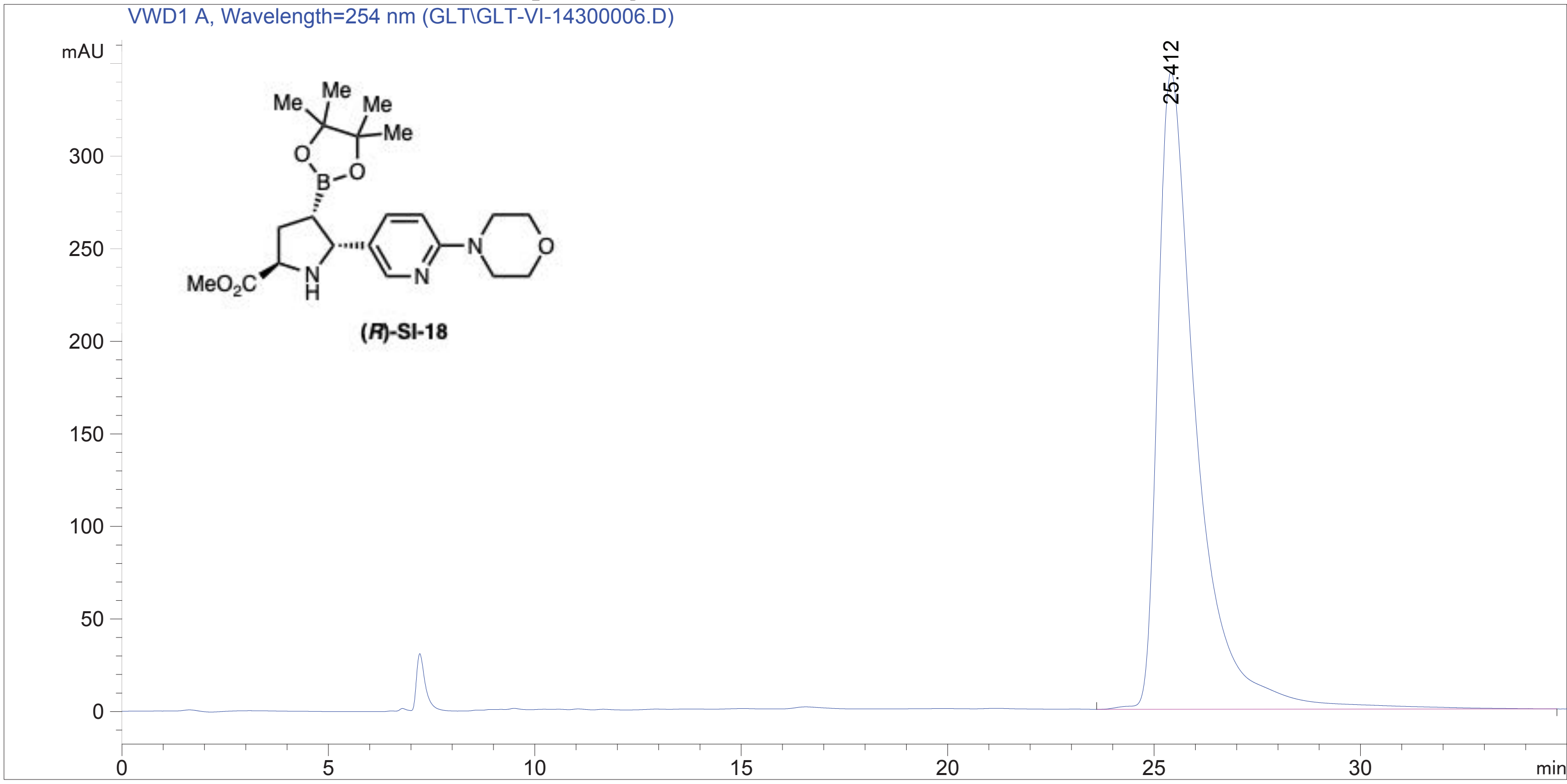

===
Area Percent Report
===

Sorted By : Signal
Multiplier: : 1.0000
Dilution: : 1.0000
Use Multiplier & Dilution Factor with ISTDs

Signal 1: VWD1 A, Wavelength=254 nm

Peak #	RetTime [min]	Type	Width [min]	Area [mAU*s]	Height [mAU]	Area %
1	25.412	BB	0.9598	2.25181e4	344.38416	100.0000

Totals : 2.25181e4 344.38416

===
*** End of Report ***

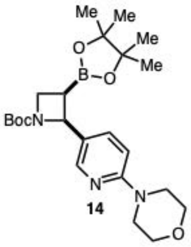

14

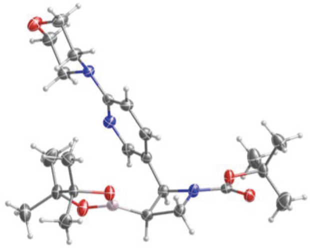

The sample was submitted by Grace Trammel (research group of Kevin Brown, Department of Chemistry, Indiana University). A colorless crystal (approximate dimensions 0.230 × 0.172 × 0.172 mm^3) was placed onto the tip of a MiTeGen loop and mounted on a Bruker Venture D8 diffractometer equipped with a PhotonIII detector at 153(2) K.

Data collection

The data collection was carried out using Mo Kα radiation (graphite monochromator) with a frame time of 1 and 20 seconds and a detector distance of 5.00 cm. A collection strategy was calculated and complete data to a resolution of 0.80 Å with a redundancy of 13.5 were collected. Ten major sections of frames were collected with 1º ω and ϕ scans. A total of 1648 frames were collected. The total exposure time was 4.81 hours. The frames were integrated with the Bruker SAINT software package[1] using a narrow-frame algorithm. The integration of the data using a monoclinic unit cell yielded a total of 58578 reflections to a maximum θ angle of 25.04° (0.84 Å resolution), of which 4252 were independent (average redundancy 13.777, completeness = 99.9%, R_{int} = 8.97%, R_{sig} = 3.71%) and 3547 (83.42%) were greater than 2σ(F^2). The final cell constants of a = 10.5425(5) Å, b = 6.6019(3) Å, c = 34.9155(18) Å, β = 97.743(2)°, volume = 2408.0(2) $Å^3$, are based upon the refinement of the XYZ-centroids of 7892 reflections above 20 σ(I) with 4.709° < 2θ < 49.42°. Data were corrected for absorption effects using the Multi-Scan method (SADABS[2]). The ratio of minimum to maximum apparent transmission was 0.872. The calculated minimum and maximum transmission coefficients (based on crystal size) are 0.9870 and 0.9970. Please refer to Table 1 for additional crystal and refinement information.

Structure solution and refinement

The space group $P2_1/c$ was determined based on intensity statistics and systematic absences. The structure was solved and refined using the SHELX suite of programs.[3,4] An intrinsic-methods solution was calculated, which provided most non-hydrogen atoms from the E-map. Full-matrix least squares / difference Fourier cycles were performed, which located the remaining non-hydrogen atoms. All non-hydrogen atoms were refined with anisotropic displacement parameters. The hydrogen atoms were placed in ideal positions and refined as riding atoms with relative isotropic displacement parameters. The final anisotropic full-matrix least-squares refinement on F^2 with 296 variables converged at R1 = 7.38%, for the observed data and wR2 = 13.37% for all data. The goodness-of-fit was 1.218. The largest peak in the final difference electron density synthesis was 0.216 $e^-/Å^3$ and the largest hole was -0.272 $e^-/Å^3$ with an RMS deviation of 0.052 $e^-/Å^3$. On the basis of the final model, the calculated density was 1.228 g/cm^3 and F(000), 960 e^-.

1 SAINT V8.40A (2020), Bruker AXS, Madison, WI, 2018.
2 L. Krause, R. Herbst-Irmer, G. M. Sheldrick, D. Stalke:
Comparison of silver and molybdenum microfocus X-ray sources for single-crystal structure determination.
J. Appl. Cryst., 48, 3-10 (2015). doi:10.1107/S1600576714022985.
3 G. M. Sheldrick:
SHELXT--Integrated space-group and crystal-structure determination.
Acta Cryst. A71, 3-8 (2015). doi:10.1107/S2053273314026370.
4 G. M. Sheldrick:
Crystal structure refinement with SHELXL.
Acta Cryst. C71, 3-8 (2015). doi:10.1107/S2053229614024218.

Table 1. Crystal data and structure refinement for *14*.

Empirical formula	C23 H36 B N3 O5	
Formula weight	445.36	
Crystal color, shape, size	colourless plate, 0.153 × 0.096 × 0.036 mm^3	
Temperature	150(2) K	
Wavelength	0.71073 Å	
Crystal system, space group	Monoclinic, $P2_1/c$	
Unit cell dimensions	a = 10.5425(5) Å	α = 90°.
	b = 6.6019(3) Å	β = 97.743(2)°.
	c = 34.9155(18) Å	γ = 90°.
Volume	2408.0(2) $Å^3$	
Z	4	
Density (calculated)	1.228 Mg/m^3	
Absorption coefficient	0.086 mm^{-1}	
F(000)	960	

Data collection

Diffractometer	Venture D8, Bruker
Source	Iμ3.0, Incoatec
Detector	Photon III
Theta range for data collection	1.949 to 25.037°.
Index ranges	-12<=h<=12, -7<=k<=7, -41<=l<=41
Reflections collected	58578
Independent reflections	4252 [R_{int} = 0.0897]
Observed Reflections	3547
Completeness to theta = 25.037°	99.9 %

Solution and Refinement

Absorption correction	Semi-empirical from equivalents
Max. and min. transmission	0.7452 and 0.6497
Solution	Intrinsic methods
Refinement method	Full-matrix least-squares on F^2
Weighting scheme	$w = [\sigma^2Fo^2 + 4.4047P]^{-1}$, with $P = (Fo^2 + 2 Fc^2)/3$
Data / restraints / parameters	4252 / 0 / 296
Goodness-of-fit on F^2	1.218
Final R indices [I>2σ(I)]	R1 = 0.0738, wR2 = 0.1287
R indices (all data)	R1 = 0.0888, wR2 = 0.1337
Largest diff. peak and hole	0.216 and -0.272 e.$Å^{-3}$

Goodness-of-fit = $[\Sigma[w(F_o^2 - F_c^2)^2]/N_{observns} - N_{params})]^{1/2}$, all data.

R1 = $\Sigma(|F_o| - |F_c|) / \Sigma |F_o|$. wR2 = $[\Sigma[w(F_o^2 - F_c^2)^2] / \Sigma [w(F_o^2)^2]]^{1/2}$.

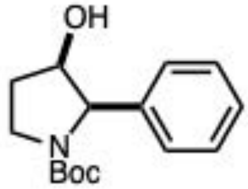

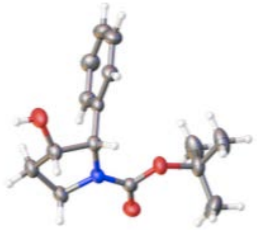

20

The sample was submitted by Grace Trammel (research group of professor Kevin Brown, Department of Chemistry, Indiana University).

Single crystals suitable for X-ray diffraction were grown by slow diffusion of diethyl ether in a pentane solution. A colourless crystal (plate, approximate dimensions 0.53 × 0.08 × 0.04 mm^3) was placed onto the tip of a MiTeGen pin and mounted on a Bruker Venture D8 diffractometer equipped with a PhotonIII detector at 179.0 K.

Data collection

The data collection was carried out using Mo Kα radiation (λ = 0.71073 Å, graphite monochromator) with a frame time of 2 seconds for low angle scans and 8 seconds for high angle scans. The detector distance was 120 mm. A collection strategy was calculated and complete data to a resolution of 0.70 Å with a redundancy of 7 were collected. A total of 3464 frames were collected. The total exposure time was 6.10 hours. The frames were integrated with the Bruker SAINT[1] software package using a narrow-frame algorithm. The integration of the data using a monoclinic unit cell yielded a total of 47521 reflections to a maximum θ angle of 27.51° (0.77 Å resolution), of which 3385 were independent (average redundancy 14.039, completeness = 99.9%, R_{int} = 11.83%, R_{sig} = 3.69%) and 2623 (77.49%) were greater than $2\sigma(F^2)$. The final cell constants of a = 6.0063(4) Å, b = 25.4606(18) Å, c = 9.8048(7) Å, β = 100.855(3)°, volume = 1472.56(18) Å3, are based upon the refinement of the XYZ-centroids of 5092 reflections above 20 σ(I) with 4.522° < 2θ < 48.95°. Data were corrected for absorption effects using the Multi-Scan method (SADABS).[2] The ratio of minimum to maximum apparent transmission was 0.748. The calculated minimum and maximum transmission coefficients (based on crystal size) are 0.9580 and 0.9970. Please refer to Table 1 for additional crystal and refinement information.

Structure solution and refinement

The space group P 1 21/c 1 was determined based on intensity statistics and systematic absences. The structure was solved using the SHELX suite of programs [3] and refined using full-matrix least-squares on F^2 within the OLEX2 suite.[4] An intrinsic phasing solution was calculated, which provided most non-hydrogen atoms from the E-map. Full-matrix least squares / difference Fourier cycles were performed, which located the remaining non-hydrogen atoms. All non-hydrogen atoms were refined with anisotropic displacement parameters. The hydrogen atoms were placed in ideal positions and refined as riding atoms with relative isotropic displacement parameters. The final full matrix least squares refinement converged to R1 = 0.0435 and wR2 = 0.1094 (F^2, all data). The goodness-of-fit was 1.073. On the basis of the final model, the calculated density was 1.188 g/cm^3 and F(000), 568 e^-.

[1] SAINT, Bruker Analytical X-Ray Systems, Madison, WI, current version.

[2] SADABS, Bruker Analytical X-Ray Systems, Madison, WI, current version.

[3] G. M. Sheldrick, Acta Cryst. A64, 112 - 122 (2008). Sheldrick, G.M. (2015). Acta Cryst. A71, 3-8.

[4] O. V. Dolomanov, L. J. Bourhis, R. J. Gildea, J. A. K. Howard and H. Puschmann, J. Appl. Crystallogr., 2009, 42, 339–341.

Table 1. Crystal data and structure refinement for 20.

Empirical formula	C15 H21 N O3	
Formula weight	263.33	
Crystal color, shape, size	colourless plate, 0.53 × 0.08 × 0.04 mm^3	
Temperature	179.0 K	
Wavelength	0.71073 Å	
Crystal system, space group	Monoclinic, P 1 21/c 1	
Unit cell dimensions	a = 6.0063(4) Å	α = 90°.
	b = 25.4606(18) Å	β = 100.855(3)°.
	c = 9.8048(7) Å	γ = 90°.
Volume	1472.56(18) $Å^3$	
Z	4	
Density (calculated)	1.188 g/cm^3	
Absorption coefficient	0.082 mm^{-1}	
F(000)	568	
**Data collection**		
Diffractometer	Bruker VENTURE D8	
Theta range for data collection	2.261 to 27.511°.	
Index ranges	-7<=h<=7, -33<=k<=33, -12<=l<=12	
Reflections collected	47521	
Independent reflections	3385 [R_{int} = 0.1183]	
Observed Reflections	2623	
Completeness to theta = 25.242°	100.0 %	
**Solution and Refinement**		
Absorption correction	Semi-empirical from equivalents	
Max. and min. transmission	0.7456 and 0.5579	
Solution	Intrinsic methods	
Refinement method	Full-matrix least-squares on F^2	
Weighting scheme	$w = [\sigma^2Fo^2 + AP^2 + BP]^{-1}$, with	
	$P = (Fo^2 + 2 Fc^2)/3$, A = 0.0472, B = 0.1928	
Data / restraints / parameters	3385 / 0 / 176	
Goodness-of-fit on F^2	1.073	
Final R indices [$I>2\sigma(I)$]	R1 = 0.0435, wR2 = 0.1025	
R indices (all data)	R1 = 0.0591, wR2 = 0.1094	
Extinction coefficient	n/a	
Largest diff. peak and hole	0.152 and -0.179 e.$Å^{-3}$	

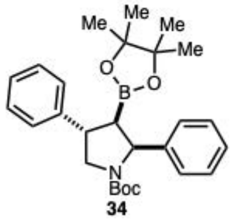

34

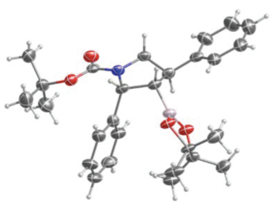

The sample was submitted by Grace Trammel (research group of Kevin Brown, Department of Chemistry, Indiana University). A colorless crystal (approximate dimensions 0.327 × 0.078 × 0.036 mm^3) was placed onto the tip of a MiTeGen loop and mounted on a Bruker Venture D8 diffractometer equipped with a PhotonIII detector at 153(2) K.

Data collection

The data collection was carried out using Mo Kα radiation (graphite monochromator) with a frame time of 120 and 5 seconds and a detector distance of 5.00 cm. A collection strategy was calculated and complete data to a resolution of 0.84 Å with a redundancy of 9.1 were collected. Six major sections of frames were collected with 1º ω and ϕ scans. A total of 1216 frames were collected. The total exposure time was 21.11 hours. The frames were integrated with the Bruker SAINT software package[1] using a narrow-frame algorithm. The integration of the data using a monoclinic unit cell yielded a total of 41120 reflections to a maximum θ angle of 25.03° (0.84 Å resolution), of which 4417 were independent (average redundancy 9.309, completeness = 99.9%, R_{int} = 11.28%, R_{sig} = 7.32%) and 3000 (67.92%) were greater than $2\sigma(F^2)$. The final cell constants of $\underline{a}$ = 6.3954(12) Å, $\underline{b}$ = 24.271(4) Å, $\underline{c}$ = 16.115(3) Å, β = 92.708(5)°, volume = 2498.6(8) $Å^3$, are based upon the refinement of the XYZ-centroids of 5168 reflections above 20 σ(I) with 5.061° < 2θ < 46.59°. Data were corrected for absorption effects using the Multi-Scan method (SADABS[2]). The ratio of minimum to maximum apparent transmission was 0.710. The calculated minimum and maximum transmission coefficients (based on crystal size) are 0.9750 and 0.9970. Please refer to Table 1 for additional crystal and refinement information.

Structure solution and refinement

The space group $P2_1/c$ was determined based on intensity statistics and systematic absences. The structure was solved and refined using the SHELX suite of programs.[3,4] An intrinsic-methods solution was calculated, which provided most non-hydrogen atoms from the E-map. Full-matrix least squares / difference Fourier cycles were performed, which located the remaining non-hydrogen atoms. All non-hydrogen atoms were refined with anisotropic displacement parameters. The hydrogen atoms were placed in ideal positions and refined as riding atoms with relative isotropic displacement parameters. The final anisotropic full-matrix least-squares refinement on F^2 with 305 variables converged at R1 = 10.80%, for the observed data and wR2 = 30.08% for all data. The goodness-of-fit was 1.102. The largest peak in the final difference electron density synthesis was 0.799 $e^-/Å^3$ and the largest hole was -0.295 $e^-/Å^3$ with an RMS deviation of 0.083 $e^-/Å^3$. On the basis of the final model, the calculated density was 1.195 g/cm^3 and F(000), 968 e^-.

1 SAINT V8.40A (2020), Bruker AXS, Madison, WI, 2018.
2 L. Krause, R. Herbst-Irmer, G. M. Sheldrick, D. Stalke:
Comparison of silver and molybdenum microfocus X-ray sources for single-crystal structure determination.
J. Appl. Cryst., 48, 3-10 (2015). doi:10.1107/S1600576714022985.
3 G. M. Sheldrick:
SHELXT--Integrated space-group and crystal-structure determination.
Acta Cryst. A71, 3-8 (2015). doi:10.1107/S2053273314026370.
4 G. M. Sheldrick:
Crystal structure refinement with SHELXL.
Acta Cryst. C71, 3-8 (2015). doi:10.1107/S2053229614024218.

Table 1. Crystal data and structure refinement for 34.

Empirical formula	C27 H36 B N O4	
Formula weight	449.38	
Crystal color, shape, size	colorless needle, 0.327 × 0.078 × 0.036 mm^3	
Temperature	150(2) K	
Wavelength	0.71073 Å	
Crystal system, space group	Monoclinic, $P2_1/c$	
Unit cell dimensions	a = 6.3954(12) Å	α = 90°.
	b = 24.271(4) Å	β = 92.708(5)°.
	c = 16.115(3) Å	γ = 90°.
Volume	2498.7(8) $Å^3$	
Z	4	
Density (calculated)	1.195 Mg/m^3	
Absorption coefficient	0.078 mm^{-1}	
F(000)	968	
Data collection		
Diffractometer	Venture D8, Bruker	
Source	Iμ3.0, Incoatec	
Detector	Photon III	
Theta range for data collection	2.102 to 25.032°.	
Index ranges	-7<=h<=7, -28<=k<=28, -19<=l<=19	
Reflections collected	41120	
Independent reflections	4417 [R_{int} = 0.1128]	
Observed Reflections	3000	
Completeness to theta = 25.032°	99.9 %	
Solution and Refinement		
Absorption correction	Semi-empirical from equivalents	
Max. and min. transmission	0.7452 and 0.5290	
Solution	Intrinsic methods	
Refinement method	Full-matrix least-squares on F^2	
Weighting scheme	$w = [\sigma^2Fo^2 + AP^2 + BP]^{-1}$, with	
	$P = (Fo^2 + 2 Fc^2)/3$, A = 0.1014, B = 9.4738	
Data / restraints / parameters	4417 / 0 / 305	
Goodness-of-fit on F^2	1.102	
Final R indices [I>2σ(I)]	R1 = 0.1080, wR2 = 0.2789	
R indices (all data)	R1 = 0.1448, wR2 = 0.3008	
Largest diff. peak and hole	0.799 and -0.295 e.$Å^{-3}$	

Goodness-of-fit = $[\Sigma[w(F_o^2 - F_c^2)^2]/N_{observns} - N_{params})]^{1/2}$, all data.

R1 = $\Sigma(|F_o| - |F_c|) / \Sigma |F_o|$. $wR2 = [\Sigma[w(F_o^2 - F_c^2)^2] / \Sigma [w(F_o^2)^2]]^{1/2}$.

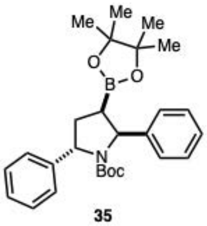

35

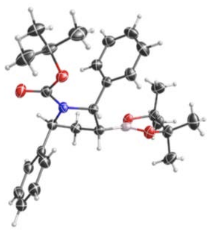

The sample was submitted by Grace Trammel (research group of Kevin Brown, Department of Chemistry, Indiana University). A colorless crystal (approximate dimensions 0.271 × 0.133 × 0.130 mm^3) was placed onto the tip of a MiTeGen loop and mounted on a Bruker Venture D8 diffractometer equipped with a PhotonIII detector at 153(2) K.

Data collection

The data collection was carried out using Mo $K\alpha$ radiation (graphite monochromator) with a frame time of 1 seconds and a detector distance of 5.00 cm. A collection strategy was calculated and complete data to a resolution of 0.77 Å with a redundancy of 13.5 were collected. Eighteen major sections of frames were collected with 1º ω and ϕ scans. A total of 2740 frames were collected. The total exposure time was 0.88 hours. The frames were integrated with the Bruker SAINT software package[1] using a narrow-frame algorithm. The integration of the data using a triclinic unit cell yielded a total of 90511 reflections to a maximum θ angle of 26.41° (0.80 Å resolution), of which 5302 were independent (average redundancy 17.071, completeness = 99.9%, R_{int} = 12.41%, R_{sig} = 4.22%) and 3505 (66.11%) were greater than $2\sigma(F^2)$. The final cell constants of a = 10.2678(13) Å, b = 11.4790(14) Å, c = 11.6940(13) Å, α = 79.962(4)°, β = 71.850(4)°, γ = 86.356(4)°, volume = 1289.6(3) $Å^3$, are based upon the refinement of the XYZ-centroids of 9988 reflections above 20 σ(I) with 4.735° < 2θ < 51.52°. Data were corrected for absorption effects using the Multi-Scan method (SADABS[2]). The ratio of minimum to maximum apparent transmission was 0.935. The calculated minimum and maximum transmission coefficients (based on crystal size) are 0.9800 and 0.9900. Please refer to Table 1 for additional crystal and refinement information.

Structure solution and refinement

The space group P-1 was determined based on intensity statistics and the lack of systematic absences. The structure was solved and refined using the SHELX suite of programs.[3,4] An intrinsic-methods solution was calculated, which provided most non-hydrogen atoms from the E-map. Full-matrix least squares / difference Fourier cycles were performed, which located the remaining non-hydrogen atoms. All non-hydrogen atoms were refined with anisotropic displacement parameters. The hydrogen atoms were placed in ideal positions and refined as riding atoms with relative isotropic displacement parameters. The final anisotropic full-matrix least-squares refinement on F^2 with 305 variables converged at R1 = 4.13%, for the observed data and wR2 = 11.10% for all data. The goodness-of-fit was 1.039. The largest peak in the final difference electron density synthesis was 0.181 $e^-/Å^3$ and the largest hole was -0.196 $e^-/Å^3$ with an RMS deviation of 0.039 $e^-/Å^3$. On the basis of the final model, the calculated density was 1.157 g/cm^3 and F(000), 484 e^-.

1 SAINT V8.40A (2020), Bruker AXS, Madison, WI, 2018.
2 L. Krause, R. Herbst-Irmer, G. M. Sheldrick, D. Stalke:
Comparison of silver and molybdenum microfocus X-ray sources for single-crystal structure determination.
J. Appl. Cryst., 48, 3-10 (2015). doi:10.1107/S1600576714022985.
3 G. M. Sheldrick:
SHELXT--Integrated space-group and crystal-structure determination.
Acta Cryst. A71, 3-8 (2015). doi:10.1107/S2053273314026370.
4 G. M. Sheldrick:
Crystal structure refinement with SHELXL.
Acta Cryst. C71, 3-8 (2015). doi:10.1107/S2053229614024218.

Table 1. Crystal data and structure refinement for 35.

Empirical formula	C27 H36 B N O4	
Formula weight	449.38	
Crystal color, shape, size	colourless block, 0.271 × 0.133 × 0.130 mm^3	
Temperature	153(2) K	
Wavelength	0.71073 Å	
Crystal system, space group	Triclinic, P-1	
Unit cell dimensions	a = 10.2678(13) Å	α = 79.962(4)°.
	b = 11.4790(14) Å	β = 71.850(4)°.
	c = 11.6940(13) Å	γ = 86.356(4)°.
Volume	1289.6(3) $Å^3$	
Z	2	
Density (calculated)	1.157 Mg/m^3	
Absorption coefficient	0.076 mm^{-1}	
F(000)	484	
Data collection		
Diffractometer	Venture D8, Bruker	
Source	IμS 3.0, Incoatec	
Detector	Photon III	
Theta range for data collection	1.857 to 26.415°.	
Index ranges	-12<=h<=12, -14<=k<=14, -14<=l<=14	
Reflections collected	90511	
Independent reflections	5302 [R_{int} = 0.1241]	
Observed Reflections	3505	
Completeness to theta = 25.242°	100.0 %	
Solution and Refinement		
Absorption correction	Semi-empirical from equivalents	
Max. and min. transmission	0.7454 and 0.6968	
Solution	Intrinsic methods	
Refinement method	Full-matrix least-squares on F^2	
Weighting scheme	w = $[\sigma^2Fo^2+ AP^2+ BP]^{-1}$, with	
	P = $(Fo^2+ 2 Fc^2)/3$, A = 0.0449, B = 0.2640	
Data / restraints / parameters	5302 / 0 / 305	
Goodness-of-fit on F^2	1.039	
Final R indices [I>2σ(I)]	R1 = 0.0413, wR2 = 0.0967	
R indices (all data)	R1 = 0.0757, wR2 = 0.1110	
Largest diff. peak and hole	0.181 and -0.196 e.$Å^{-3}$	

Goodness-of-fit = $[\Sigma[w(F_o^2 - F_c^2)^2]/N_{observns} - N_{params})]^{1/2}$, all data.

R1 = $\Sigma(|F_o| - |F_c|) / \Sigma |F_o|$. $wR2 = [\Sigma[w(F_o^2 - F_c^2)^2] / \Sigma [w(F_o^2)^2]]^{1/2}$.

The sample was submitted by Grace Trammel (research group of Professor Kevin Brown, Department of Chemistry, Indiana University). Single crystals suitable for X-ray diffraction were grown by slow diffusion from diethyl ether in a pentane solution. A colourless crystal (Figure 1, needle, approximate dimensions 0.3 × 0.08 × 0.05 mm^3) was placed onto the tip of a MiTeGen pin and mounted on a Bruker Venture D8 diffractometer equipped with a PhotonIII detector at 173.0 K.

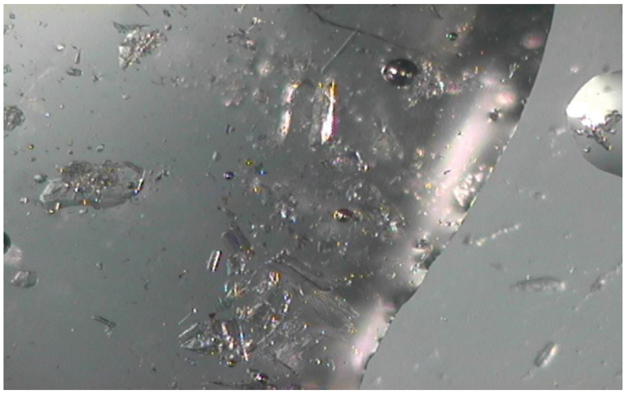

Figure 1. Mycroscope image of bulk material.

Data collection

The data collection was carried out using Mo Kα radiation (λ = 0.71073 Å, graphite monochromator) with a frame time of 2 seconds and a detector distance of 40 mm. A collection strategy was calculated and complete data to a resolution of 0.80 Å with a redundancy of 2 were collected. The frames were integrated with the Bruker SAINT[1]software package using a narrow-frame algorithm. The integration of the data using a monoclinic unit cell yielded a total of 51902 reflections to a maximum θ angle of 26.39° (0.80 Å resolution), of which 4576 were independent (average redundancy 11.342, completeness = 99.6%, Rint = 13.13%, Rsig = 4.81%) and 3086 (67.44%) were greater than $2\sigma(F^2)$. The final cell constants of a = 6.4920(2) Å, b = 20.6253(9) Å, c = 16.9512(8) Å, β = 98.739(2)°, volume = 2243.41(16) $Å^3$, are based upon the refinement of the XYZ-centroids of reflections above 20 σ(I). Data were corrected for absorption effects using the Multi-Scan method (SADABS).[2] The calculated minimum and maximum transmission coefficients (based on crystal size) are 0.9770 and 0.9960. Please refer to Table 1 for additional crystal and refinement information.

Structure solution and refinement

The space group P 1 21/c 1 was determined based on intensity statistics and systematic absences. The structure was solved using the SHELX suite of programs [3] and refined using full-matrix least-squares on F^2 within the OLEX2 suite.[4] An intrinsic phasing solution was calculated, which provided most non-hydrogen atoms from the E-map. Full-matrix least squares / difference Fourier cycles were performed, which located the remaining non-hydrogen atoms. All non-hydrogen atoms were refined with anisotropic displacement parameters. The hydrogen atoms were placed in ideal positions and refined as riding atoms with relative isotropic displacement parameters. The

final full matrix least squares refinement converged to R1 = 0.0494 and wR2 = 0.1387 (F^2, all data). The goodness-of-fit was 1.068. On the basis of the final model, the calculated density was 1.147 g/cm^3 and F(000), 840 e^-.

[1]SAINT, V8.30A, Bruker Analytical X-Ray Systems, Madison, WI, 2012.

[2]SADABS, 2.03, Bruker Analytical X-Ray Systems, Madison, WI, 2016.

[3]G. M. Sheldrick, Acta Cryst. A64, 112 - 122 (2008). Sheldrick, G.M. (2015). Acta Cryst. A71, 3-8.

[4]O. V. Dolomanov, L. J. Bourhis, R. J. Gildea, J. A. K. Howard and H. Puschmann, J. Appl. Crystallogr., 2009, 42, 339–341.

Structure description

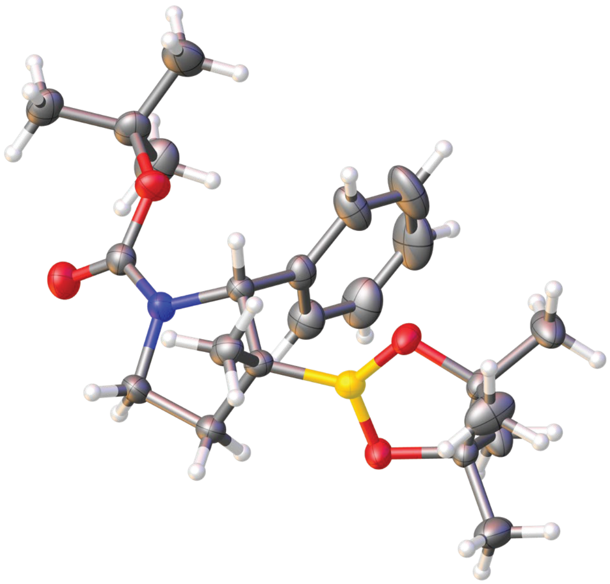

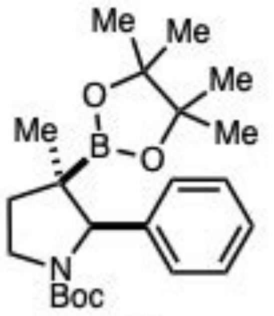

36

Table 1. Crystal data and structure refinement for 36.

Empirical formula	C22 H34 B N O4	
Formula weight	387.31	
Crystal color, shape, size	colourless needle, 0.3 × 0.08 × 0.05 mm^3	
Temperature	173.0 K	
Wavelength	0.71073 Å	
Crystal system, space group	Monoclinic, P 1 21/c 1	
Unit cell dimensions	a = 6.4920(2) Å	α = 90°.
	b = 20.6253(9) Å	β = 98.739(2)°.
	c = 16.9512(8) Å	γ = 90°.
Volume	2243.41(16) $Å^3$	
Z	4	
Density (calculated)	1.147 g/cm^3	
Absorption coefficient	0.077 mm^{-1}	
F(000)	840	
**Data collection**		
Diffractometer	Bruker Venture D8	
Theta range for data collection	2.431 to 26.394°.	
Index ranges	-8<=h<=8, -25<=k<=25, -21<=l<=21	
Reflections collected	51902	
Independent reflections	4576 [R_{int} = 0.1313]	
Observed Reflections	3086	
Completeness to theta = 25.242°	99.7 %	
**Solution and Refinement**		
Absorption correction	Semi-empirical from equivalents	
Max. and min. transmission	0.7405 and 0.6885	
Solution	Intrinsic methods	
Refinement method	Full-matrix least-squares on F^2	
Weighting scheme	$w = [\sigma^2Fo^2 + AP^2 + BP]^{-1}$, with	
	$P = (Fo^2 + 2 Fc^2)/3$, A = 0.0411, B = 1.3368	
Data / restraints / parameters	4576 / 0 / 261	
Goodness-of-fit on F^2	1.068	
Final R indices [$I>2\sigma(I)$]	R1 = 0.0494, wR2 = 0.1085	
R indices (all data)	R1 = 0.0839, wR2 = 0.1387	
Extinction coefficient	n/a	
Largest diff. peak and hole	0.342 and -0.232 e.$Å^{-3}$	

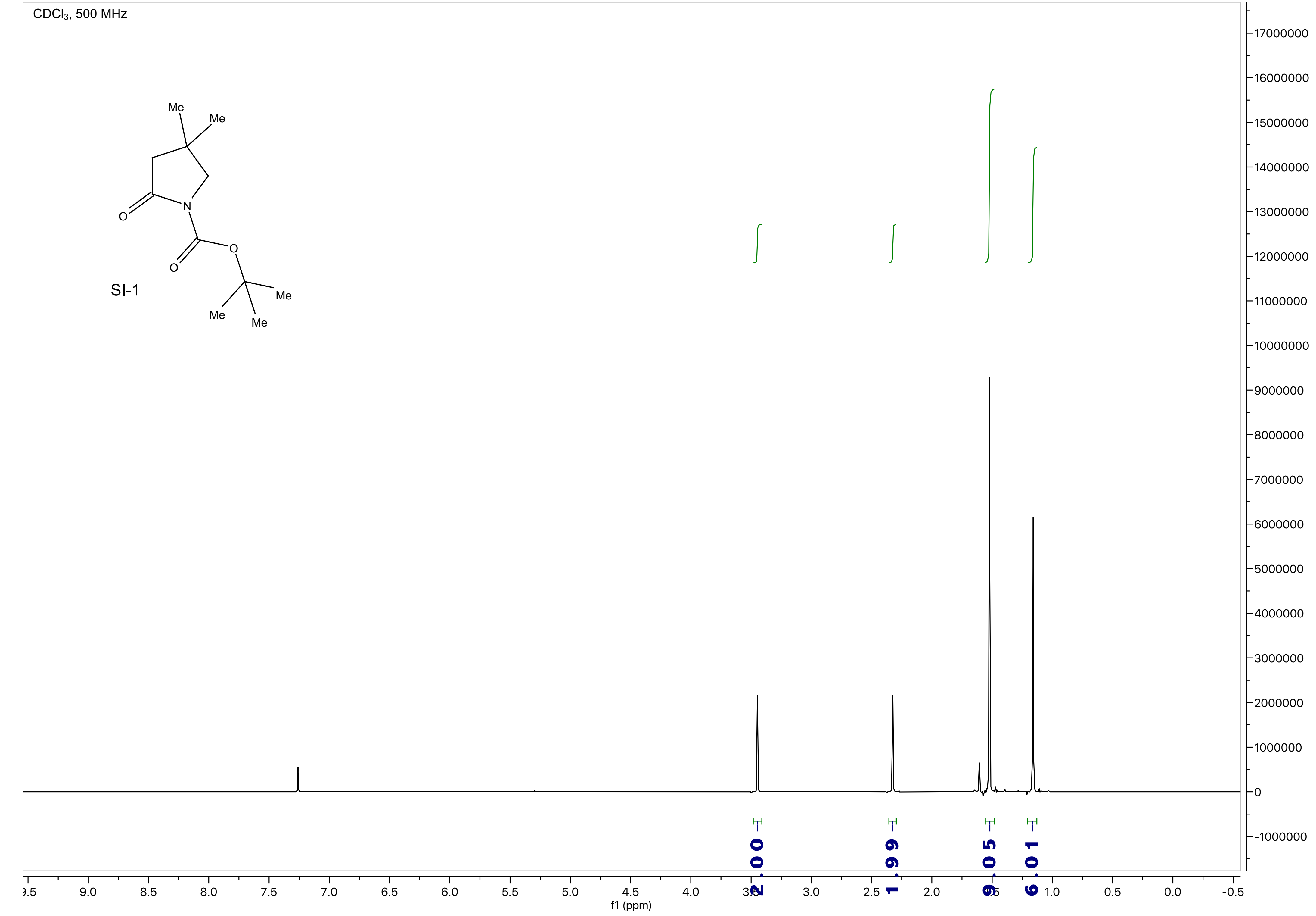
CDCl3, 500 MHz
SI-1
Me
Me
N
O
O
O
Me
Me
Me
2.00
1.99
9.05
6.01
f1 (ppm)
9.0
8.5
8.0
7.5
7.0
6.5
6.0
5.5
5.0
4.5
4.0
3.0
2.5
2.0
1.0
0.5
0.0
-0.5
17000000
16000000
15000000
14000000
13000000
12000000
11000000
10000000
9000000
8000000
7000000
6000000
5000000
4000000
3000000
2000000
1000000
0
-1000000

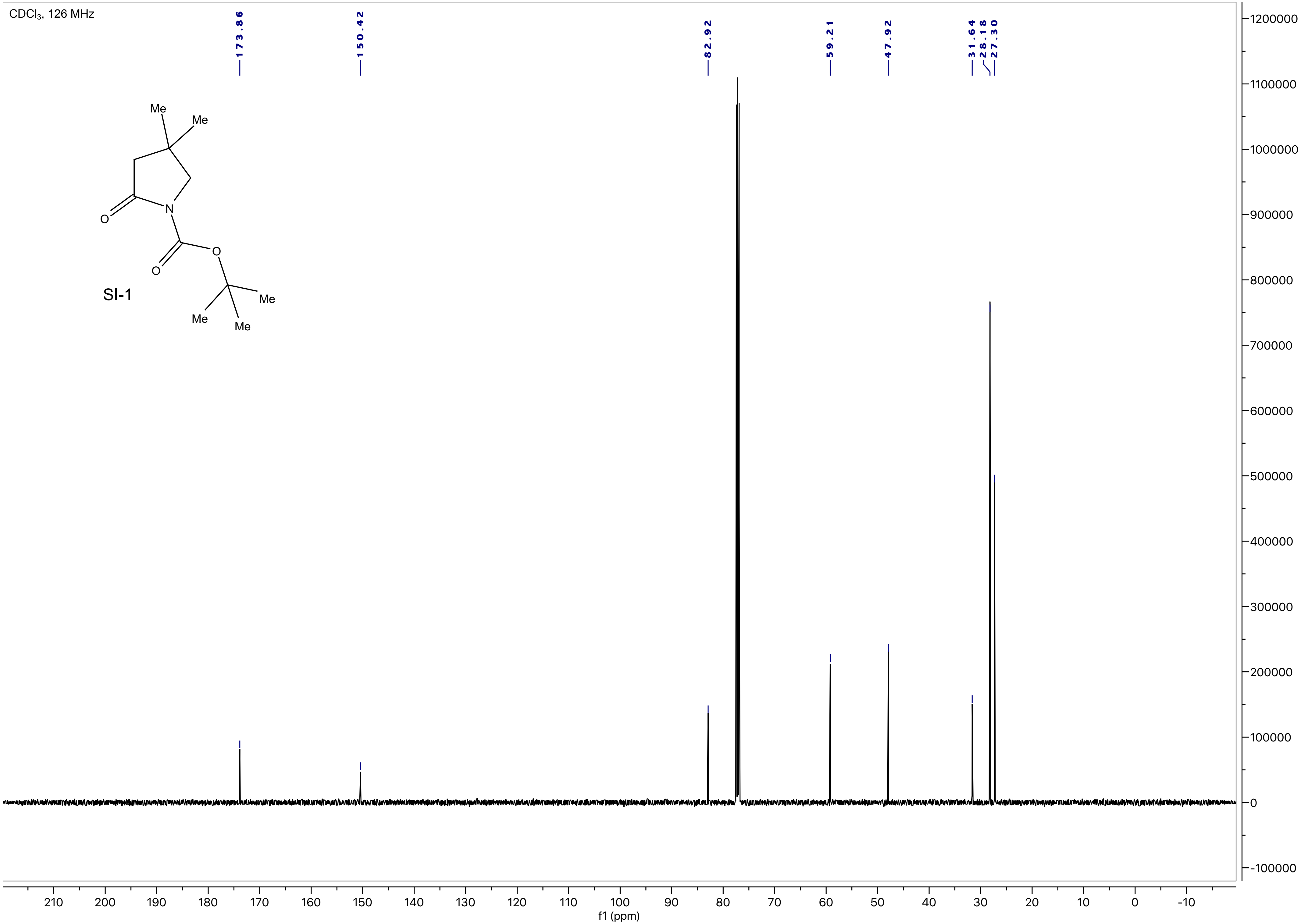

CDCl3, 126 MHz
173.86
150.42
82.92
59.21
47.92
31.64
28.18
27.30
Me
Me
N
O
O
O
Me
Me
Me
SI-1
f1 (ppm)

CDCl$_3$, 500 MHz, 1.3:1 mixture of rotamers

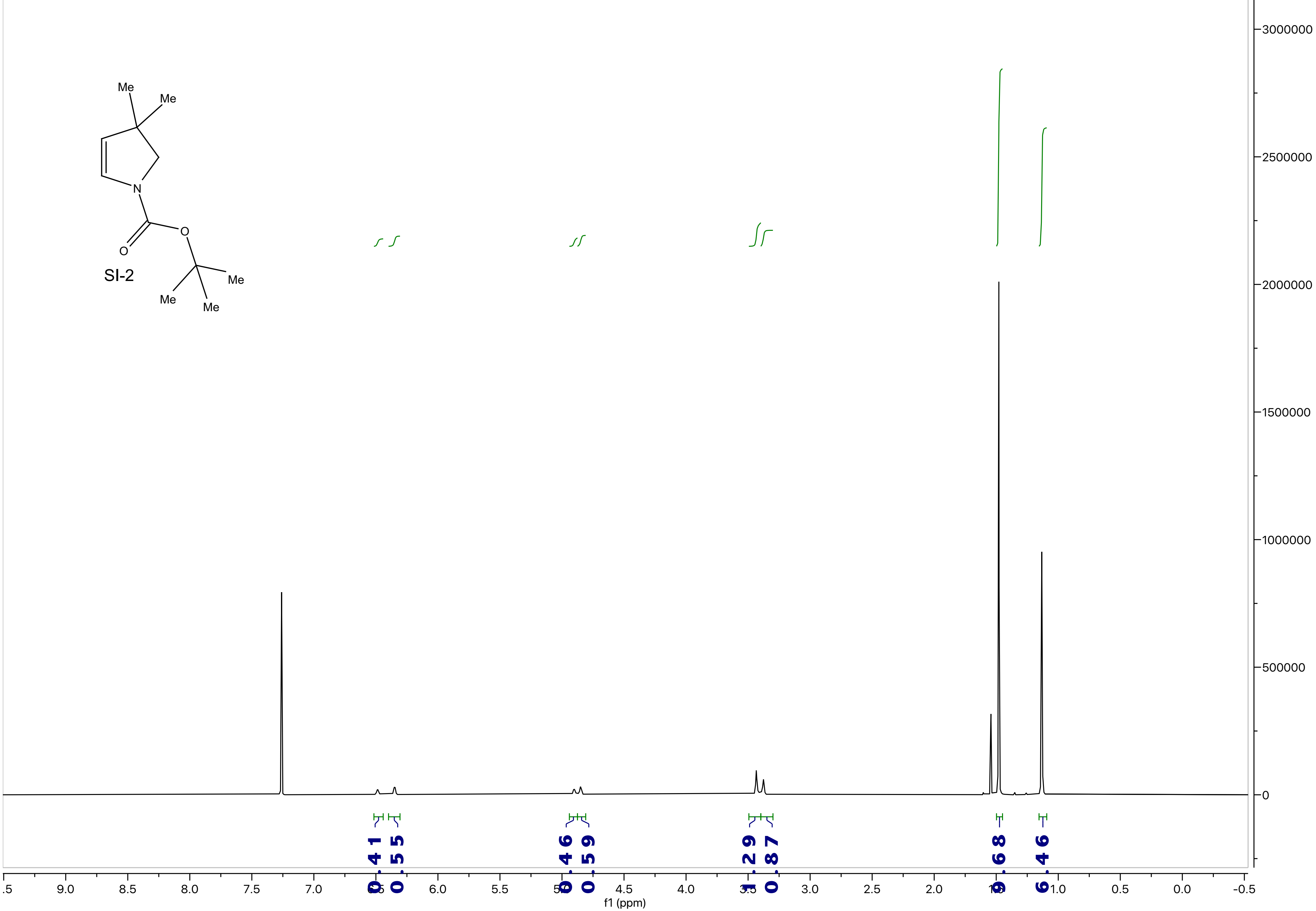

CDCl$_3$, 126 MHz, mixture of rotamers

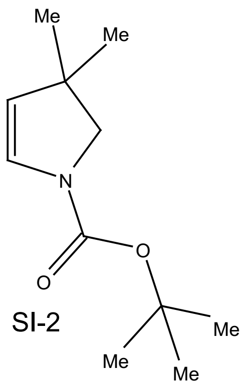

SI-2

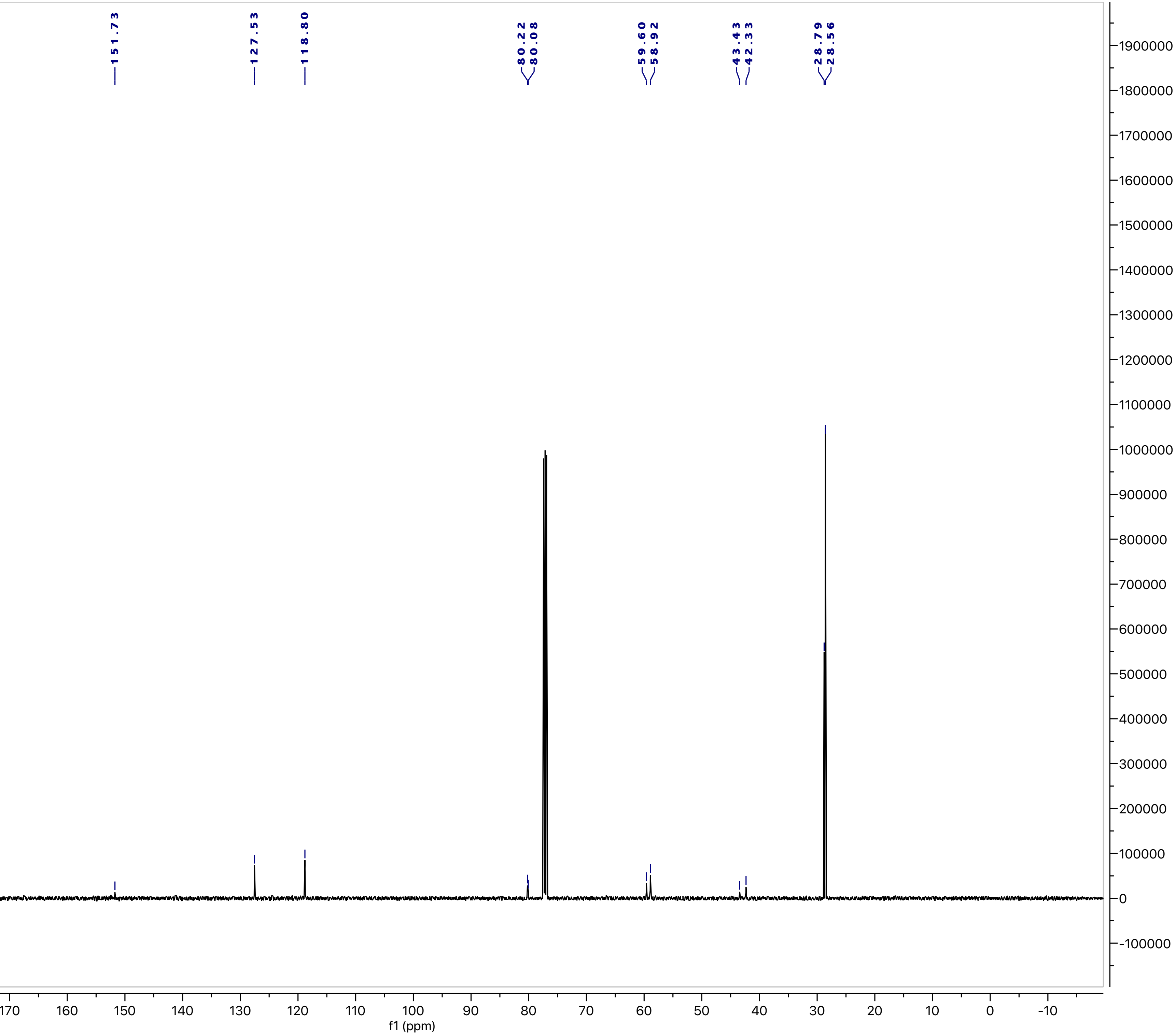

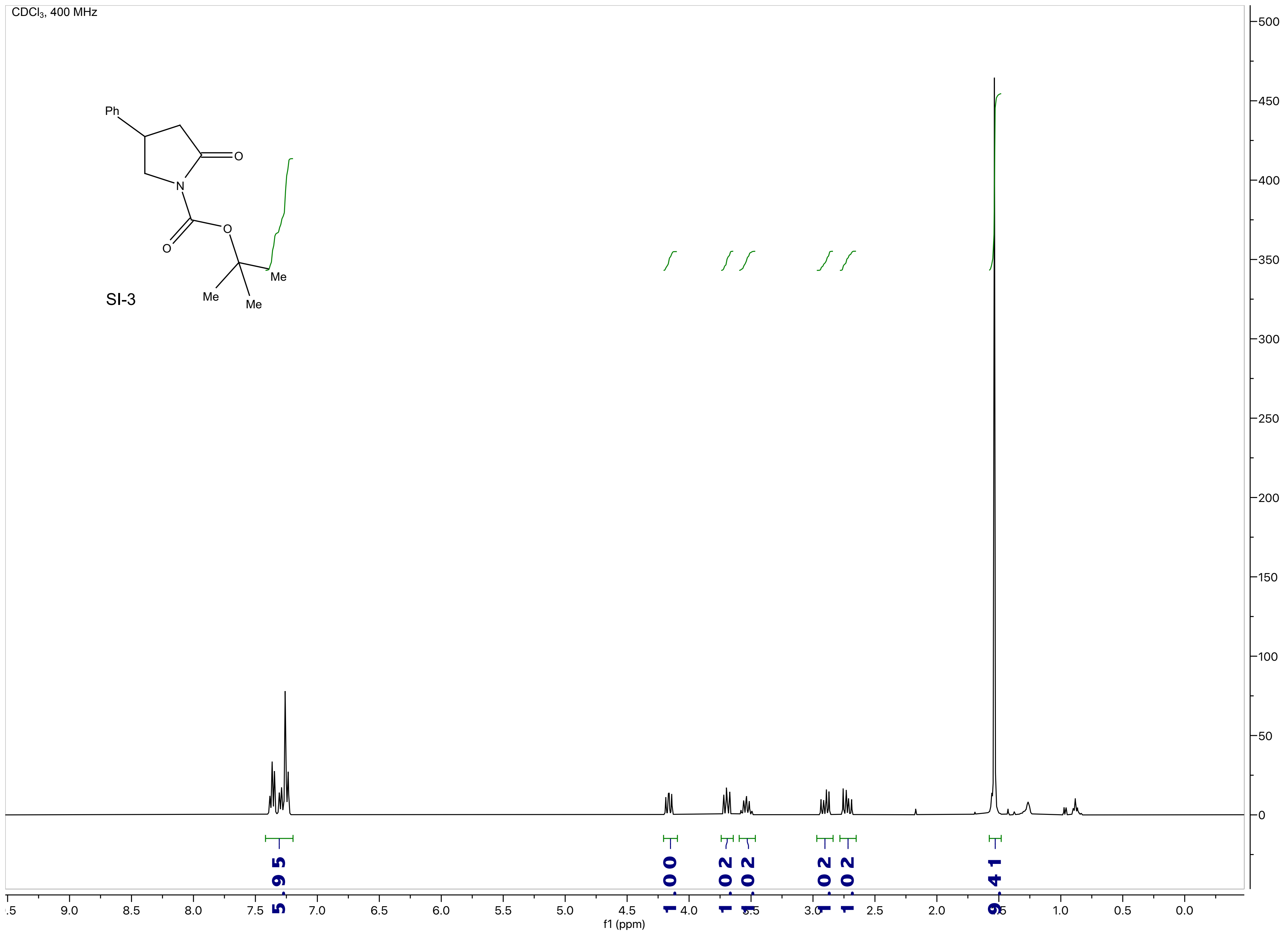

CDCl3, 400 MHz
Ph
O
N
O
O
Me
Me
Me
SI-3
5.95
1.00
1.02
1.02
1.02
1.02
9.41
f1 (ppm)

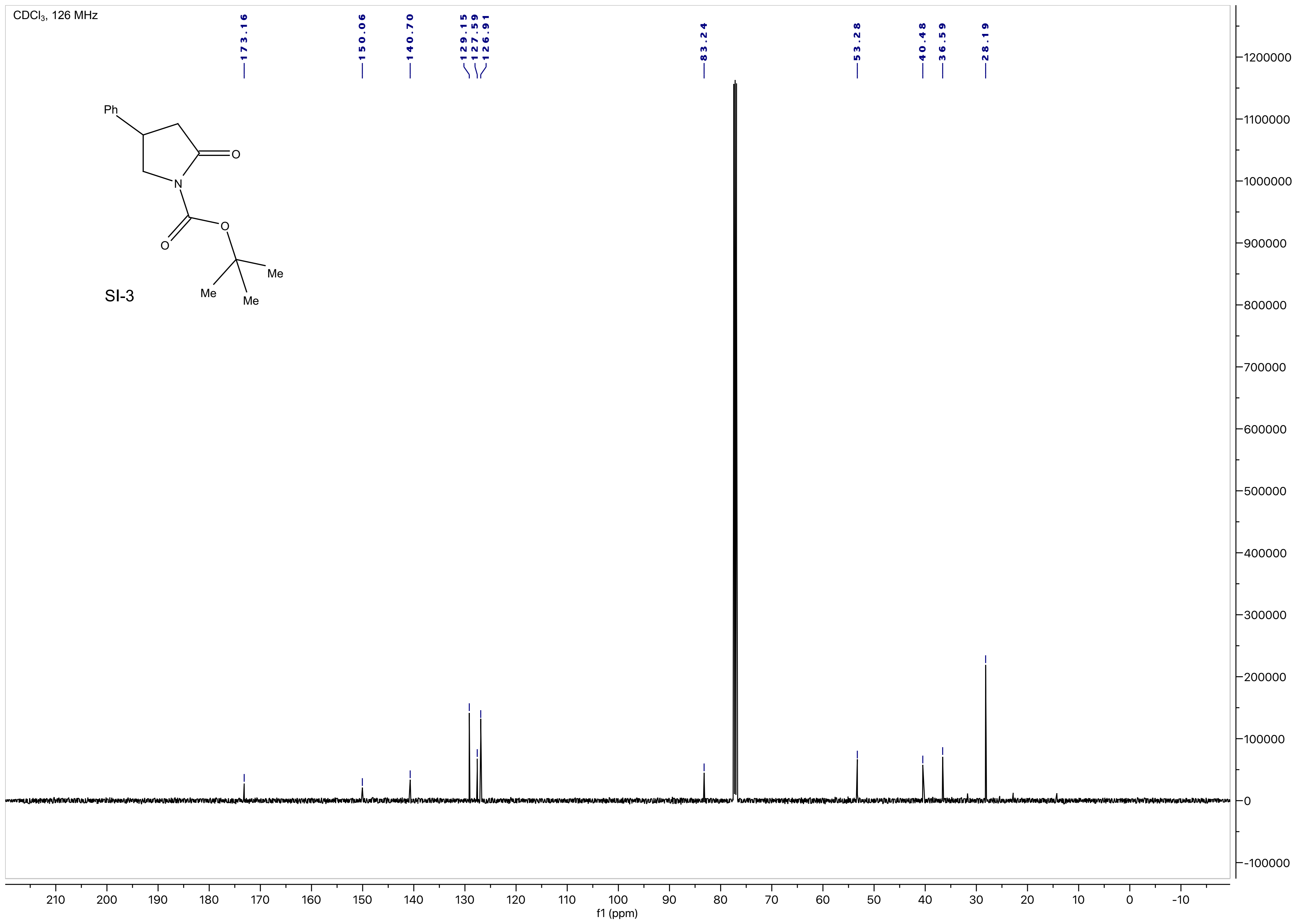

CDCl3, 126 MHz
173.16
150.06
140.70
129.15
127.59
126.91
83.24
53.28
40.48
36.59
28.19
Ph
O
N
O
O
Me
Me
Me
SI-3
f1 (ppm)

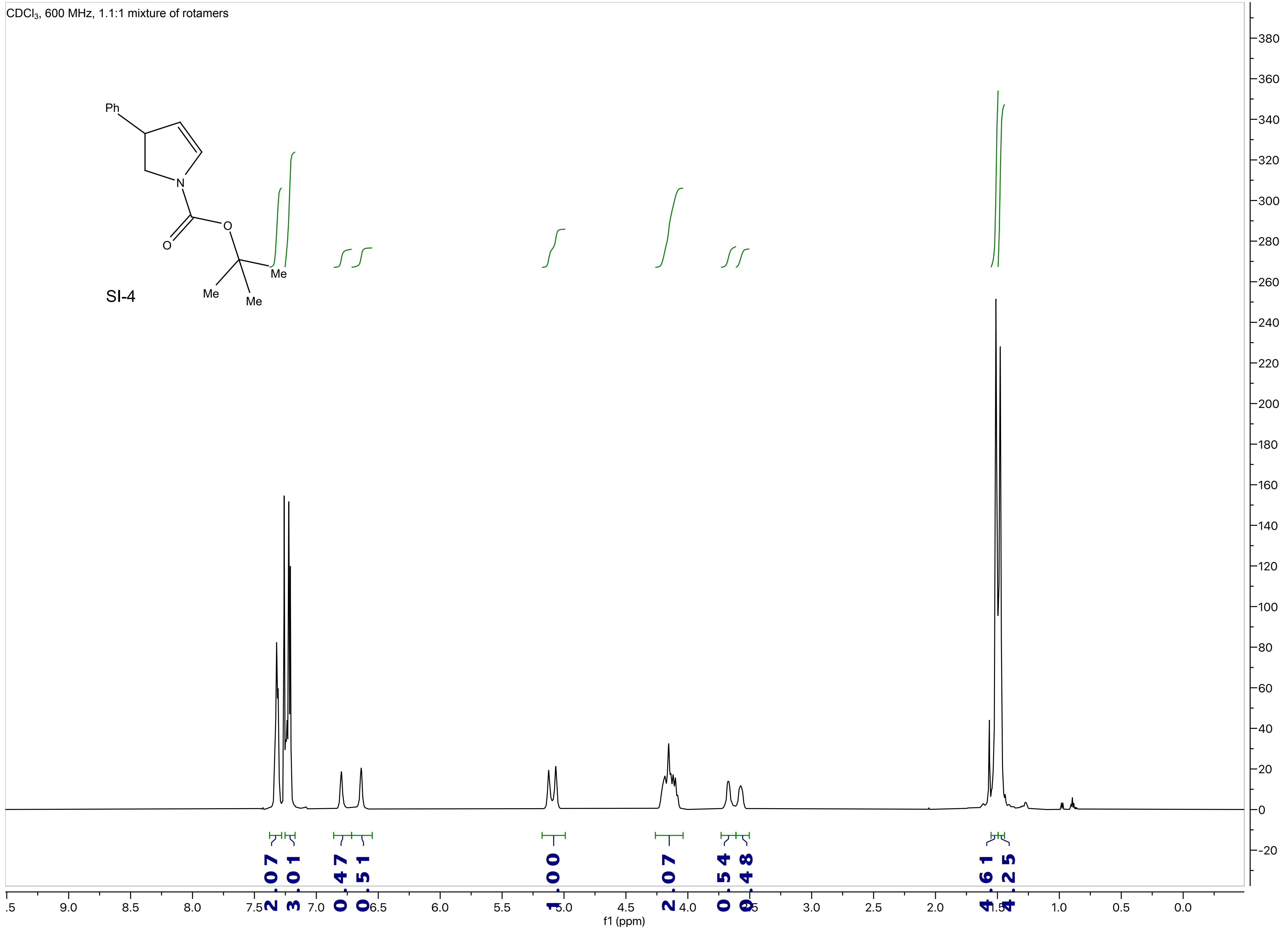
CDCl3, 600 MHz, 1.1:1 mixture of rotamers
Ph
N
O
O
Me
Me
Me
SI-4
2.07
3.01
0.47
0.51
1.00
2.07
0.54
0.48
4.61
4.25
f1 (ppm)

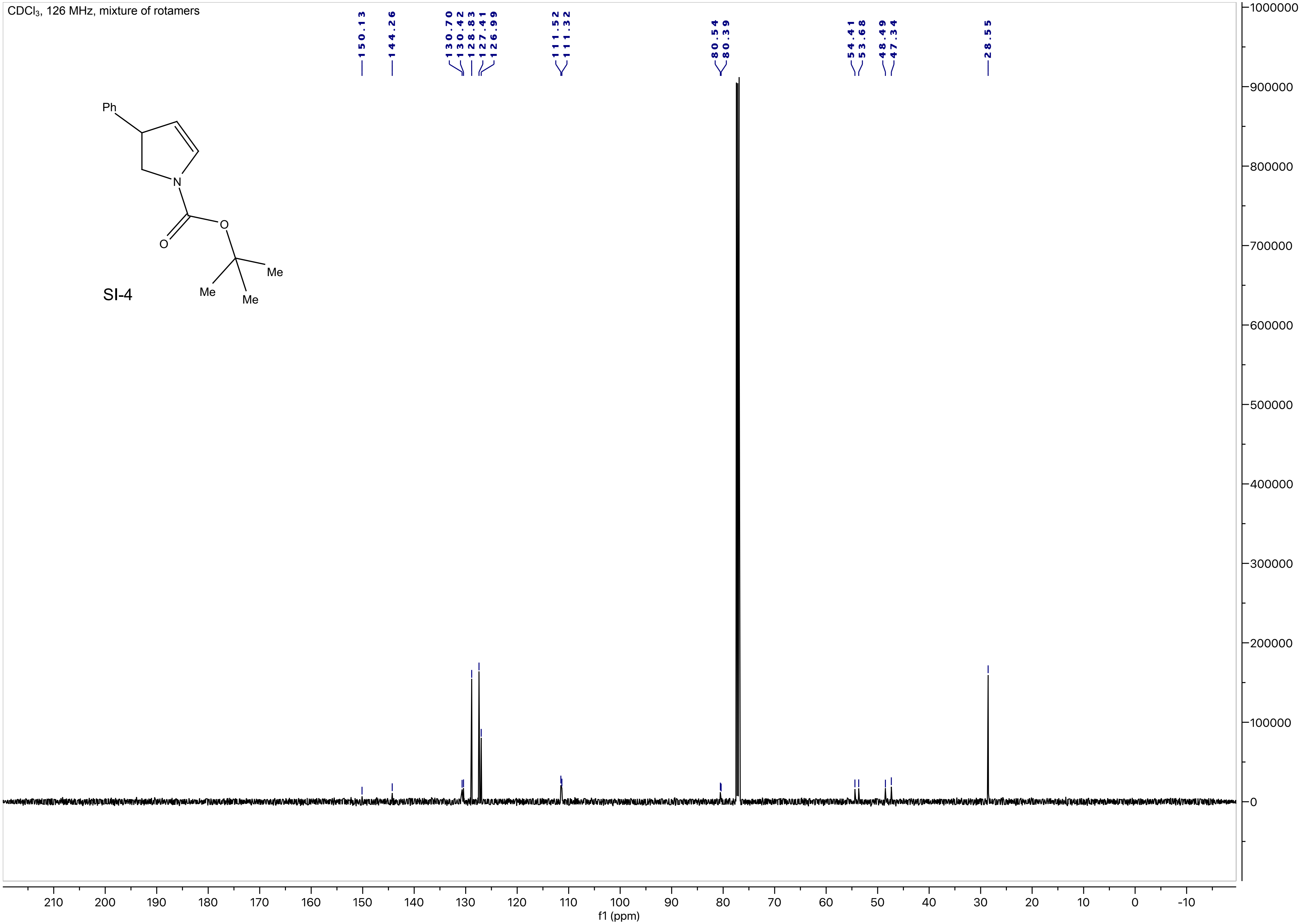
CDCl$_3$, 126 MHz, mixture of rotamers
150.13
144.26
130.70
130.42
128.83
127.41
126.99
111.52
111.32
80.54
80.39
54.41
53.68
48.49
47.34
28.55
Ph
N
O
O
Me
Me
Me
SI-4
1000000
900000
800000
700000
600000
500000
400000
300000
200000
100000
0
210 200 190 180 170 160 150 140 130 120 110 100 90 80 70 60 50 40 30 20 10 0 -10
f1 (ppm)

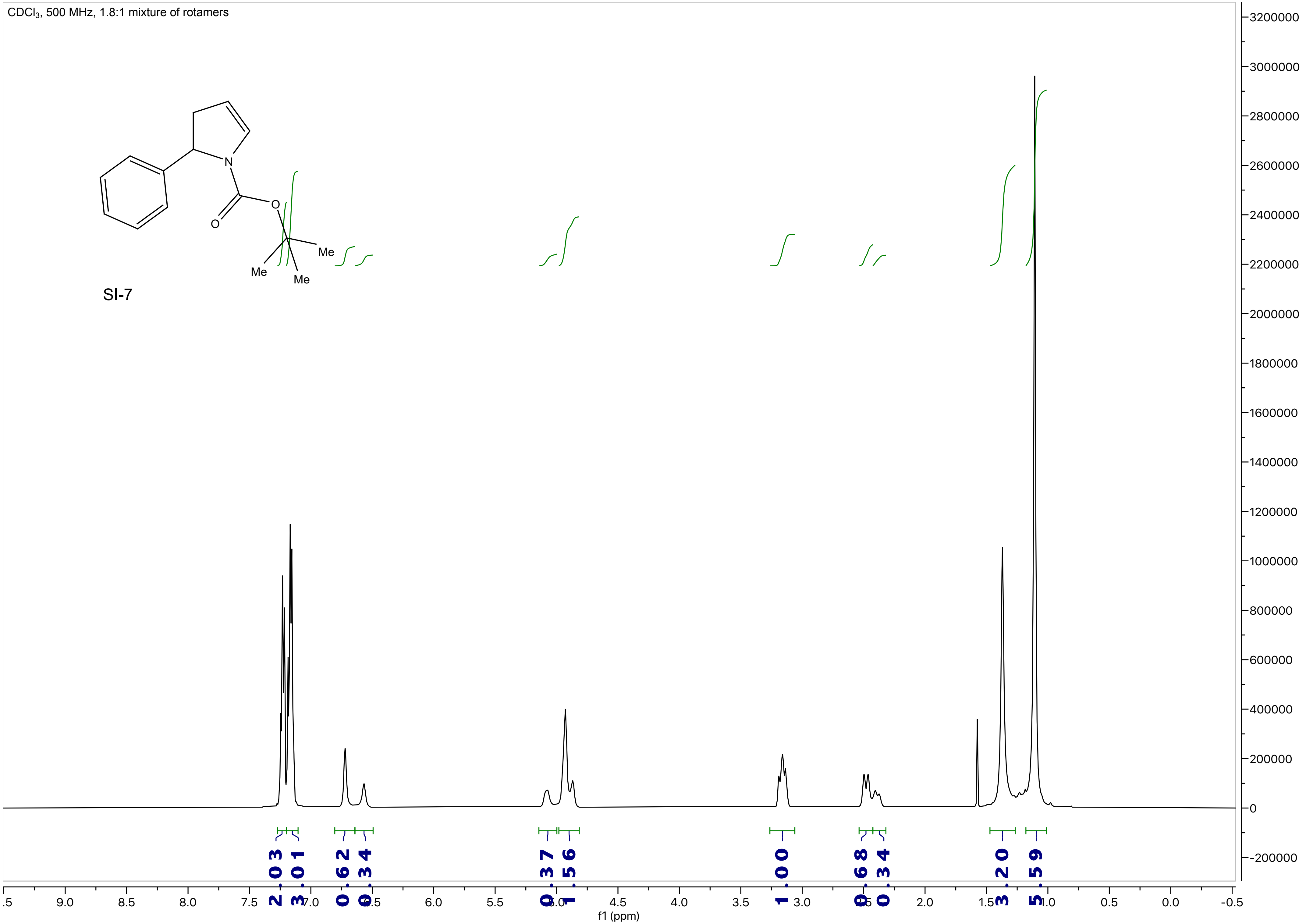
CDCl3, 500 MHz, 1.8:1 mixture of rotamers
SI-7
f1 (ppm)

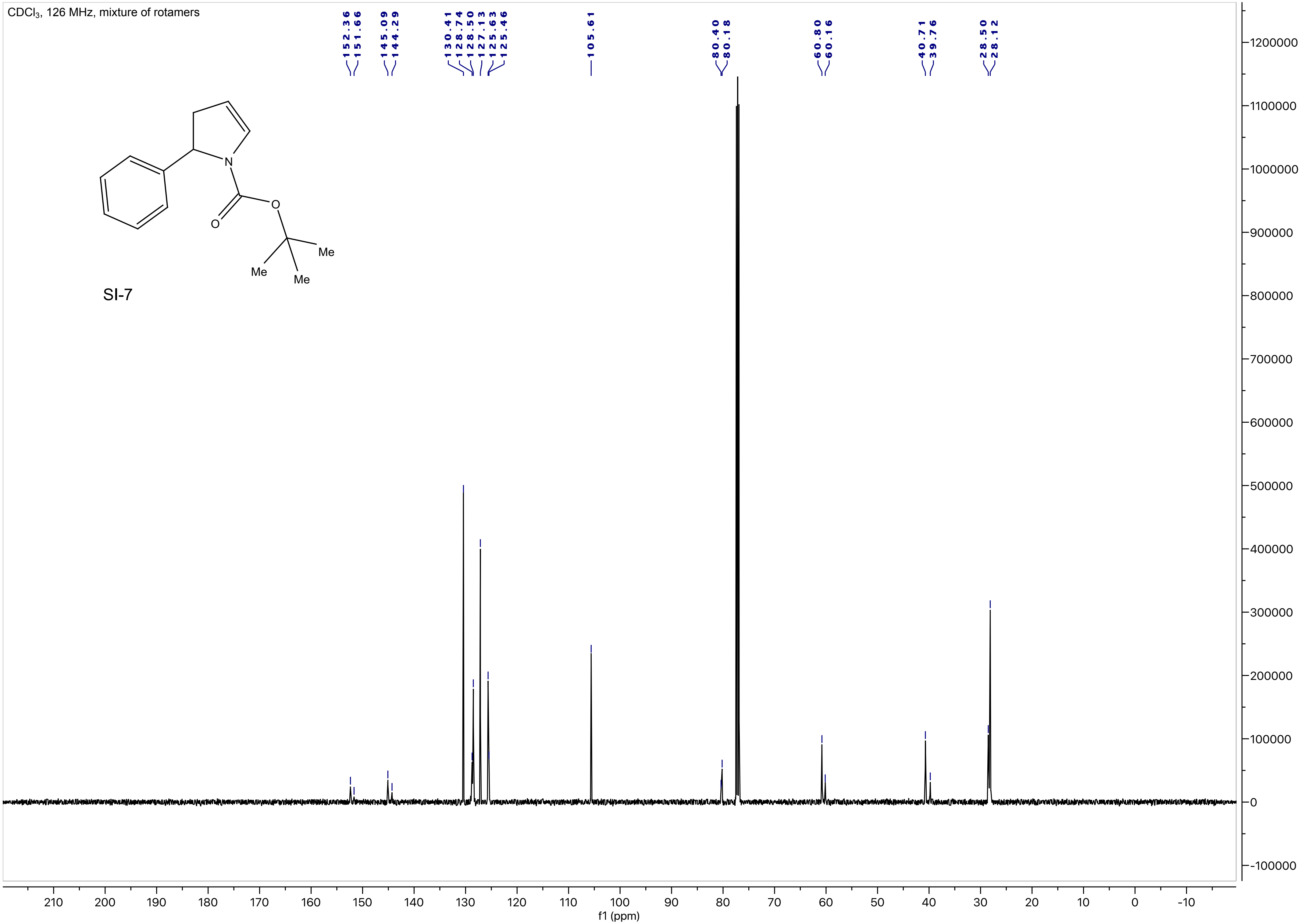
CDCl$_3$, 126 MHz, mixture of rotamers
152.36
151.66
145.09
144.29
130.41
128.74
128.50
127.13
125.63
125.46
105.61
80.40
80.18
60.80
60.16
40.71
39.76
28.50
28.12
N
O
O
Me
Me
Me
SI-7
f1 (ppm)

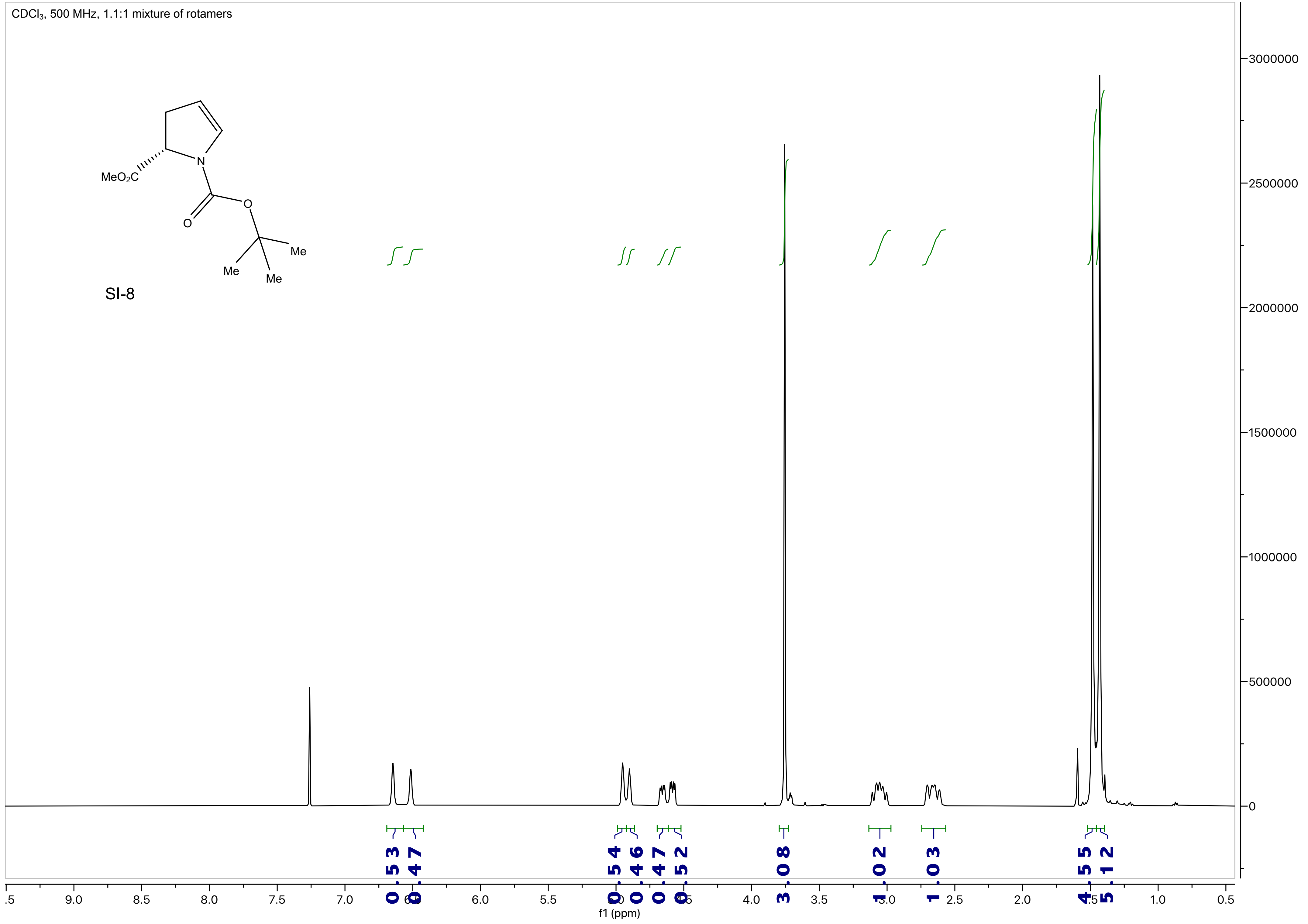
CDCl3, 500 MHz, 1.1:1 mixture of rotamers
MeO2C
N
O
O
Me
Me
Me
SI-8
0.53
0.47
0.54
0.46
0.47
0.52
3.08
1.02
1.03
4.55
5.12
f1 (ppm)

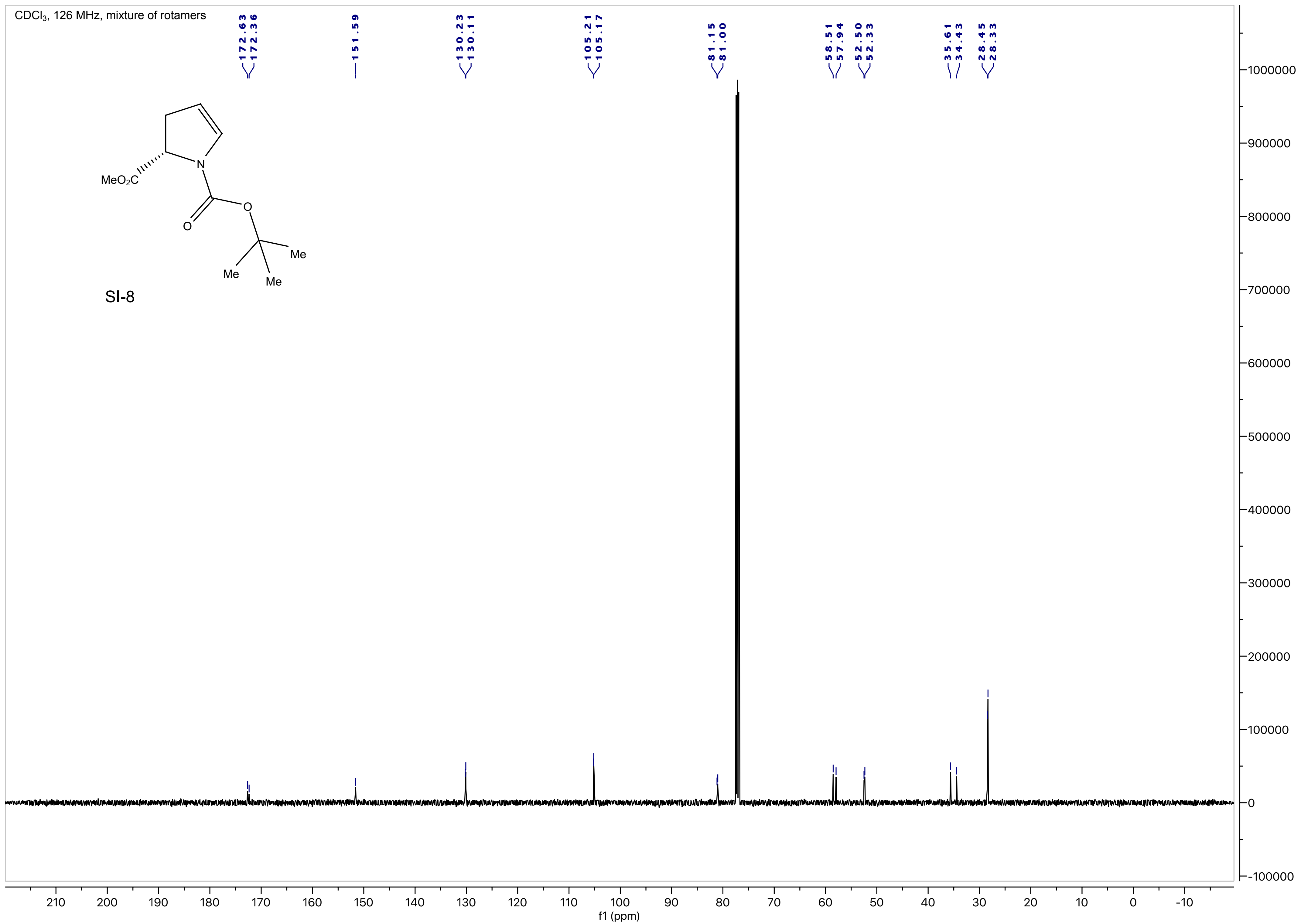

CDCl3, 126 MHz, mixture of rotamers
172.63
172.36
151.59
130.23
130.11
105.21
105.17
81.15
81.00
58.51
57.94
52.50
52.33
35.61
34.43
28.45
28.33
MeO2C
N
O
O
Me
Me
Me
SI-8
f1 (ppm)

CDCl3, 500 MHz, mixture of rotamers

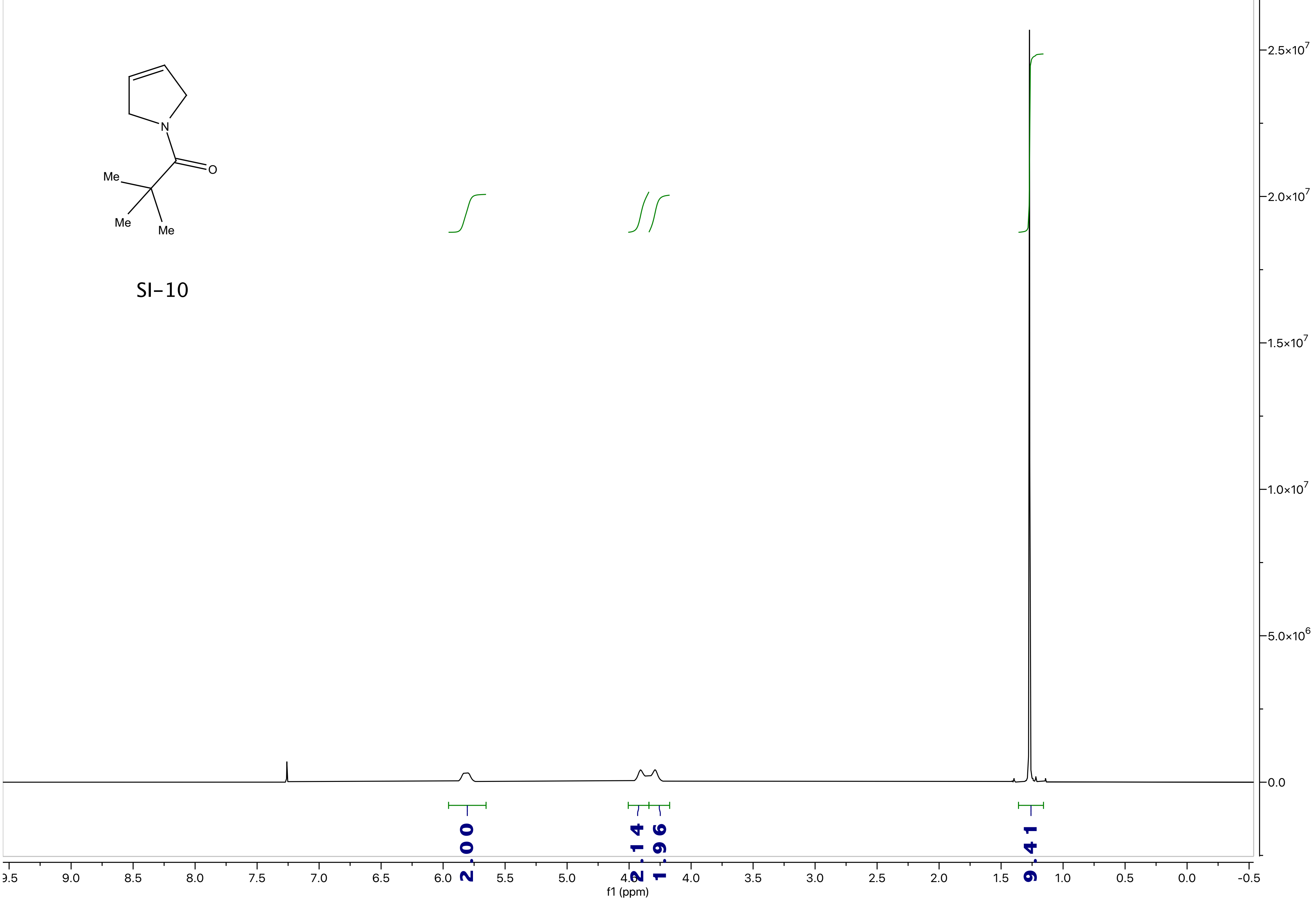

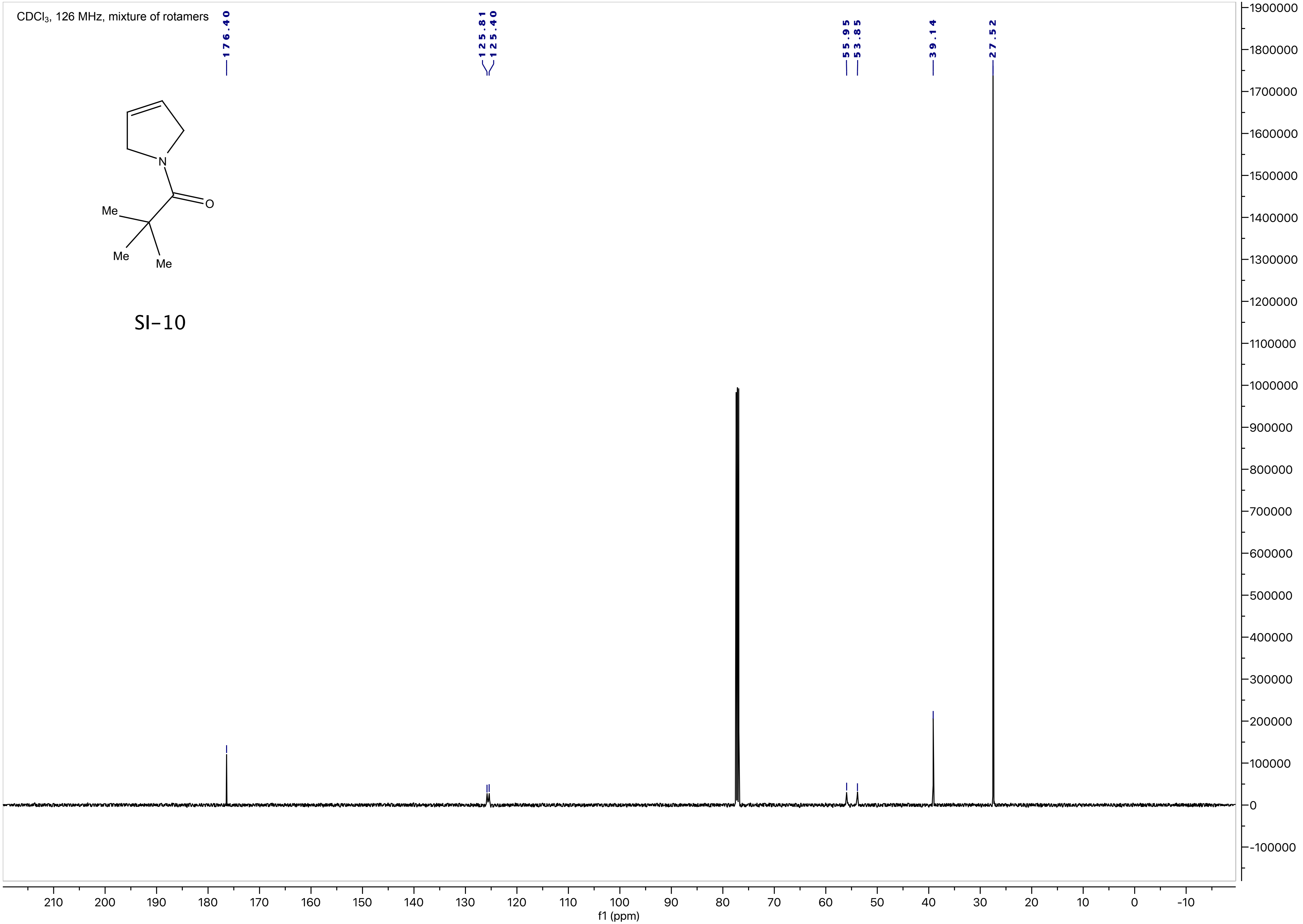
CDCl$_3$, 126 MHz, mixture of rotamers
176.40
125.81
125.40
55.95
53.85
39.14
27.52
N
O
Me
Me
Me
SI-10
f1 (ppm)
210
200
190
180
170
160
150
140
130
120
110
100
90
80
70
60
50
40
30
20
10
0
-10
1900000
1800000
1700000
1600000
1500000
1400000
1300000
1200000
1100000
1000000
900000
800000
700000
600000
500000
400000
300000
200000
100000
0
-100000

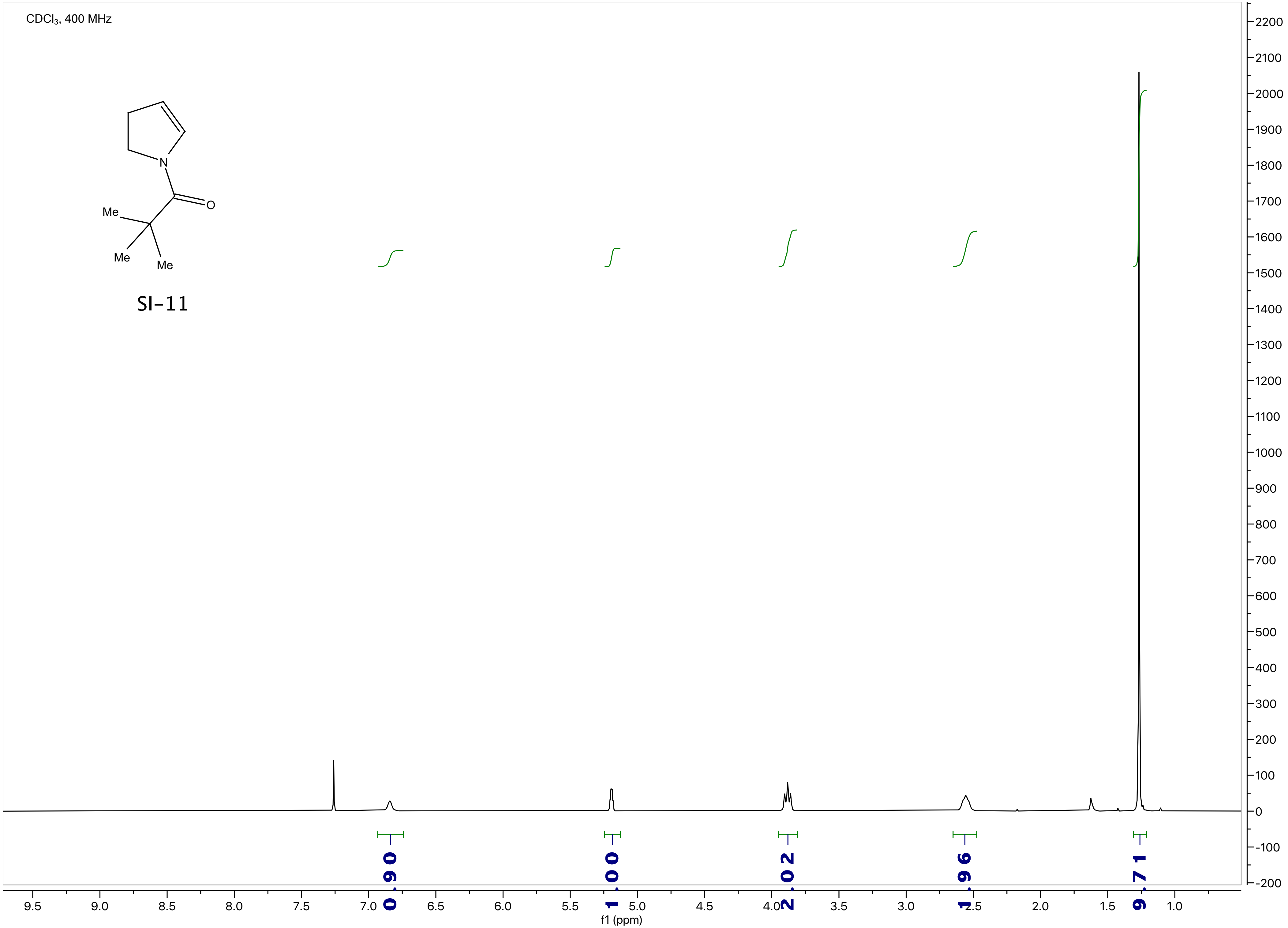

CDCl3, 400 MHz
N
O
Me
Me
Me
SI-11
0.90
1.00
2.02
1.96
9.71
9.5
9.0
8.5
8.0
7.5
7.0
6.5
6.0
5.5
5.0
4.5
4.0
3.5
3.0
2.5
2.0
1.5
1.0
f1 (ppm)
2200
2100
2000
1900
1800
1700
1600
1500
1400
1300
1200
1100
1000
900
800
700
600
500
400
300
200
100
0
-100
-200

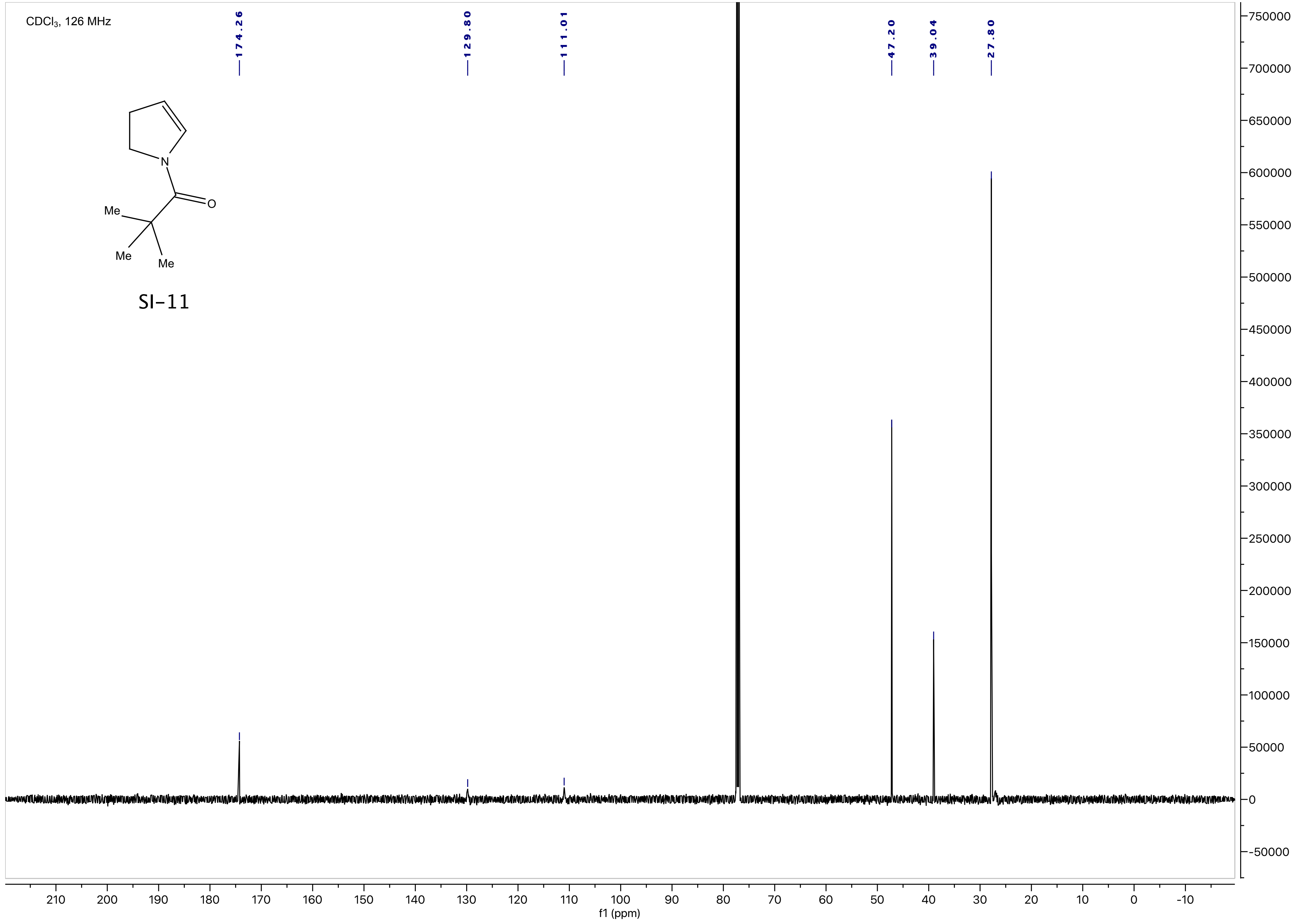

CDCl3, 126 MHz
174.26
129.80
111.01
47.20
39.04
27.80
N
O
Me
Me
Me
SI-11
f1 (ppm)

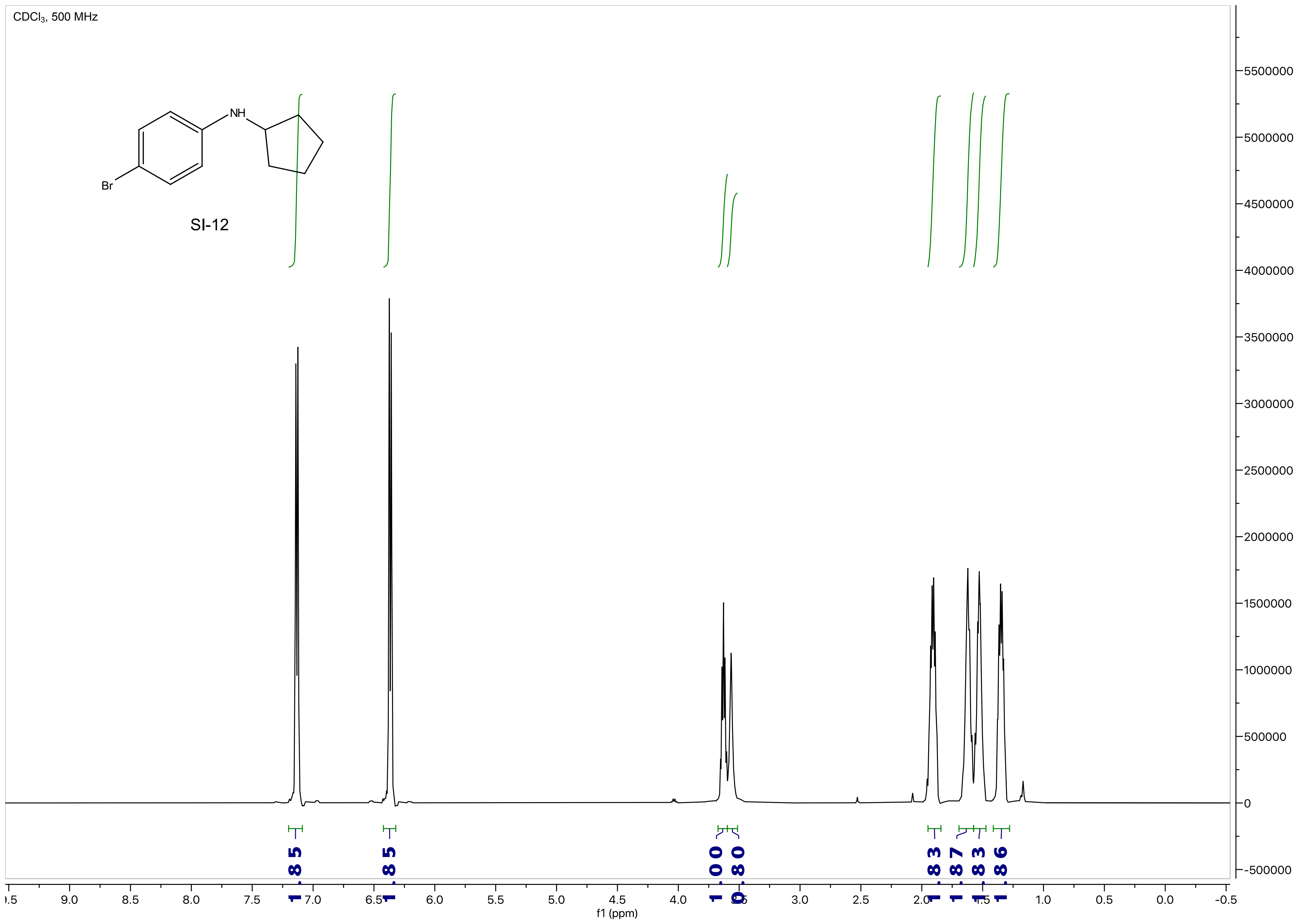
CDCl3, 500 MHz
NH
Br
SI-12
f1 (ppm)

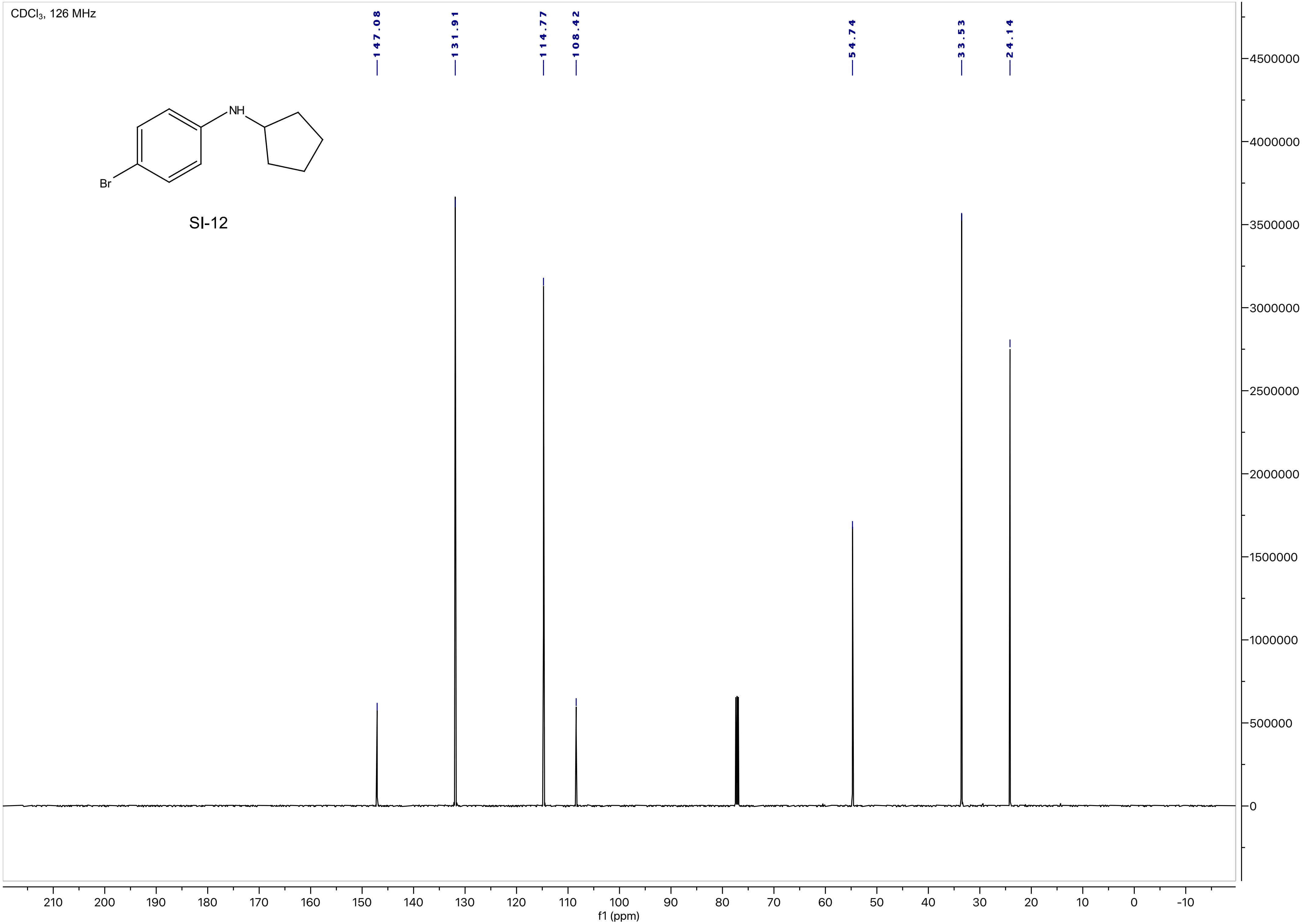
CDCl3, 126 MHz
147.08
131.91
114.77
108.42
54.74
33.53
24.14
NH
Br
SI-12
4500000
4000000
3500000
3000000
2500000
2000000
1500000
1000000
500000
0
210
200
190
180
170
160
150
140
130
120
110
100
90
80
70
60
50
40
30
20
10
0
-10
f1 (ppm)

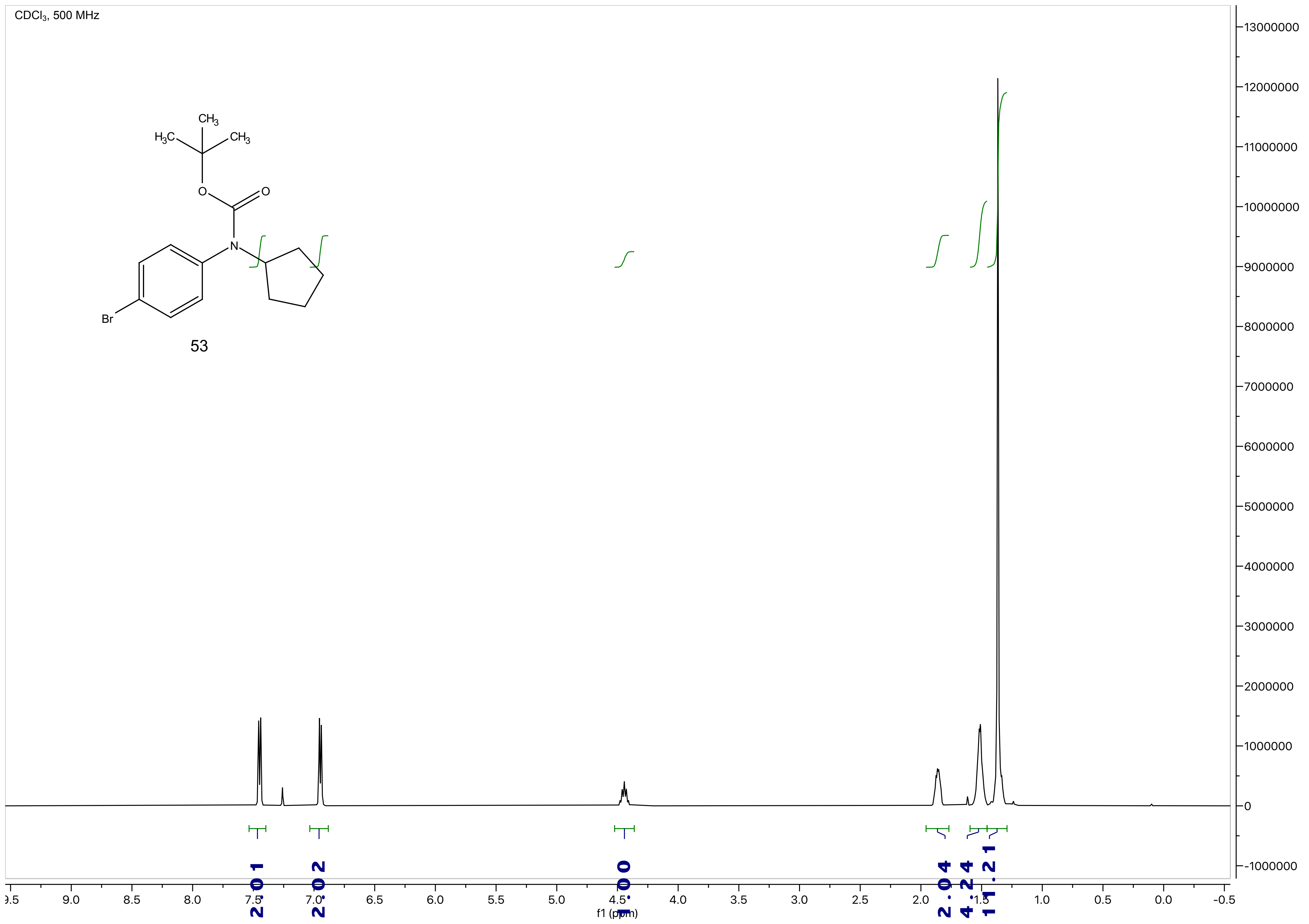

CDCl3, 500 MHz
CH3
H3C
CH3
O
O
N
Br
53
2.01
2.02
1.00
2.04
4.24
11.21
9.0 8.5 8.0 7.5 7.0 6.5 6.0 5.5 5.0 4.5 4.0 3.5 3.0 2.5 2.0 1.5 1.0 0.5 0.0 -0.5
f1 (ppm)

CDCl$_3$, 126 MHz

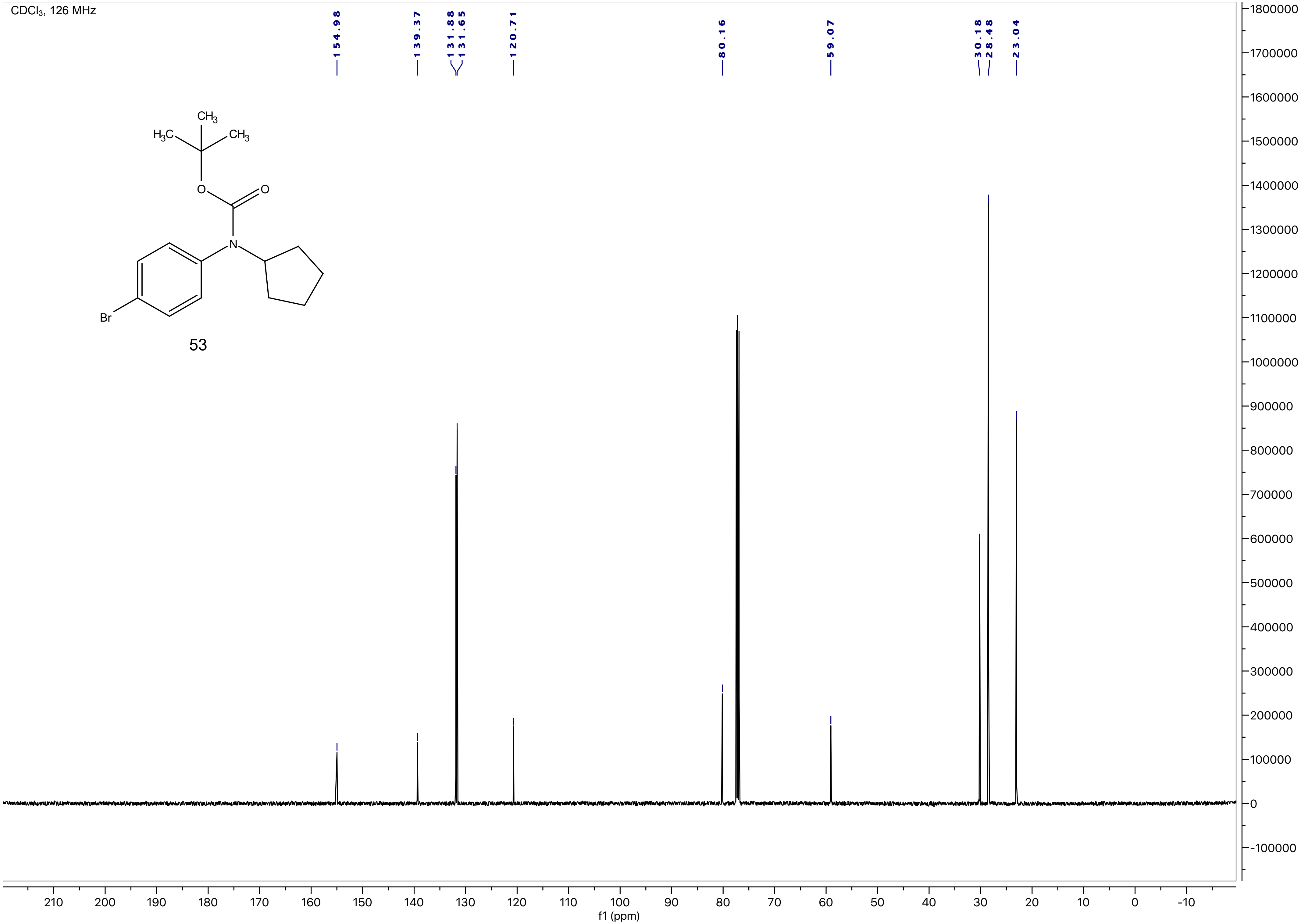

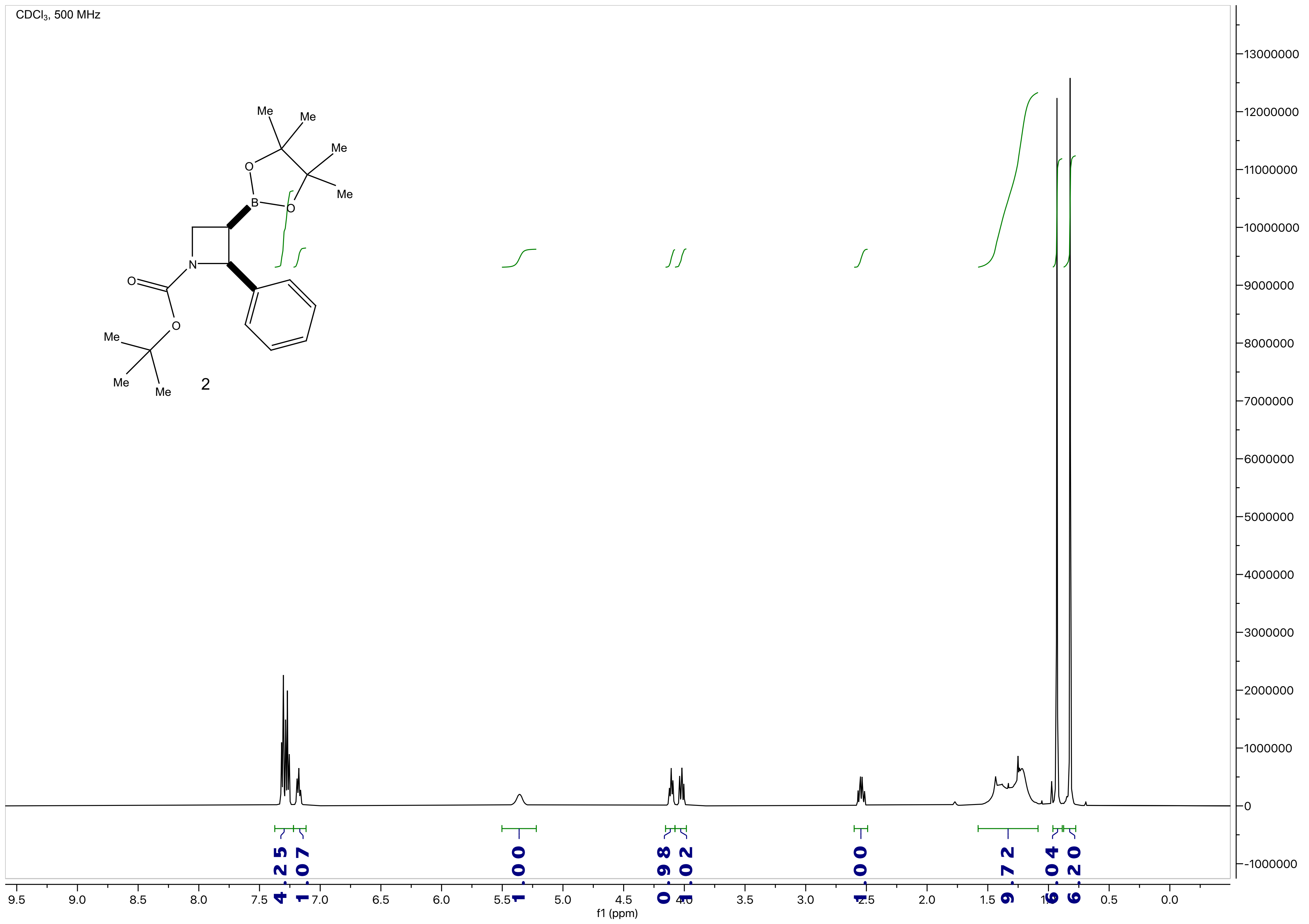

CDCl3, 500 MHz
Me
Me
Me
Me
O
O
B
N
O
O
Me
Me
Me
2
4.25
1.07
1.00
0.98
1.02
1.00
9.72
6.04
6.20
f1 (ppm)

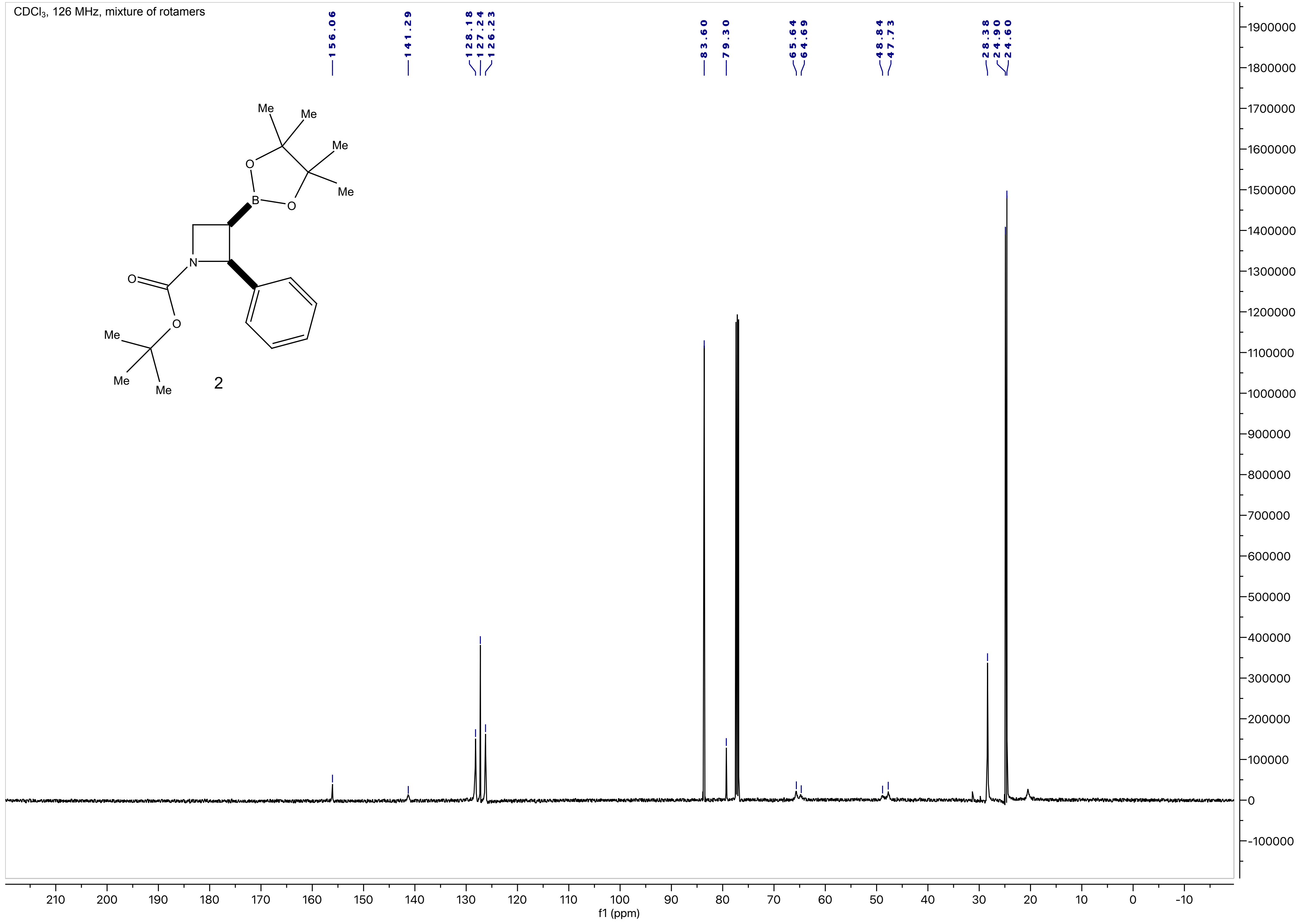
CDCl3, 126 MHz, mixture of rotamers
156.06
141.29
128.18
127.24
126.23
83.60
79.30
65.64
64.69
48.84
47.73
28.38
24.90
24.60
Me
Me
Me
Me
O
O
B
N
O
O
Me
Me
Me
2
f1 (ppm)

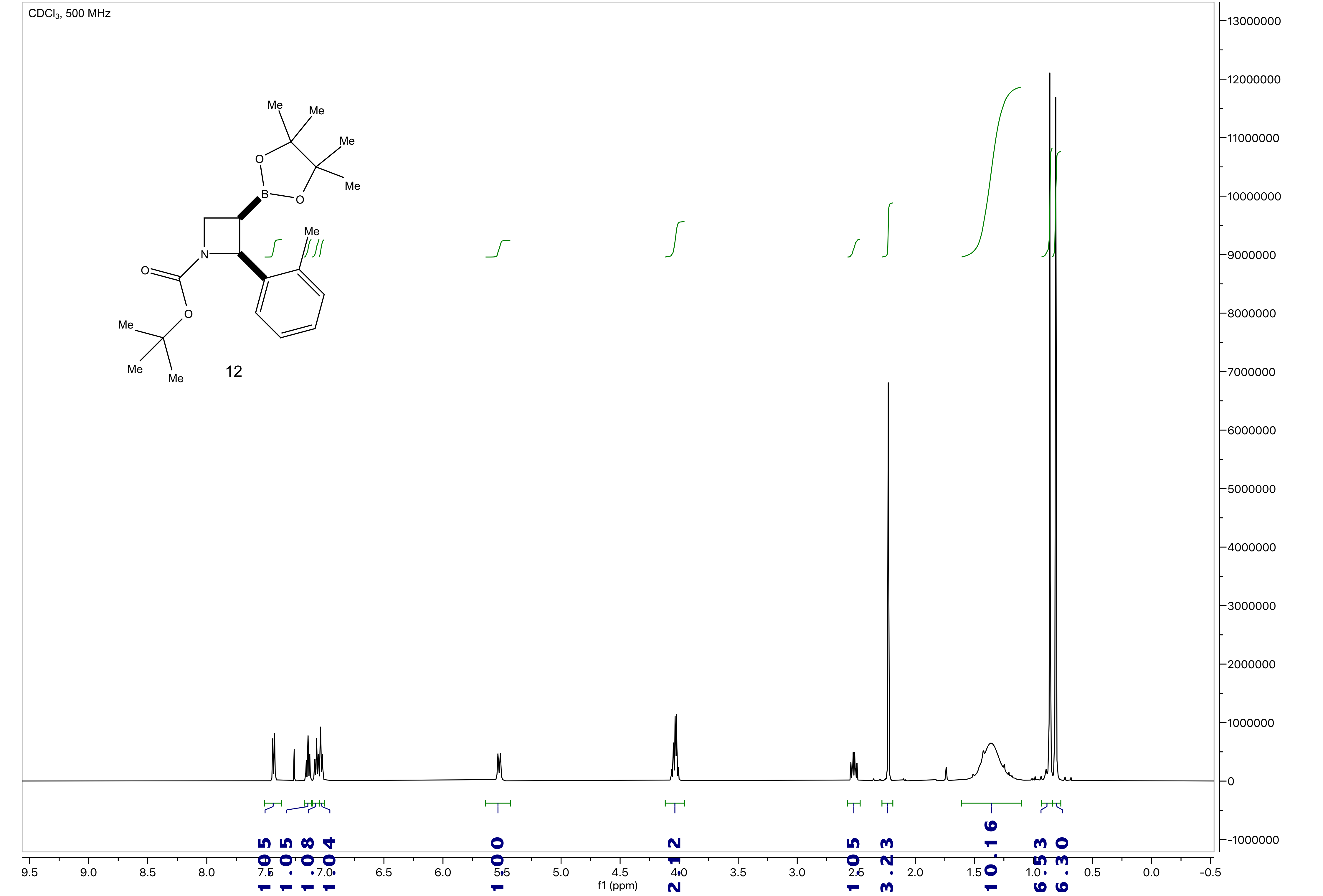

CDCl$_3$, 500 MHz
12
Me
O
B
N
1.05
1.05
1.08
1.04
1.00
2.12
1.05
3.23
10.16
6.53
6.30
f1 (ppm)

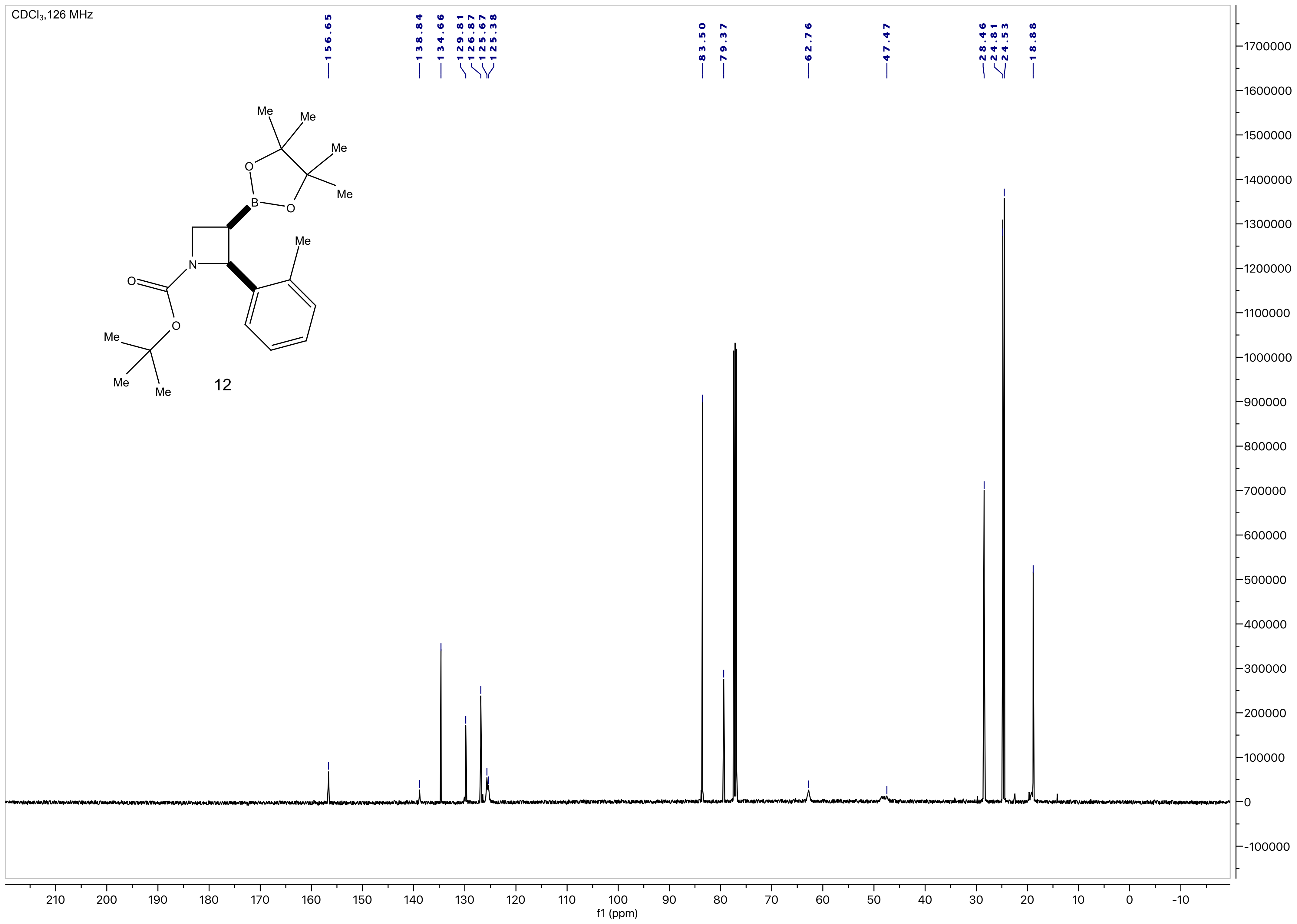

CDCl3,126 MHz
156.65
138.84
134.66
129.81
126.87
125.67
125.38
83.50
79.37
62.76
47.47
28.46
24.81
24.53
18.88
Me
Me
Me
Me
O
B
O
N
O
O
Me
Me
Me
Me
12
f1 (ppm)

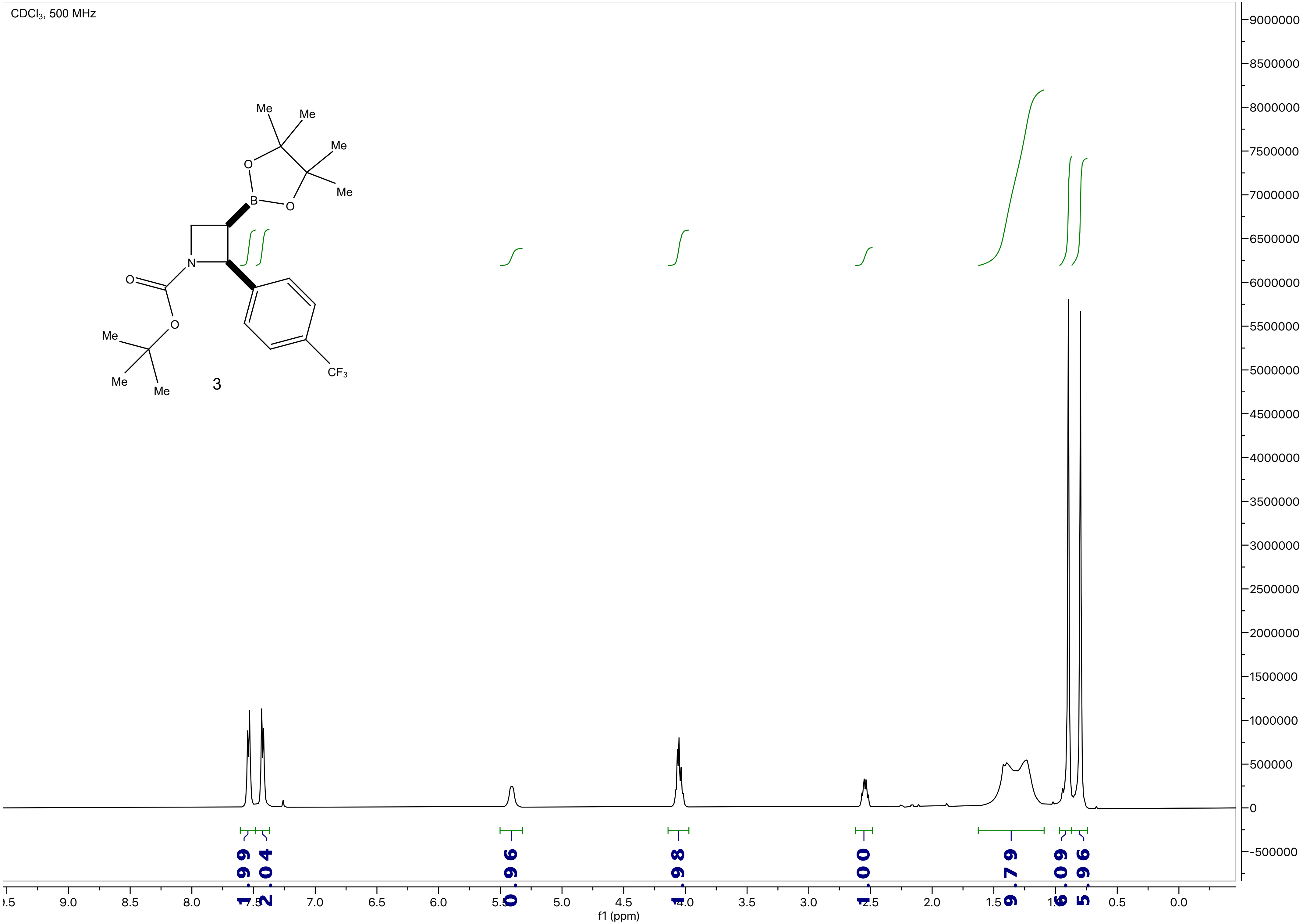

CDCl₃, 500 MHz
Me
Me
Me
Me
O
B
O
N
O
O
Me
Me
Me
CF₃
3
2.04
0.96
1.00
9.79
6.09
5.96
f1 (ppm)

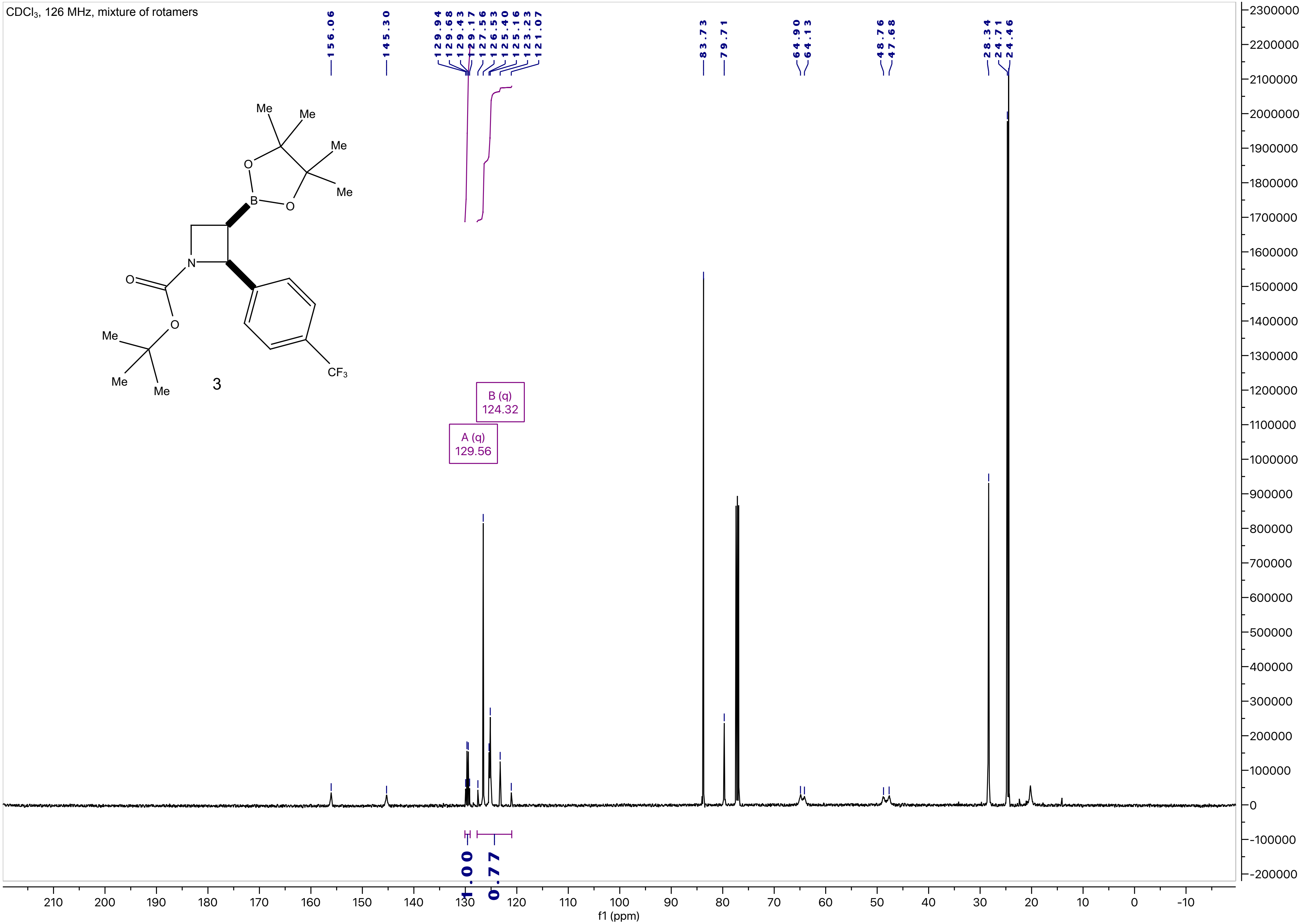

CDCl3, 126 MHz, mixture of rotamers
156.06
145.30
129.94
129.68
129.43
129.17
127.56
126.53
125.40
125.16
123.23
121.07
83.73
79.71
64.90
64.13
48.76
47.68
28.34
24.71
24.46
Me
Me
Me
Me
O
B
O
N
O
O
Me
Me
Me
CF3
3
B (q)
124.32
A (q)
129.56
1.00
0.77
f1 (ppm)

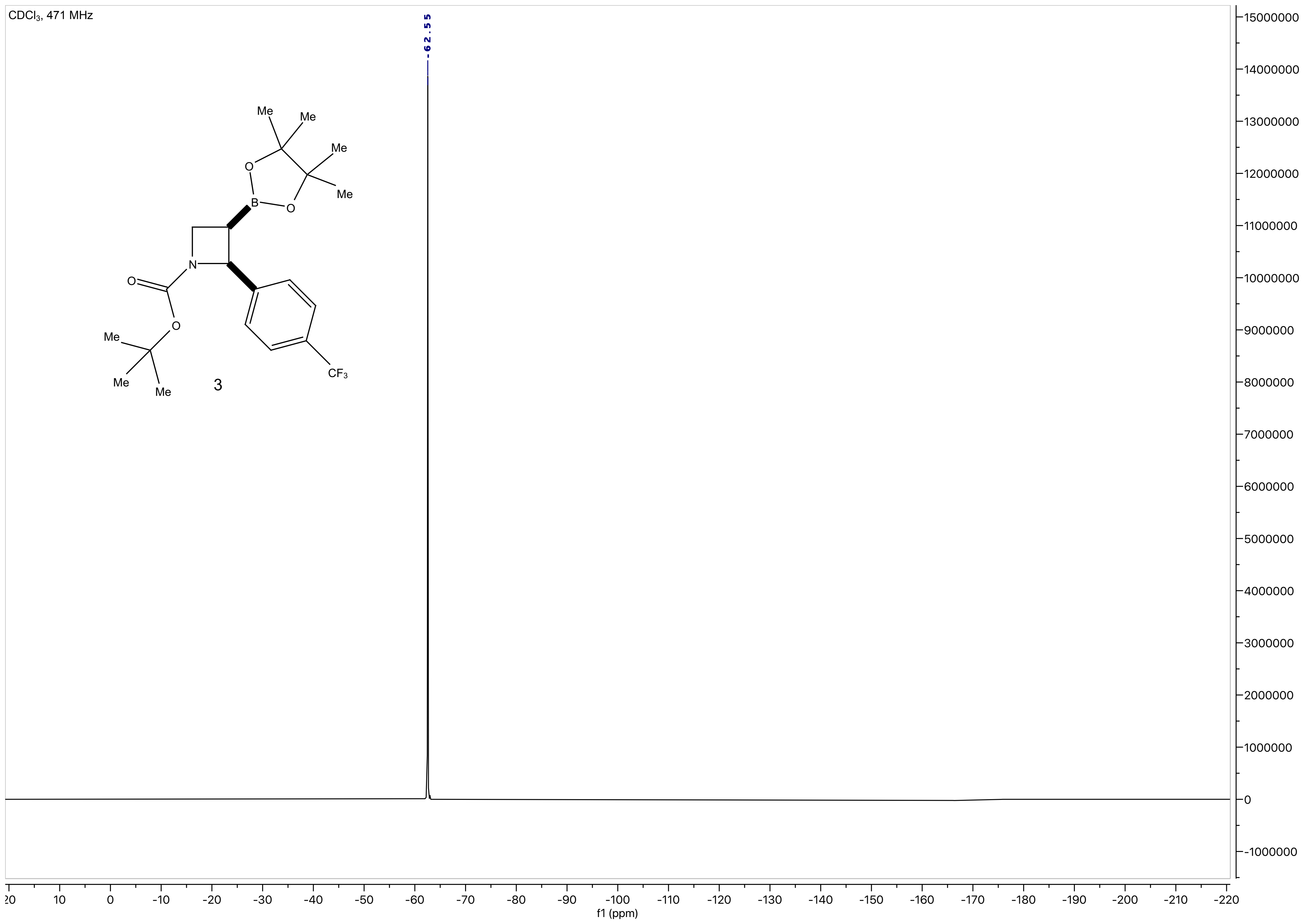
CDCl3, 471 MHz
-62.55
Me
Me
Me
Me
O
B
O
N
O
O
Me
Me
Me
CF3
3
f1 (ppm)
20
10
0
-10
-20
-30
-40
-50
-60
-70
-80
-90
-100
-110
-120
-130
-140
-150
-160
-170
-180
-190
-200
-210
-220
15000000
14000000
13000000
12000000
11000000
10000000
9000000
8000000
7000000
6000000
5000000
4000000
3000000
2000000
1000000
0
-1000000

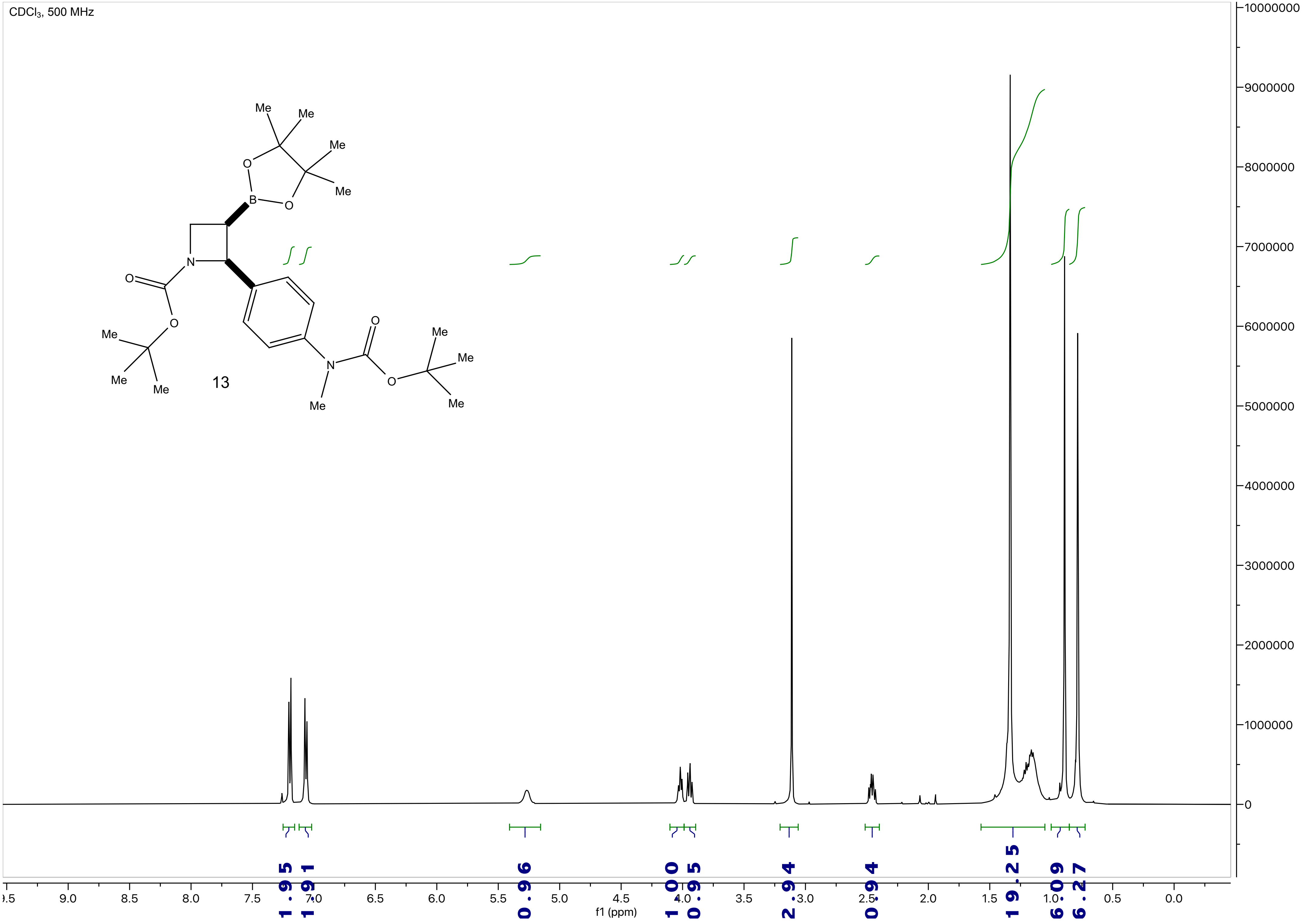
CDCl3, 500 MHz
13
f1 (ppm)
1.95
1.91
0.96
1.00
0.95
2.94
0.94
19.25
6.09
6.27

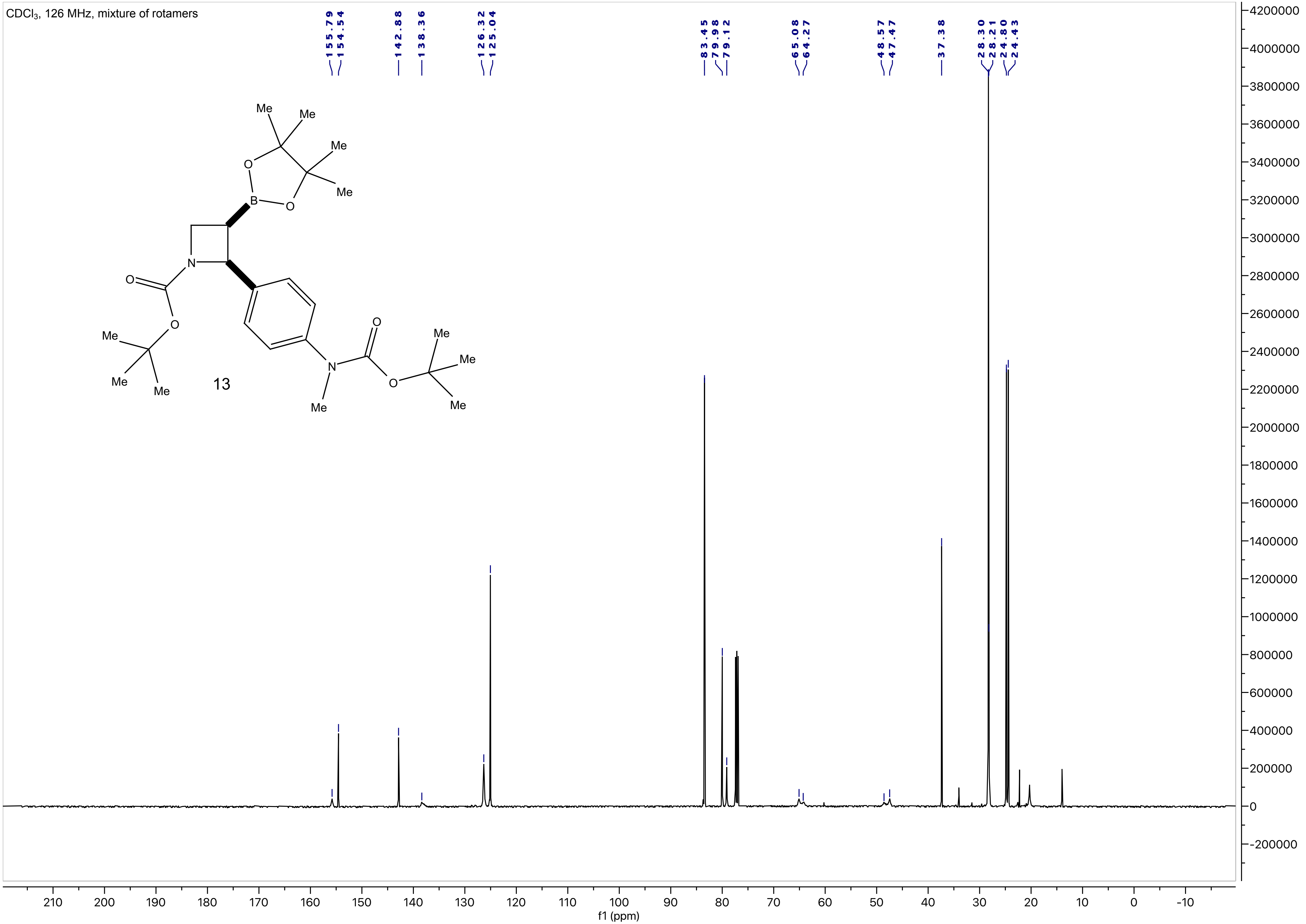
CDCl$_3$, 126 MHz, mixture of rotamers
155.79
154.54
142.88
138.36
126.32
125.04
83.45
79.98
79.12
65.08
64.27
48.57
47.47
37.38
28.30
28.21
24.80
24.43
13
f1 (ppm)

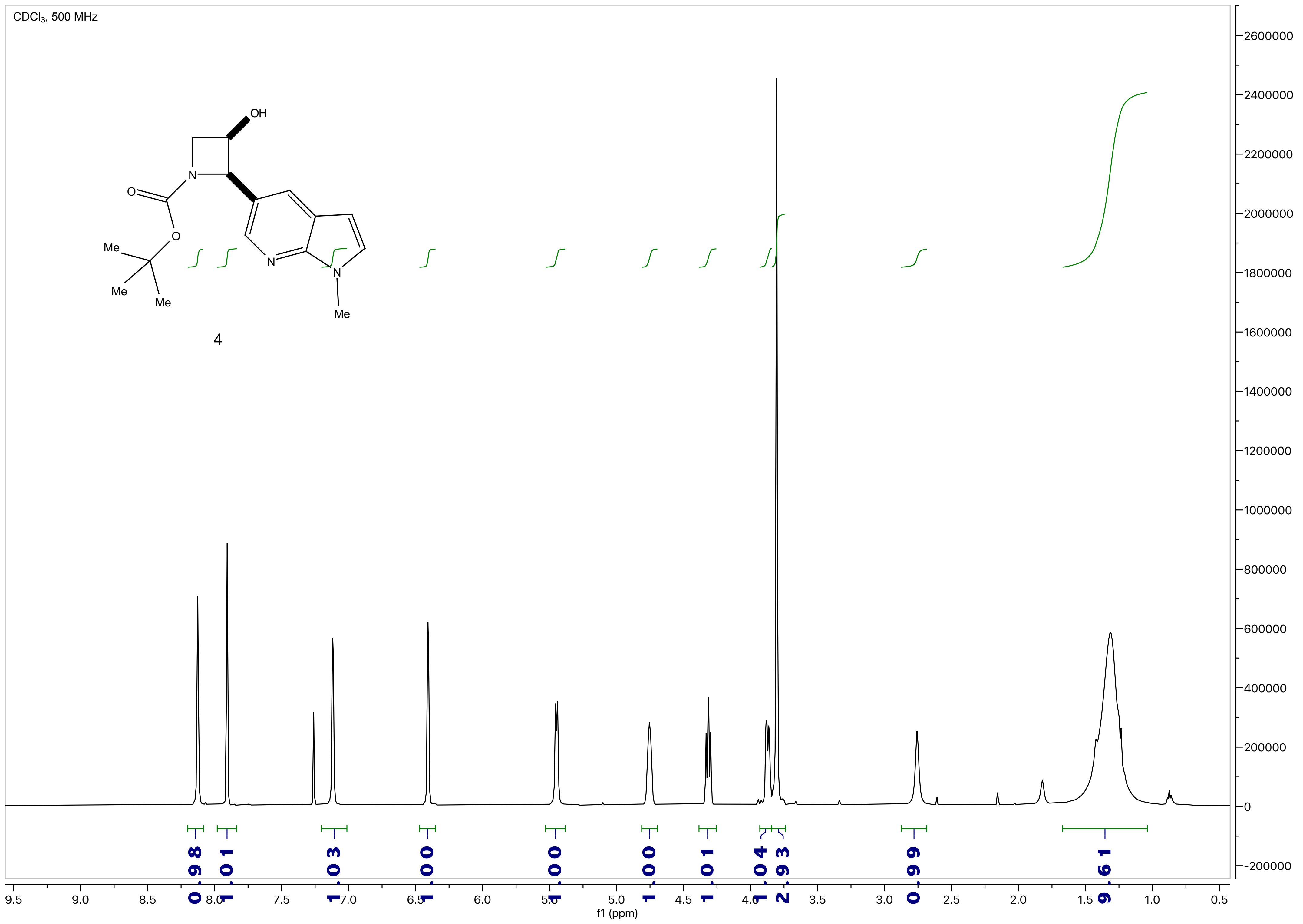

CDCl3, 500 MHz
OH
N
O
O
Me
Me
Me
N
N
Me
4
f1 (ppm)
0.98
1.01
1.03
1.00
1.00
1.00
1.01
1.04
2.93
0.99
9.61

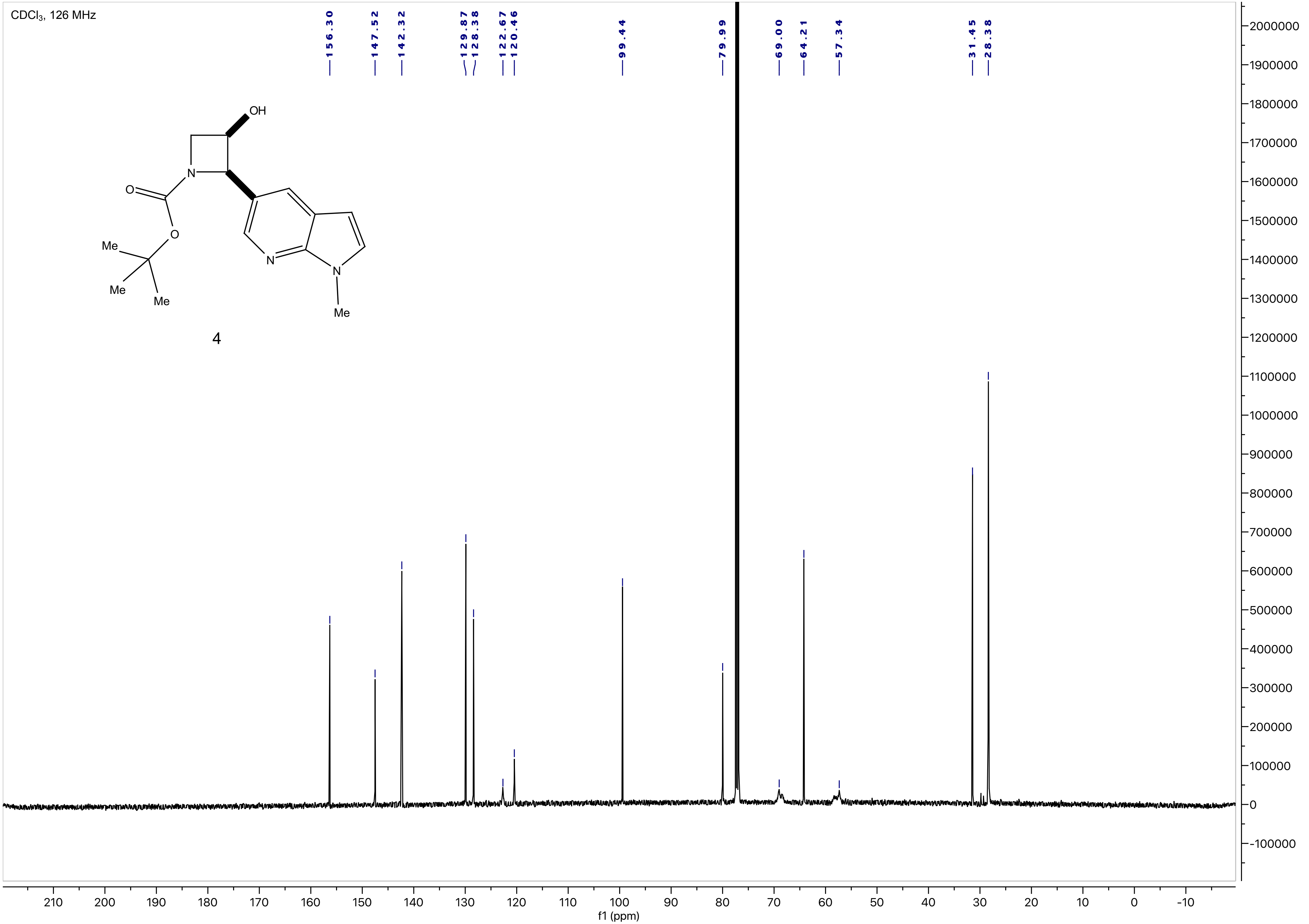
CDCl3, 126 MHz
156.30
147.52
142.32
129.87
128.38
122.67
120.46
99.44
79.99
69.00
64.21
57.34
31.45
28.38
OH
N
O
O
Me
Me
Me
N
N
Me
4
f1 (ppm)

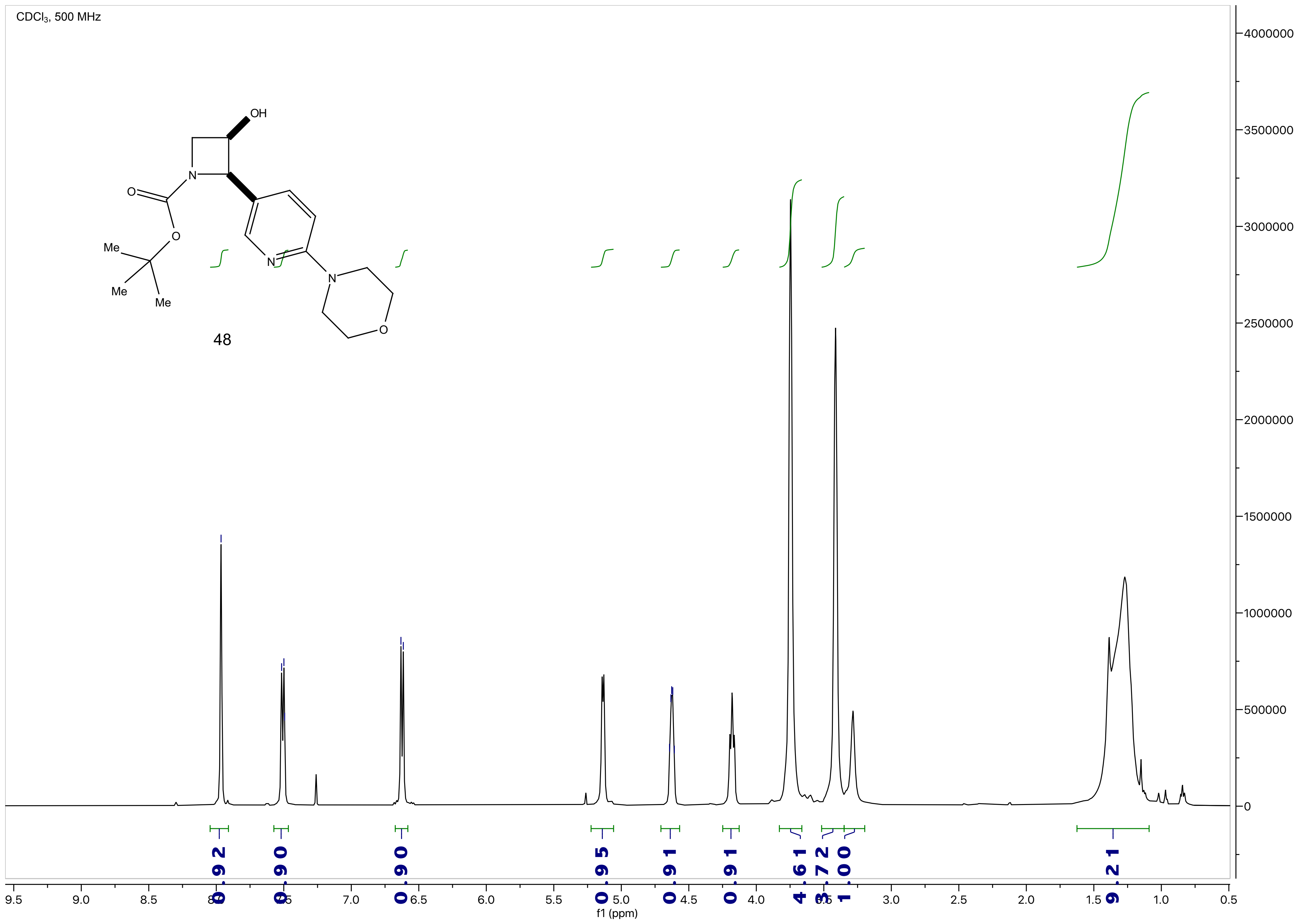

CDCl3, 500 MHz
OH
N
O
O
Me
Me
Me
N
N
O
48
0.92
0.90
0.90
0.95
0.91
0.91
4.61
3.72
1.00
9.21
9.5
9.0
8.5
7.0
6.5
6.0
5.5
5.0
4.5
4.0
3.0
2.5
2.0
1.5
1.0
0.5
f1 (ppm)
4000000
3500000
3000000
2500000
2000000
1500000
1000000
500000
0

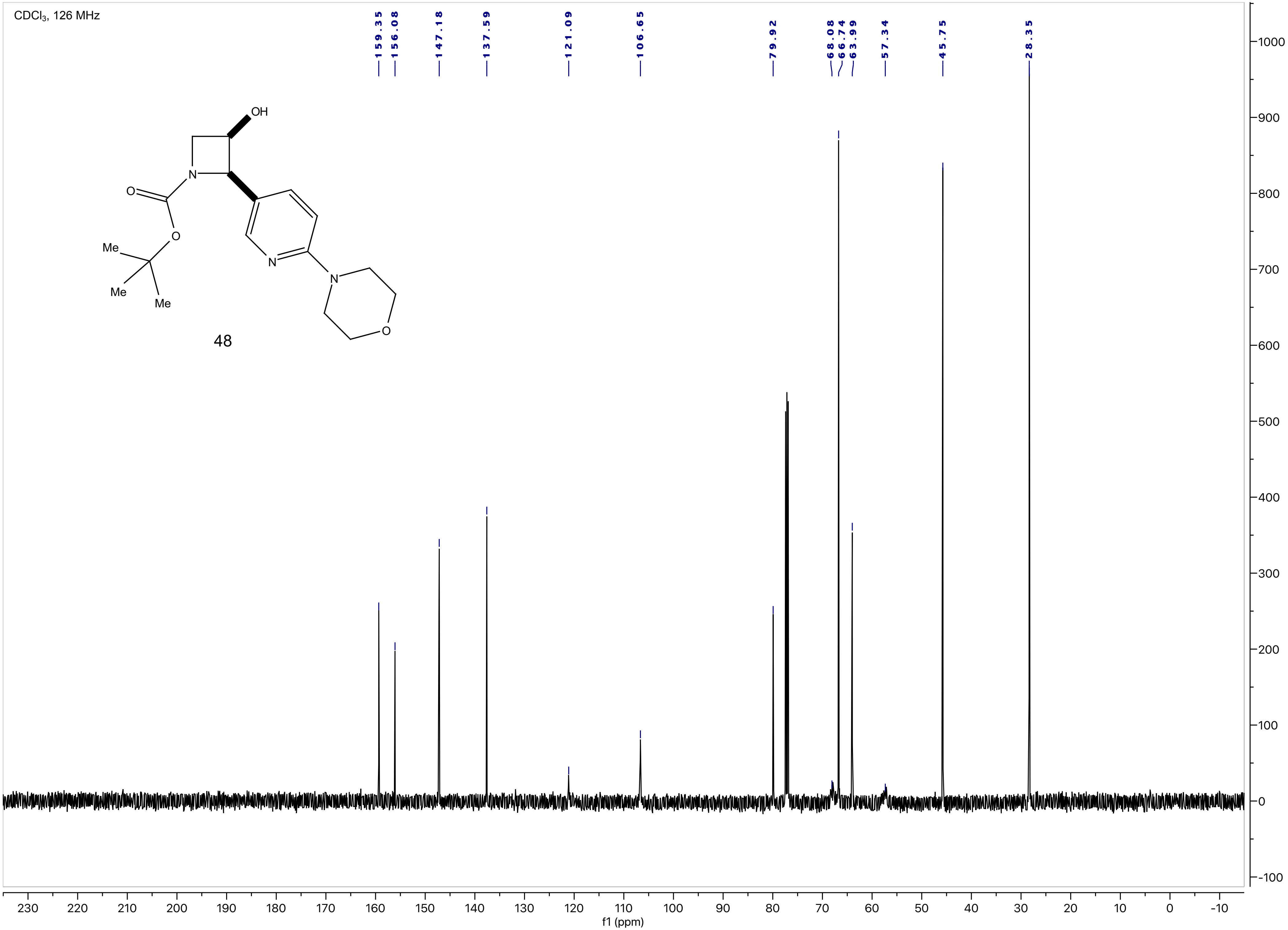
CDCl3, 126 MHz
159.35
156.08
147.18
137.59
121.09
106.65
79.92
68.08
66.74
63.99
57.34
45.75
28.35
OH
N
O
Me
Me
Me
N
N
O
48
f1 (ppm)

CDCl$_3$, 500 MHz

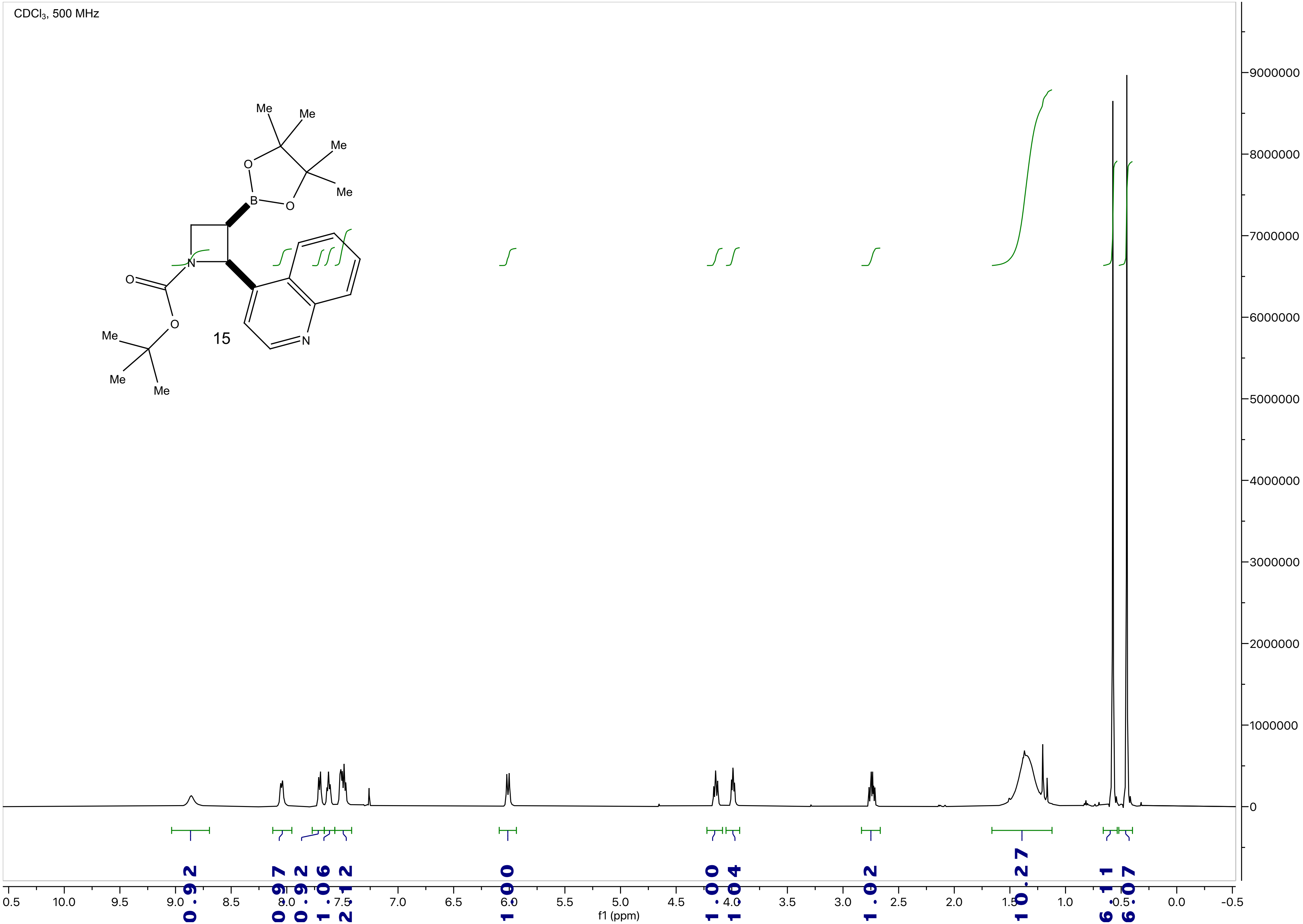

15

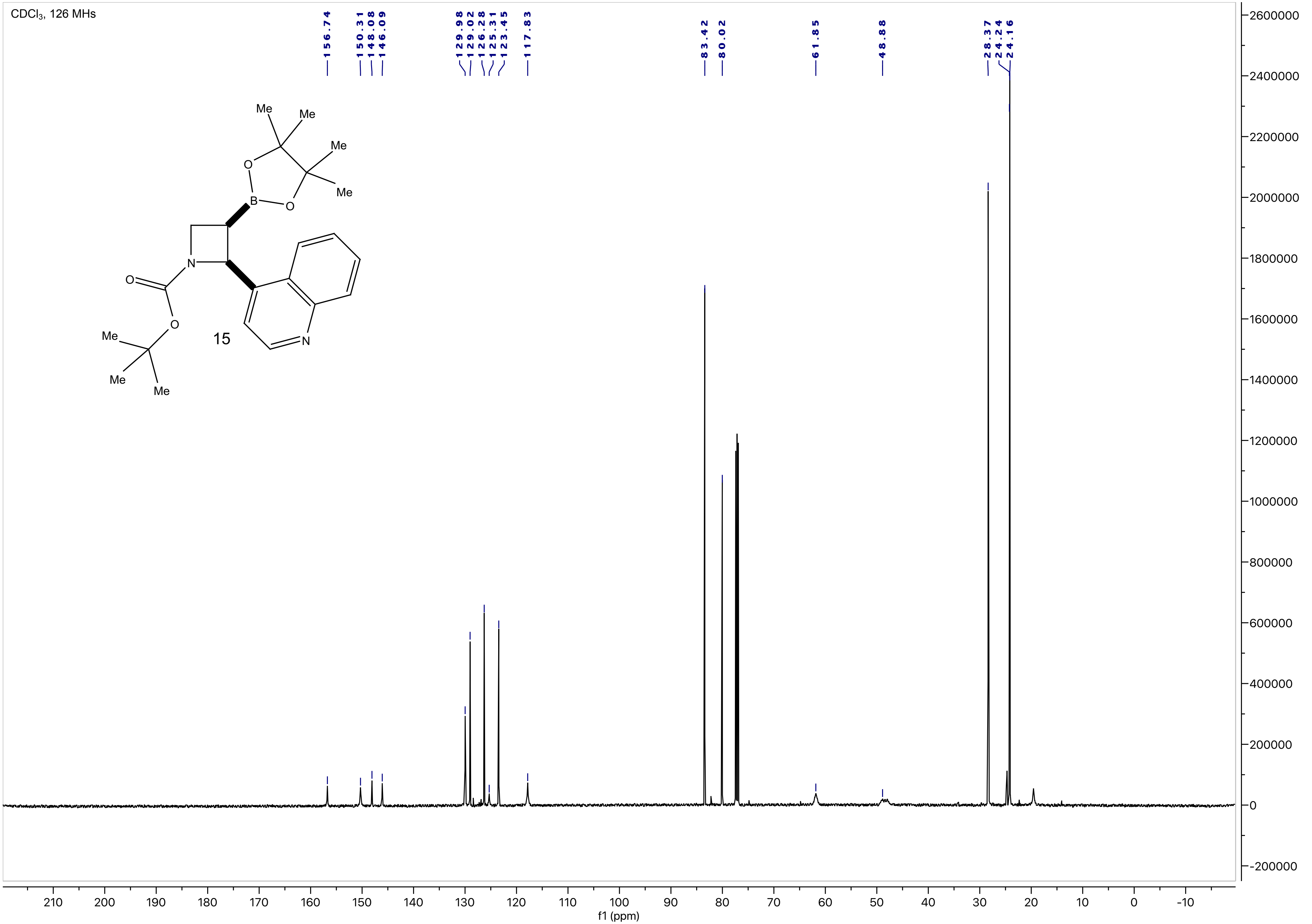

CDCl3, 126 MHs
156.74
150.31
148.08
146.09
129.98
129.02
126.28
125.31
123.45
117.83
83.42
80.02
61.85
48.88
28.37
24.24
24.16
15
f1 (ppm)

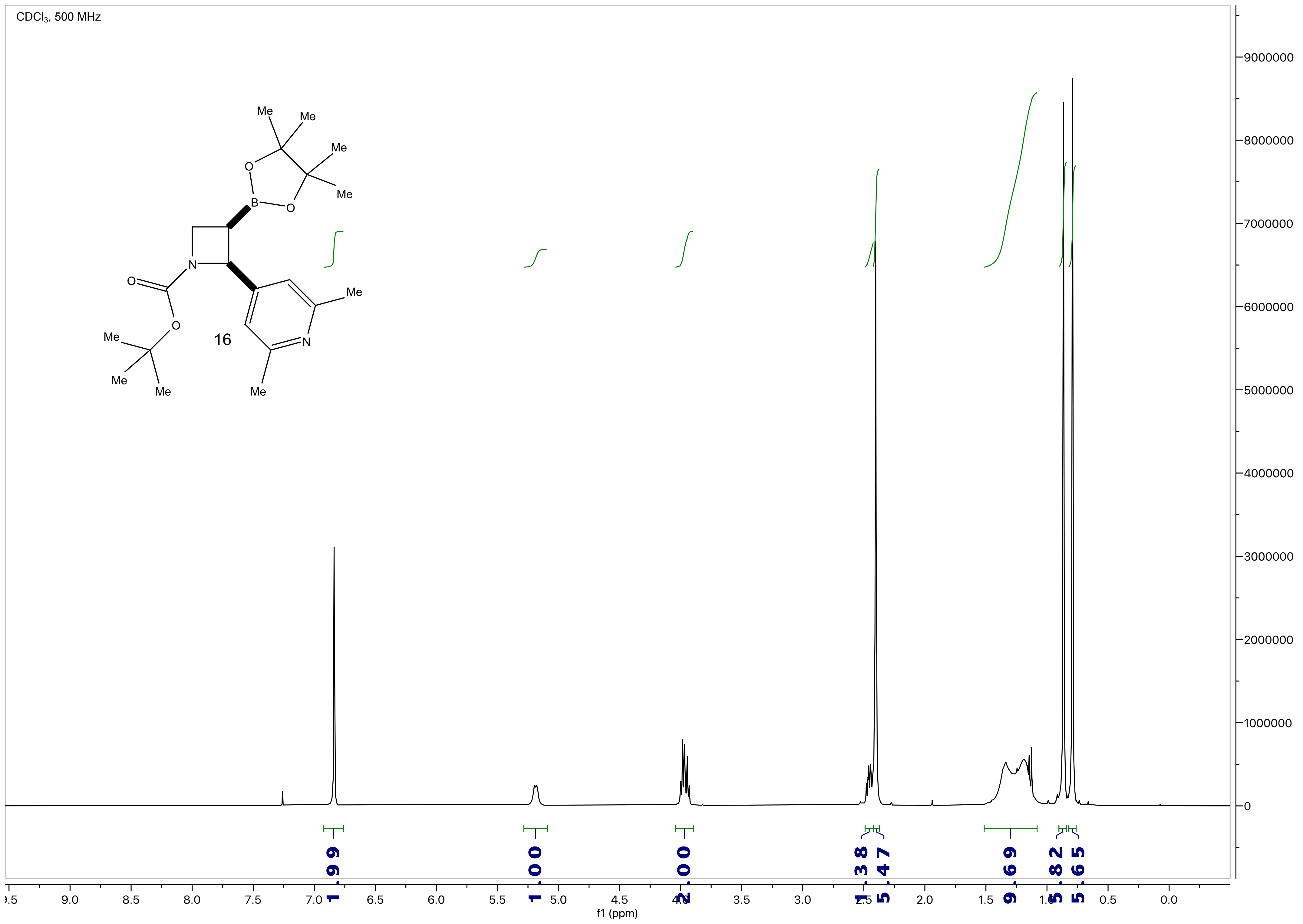

CDCl3, 500 MHz
16
1.99
1.00
2.00
1.38
5.47
9.69
5.82
5.65
f1 (ppm)

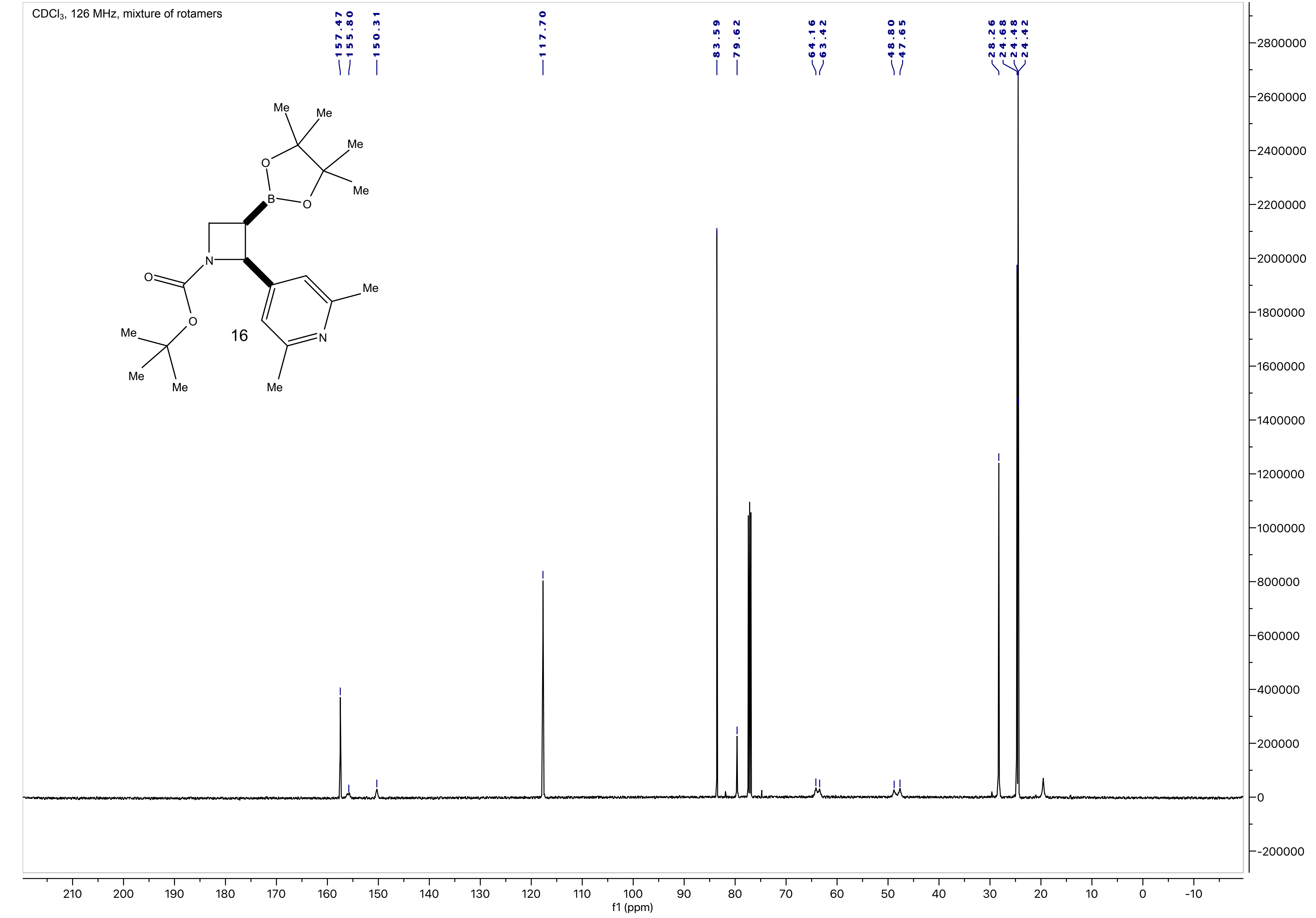
CDCl3, 126 MHz, mixture of rotamers
157.47
155.80
150.31
117.70
83.59
79.62
64.16
63.42
48.80
47.65
28.26
24.68
24.48
24.42
16
Me
O
B
N
f1 (ppm)

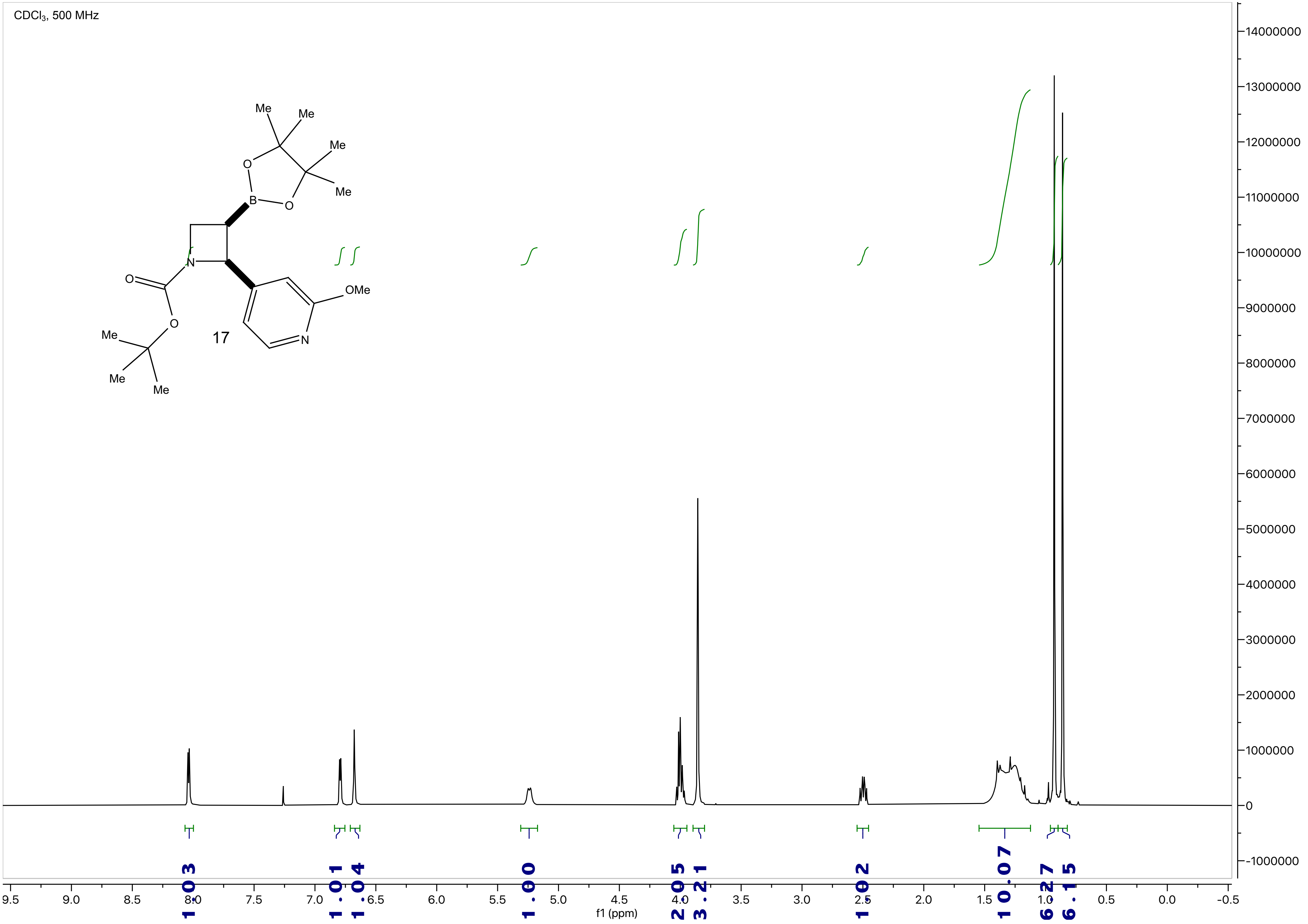

CDCl3, 500 MHz
17
OMe
1.03
1.01
1.04
1.00
2.05
3.21
1.02
10.07
6.27
6.15
f1 (ppm)

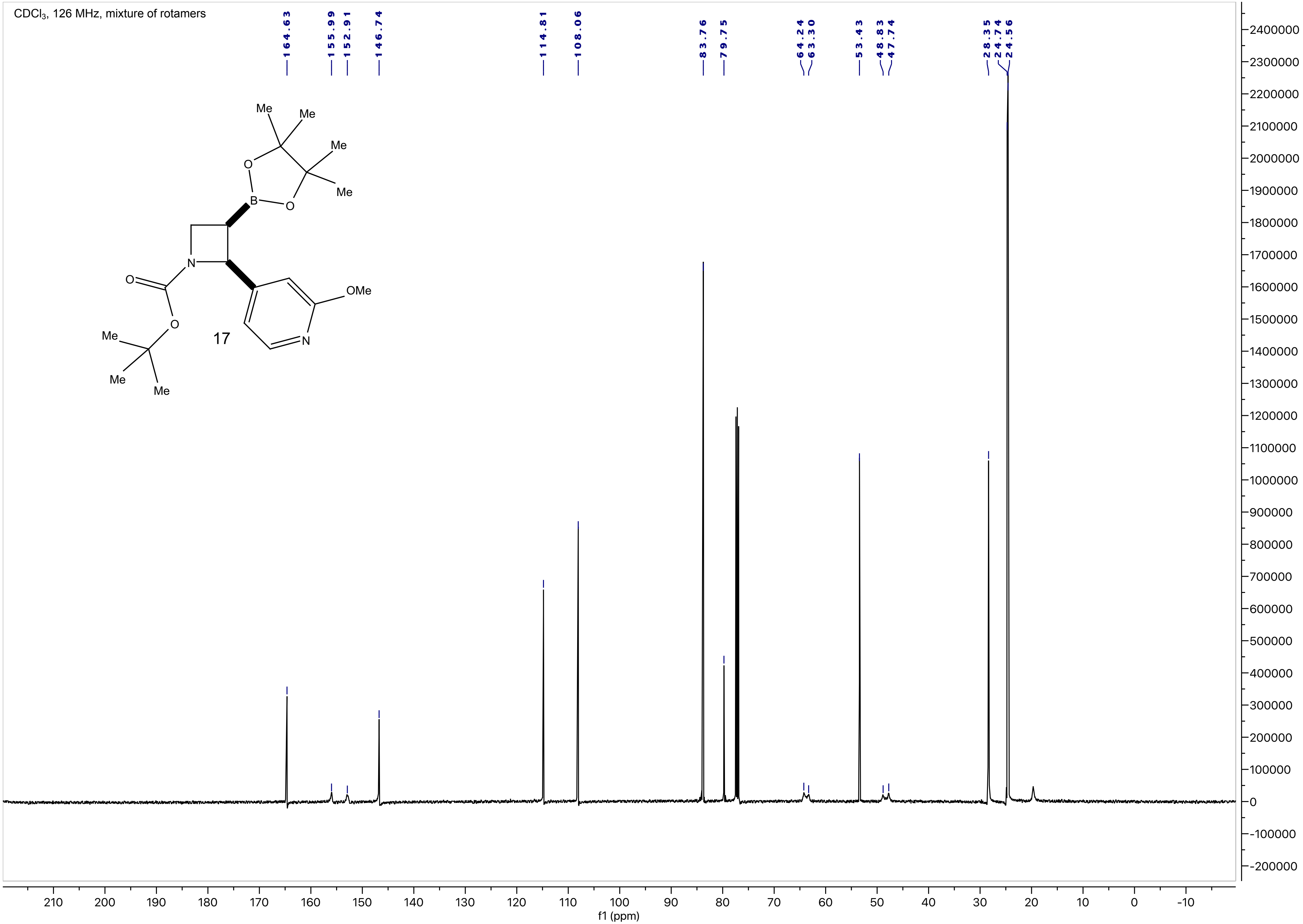
CDCl3, 126 MHz, mixture of rotamers
164.63
155.99
152.91
146.74
114.81
108.06
83.76
79.75
64.24
63.30
53.43
48.83
47.74
28.35
24.74
24.56
17
f1 (ppm)

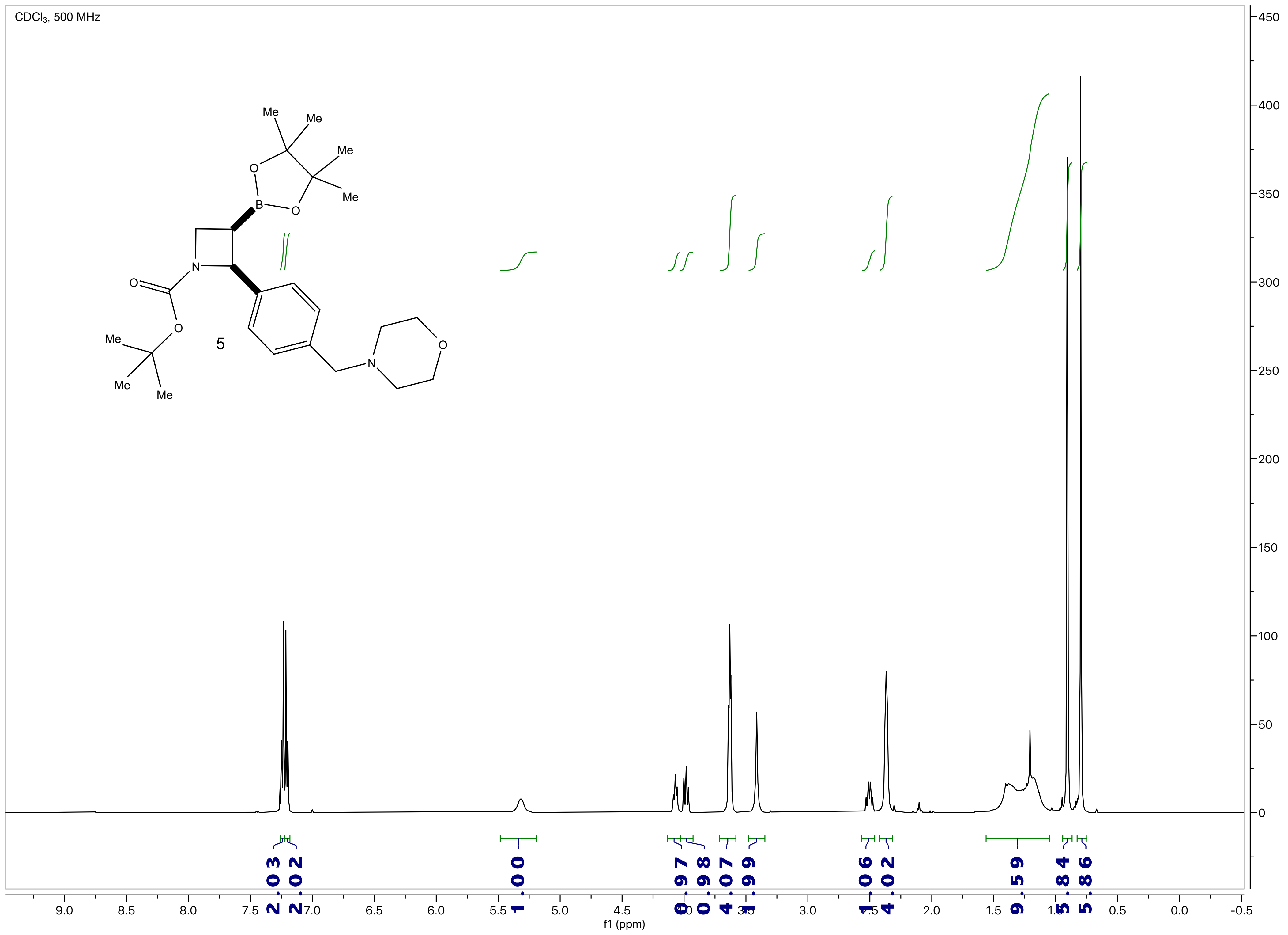

CDCl3, 500 MHz
5
f1 (ppm)
2.03
2.02
1.00
0.97
0.98
4.07
1.99
1.06
4.02
9.59
5.84
5.86

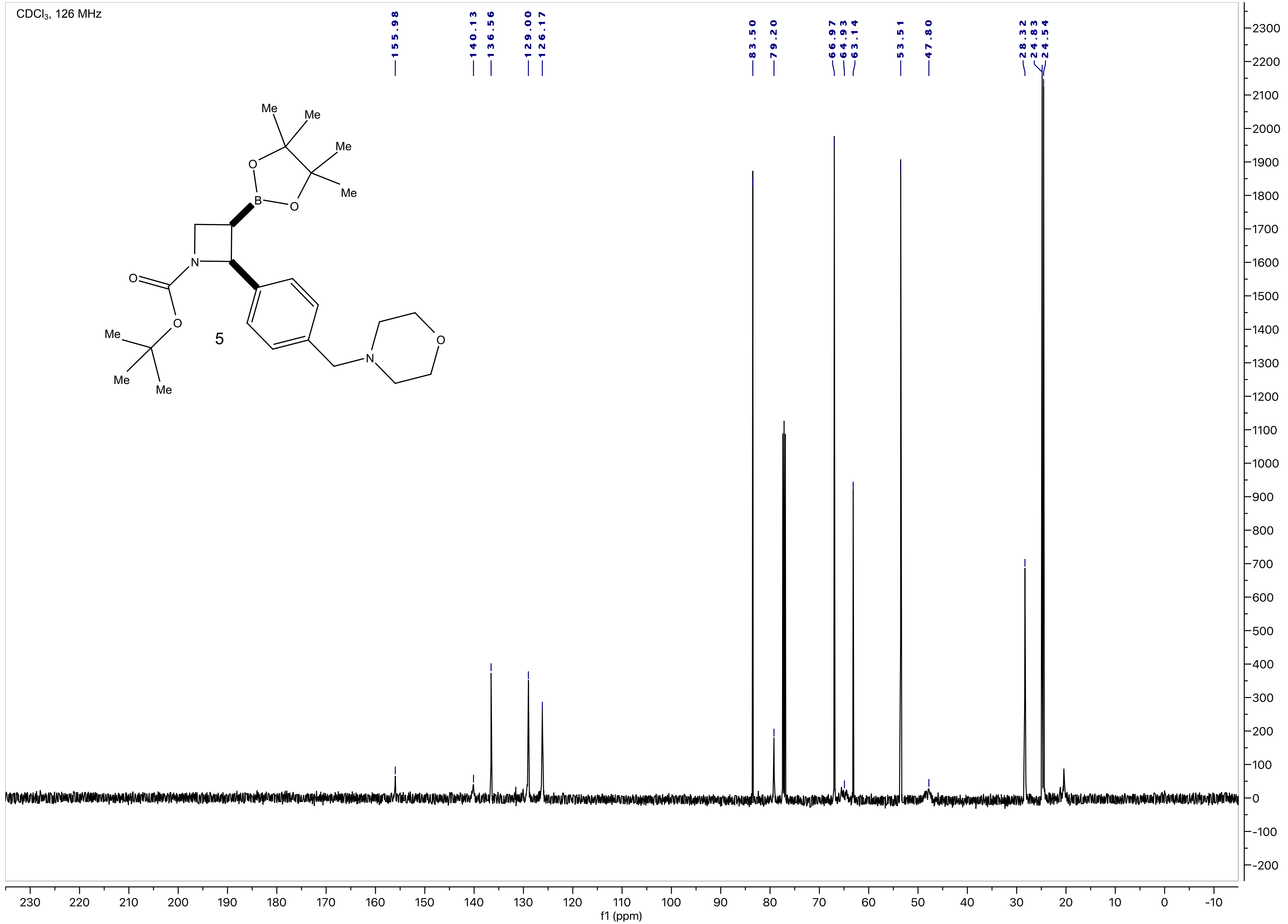
CDCl3, 126 MHz
155.98
140.13
136.56
129.00
126.17
83.50
79.20
66.97
64.93
63.14
53.51
47.80
28.32
24.83
24.54
5
f1 (ppm)

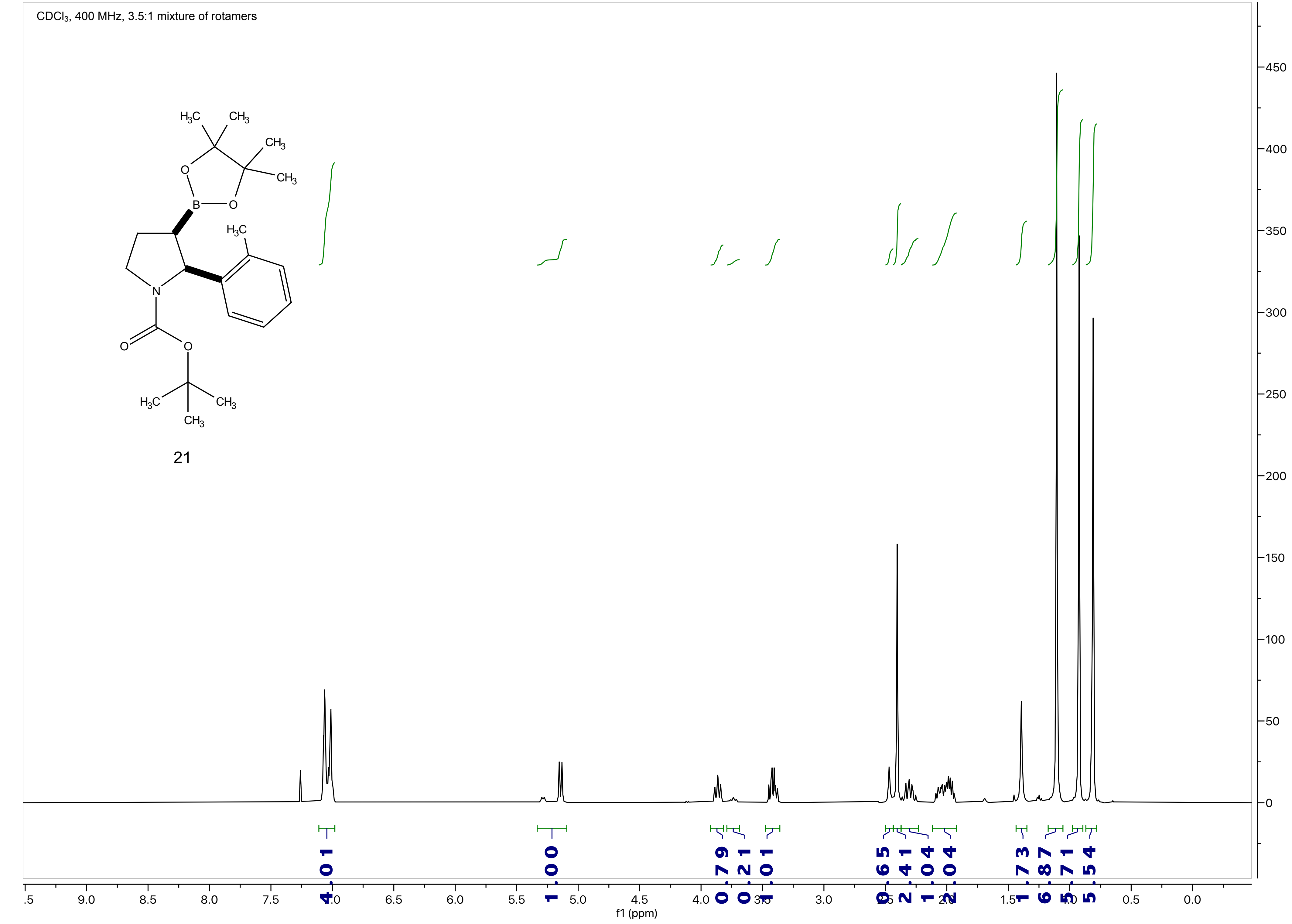
CDCl3, 400 MHz, 3.5:1 mixture of rotamers
21
4.01
1.00
0.79
0.21
1.01
0.65
2.41
1.04
2.04
1.73
6.87
5.71
5.54
f1 (ppm)

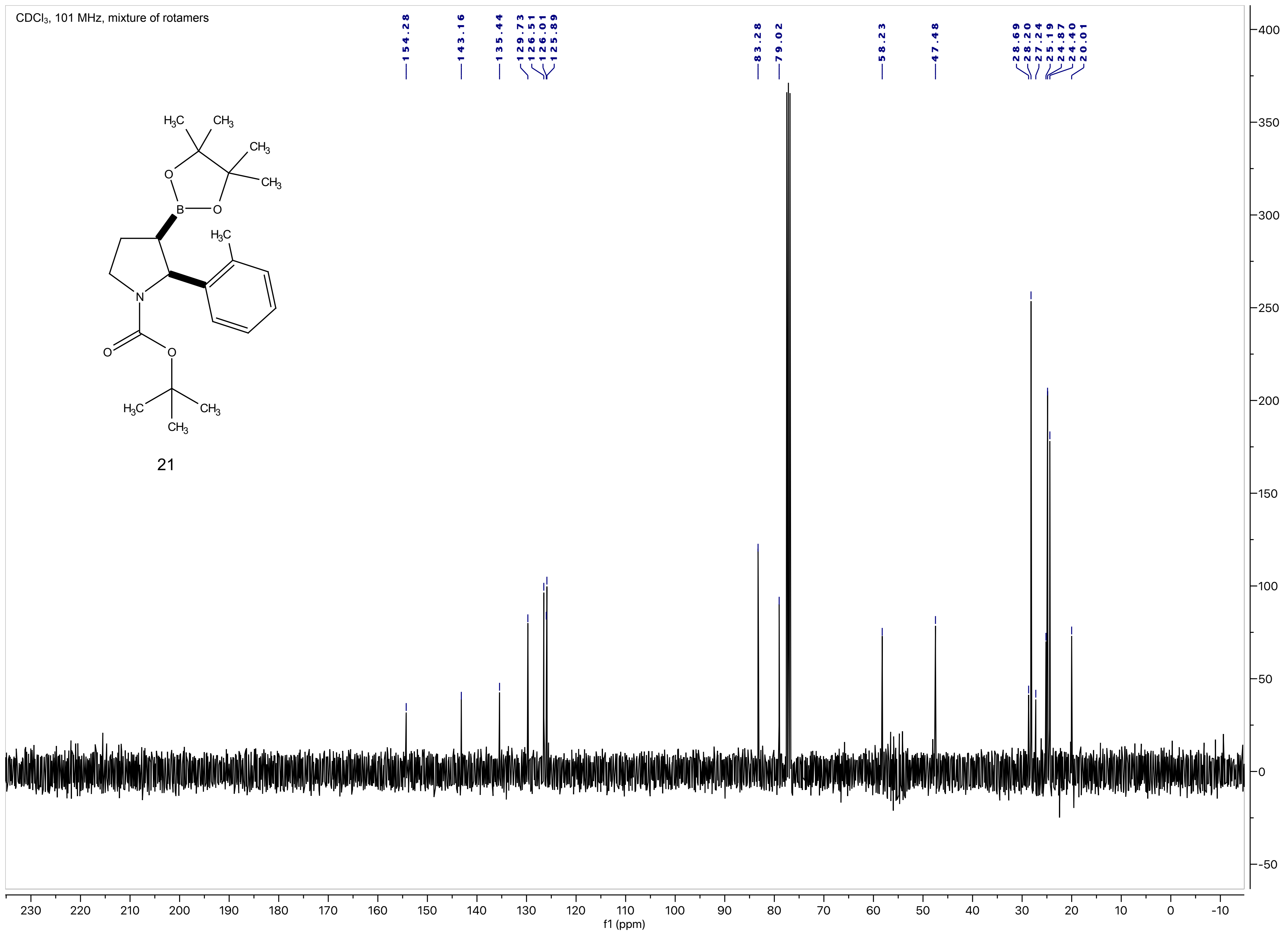
CDCl3, 101 MHz, mixture of rotamers
154.28
143.16
135.44
129.73
126.51
126.01
125.89
83.28
79.02
58.23
47.48
28.69
28.20
27.24
25.19
24.87
24.40
20.01
21
f1 (ppm)

CDCL3, 500 MHz, 2.4:1 mixture of rotamers

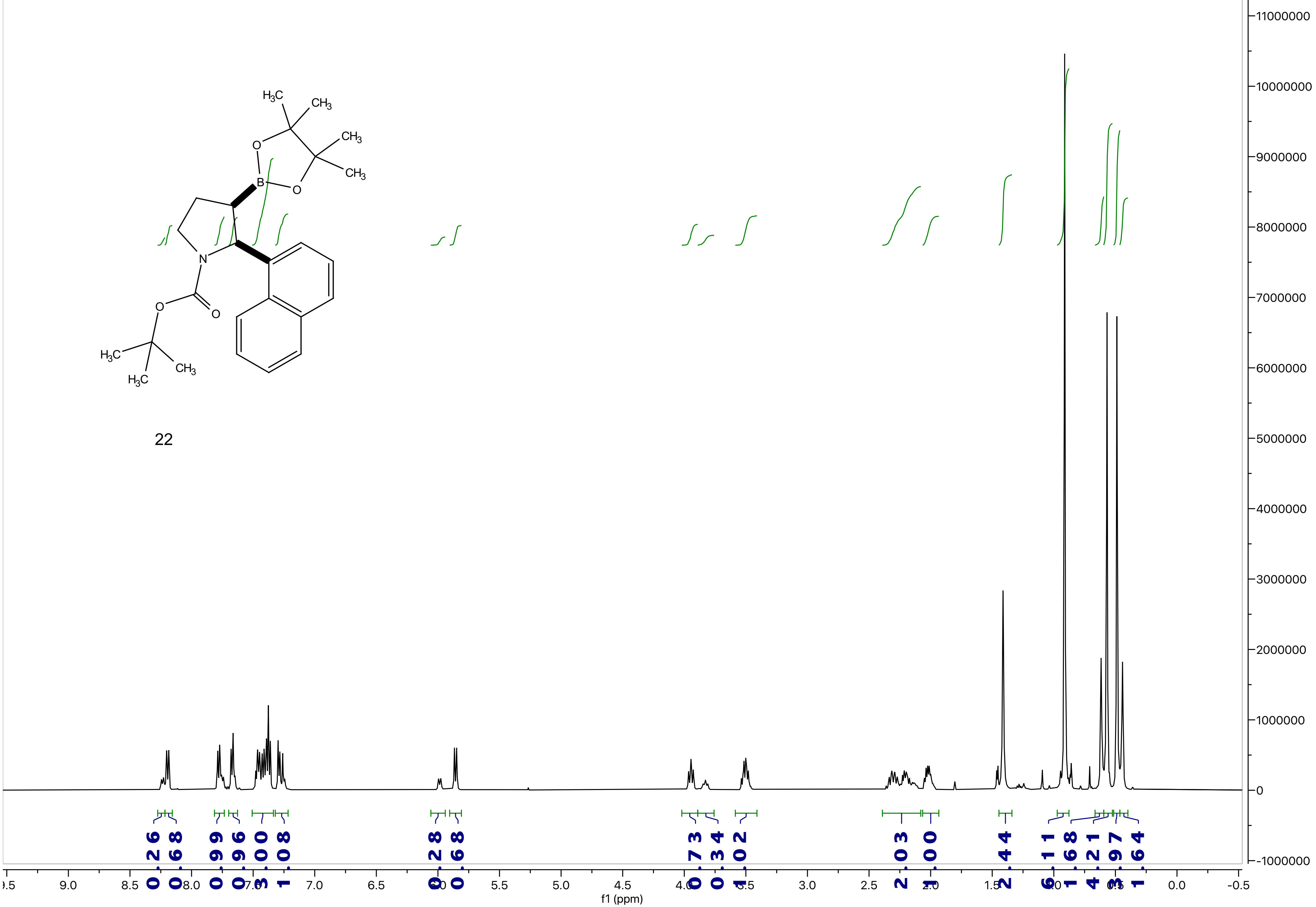

22

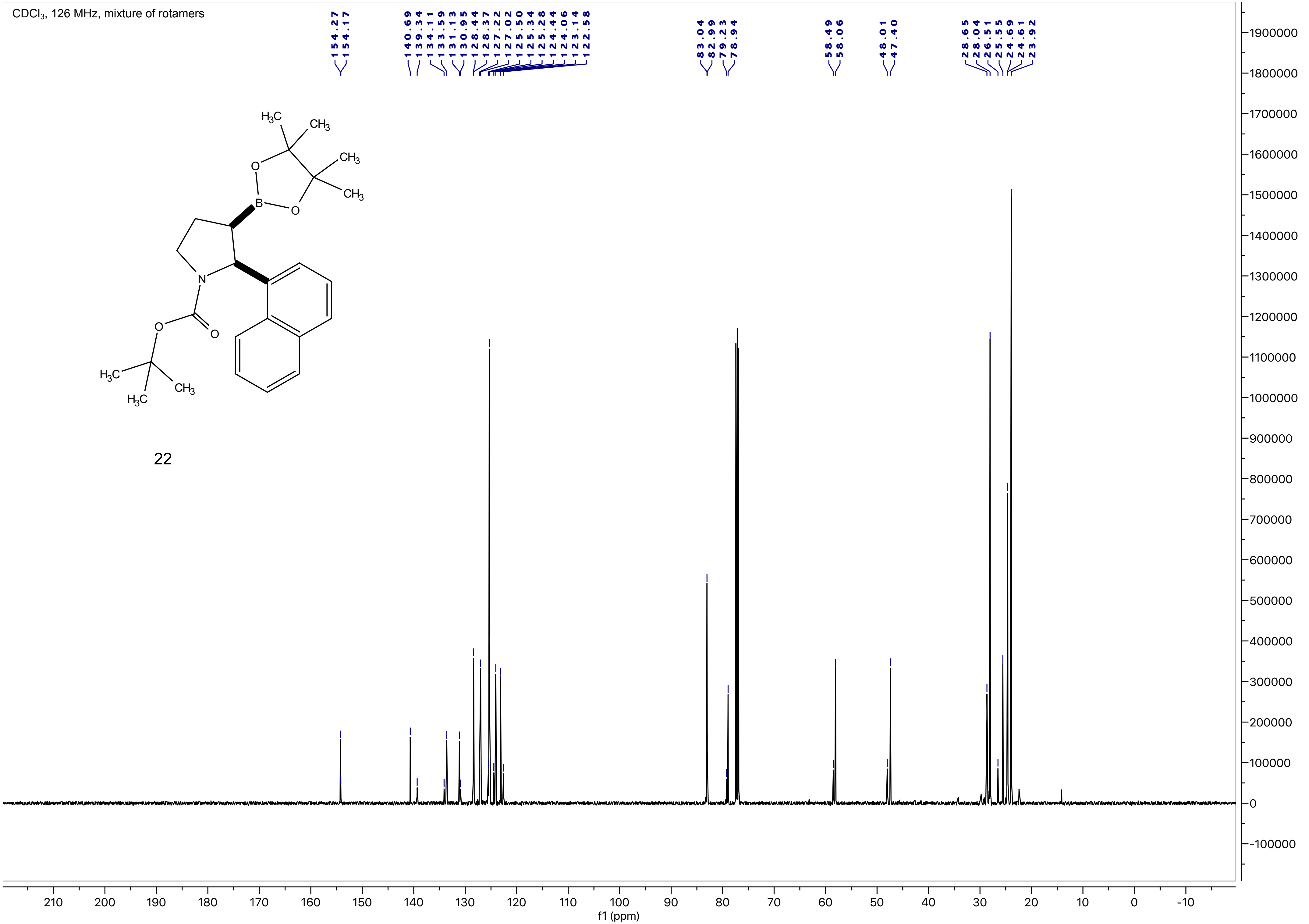
CDCl3, 126 MHz, mixture of rotamers
154.27
154.17
140.69
139.34
134.11
133.59
131.13
130.95
128.44
128.37
127.22
127.02
125.50
125.34
125.28
124.44
124.06
123.14
122.58
83.04
82.99
79.23
78.94
58.49
58.06
48.01
47.40
28.65
28.04
26.51
25.55
24.69
24.61
23.92
22
f1 (ppm)

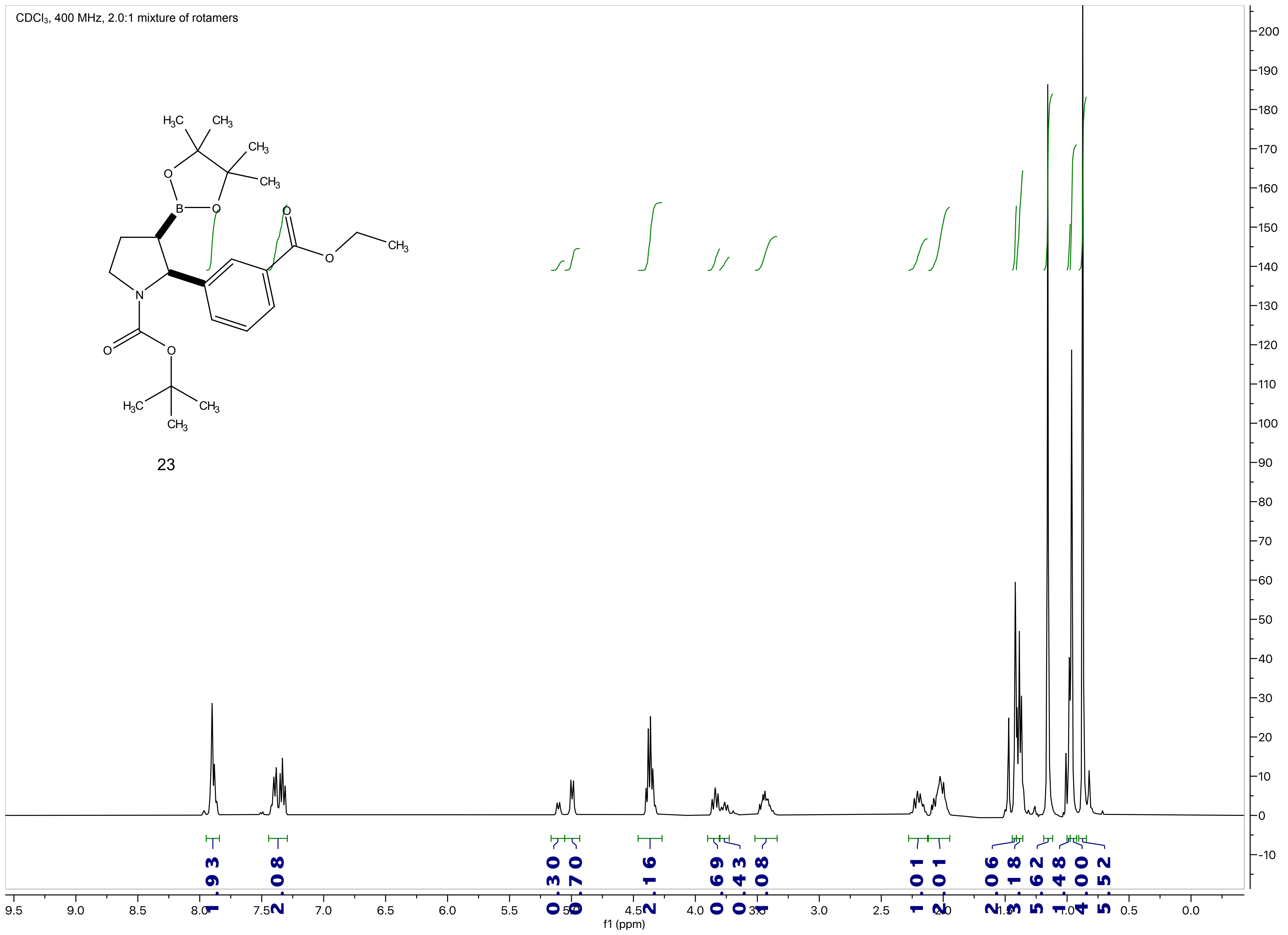
CDCl3, 400 MHz, 2.0:1 mixture of rotamers
23
1.93
2.08
0.30
0.70
2.16
0.69
0.43
1.08
1.01
2.01
2.06
3.18
5.62
1.48
4.00
5.52
f1 (ppm)

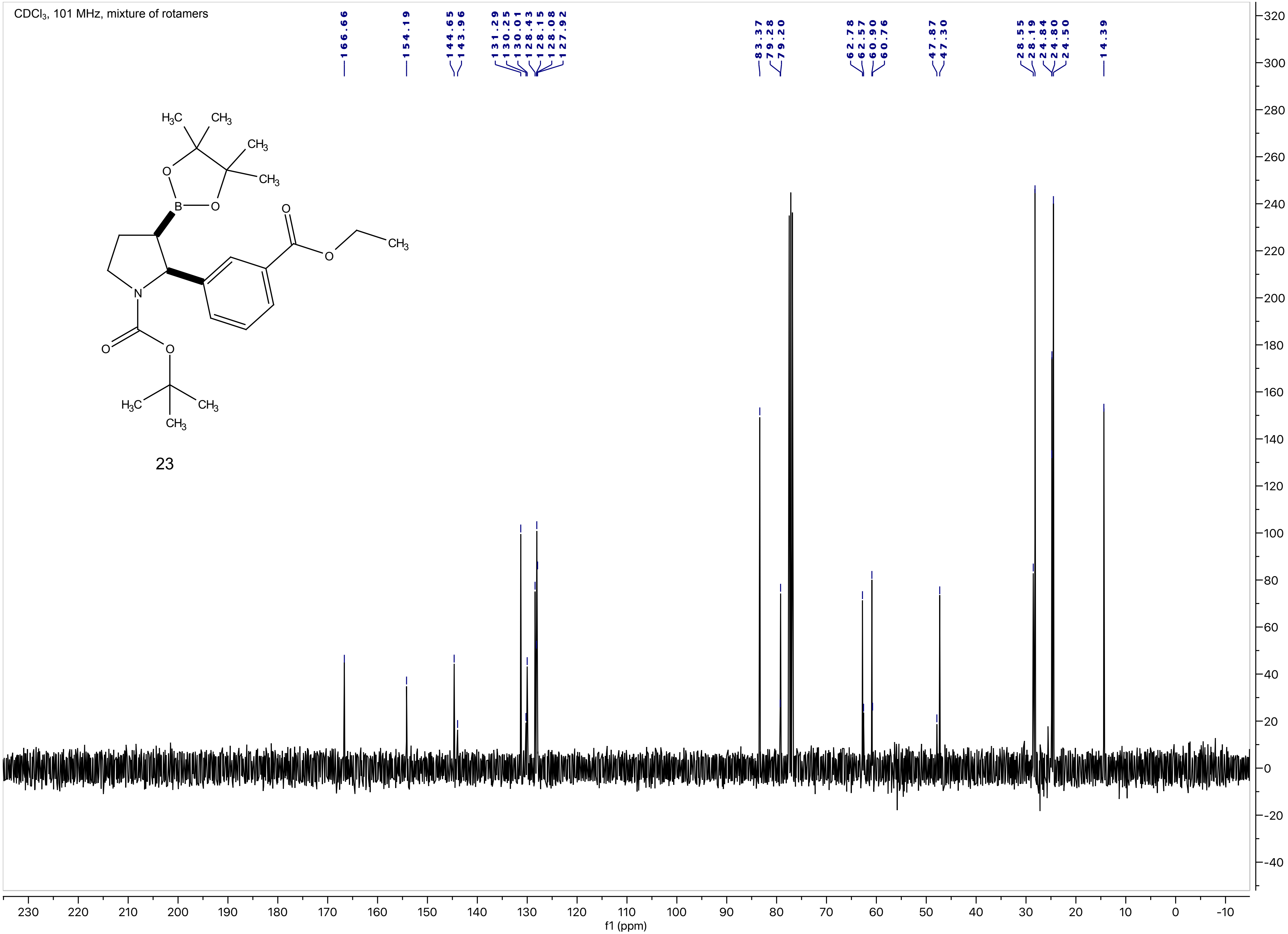

CDCl3, 101 MHz, mixture of rotamers
166.66
154.19
144.65
143.96
131.29
130.25
130.01
128.43
128.15
128.08
127.92
83.37
79.28
79.20
62.78
62.57
60.90
60.76
47.87
47.30
28.55
28.19
24.84
24.80
24.50
14.39
23
f1 (ppm)

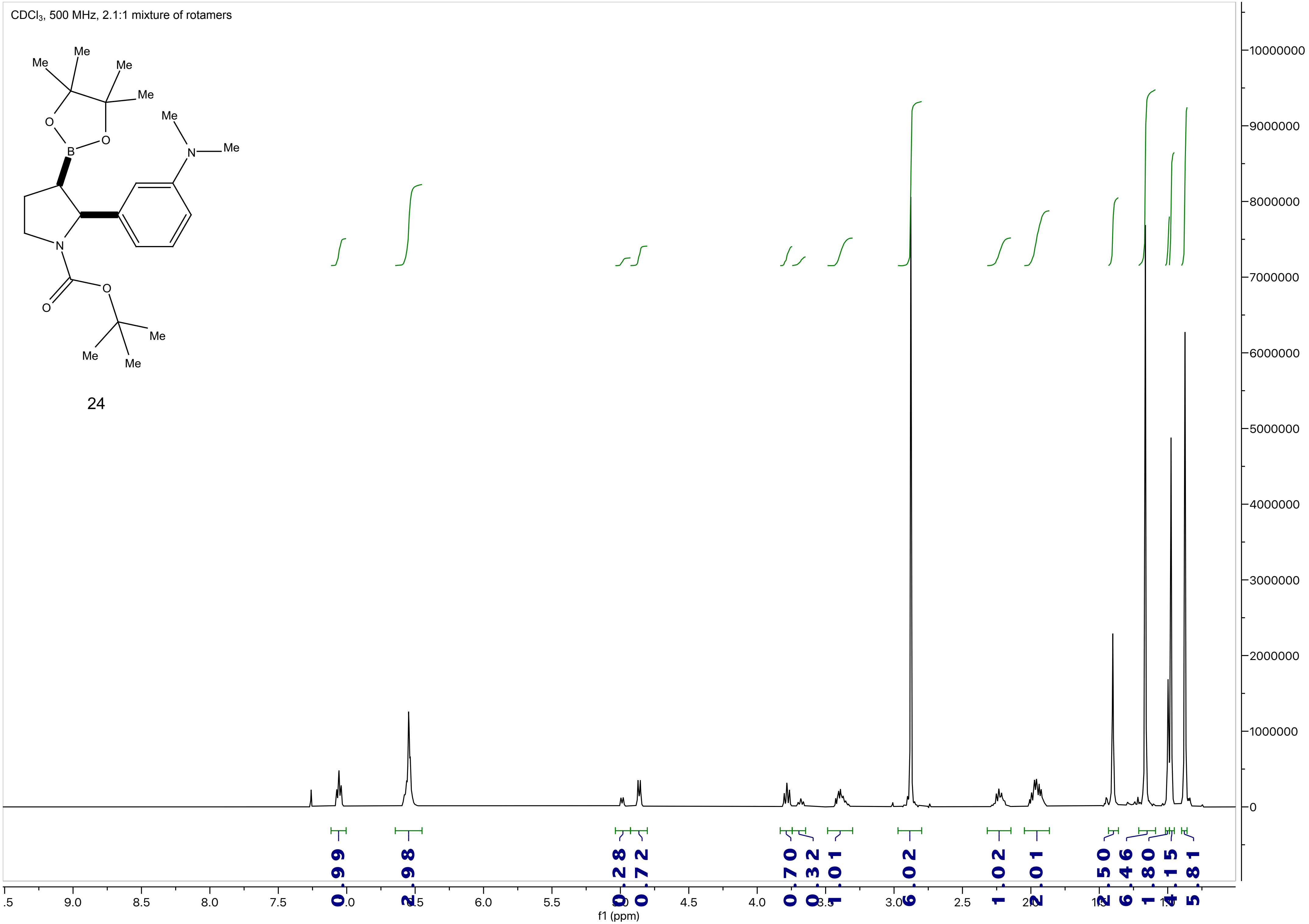
CDCl$_3$, 500 MHz, 2.1:1 mixture of rotamers
24
f1 (ppm)

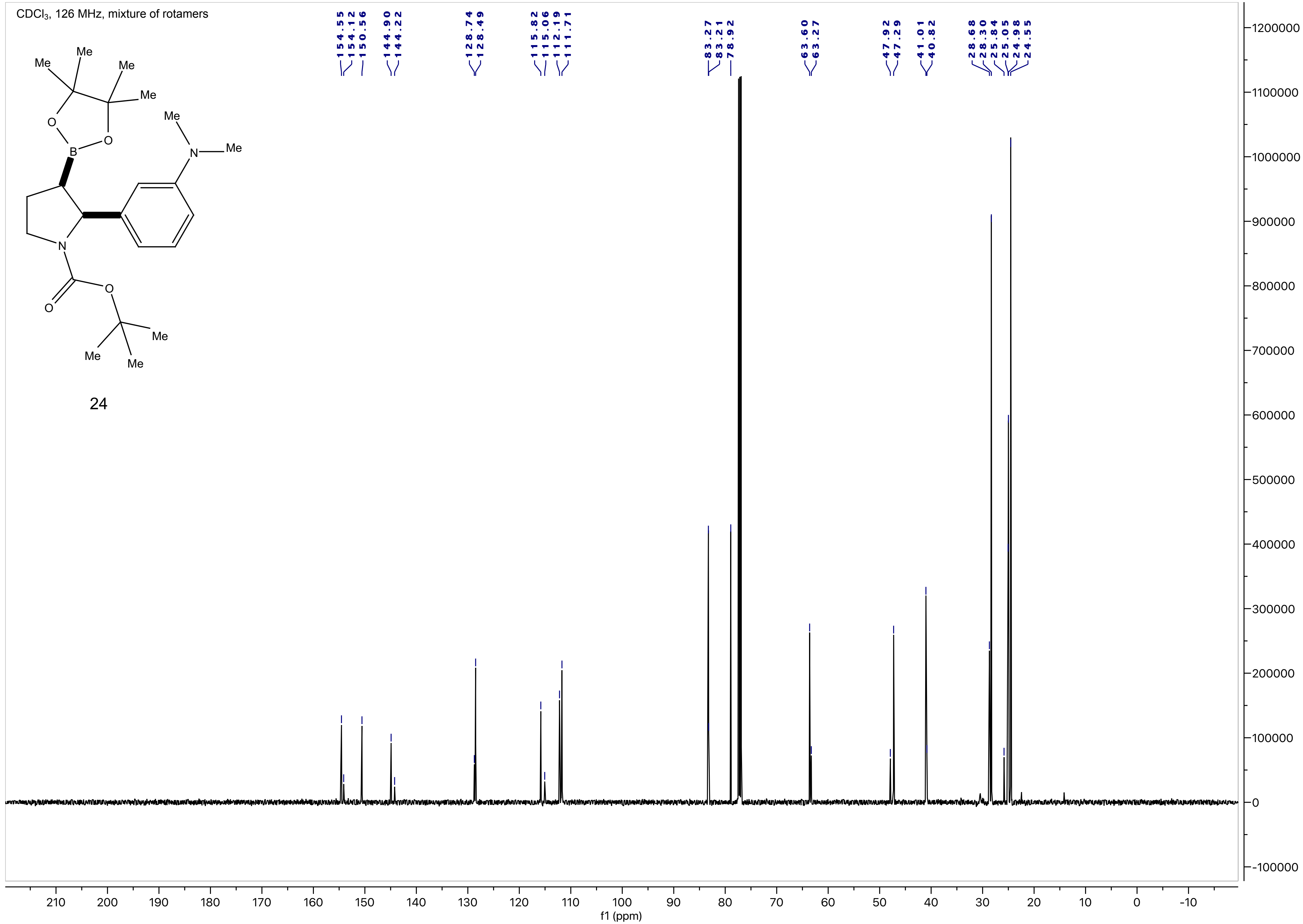
CDCl$_3$, 126 MHz, mixture of rotamers
154.55
154.12
150.56
144.90
144.22
128.74
128.49
115.82
115.06
112.19
111.71
83.27
83.21
78.92
63.60
63.27
47.92
47.29
41.01
40.82
28.68
28.30
25.84
25.05
24.98
24.55
24
f1 (ppm)

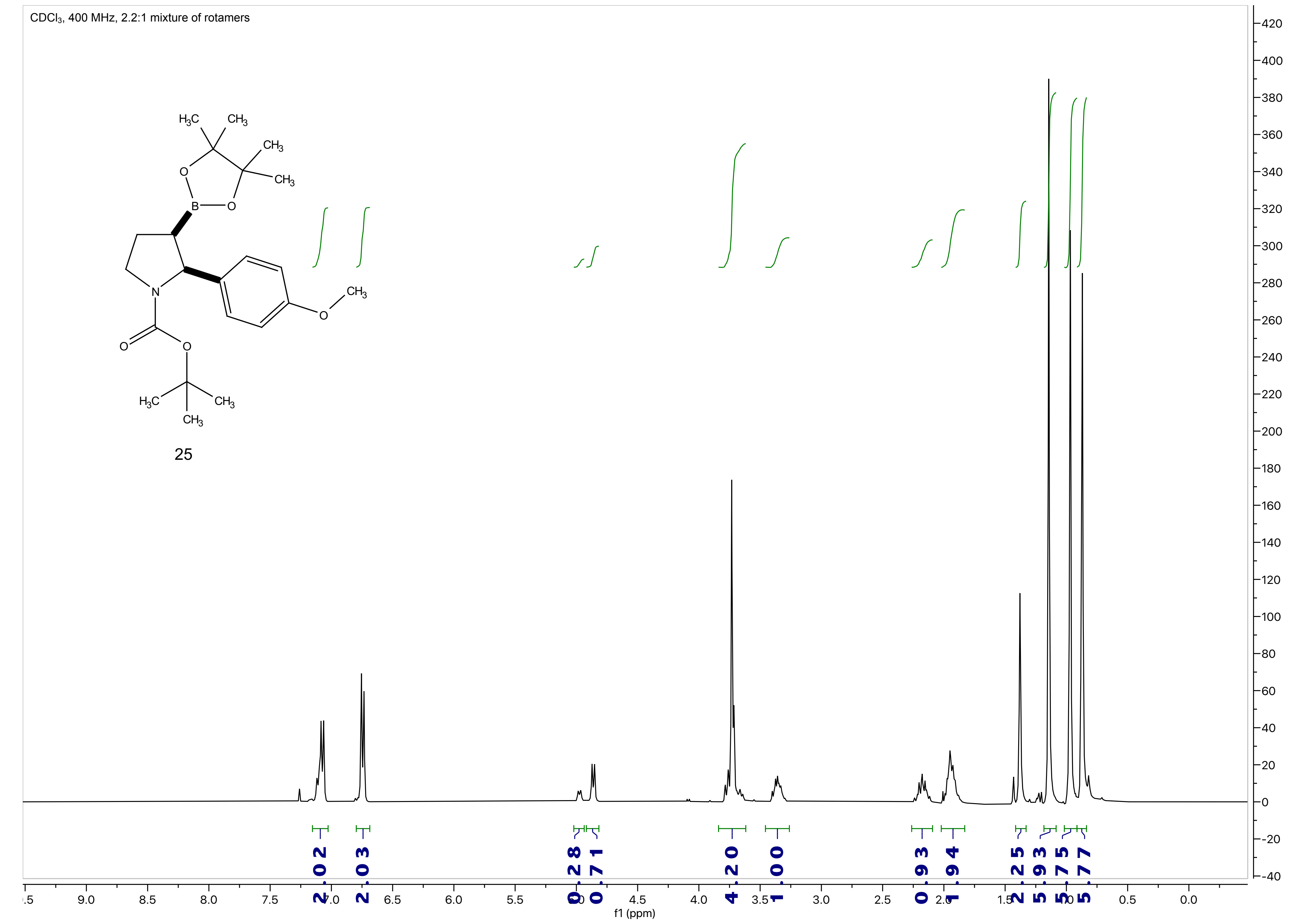
CDCl$_3$, 400 MHz, 2.2:1 mixture of rotamers
H_3C
CH_3
CH_3
CH_3
O
B
O
N
CH_3
O
O
O
H_3C
CH_3
CH_3
25
2.02
2.03
0.28
0.71
4.20
1.00
0.93
1.94
2.25
5.93
5.75
5.77
f1 (ppm)

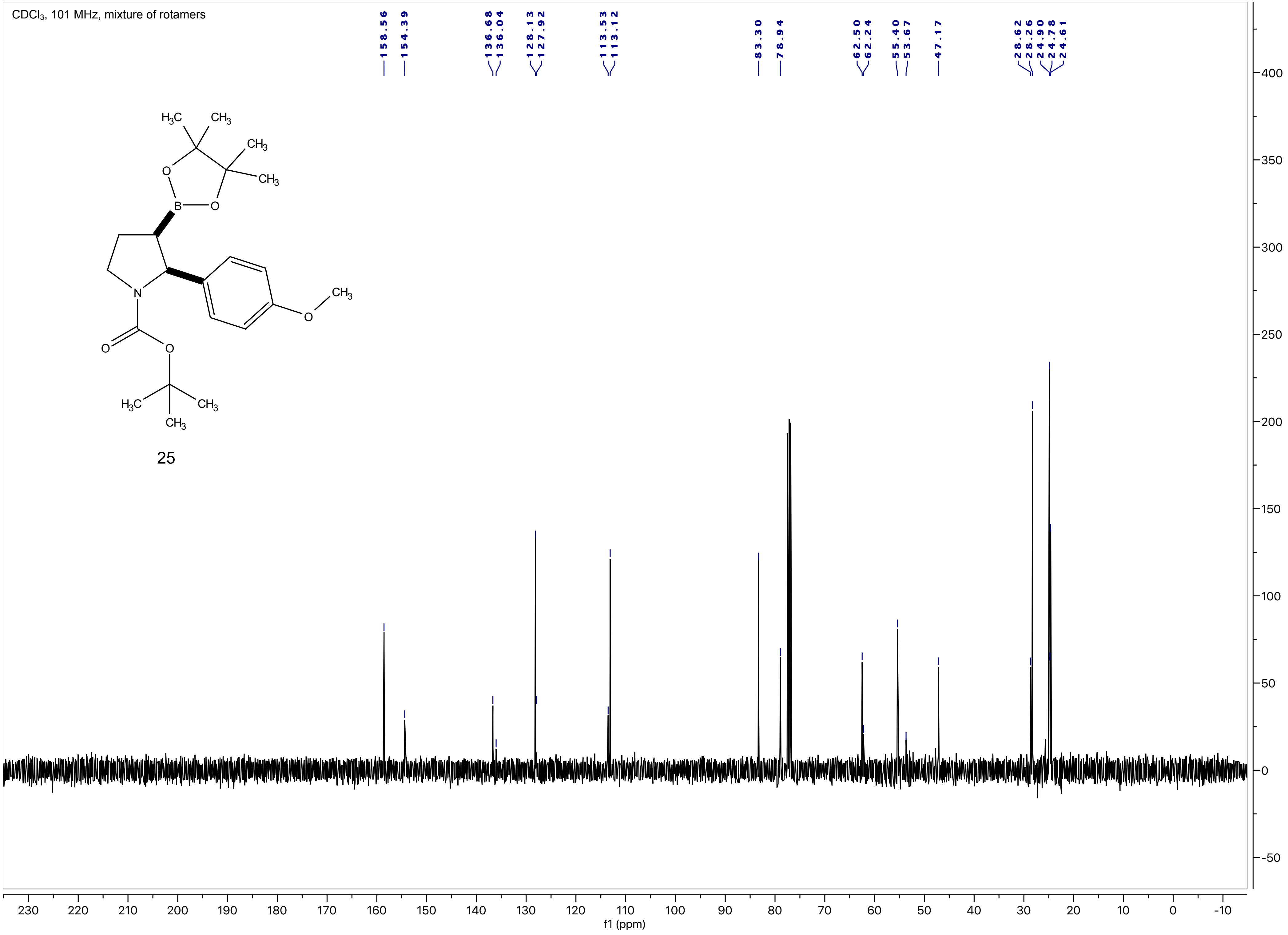

CDCl₃, 101 MHz, mixture of rotamers
158.56
154.39
136.68
136.04
128.13
127.92
113.53
113.12
83.30
78.94
62.50
62.24
55.40
53.67
47.17
28.62
28.26
24.90
24.78
24.61
25
f1 (ppm)

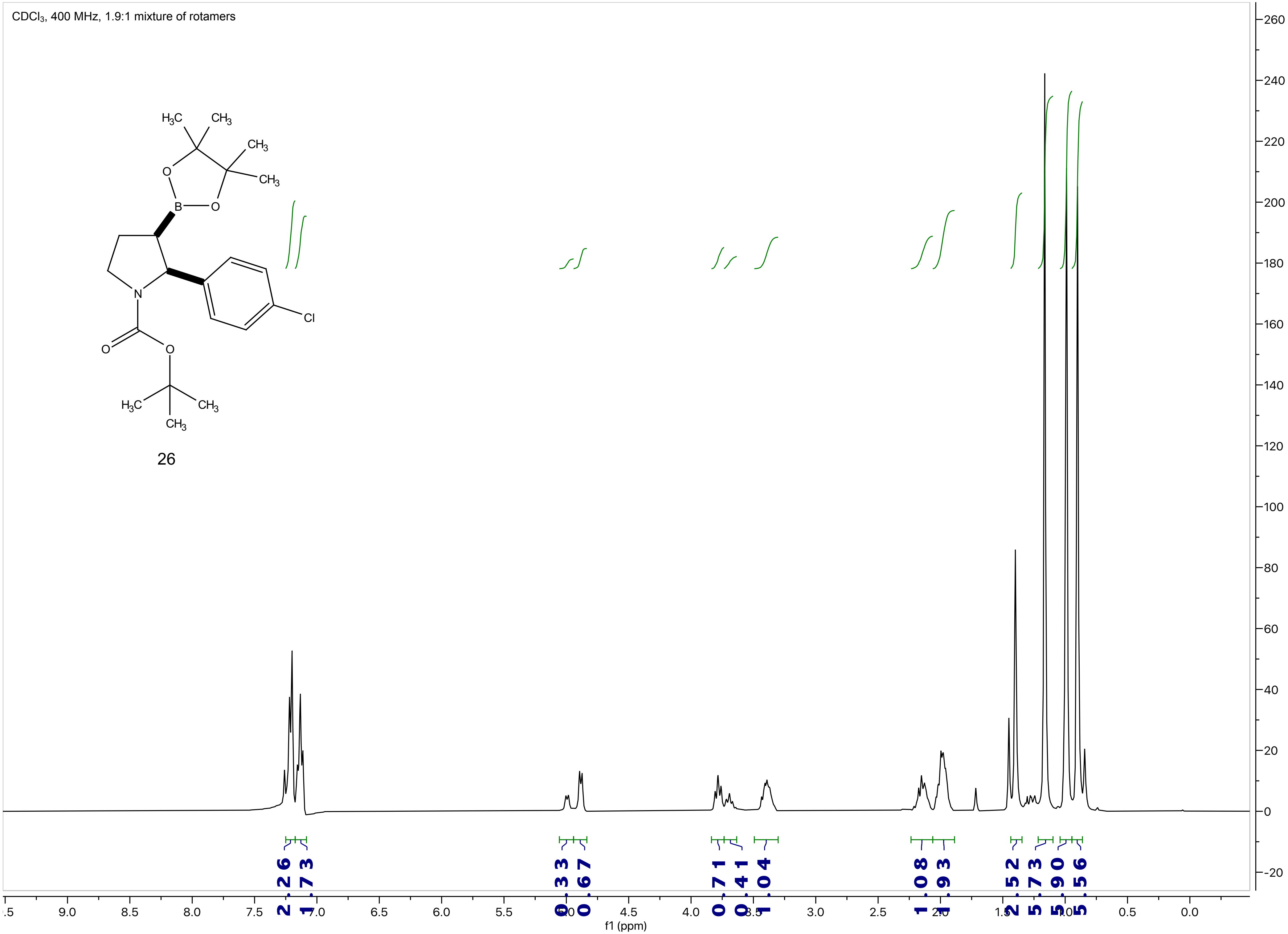
CDCl3, 400 MHz, 1.9:1 mixture of rotamers
26
2.26
1.73
0.33
0.67
0.71
0.41
1.04
1.08
1.93
2.52
5.73
5.90
5.56
f1 (ppm)

CDCl$_3$, 101 MHz, mixture of rotamers

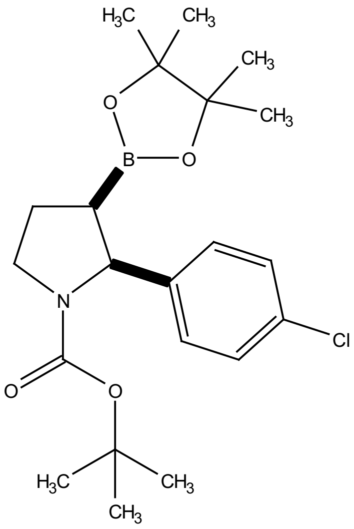

26

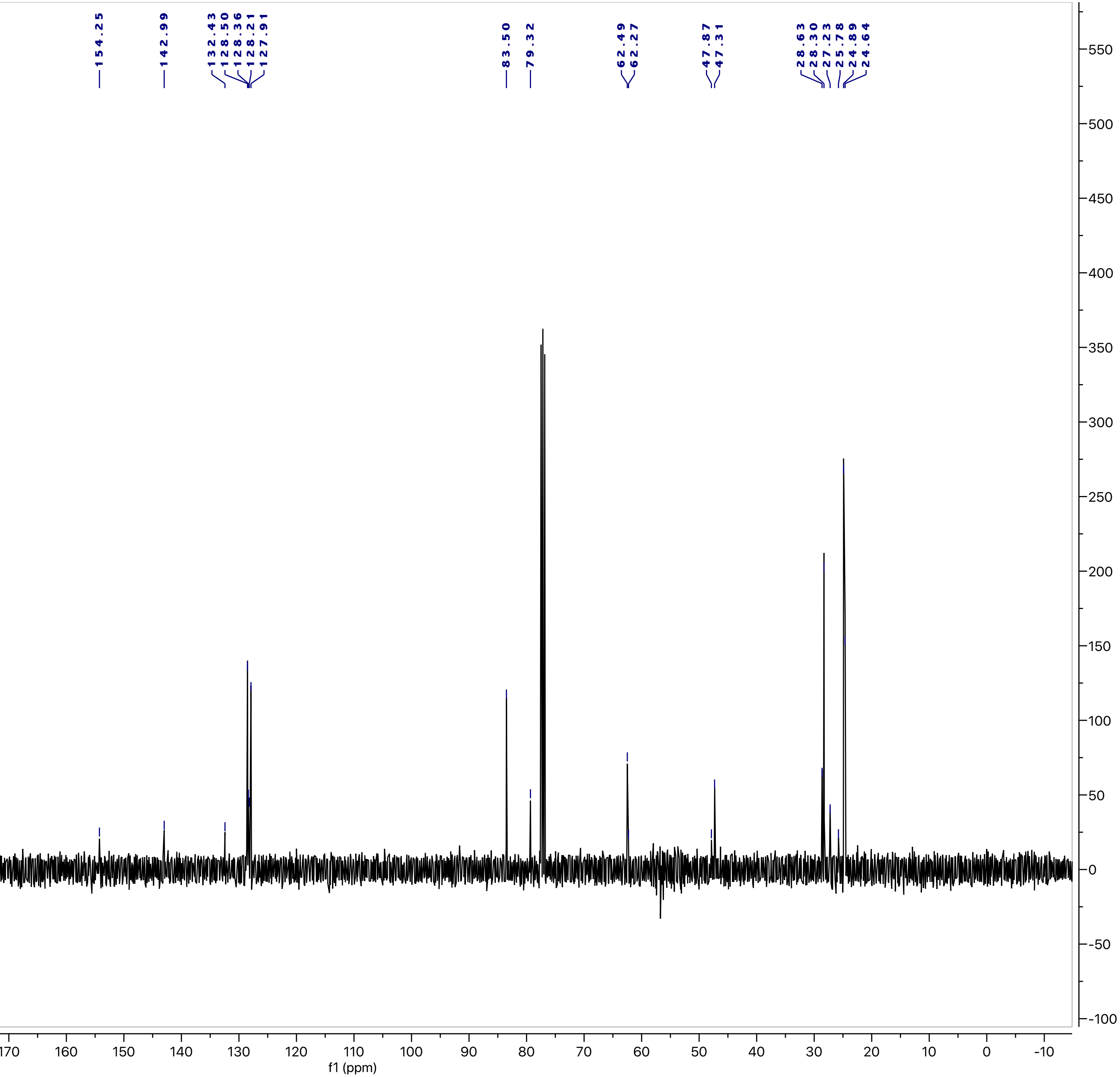

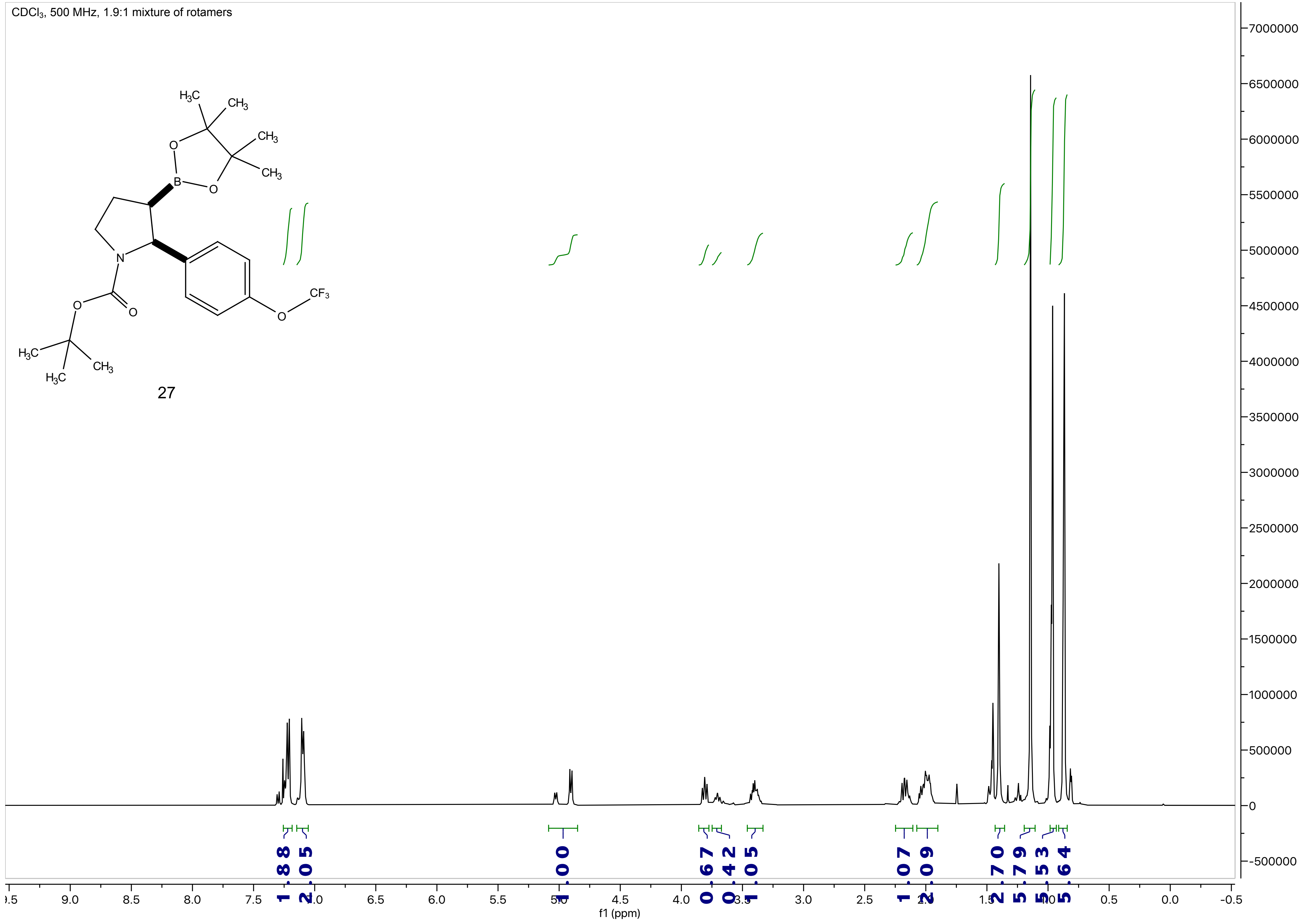
CDCl3, 500 MHz, 1.9:1 mixture of rotamers
27
1.88
2.05
1.00
0.67
0.42
1.05
1.07
2.09
2.70
5.79
5.53
5.64
f1 (ppm)

CDCl3, 126 MHz, mixture of rotamers

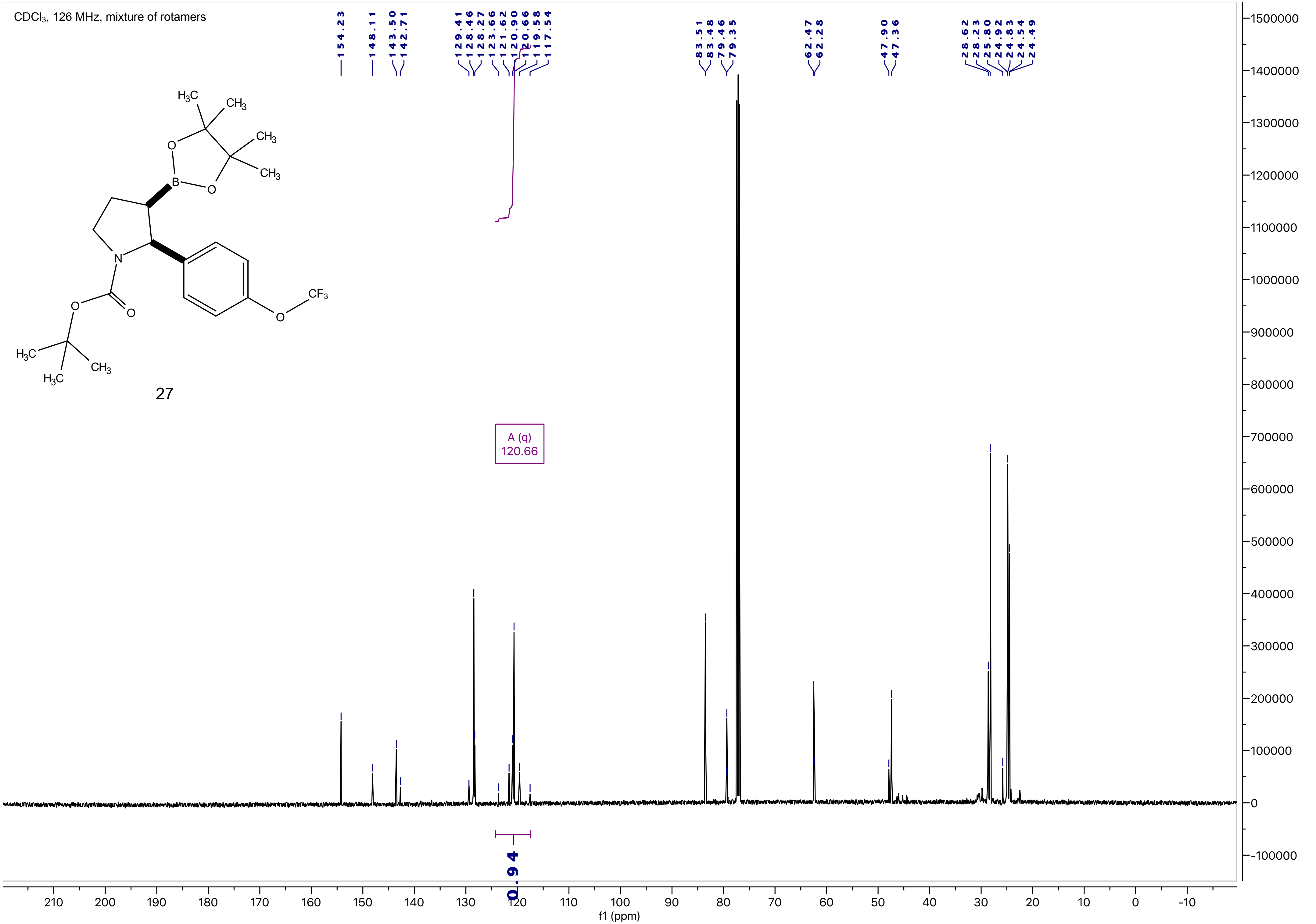

27

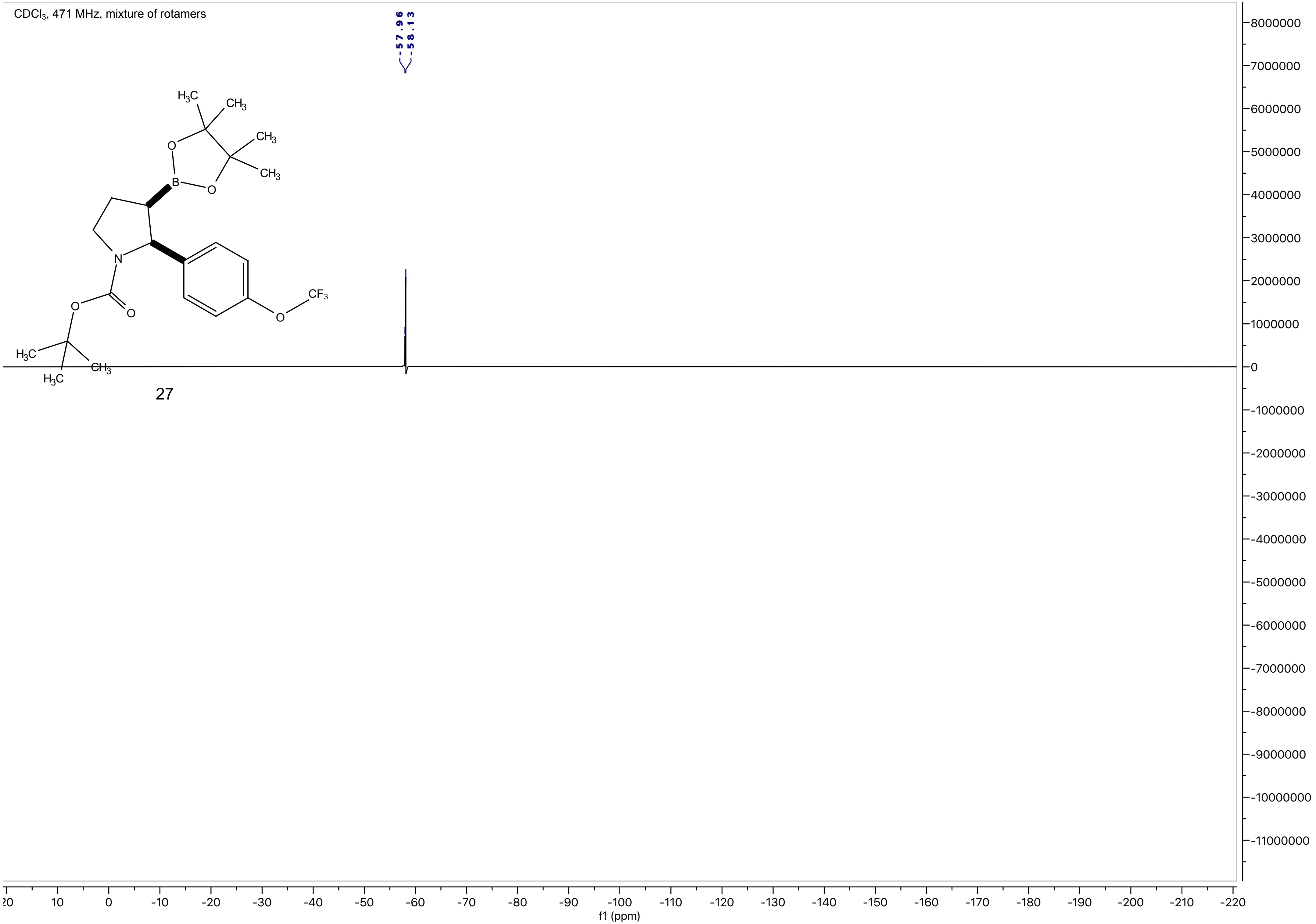

CDCl3, 471 MHz, mixture of rotamers
-57.96
-58.13
27
f1 (ppm)

CDCl$_3$, 500 MHz, 2.2:1 mixture of rotamers

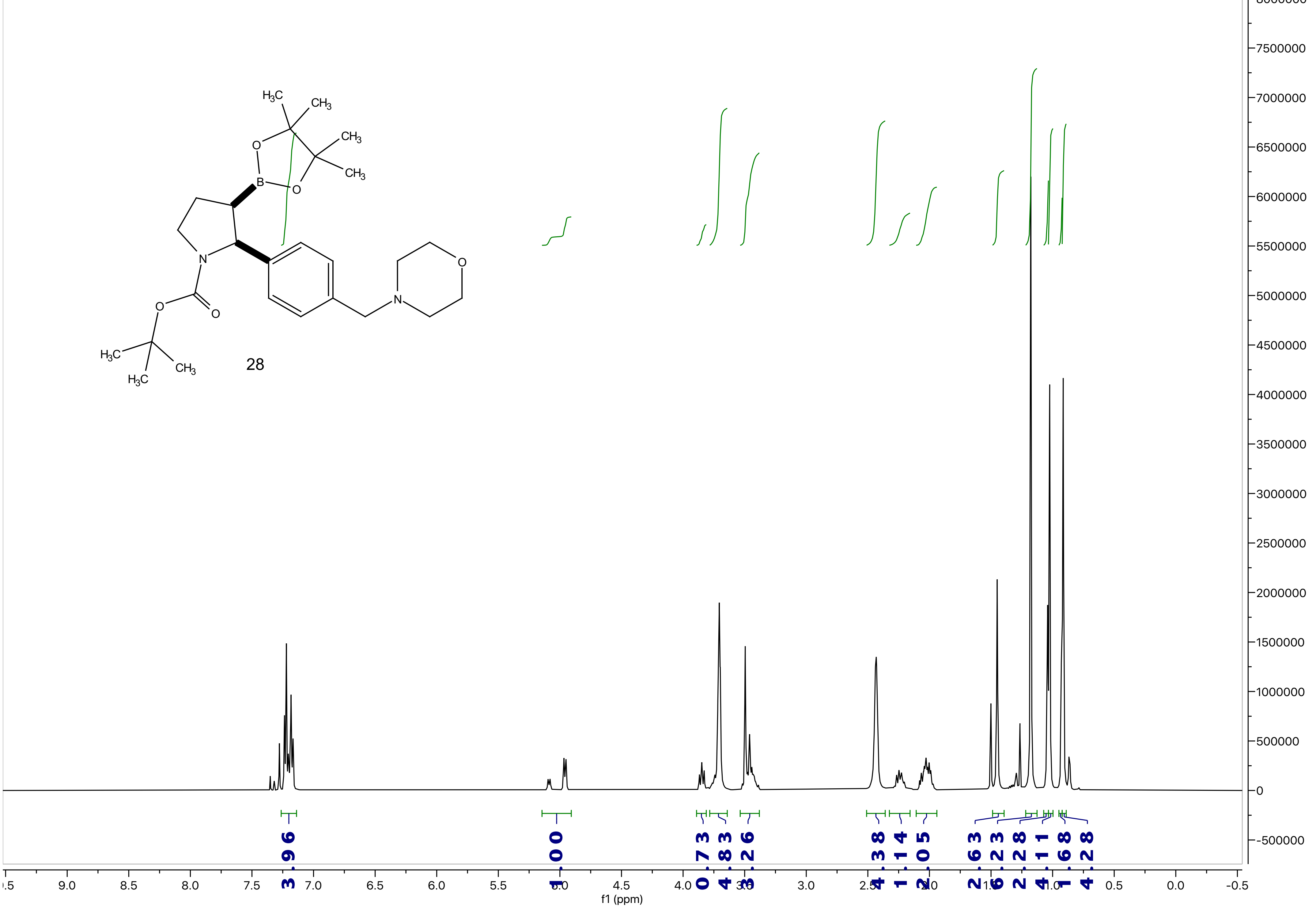

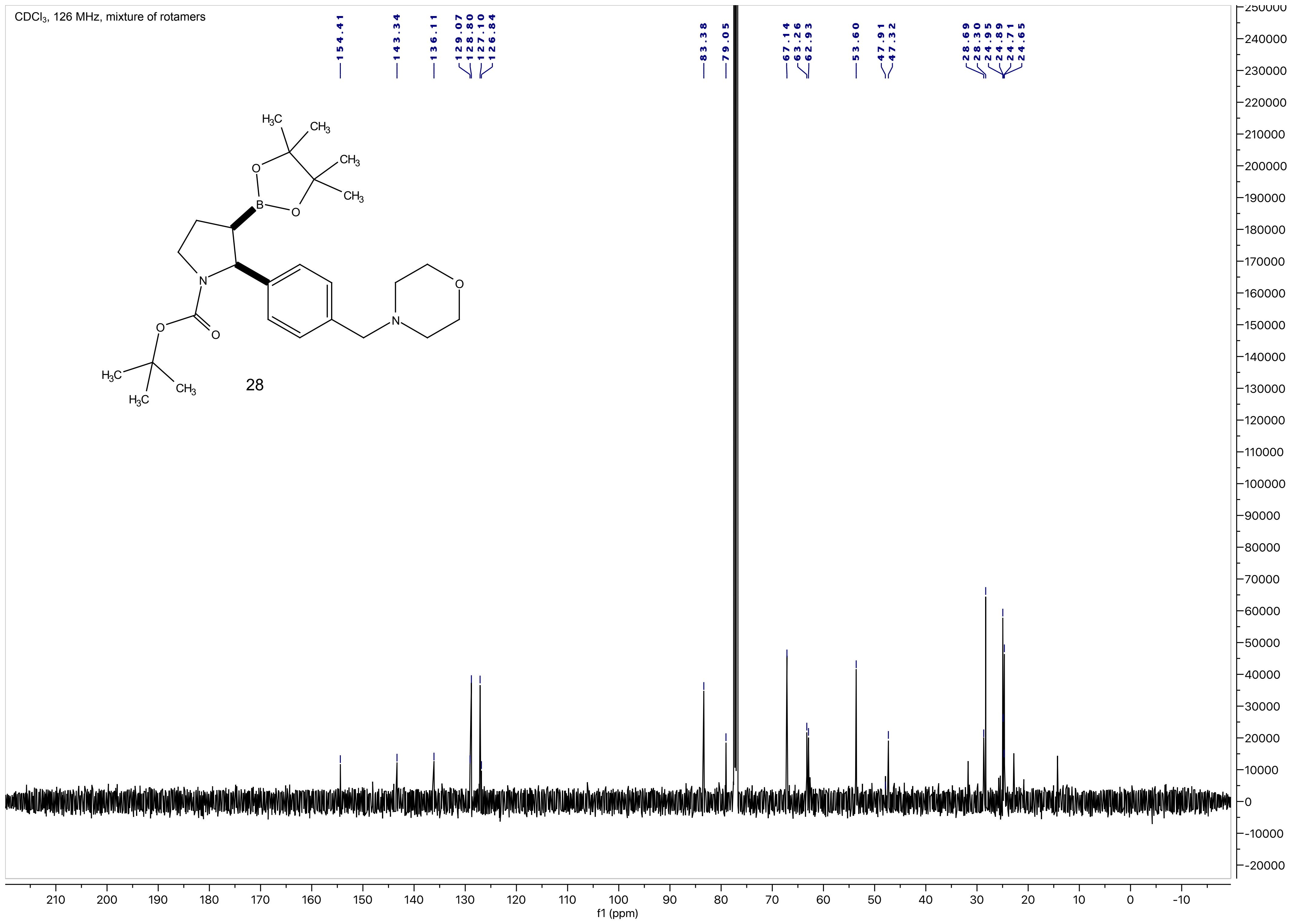

CDCl3, 126 MHz, mixture of rotamers
154.41
143.34
136.11
129.07
128.80
127.10
126.84
83.38
79.05
67.14
63.26
62.93
53.60
47.91
47.32
28.69
28.30
24.95
24.89
24.71
24.65
28
f1 (ppm)

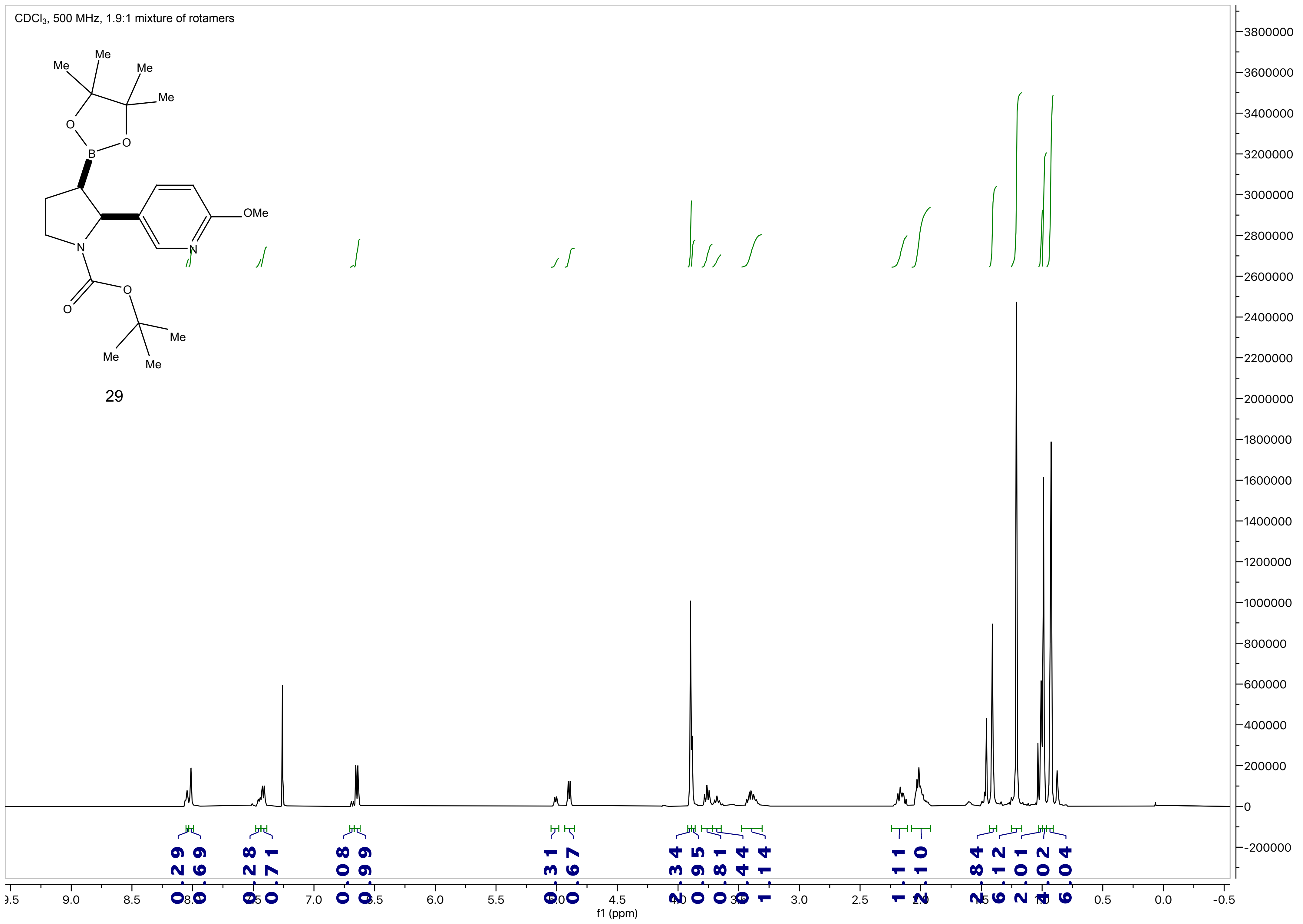
CDCl3, 500 MHz, 1.9:1 mixture of rotamers
Me
Me
Me
Me
O
O
B
OMe
N
N
O
O
Me
Me
Me
29
f1 (ppm)

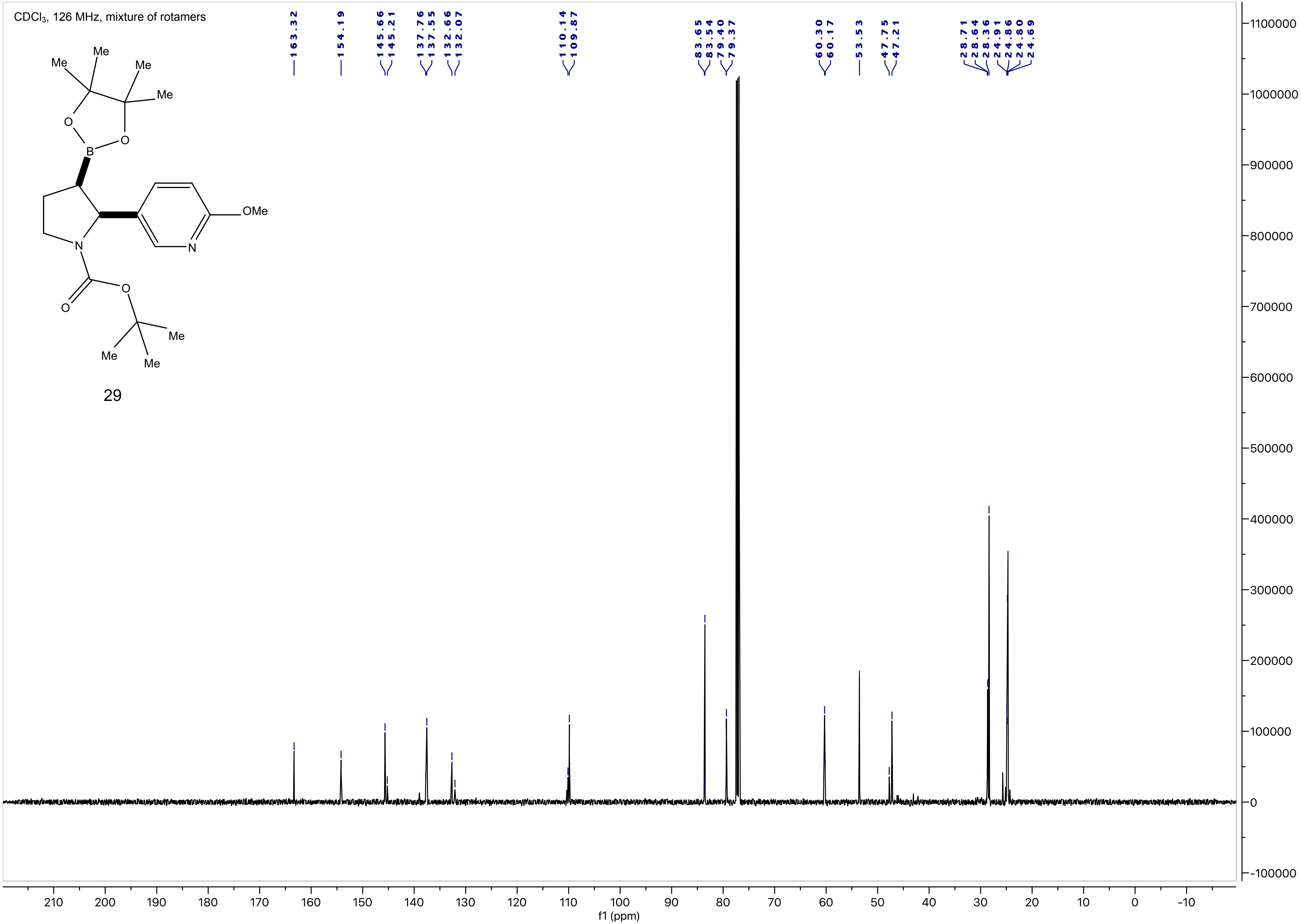
CDCl3, 126 MHz, mixture of rotamers
163.32
154.19
145.66
145.21
137.76
137.55
132.66
132.07
110.14
109.87
83.65
83.54
79.40
79.37
60.30
60.17
53.53
47.75
47.21
28.71
28.64
28.36
24.91
24.86
24.80
24.69
Me
O
B
N
OMe
29
f1 (ppm)

CDCl$_3$, 500 MHz, 2.1:1 mixture of rotamers

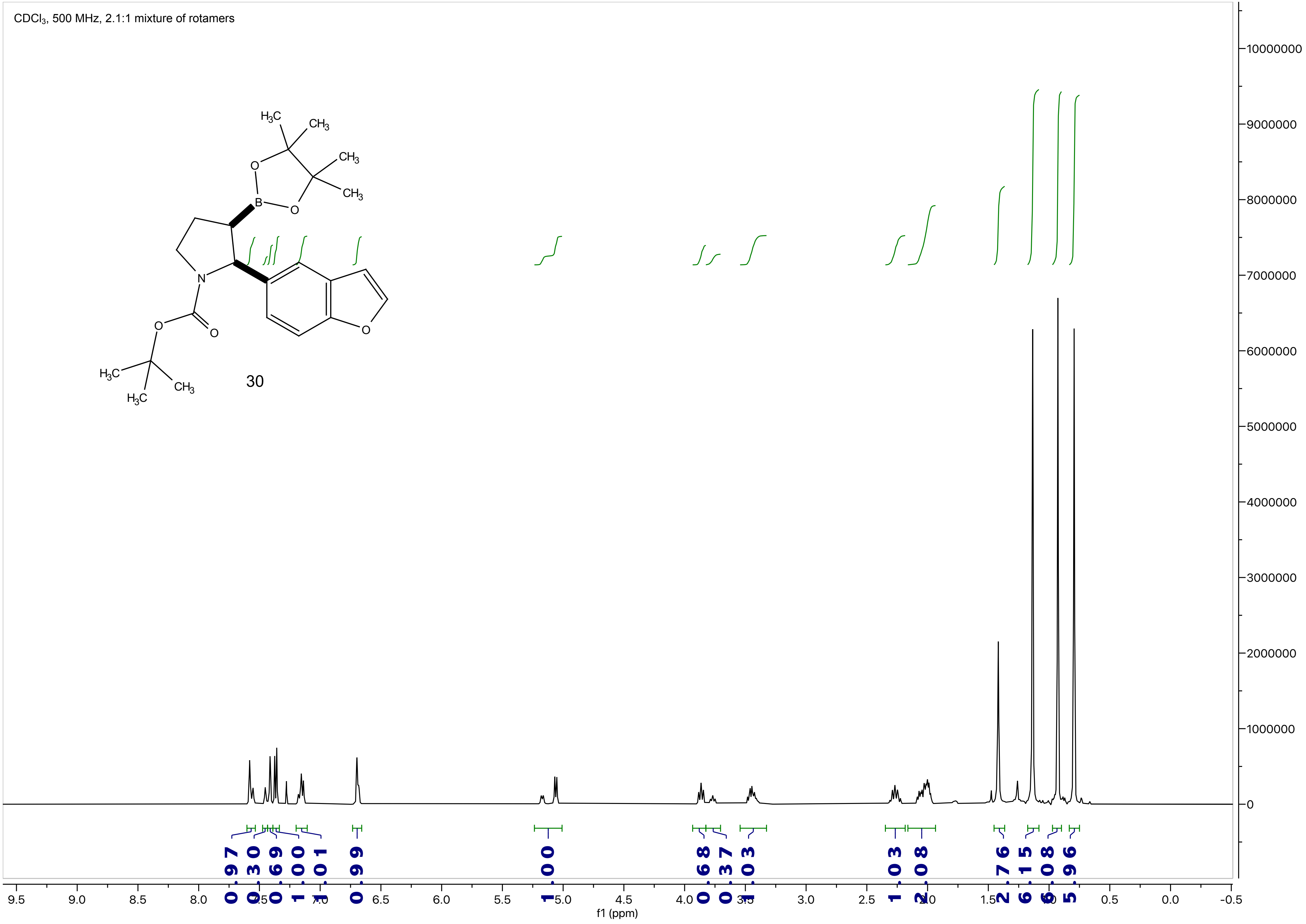

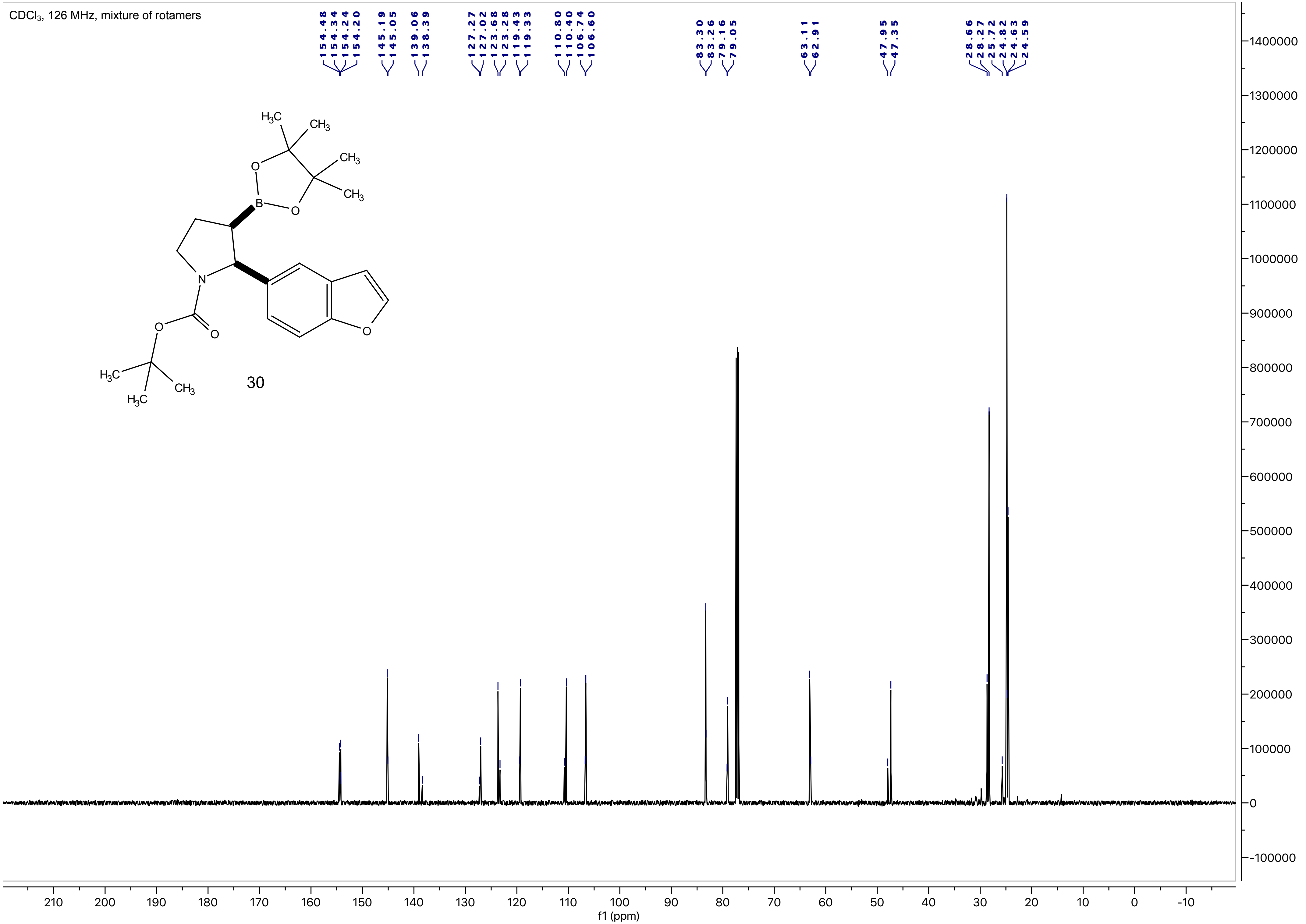

CDCl3, 126 MHz, mixture of rotamers
154.48
154.34
154.24
154.20
145.19
145.05
139.06
138.39
127.27
127.02
123.68
123.28
119.43
119.33
110.80
110.40
106.74
106.60
83.30
83.26
79.16
79.05
63.11
62.91
47.95
47.35
28.66
28.27
25.72
24.82
24.63
24.59
H3C
CH3
CH3
CH3
O
B
O
N
O
O
O
H3C
CH3
H3C
30
f1 (ppm)

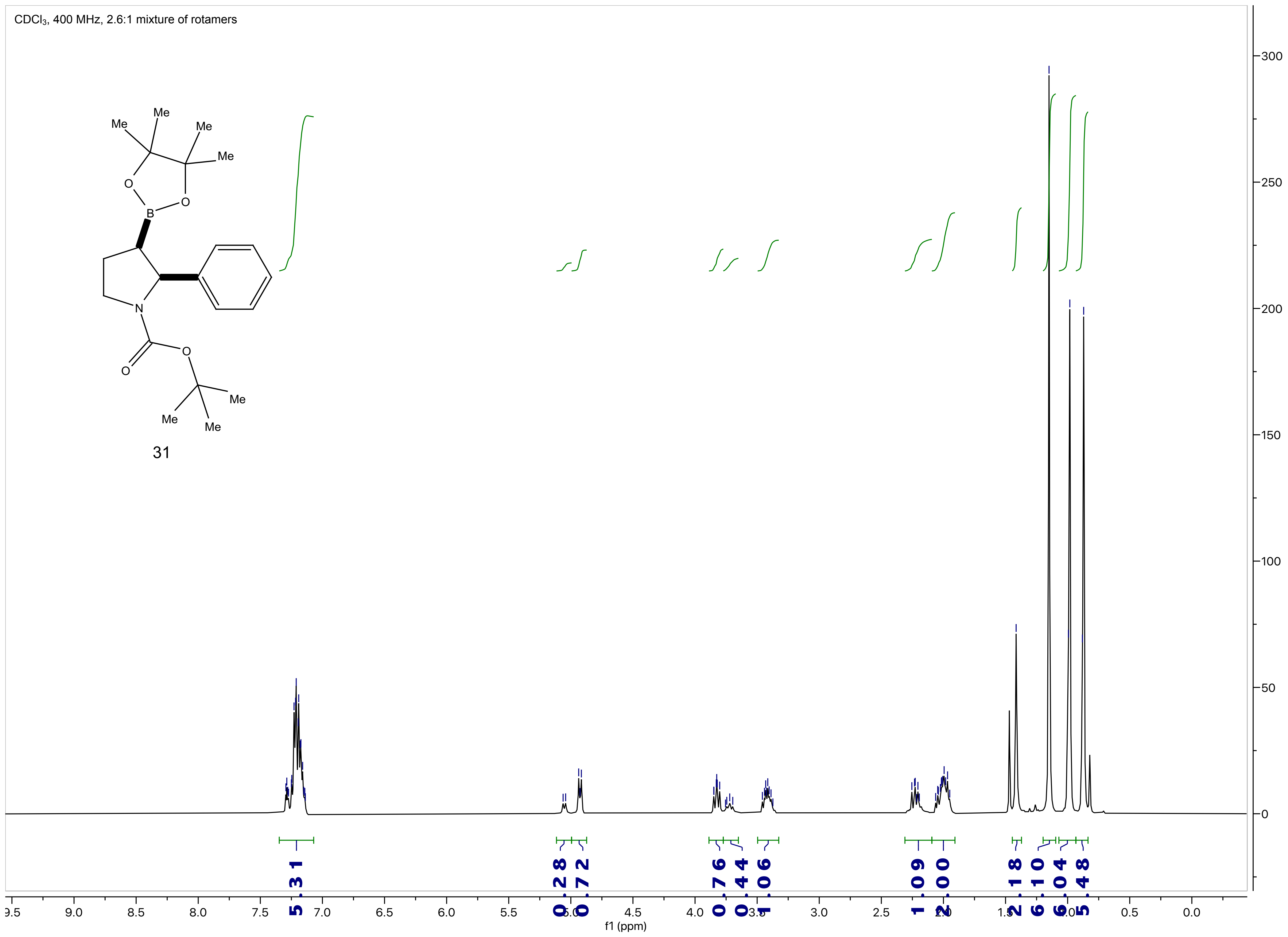

CDCl3, 400 MHz, 2.6:1 mixture of rotamers
31
5.31
0.28
0.72
0.76
0.44
1.06
1.09
2.00
2.18
6.10
6.04
5.48
f1 (ppm)

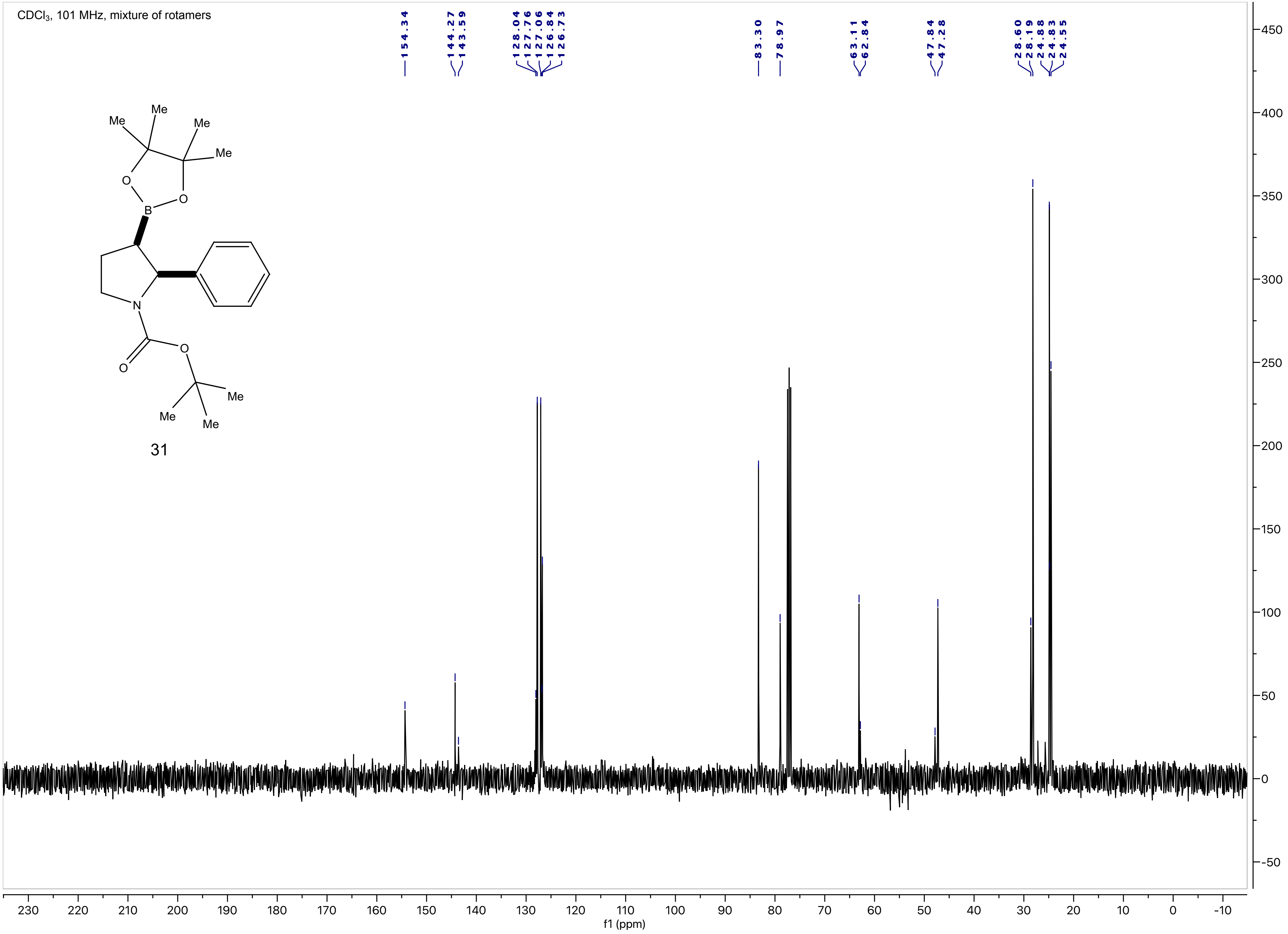
CDCl3, 101 MHz, mixture of rotamers
154.34
144.27
143.59
128.04
127.76
127.06
126.84
126.73
83.30
78.97
63.11
62.84
47.84
47.28
28.60
28.19
24.88
24.83
24.55
Me
Me
Me
Me
O
O
B
N
O
O
Me
Me
Me
31
f1 (ppm)
230 220 210 200 190 180 170 160 150 140 130 120 110 100 90 80 70 60 50 40 30 20 10 0 -10
450 400 350 300 250 200 150 100 50 0 -50

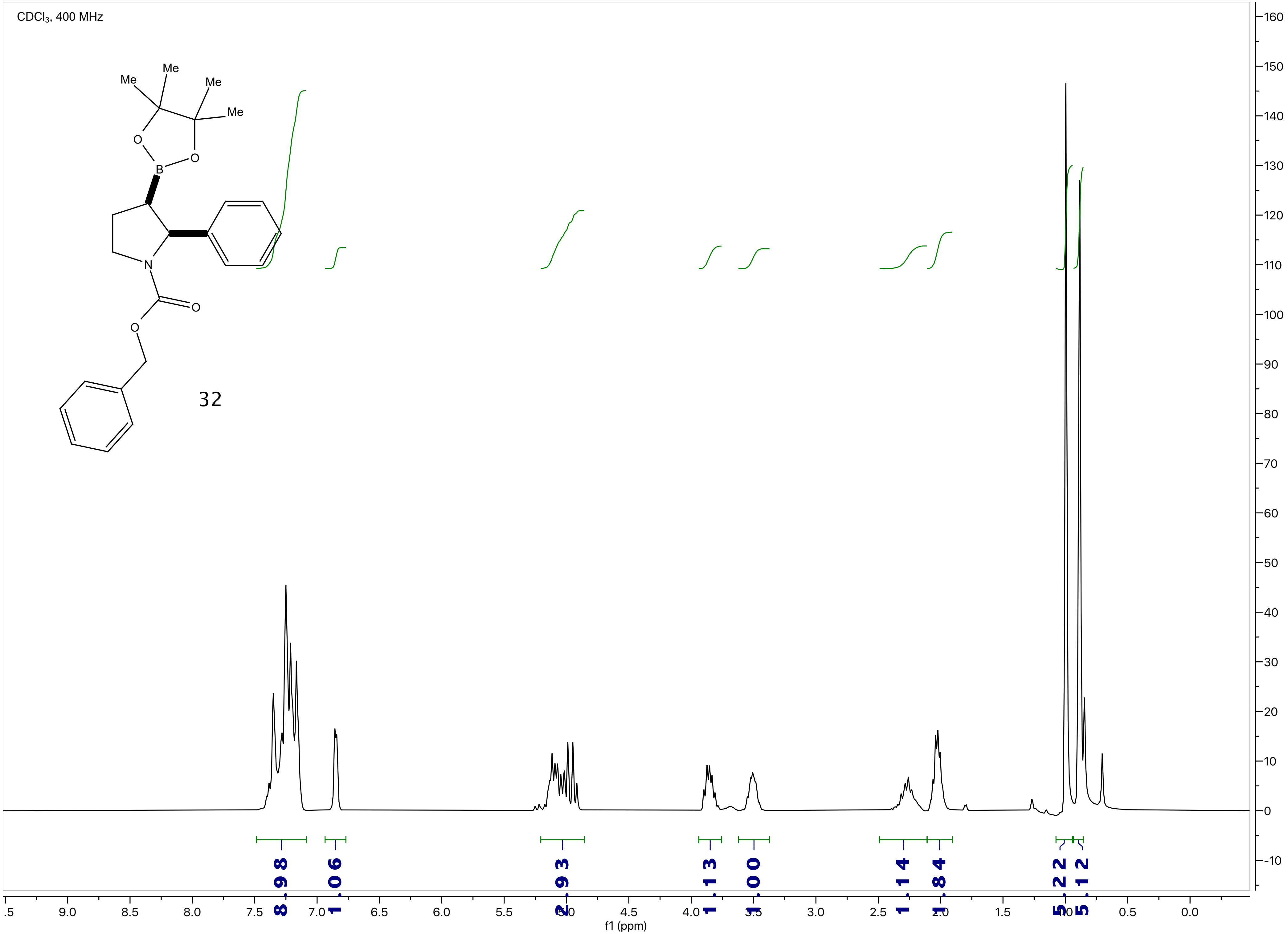
CDCl3, 400 MHz
Me
Me
Me
Me
O
O
B
N
O
O
32
8.98
1.06
2.93
1.13
1.00
1.14
1.84
5.22
5.12
f1 (ppm)

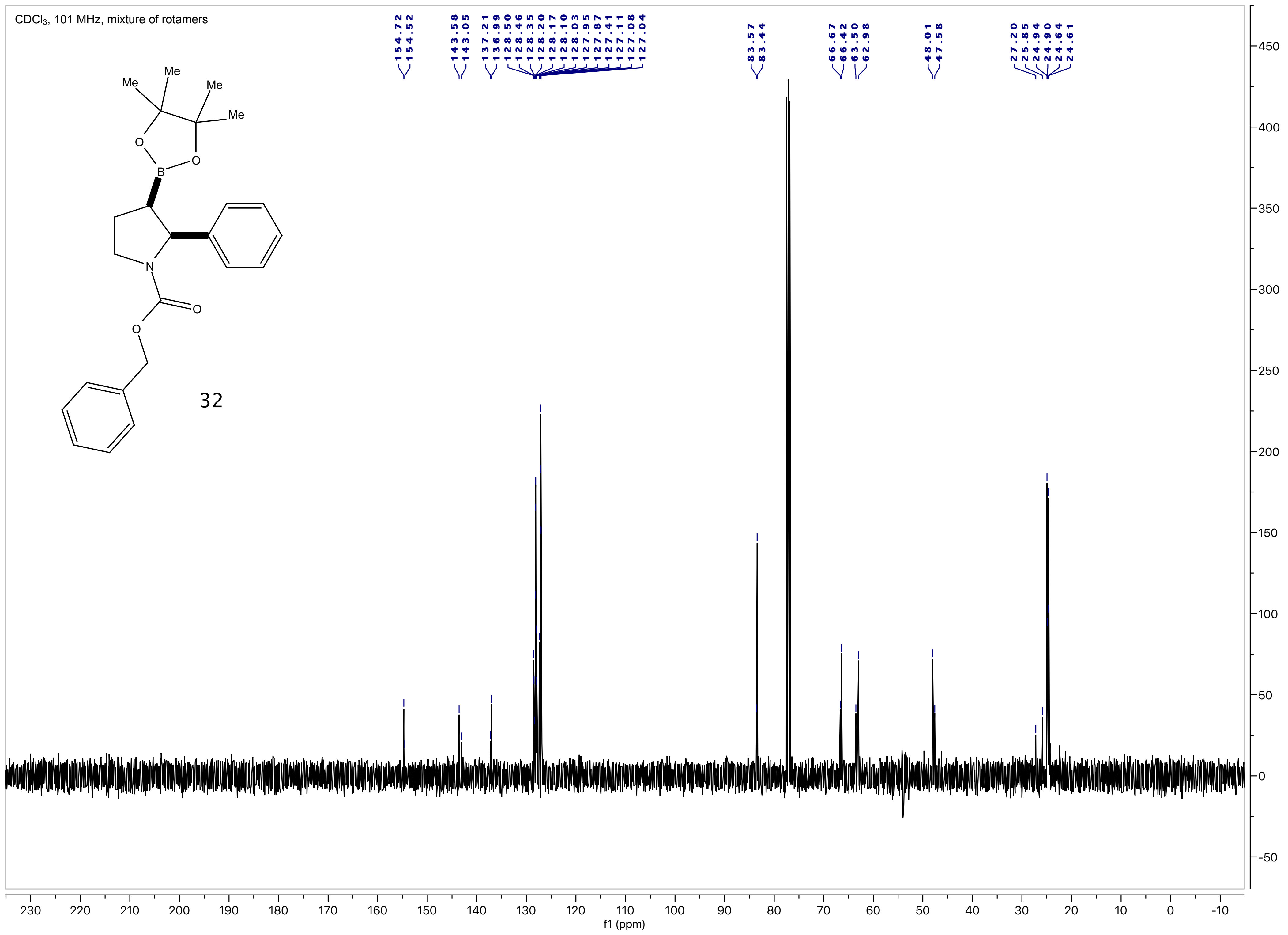

CDCl3, 101 MHz, mixture of rotamers
154.72
154.52
143.58
143.05
137.21
136.99
128.50
128.46
128.35
128.20
128.17
128.10
128.03
127.95
127.87
127.41
127.11
127.08
127.04
83.57
83.44
66.67
66.42
63.50
62.98
48.01
47.58
27.20
25.85
24.94
24.90
24.64
24.61
Me
Me
Me
Me
O
O
B
N
O
O
32
f1 (ppm)

CDCl$_3$, 500 MHz, mixture of rotamers

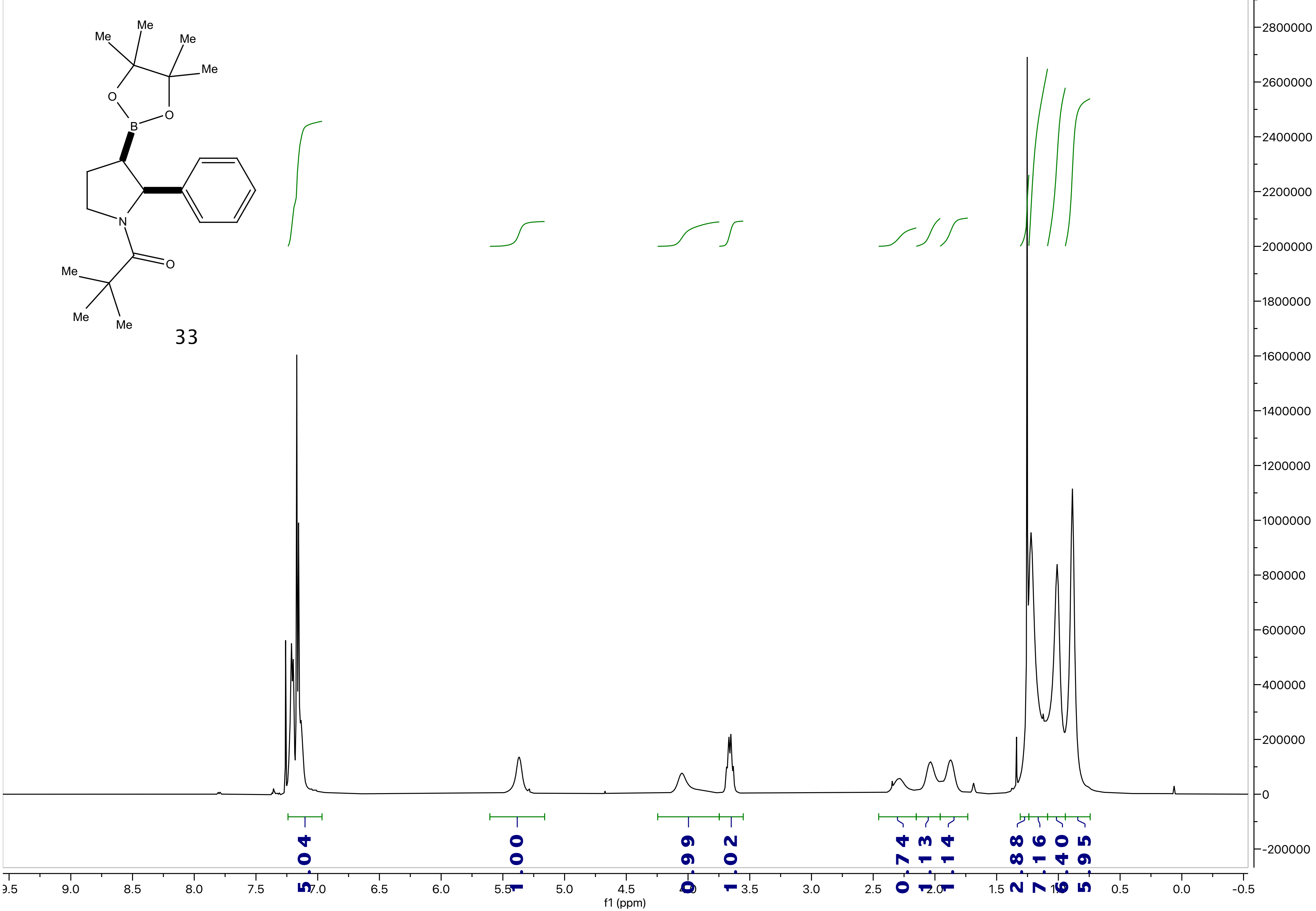

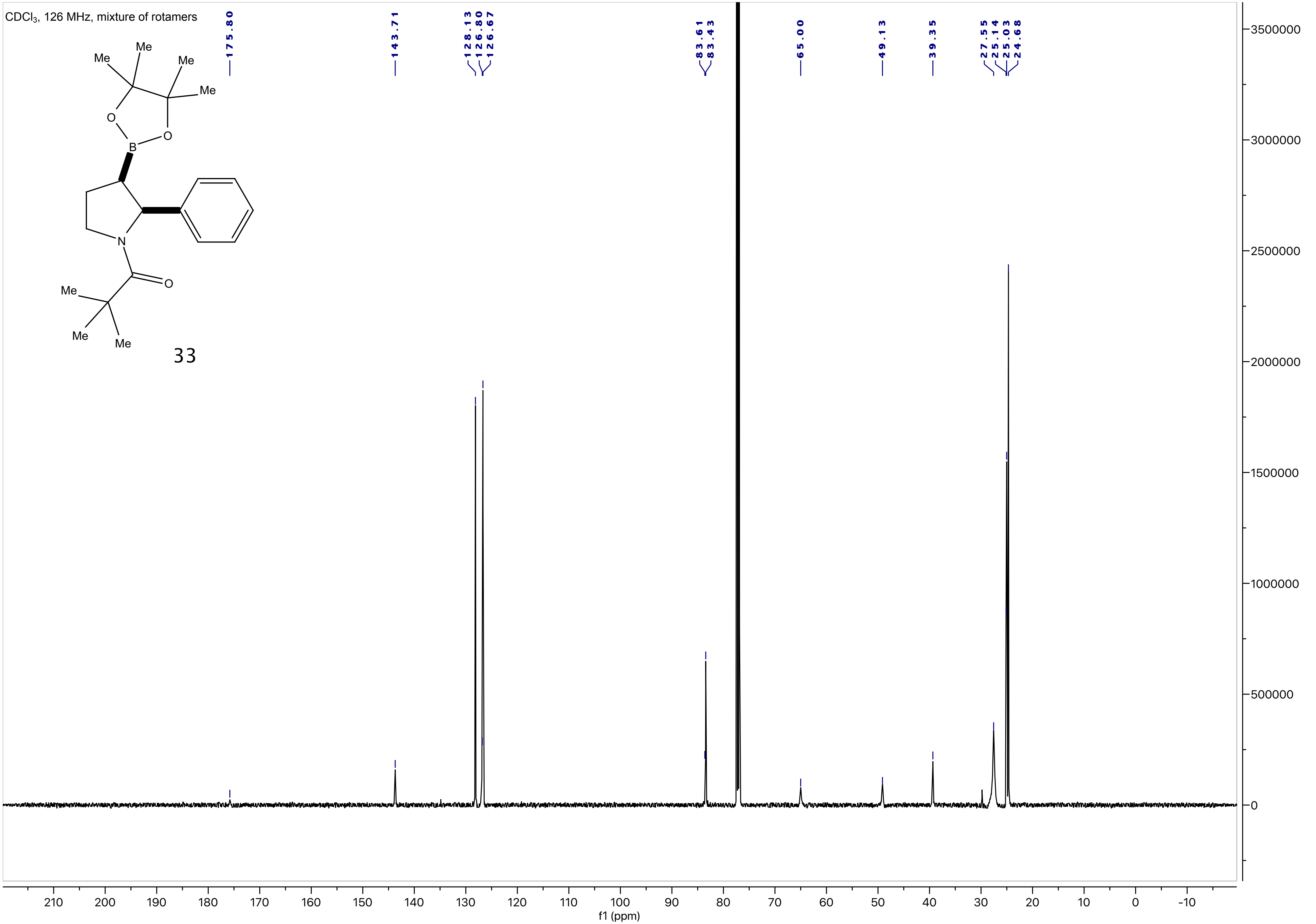

CDCl3, 126 MHz, mixture of rotamers
Me
Me
Me
Me
O
O
B
N
O
Me
Me
Me
33
175.80
143.71
128.13
126.80
126.67
83.61
83.43
65.00
49.13
39.35
27.55
25.14
25.03
24.68
3500000
3000000
2500000
2000000
1500000
1000000
500000
0
210
200
190
180
170
160
150
140
130
120
110
100
90
80
70
60
50
40
30
20
10
0
-10
f1 (ppm)

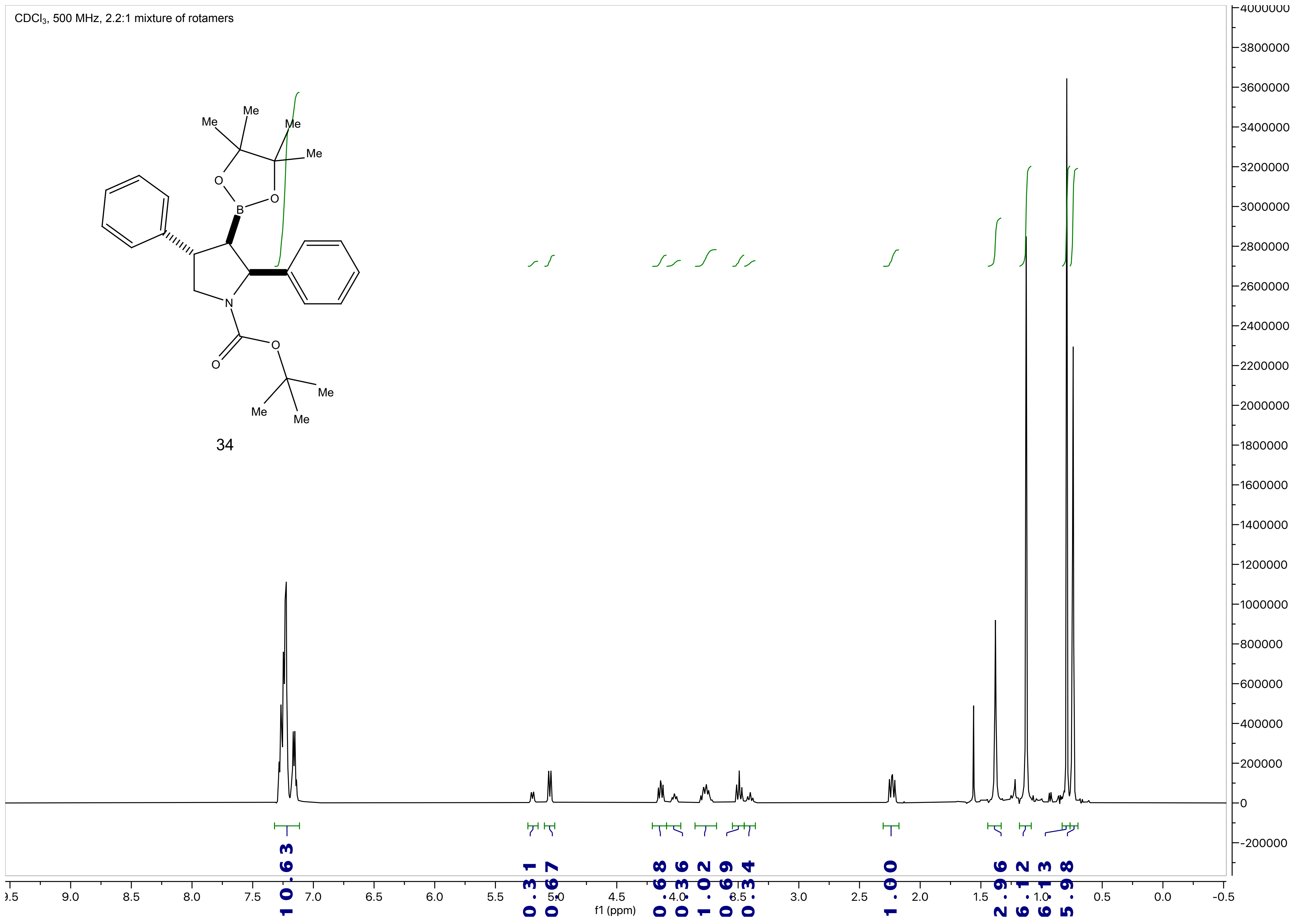

CDCl3, 500 MHz, 2.2:1 mixture of rotamers
34
f1 (ppm)
10.63
0.31
0.67
0.68
0.36
1.02
0.69
0.34
1.00
2.96
6.12
6.13
5.98

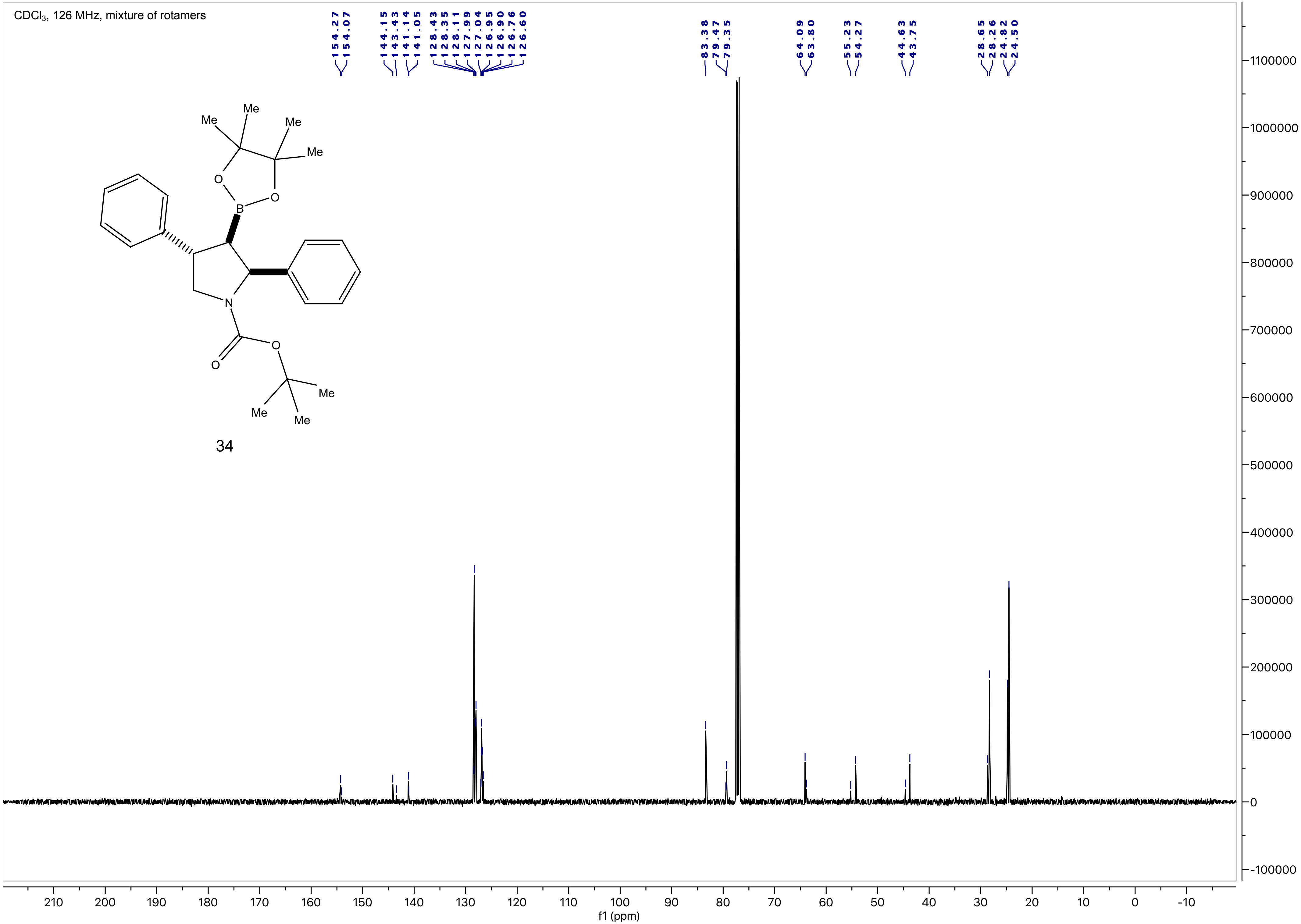

CDCl$_3$, 126 MHz, mixture of rotamers
154.27
154.07
144.15
143.43
141.14
141.05
128.43
128.35
128.11
127.99
127.04
126.95
126.90
126.76
126.60
83.38
79.47
79.35
64.09
63.80
55.23
54.27
44.63
43.75
28.65
28.26
24.82
24.50
Me
Me
Me
Me
O
B
O
N
O
O
Me
Me
Me
34
f1 (ppm)

CDCl$_3$, 500 MHz, 1.5:1 mixture of rotamers

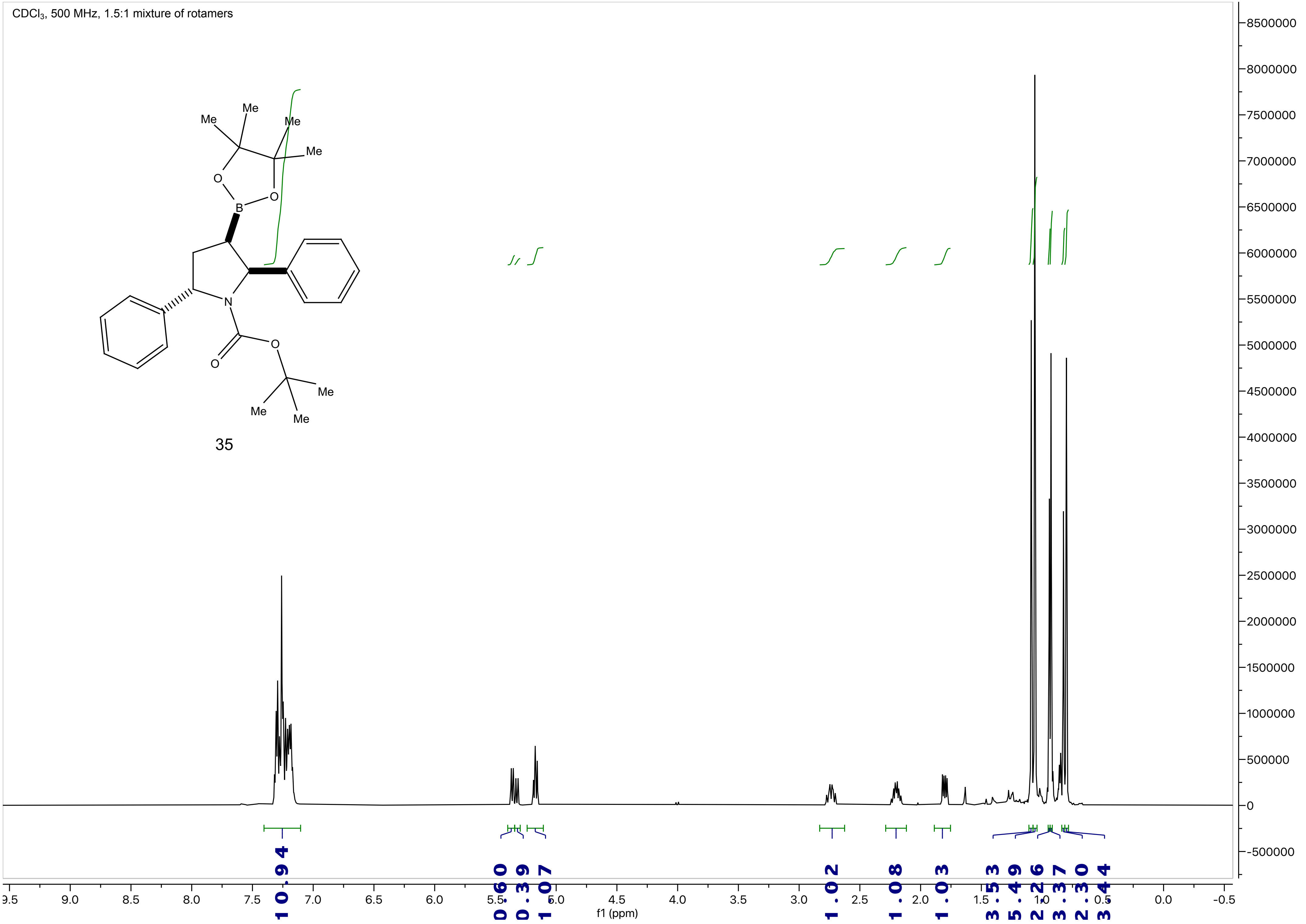

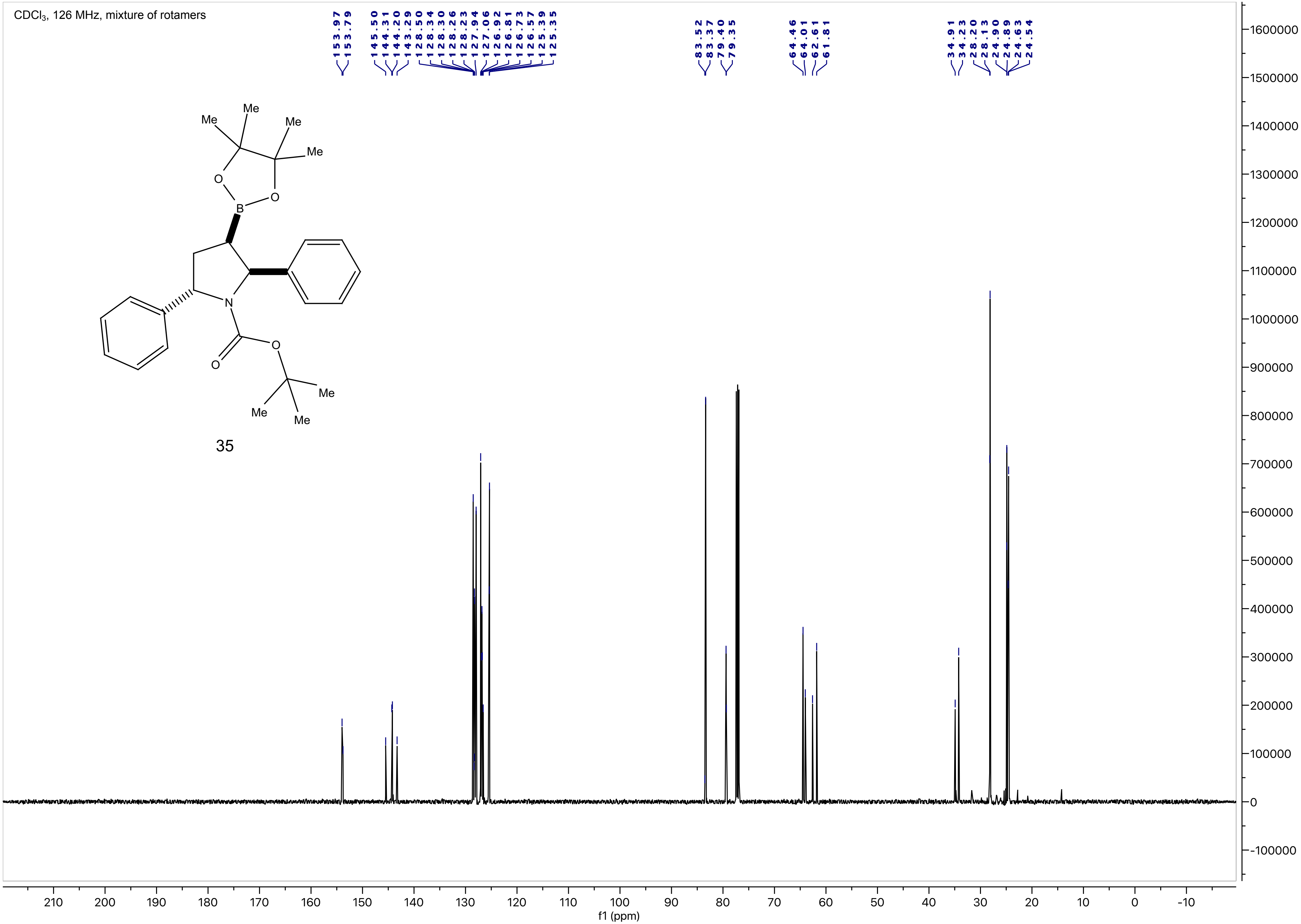
CDCl$_3$, 126 MHz, mixture of rotamers
153.97
153.79
145.50
144.31
144.20
143.29
128.50
128.34
128.30
128.26
128.23
127.94
127.06
126.92
126.81
126.73
126.57
125.39
125.35
83.52
83.37
79.40
79.35
64.46
64.01
62.61
61.81
34.91
34.23
28.20
28.13
24.90
24.89
24.63
24.54
Me
O
B
N
O
35
f1 (ppm)

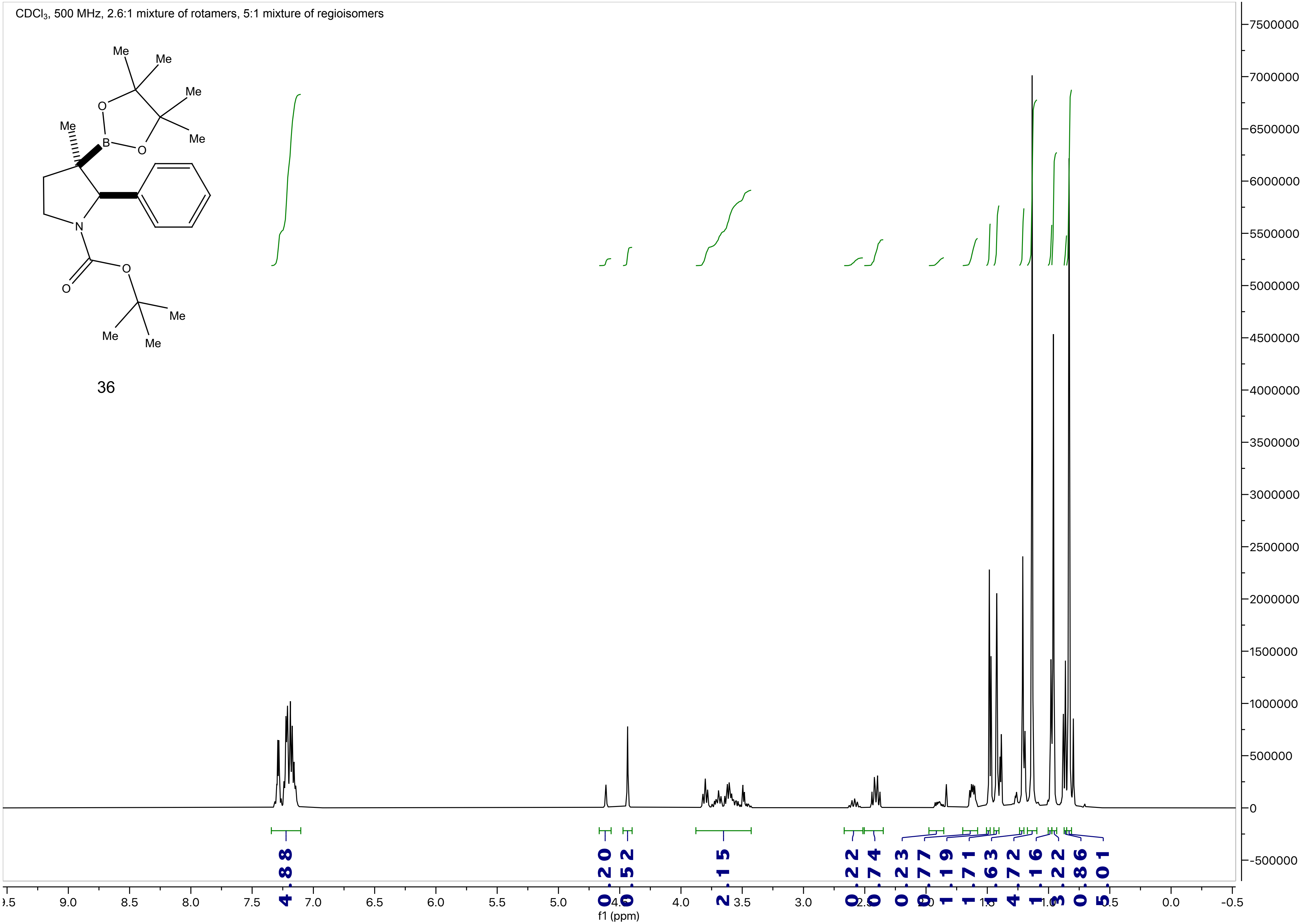

CDCl$_3$, 500 MHz, 2.6:1 mixture of rotamers, 5:1 mixture of regioisomers
Me
Me
Me
Me
Me
O
B
O
N
O
O
Me
Me
Me
36
4.88
0.20
0.52
2.15
0.22
0.74
0.23
0.77
1.19
1.71
1.63
4.72
1.16
3.22
0.86
5.01
f1 (ppm)

CDCl$_3$, 126 MHz, mixture of rotamers and regioisomers

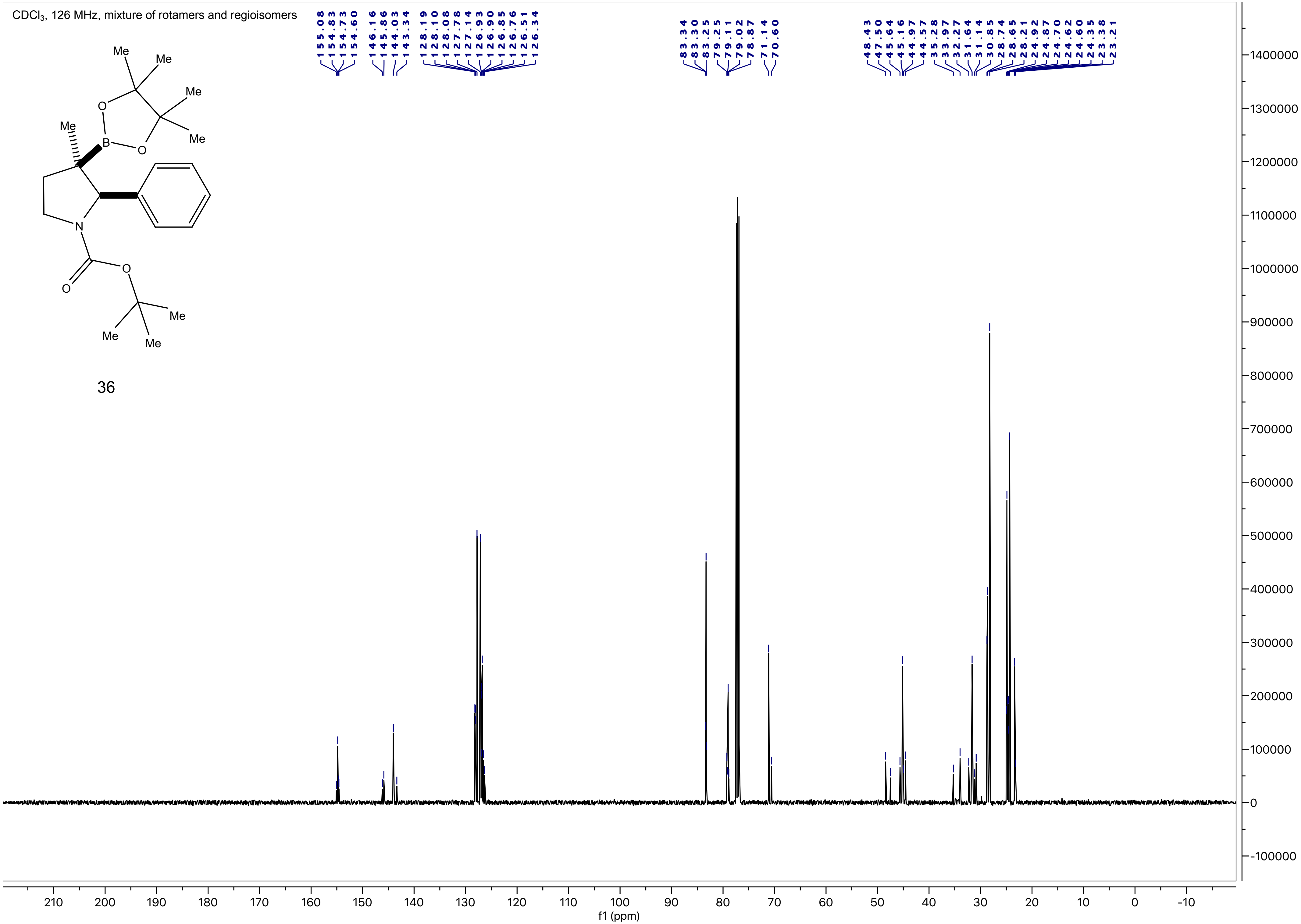

36

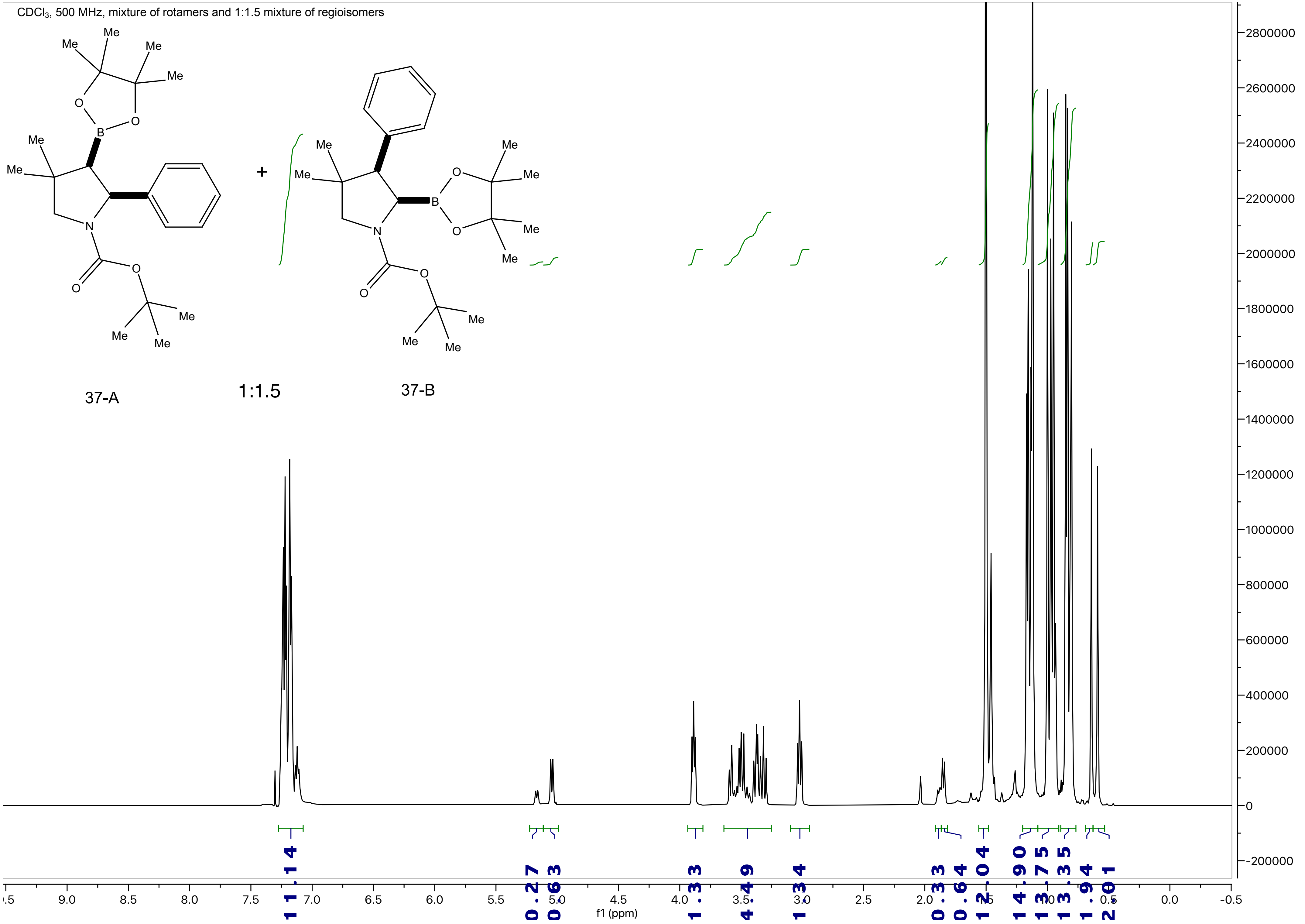
CDCl3, 500 MHz, mixture of rotamers and 1:1.5 mixture of regioisomers
37-A
1:1.5
37-B
f1 (ppm)
11.14
0.27
0.63
1.33
4.49
1.34
0.33
0.64
12.04
14.90
13.75
13.35
1.94
2.01

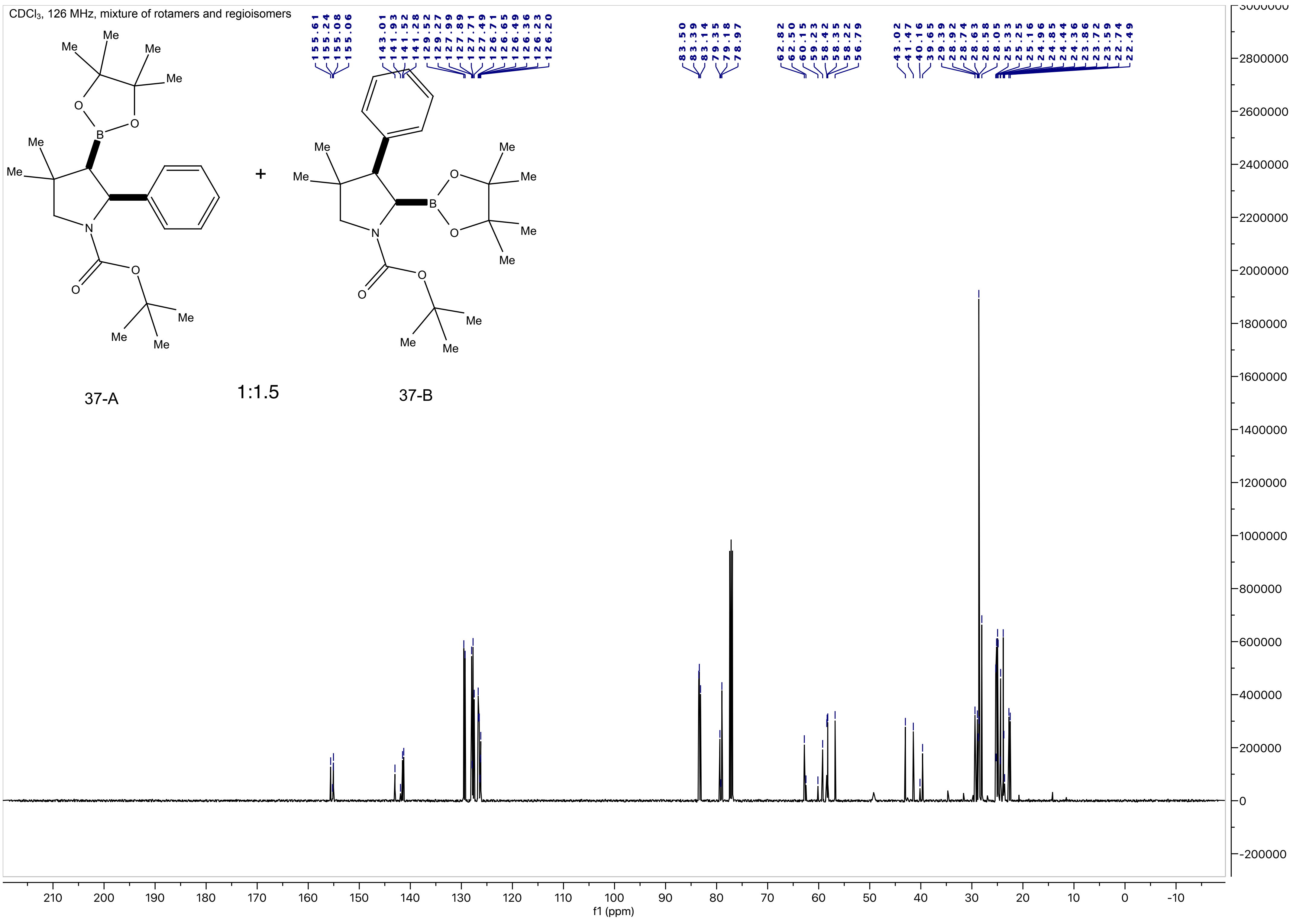

CDCl3, 126 MHz, mixture of rotamers and regioisomers
155.61
155.24
155.08
155.06
143.01
141.93
141.52
141.28
129.52
129.27
127.99
127.89
127.71
127.49
126.71
126.65
126.49
126.36
126.23
126.20
83.50
83.39
83.14
79.35
79.18
78.97
62.82
62.50
60.15
59.23
58.42
58.35
58.22
56.79
43.02
41.47
40.16
39.65
29.39
28.92
28.74
28.63
28.58
28.05
25.33
25.25
25.16
24.96
24.85
24.44
24.36
23.86
23.72
23.59
22.74
22.49
37-A
1:1.5
37-B
f1 (ppm)

CDCl$_3$, 500 MHz, 2.2:1 mixture of rotamers

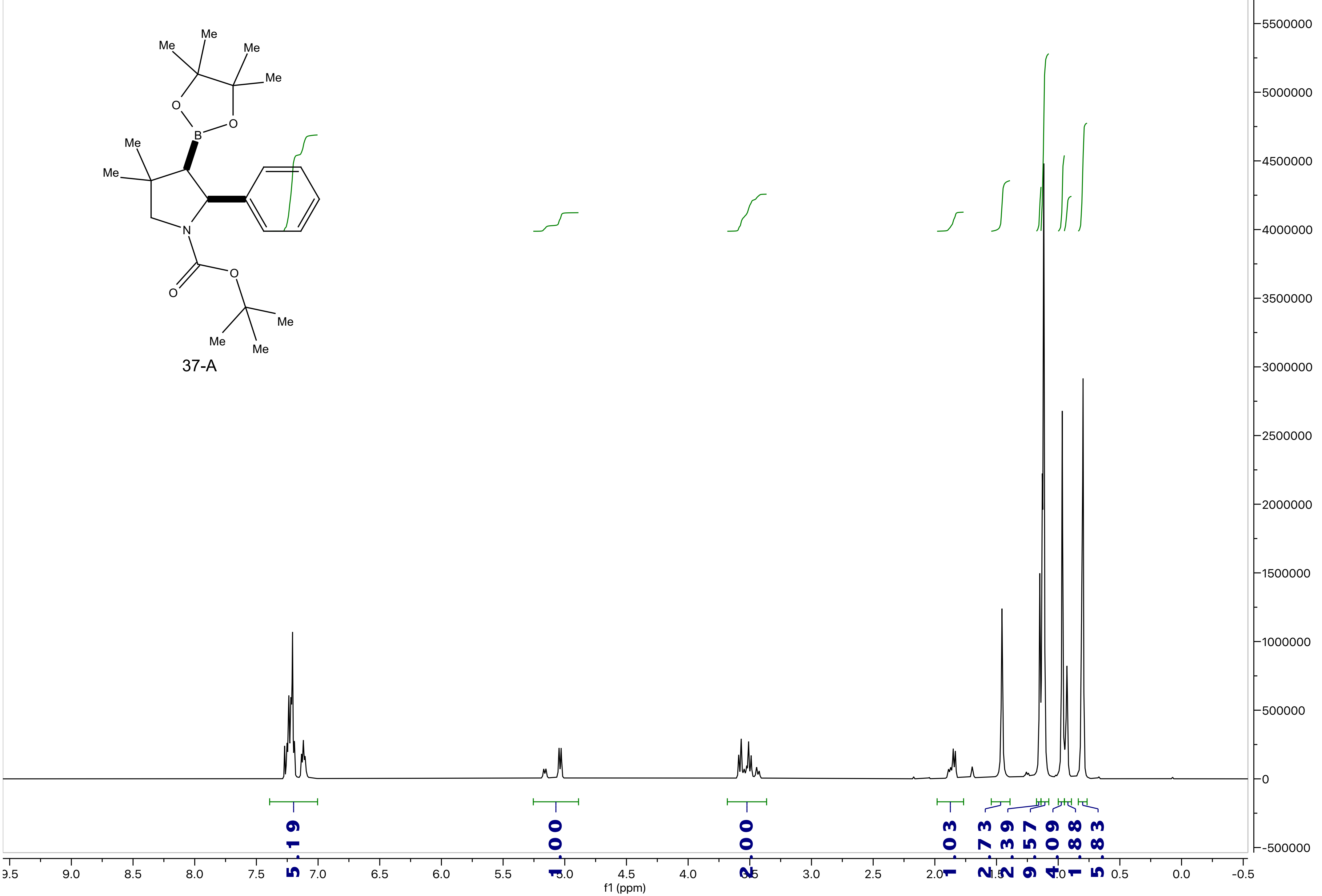

CDCl$_3$, 126 MHz, mixture of rotamers

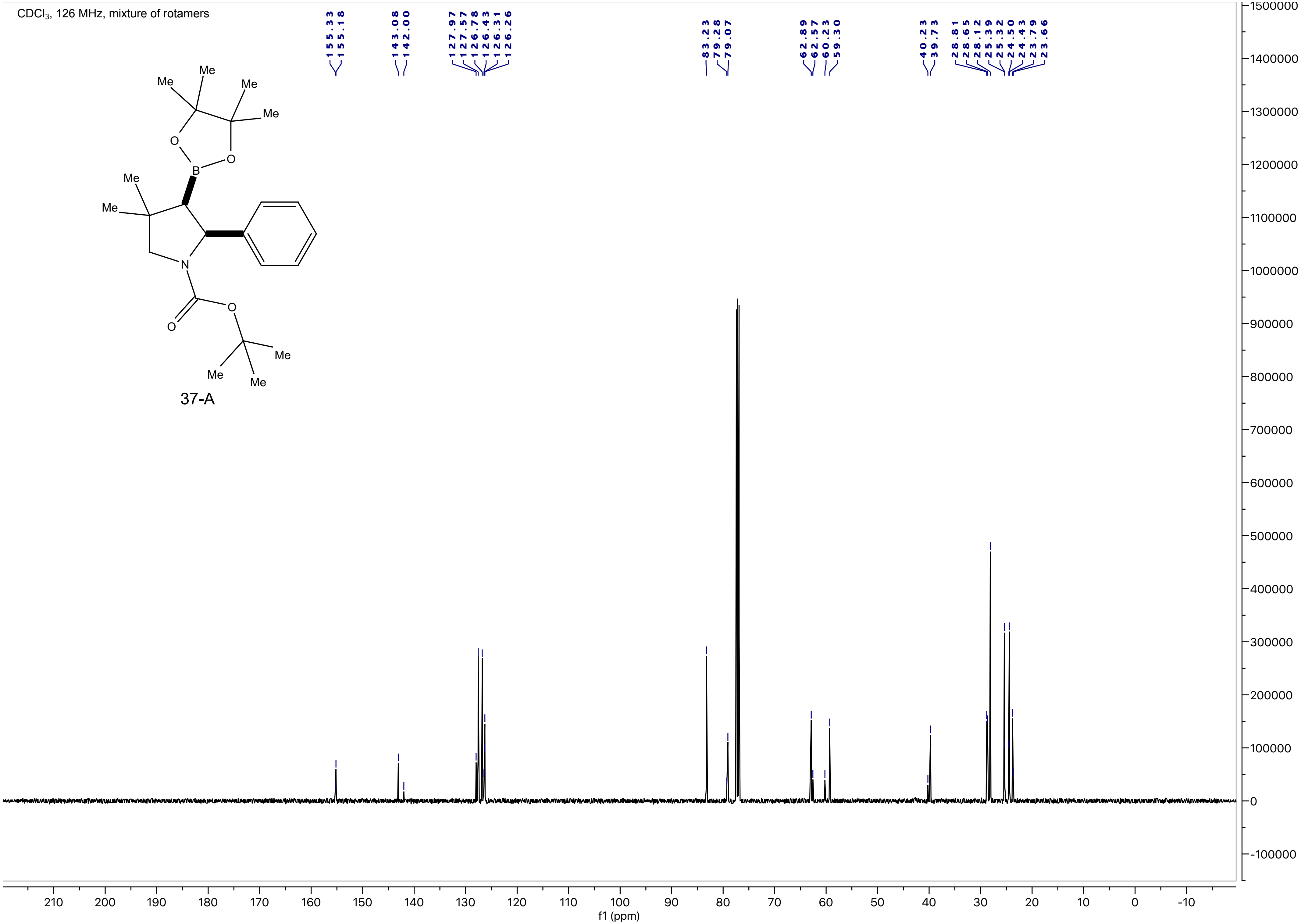

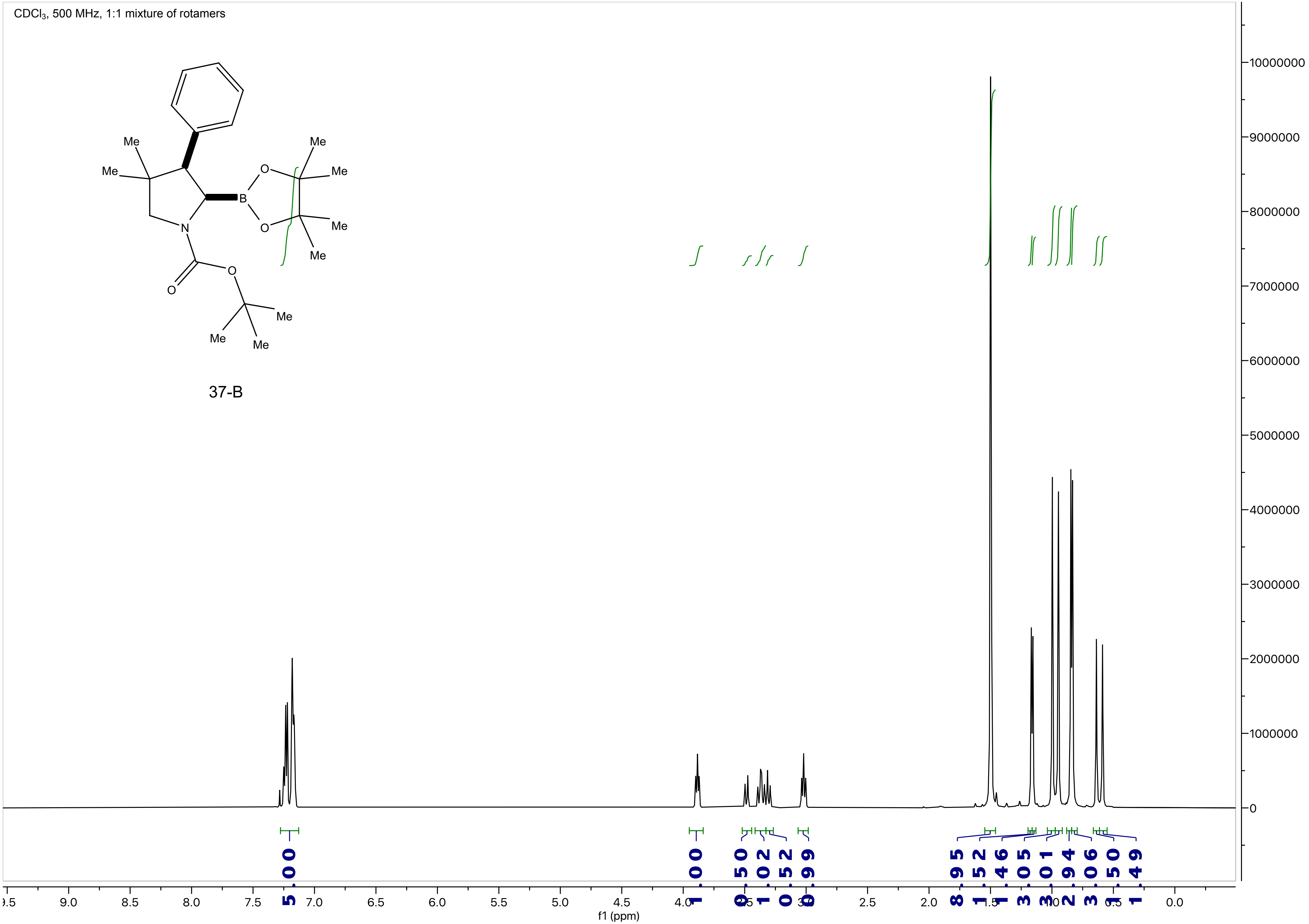
CDCl$_3$, 500 MHz, 1:1 mixture of rotamers
37-B
5.00
1.00
0.50
1.02
0.52
0.99
8.95
1.52
1.46
3.05
3.01
2.94
3.06
0.50
1.49
f1 (ppm)

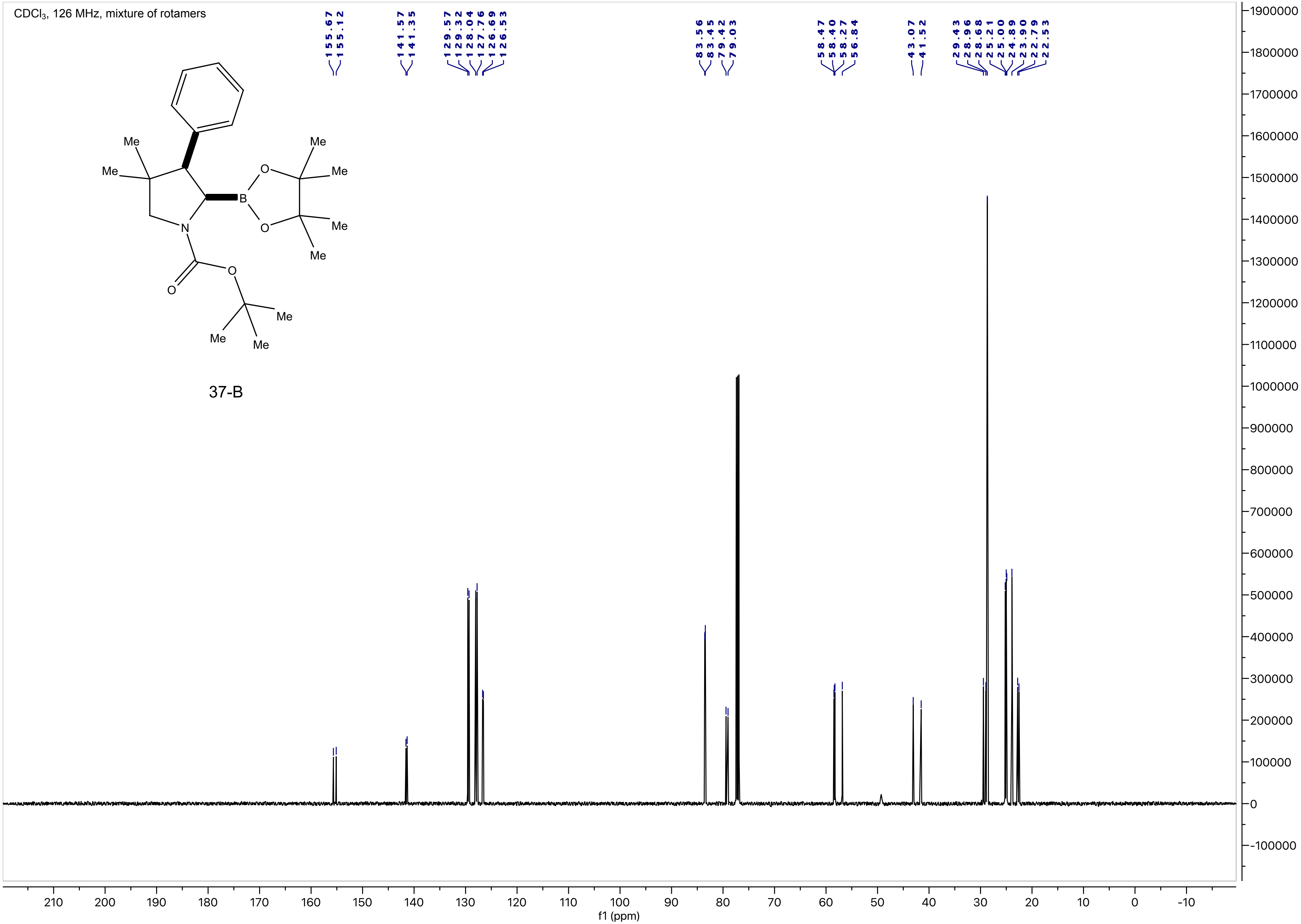
CDCl3, 126 MHz, mixture of rotamers
155.67
155.12
141.57
141.35
129.57
129.32
128.04
127.76
126.69
126.53
83.56
83.45
79.42
79.03
58.47
58.40
58.27
56.84
43.07
41.52
29.43
28.96
28.68
25.21
25.00
24.89
23.90
22.79
22.53
Me
Me
Me
Me
Me
Me
O
O
B
N
O
O
Me
Me
Me
37-B
f1 (ppm)

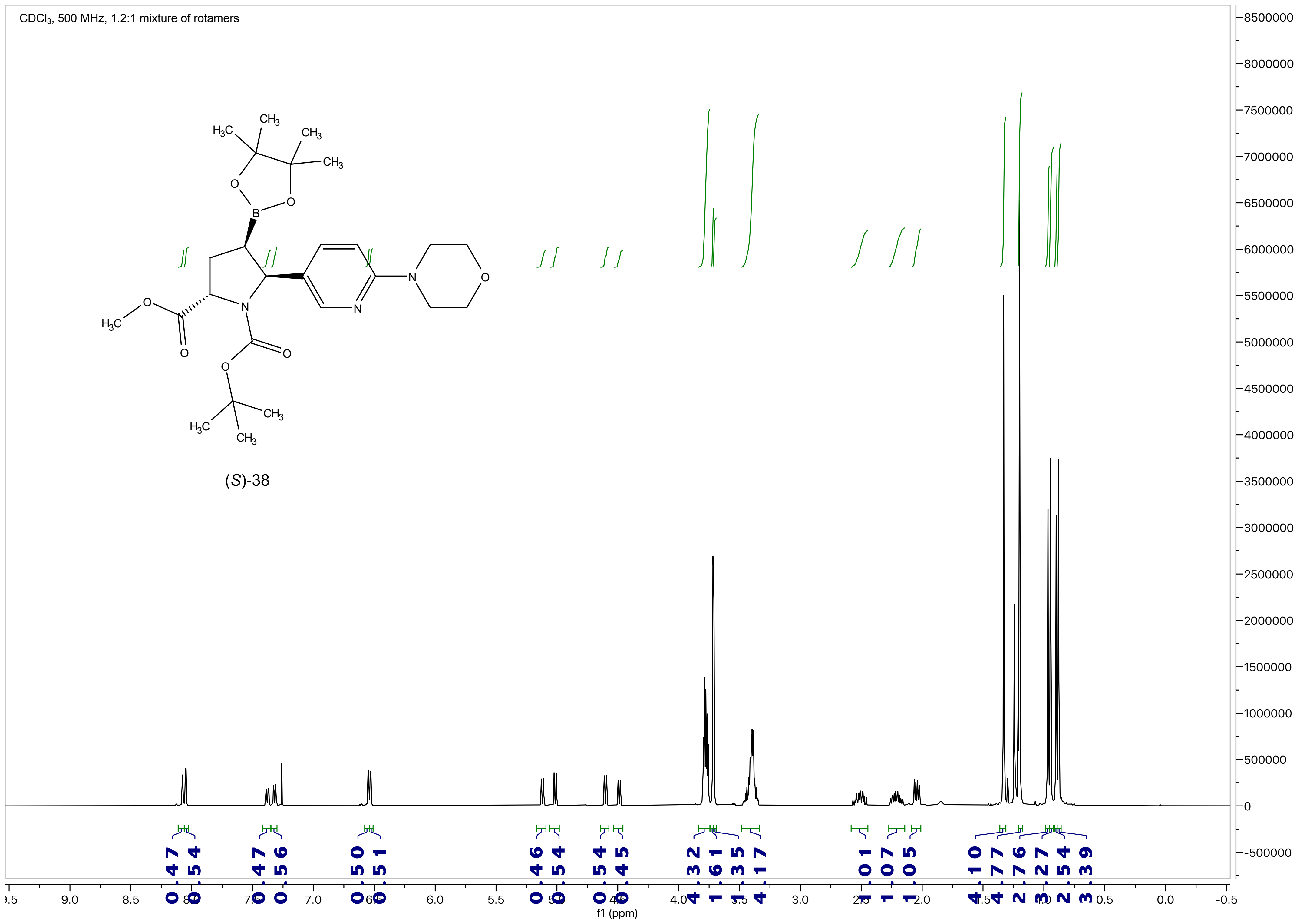
CDCl3, 500 MHz, 1.2:1 mixture of rotamers
(S)-38
f1 (ppm)

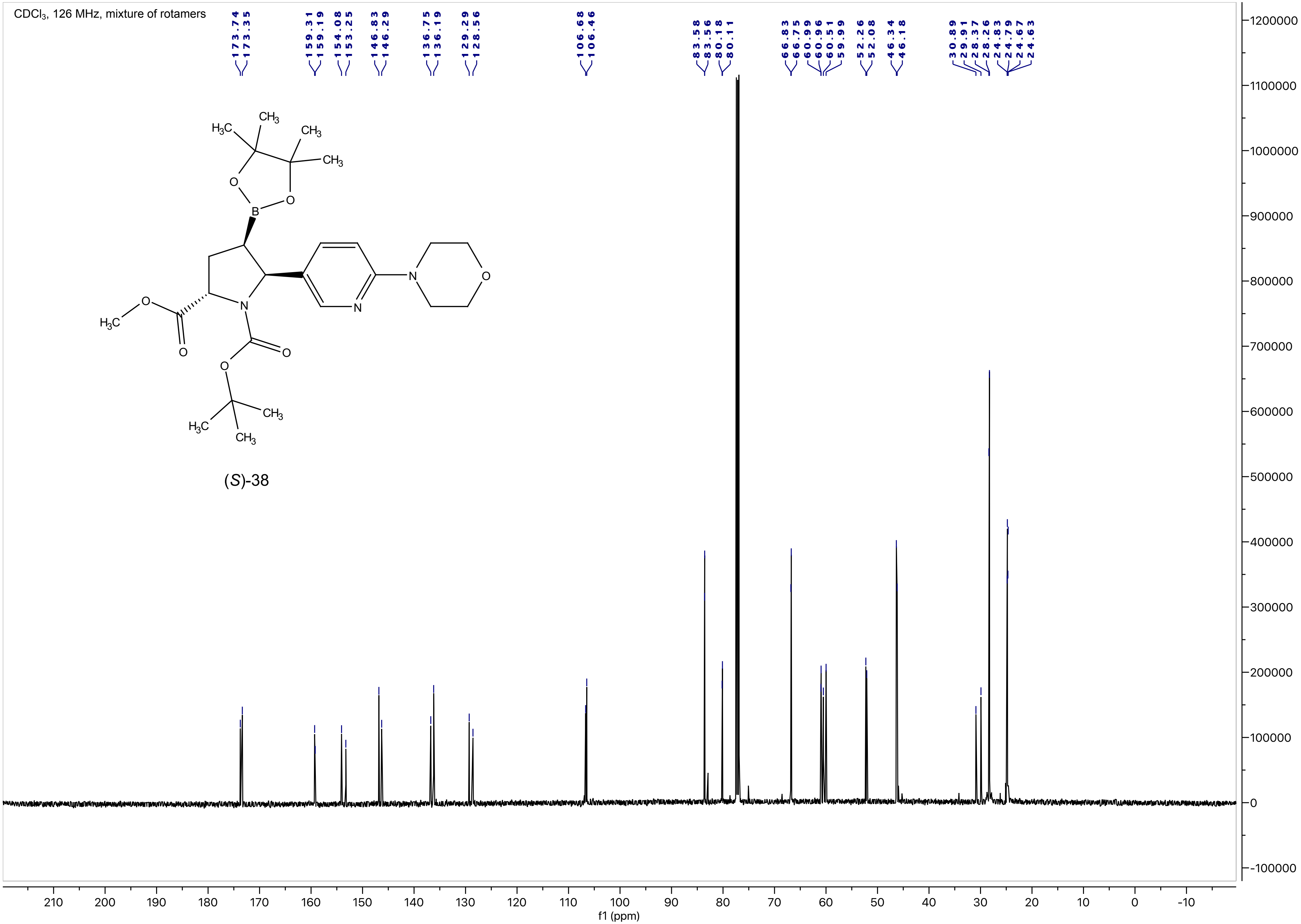
CDCl$_3$, 126 MHz, mixture of rotamers
173.74
173.35
159.31
159.19
154.08
153.25
146.83
146.29
136.75
136.19
129.29
128.56
106.68
106.46
83.58
83.56
80.18
80.11
66.83
66.75
60.99
60.96
60.51
59.99
52.26
52.08
46.34
46.18
30.89
29.91
28.37
28.26
24.83
24.79
24.67
24.63
(S)-38
f1 (ppm)

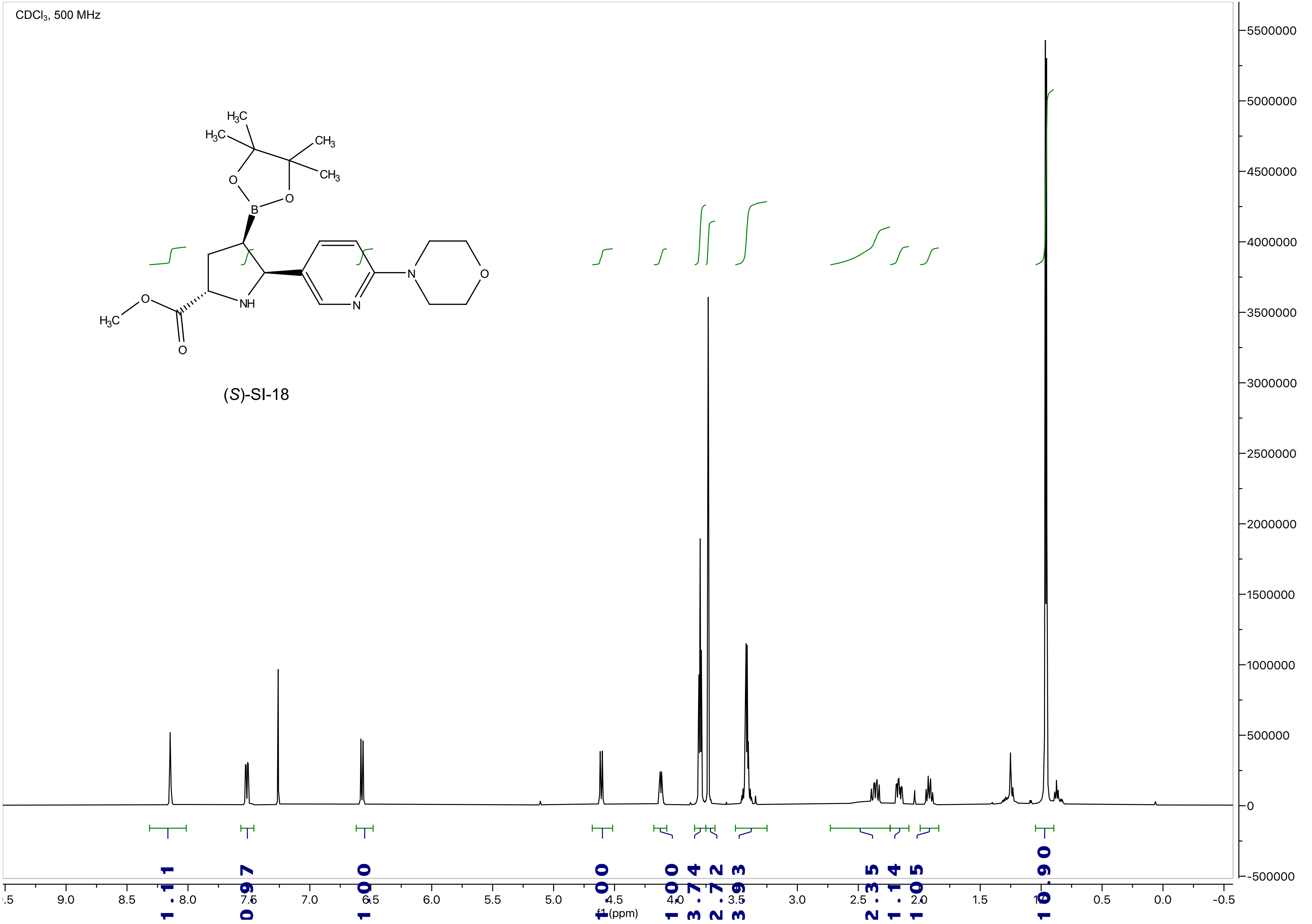
CDCl3, 500 MHz
(S)-SI-18
f1 (ppm)
1.11
0.97
1.00
1.00
1.00
3.74
2.72
3.93
2.35
1.14
1.05
10.90

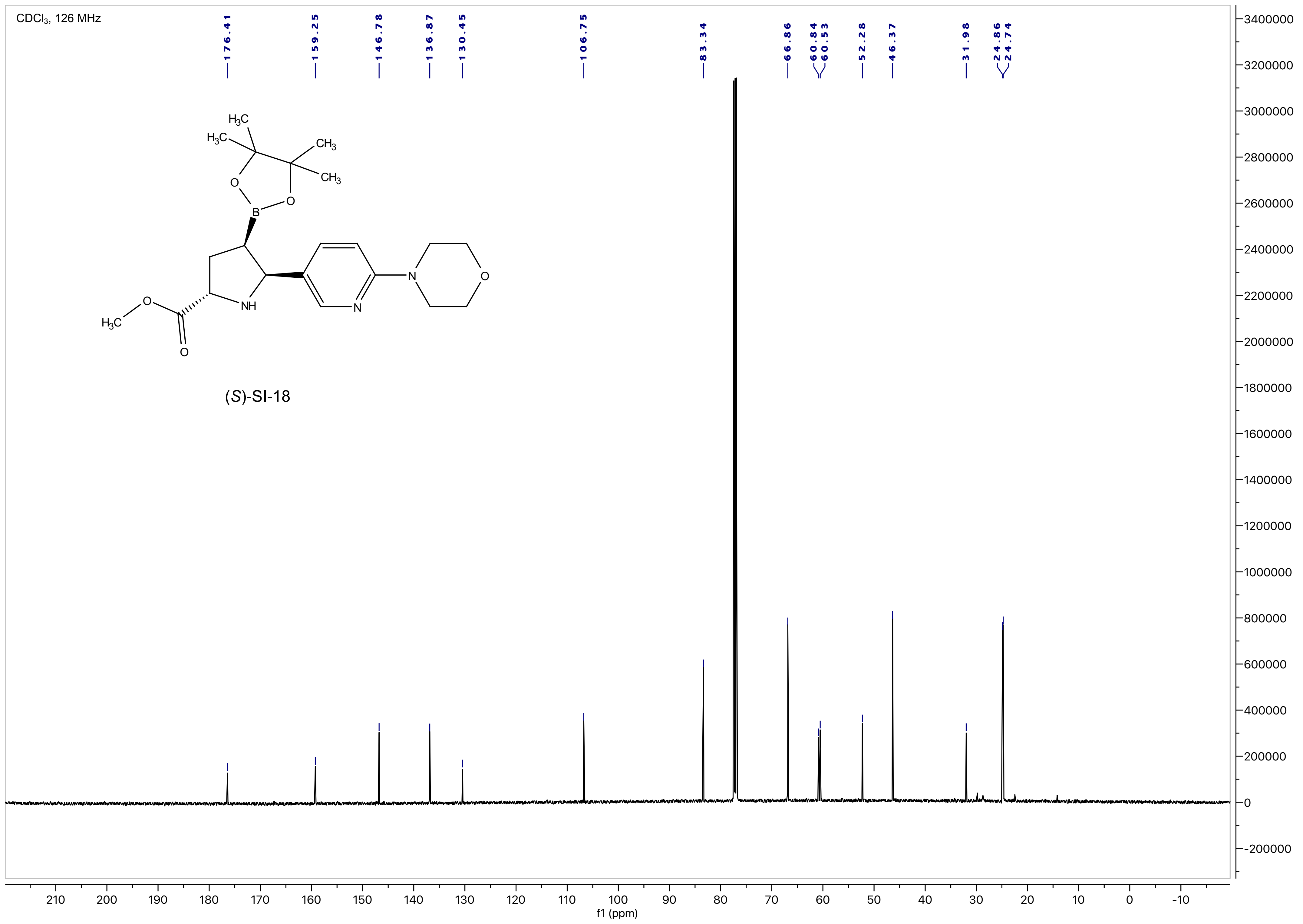

CDCl3, 126 MHz
176.41
159.25
146.78
136.87
130.45
106.75
83.34
66.86
60.84
60.53
52.28
46.37
31.98
24.86
24.74
(S)-SI-18
f1 (ppm)

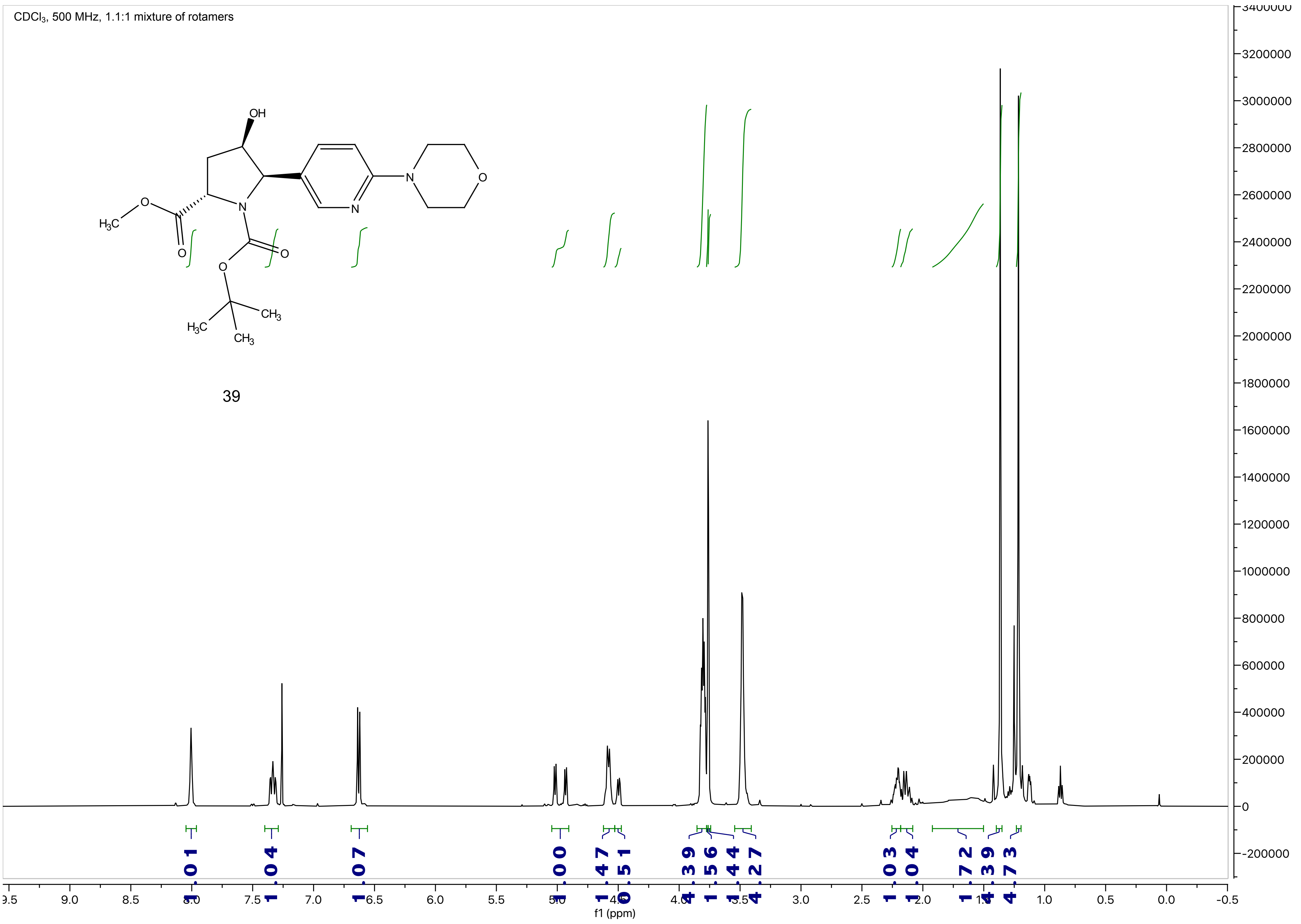

CDCl3, 500 MHz, 1.1:1 mixture of rotamers
39
f1 (ppm)

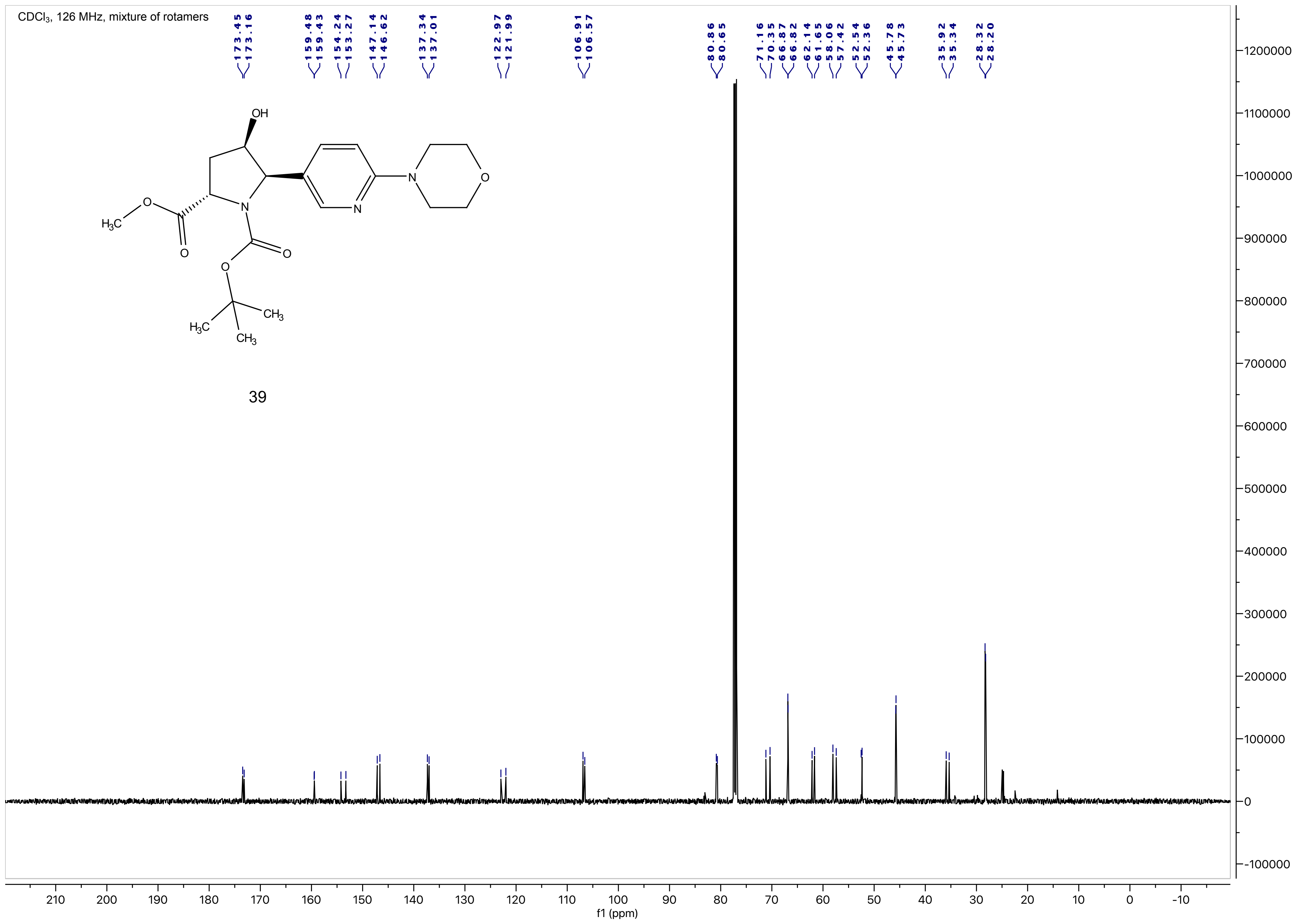
CDCl3, 126 MHz, mixture of rotamers
173.45
173.16
159.48
159.43
154.24
153.27
147.14
146.62
137.34
137.01
122.97
121.99
106.91
106.57
80.86
80.65
71.16
70.35
66.87
66.82
62.14
61.65
58.06
57.42
52.54
52.36
45.78
45.73
35.92
35.34
28.32
28.20
OH
N
O
H3C
O
O
N
N
O
O
CH3
H3C
CH3
39
f1 (ppm)

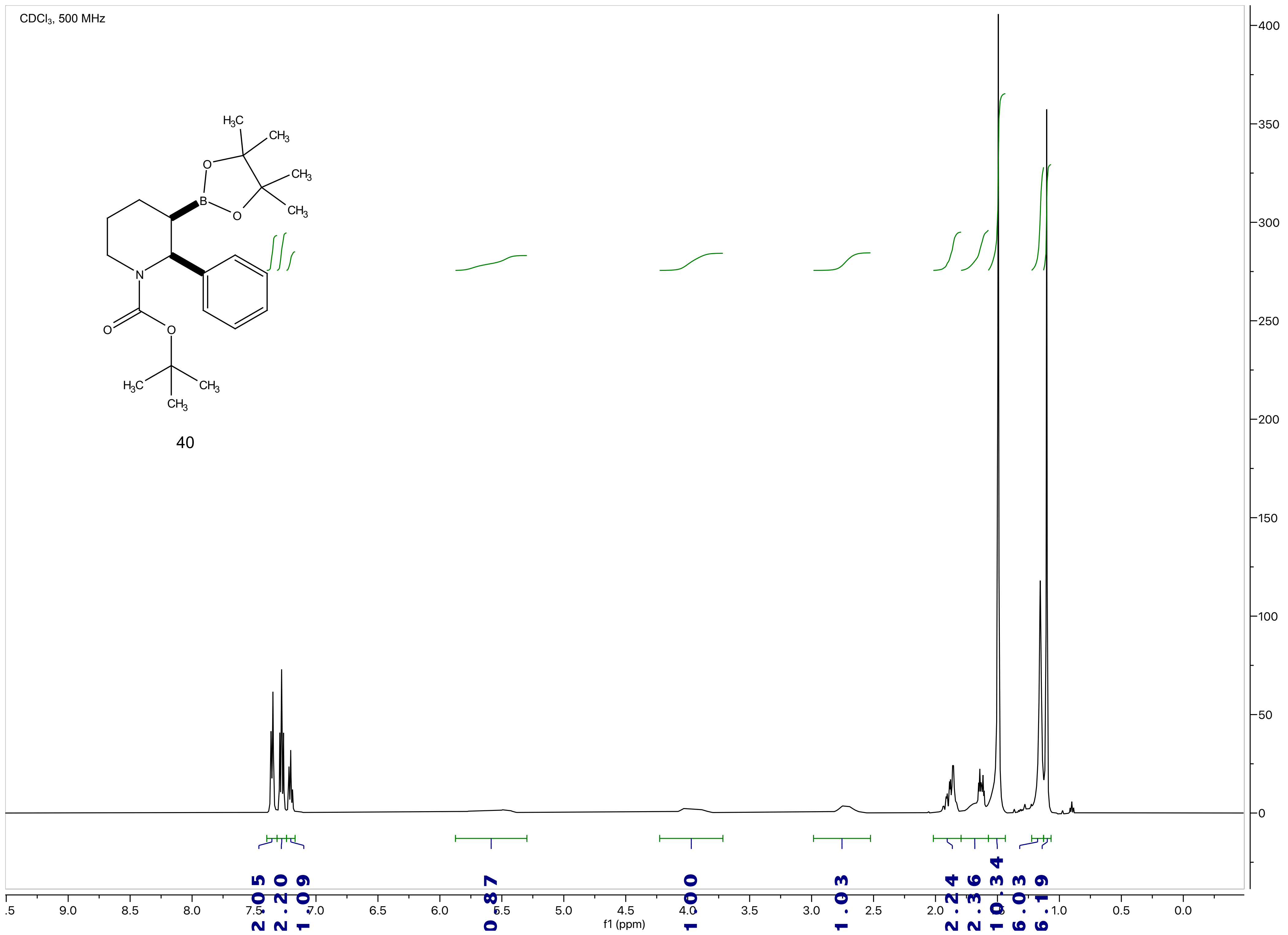
CDCl3, 500 MHz
40
f1 (ppm)
2.05
2.20
1.09
0.87
1.00
1.03
2.24
2.36
10.34
6.03
6.19

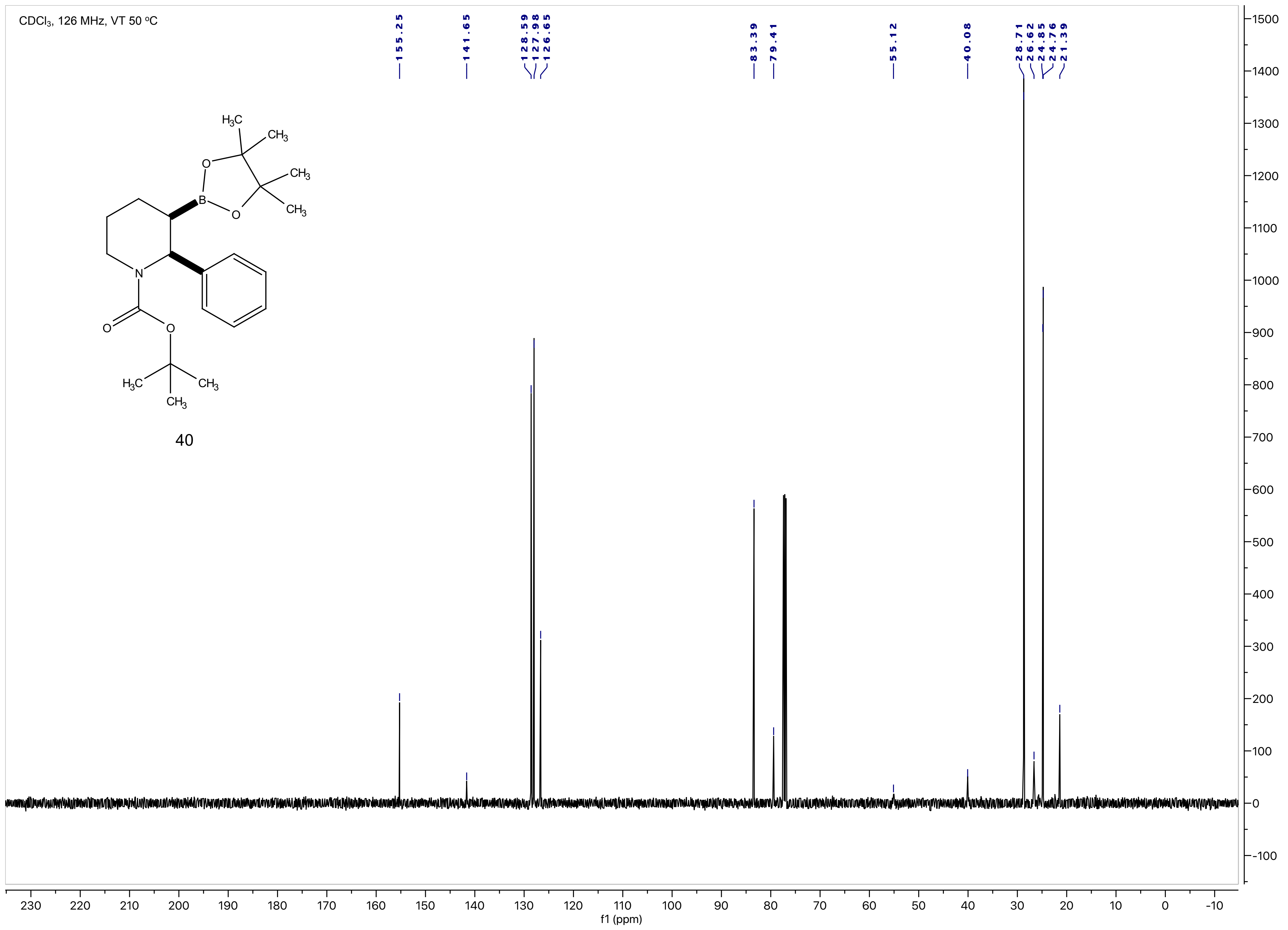

CDCl3, 126 MHz, VT 50 ºC
155.25
141.65
128.59
127.98
126.65
83.39
79.41
55.12
40.08
28.71
26.62
24.85
24.76
21.39
40
f1 (ppm)

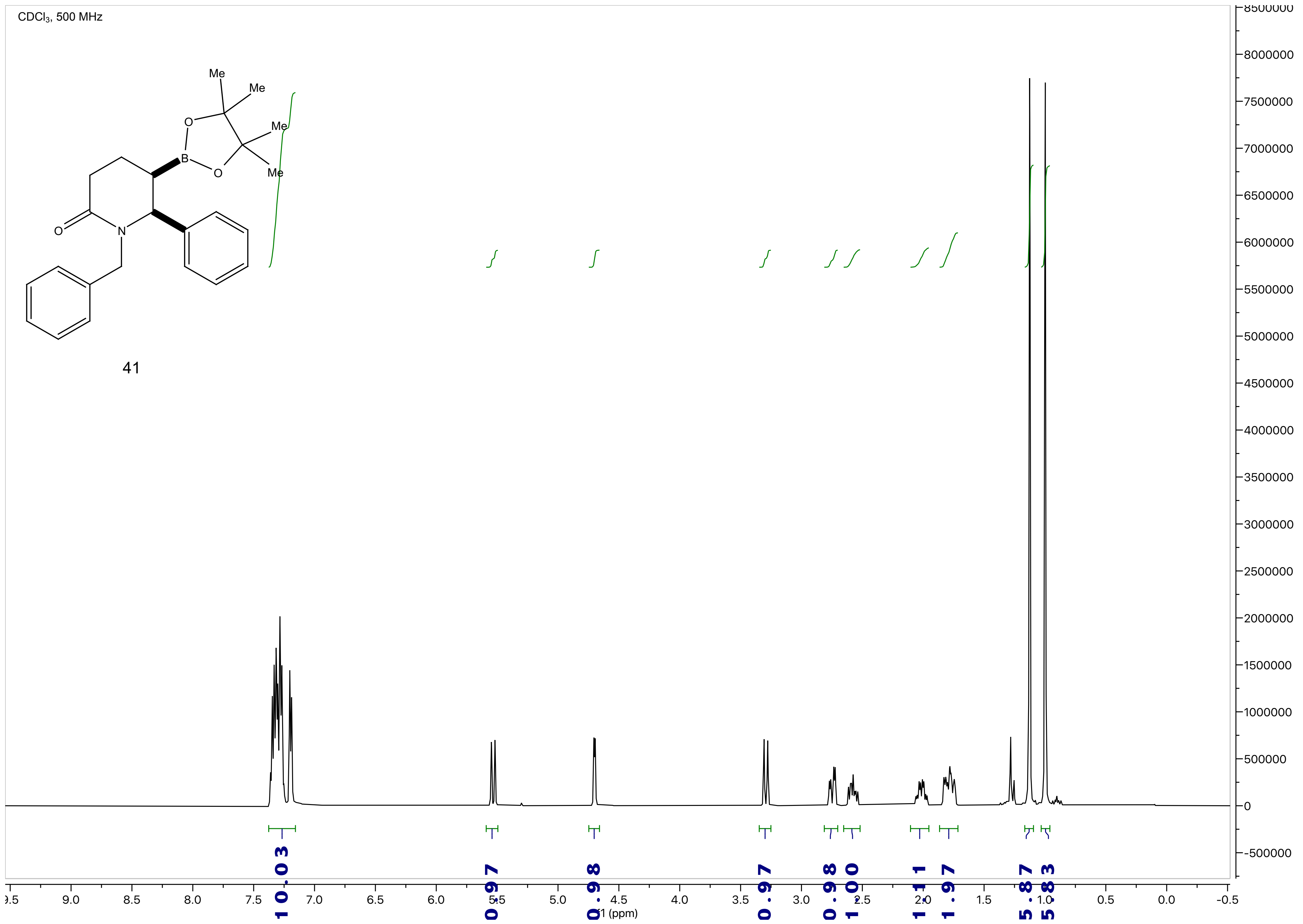
CDCl3, 500 MHz
Me
Me
Me
Me
O
O
B
O
N
41
10.03
0.97
0.98
0.97
0.98
1.00
1.11
1.97
5.87
5.83
f1 (ppm)

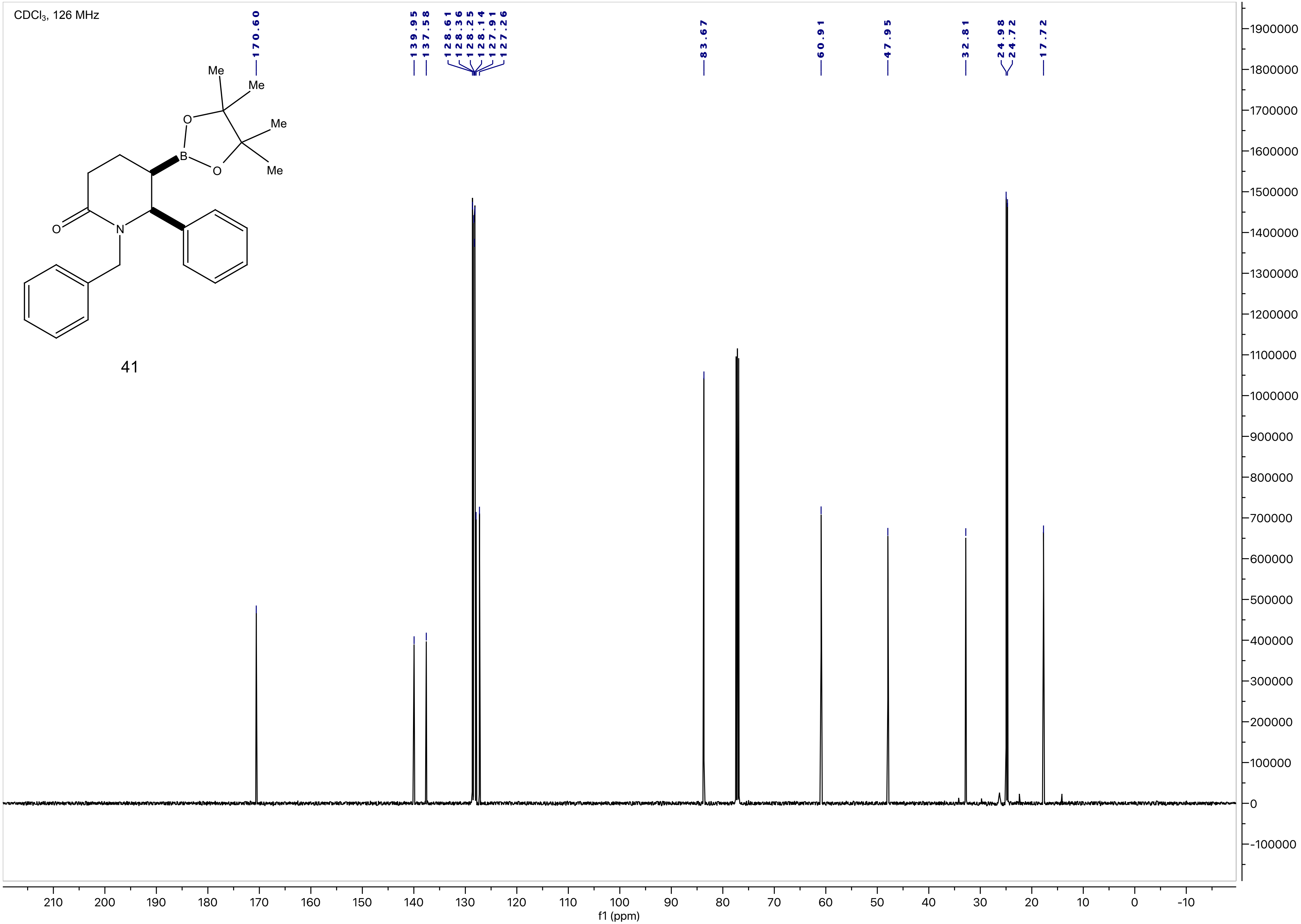
CDCl3, 126 MHz
170.60
139.95
137.58
128.61
128.36
128.25
128.14
127.91
127.26
83.67
60.91
47.95
32.81
24.98
24.72
17.72
Me
Me
Me
Me
O
B
O
O
N
41
f1 (ppm)

CDCl$_3$, 400 MHz

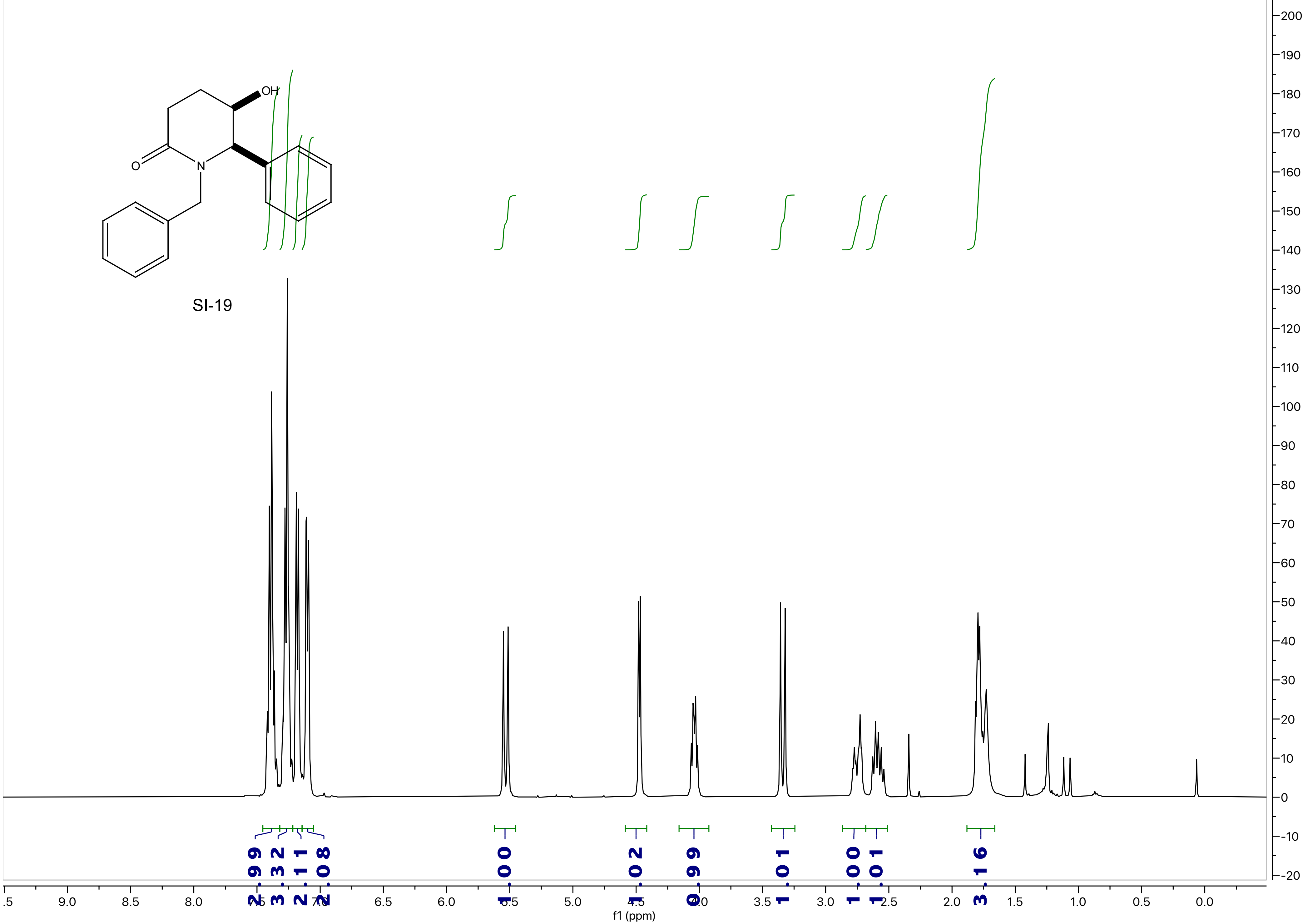

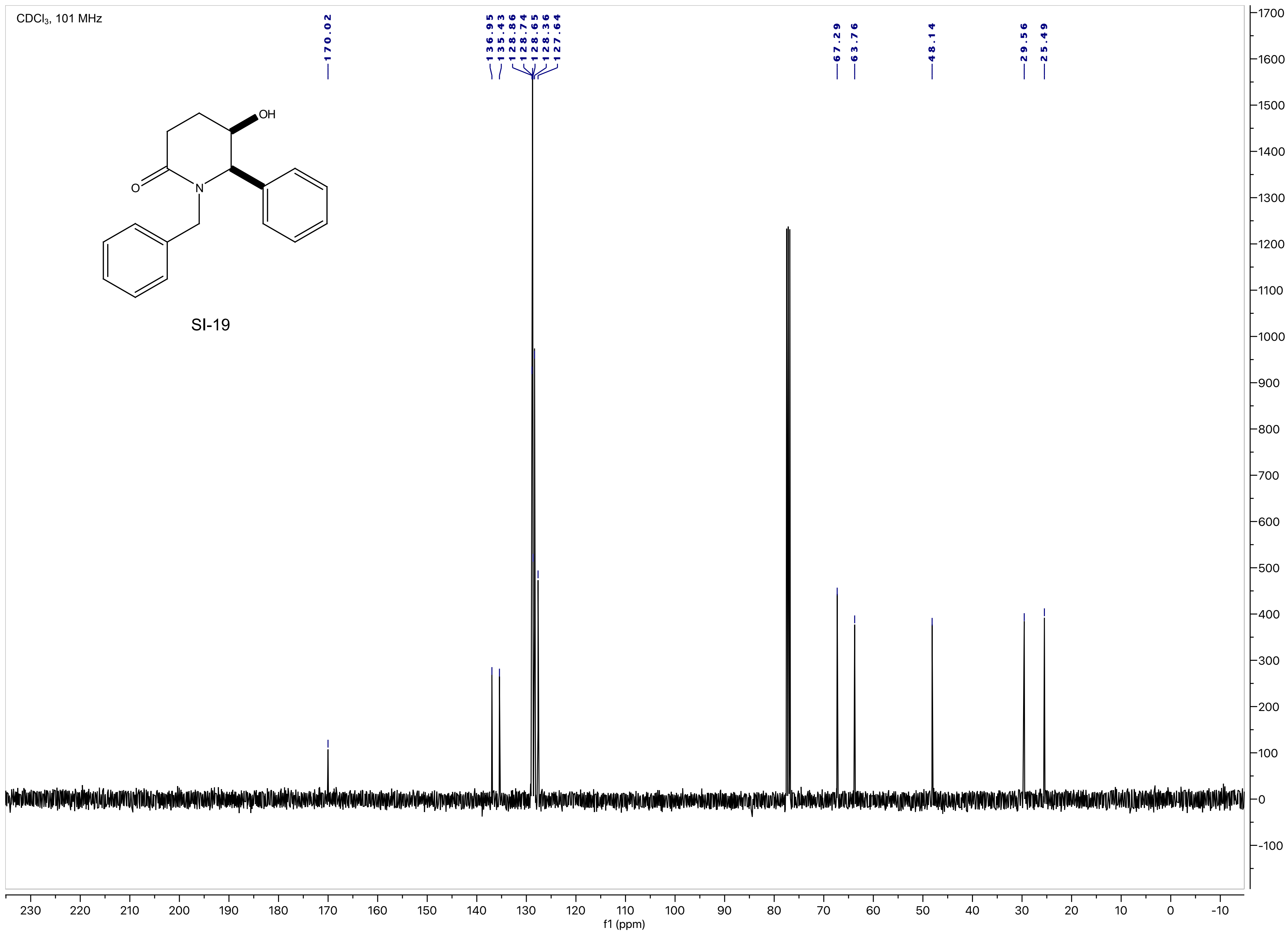

CDCl3, 101 MHz
170.02
136.95
135.43
128.86
128.74
128.65
128.36
127.64
67.29
63.76
48.14
29.56
25.49
OH
O
N
SI-19
f1 (ppm)

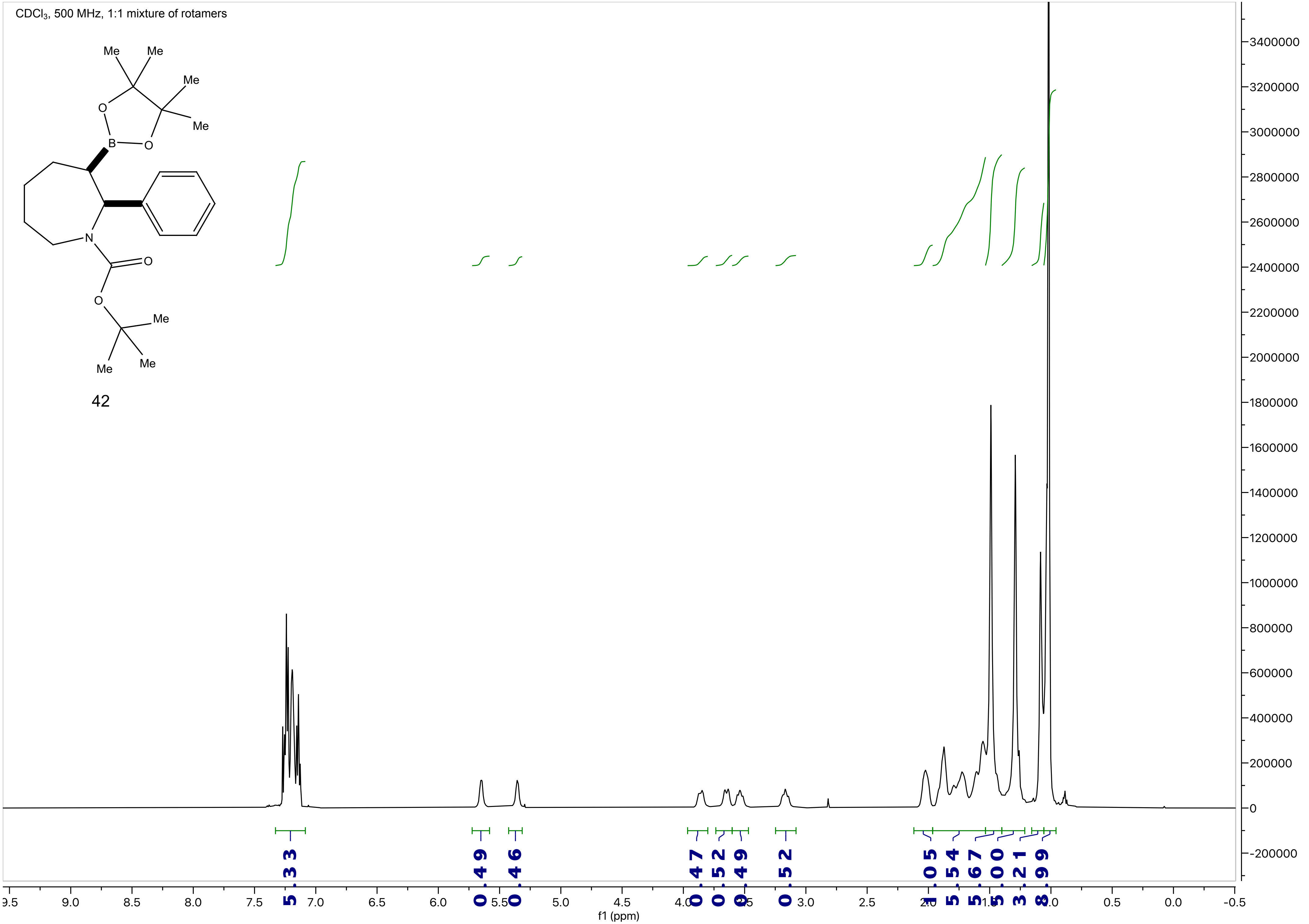

CDCl3, 500 MHz, 1:1 mixture of rotamers
42
5.33
0.49
0.46
0.47
0.52
0.49
0.52
1.05
5.54
5.67
5.00
3.21
8.99
f1 (ppm)

CDCl$_3$, 126 MHz, mixture of rotamers

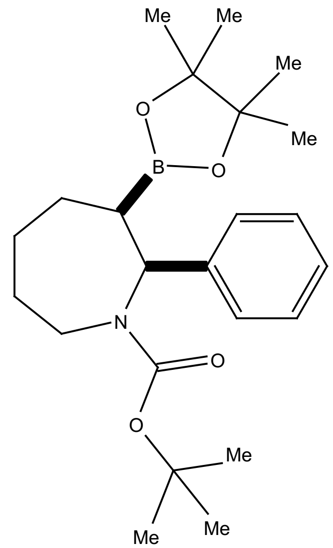

42

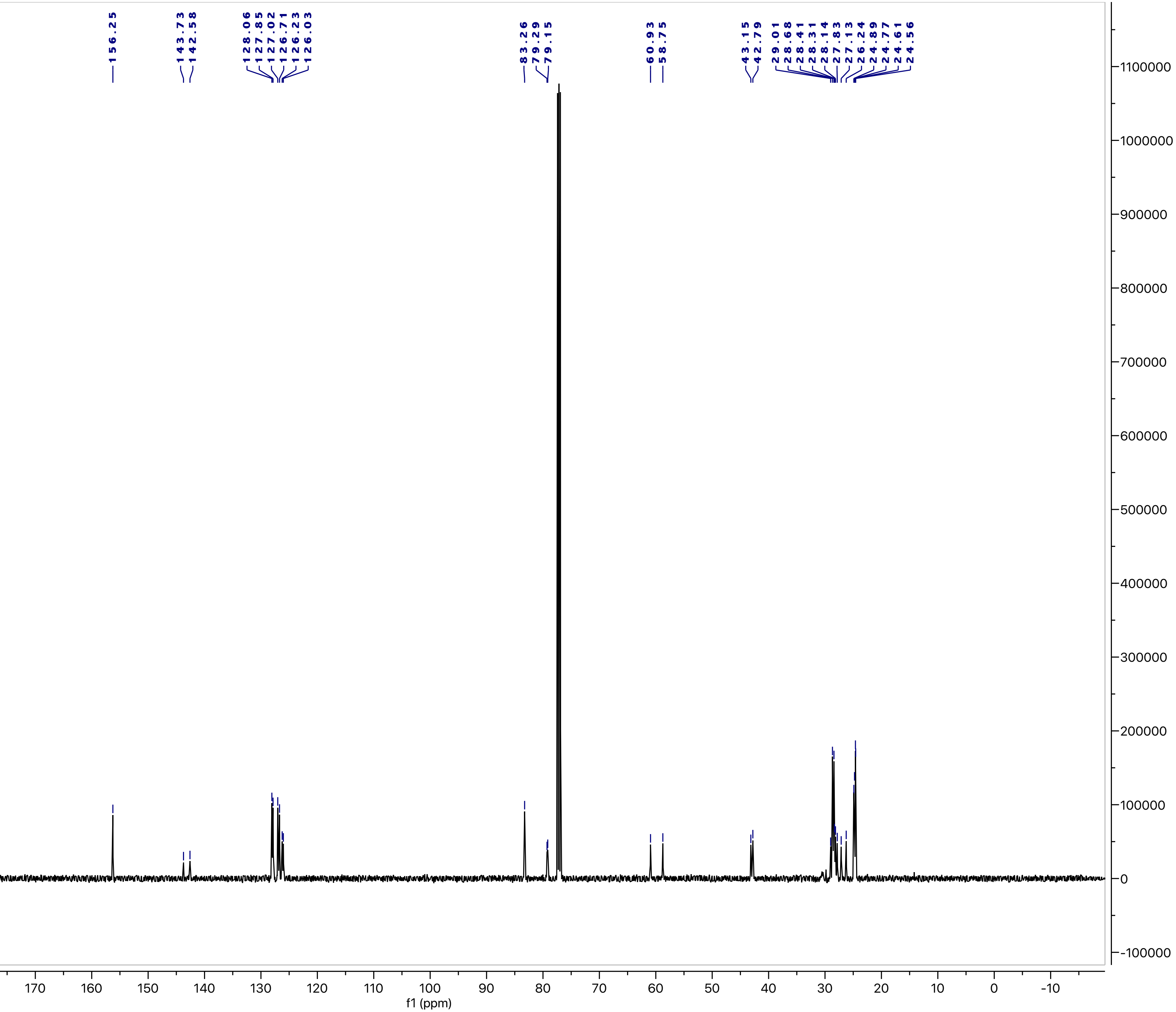

CDCl$_3$, 500 MHz, 1:1 mixture of regioisomers

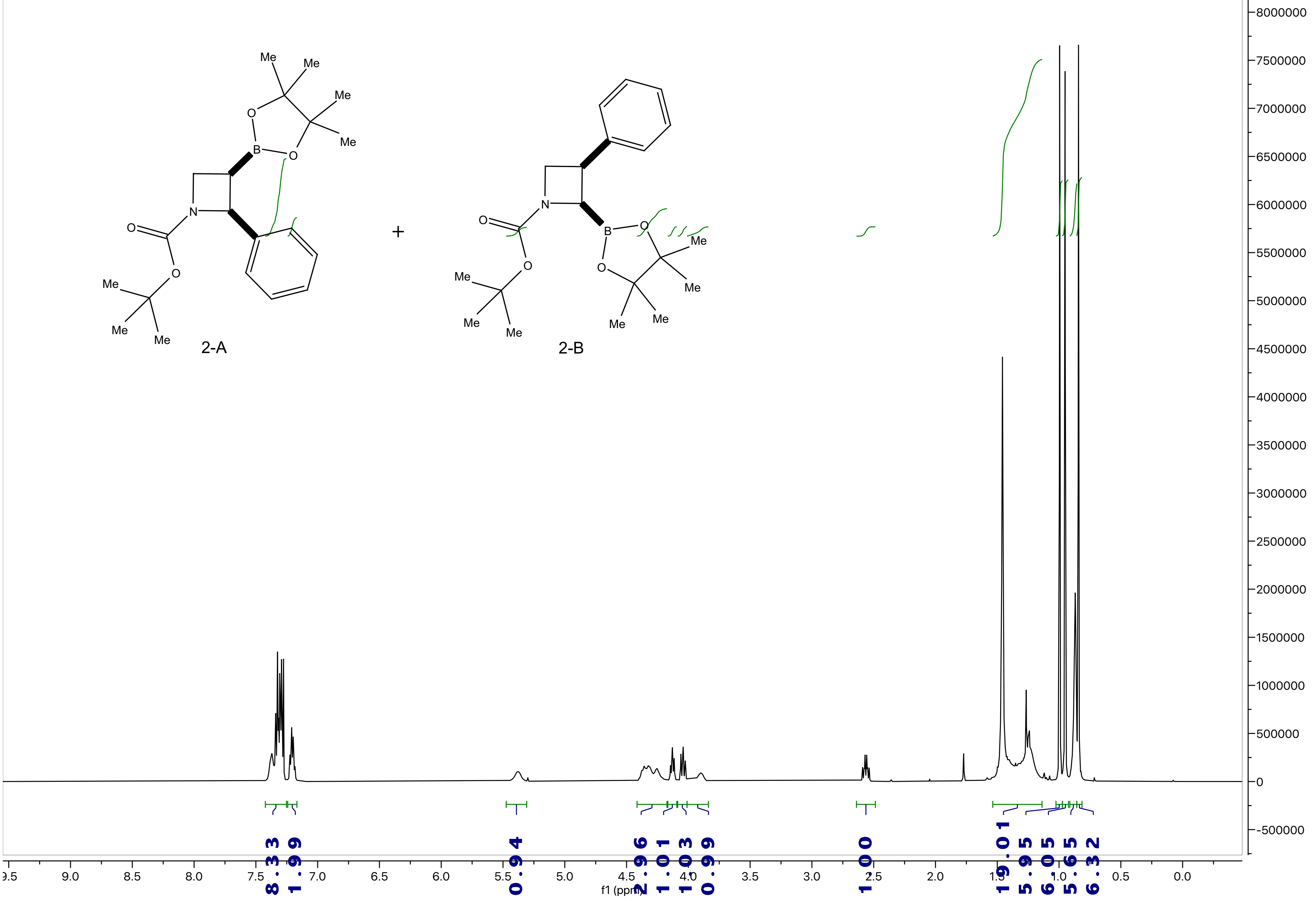

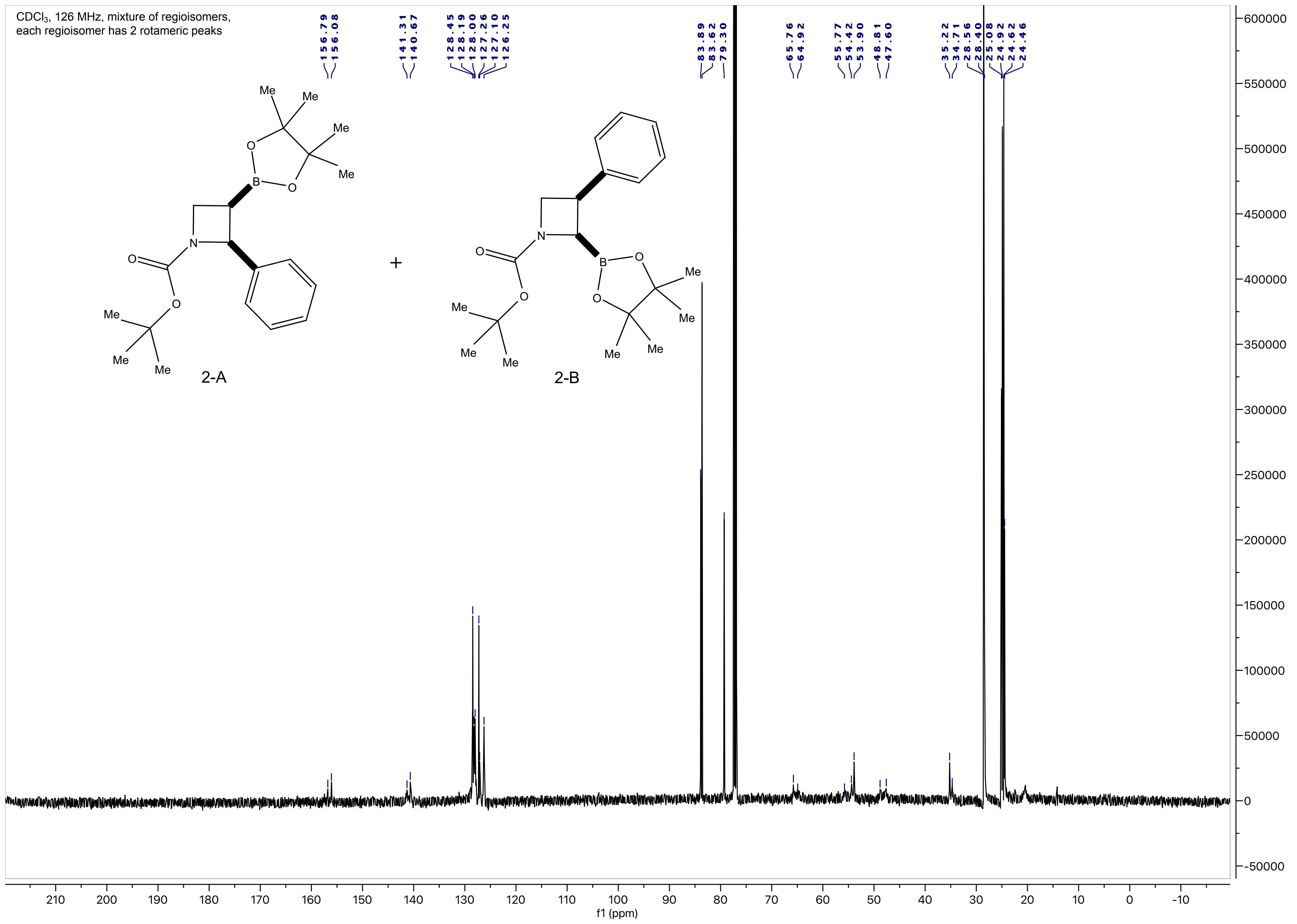

CDCl3, 126 MHz, mixture of regioisomers,
each regioisomer has 2 rotameric peaks
156.79
156.08
141.31
140.67
128.45
128.19
128.00
127.26
127.10
126.25
83.89
83.62
79.30
65.76
64.92
55.77
54.42
53.90
48.81
47.60
35.22
34.71
28.56
28.40
25.08
24.92
24.62
24.46
2-A
2-B
f1 (ppm)

CDCl$_3$, 500 MHz

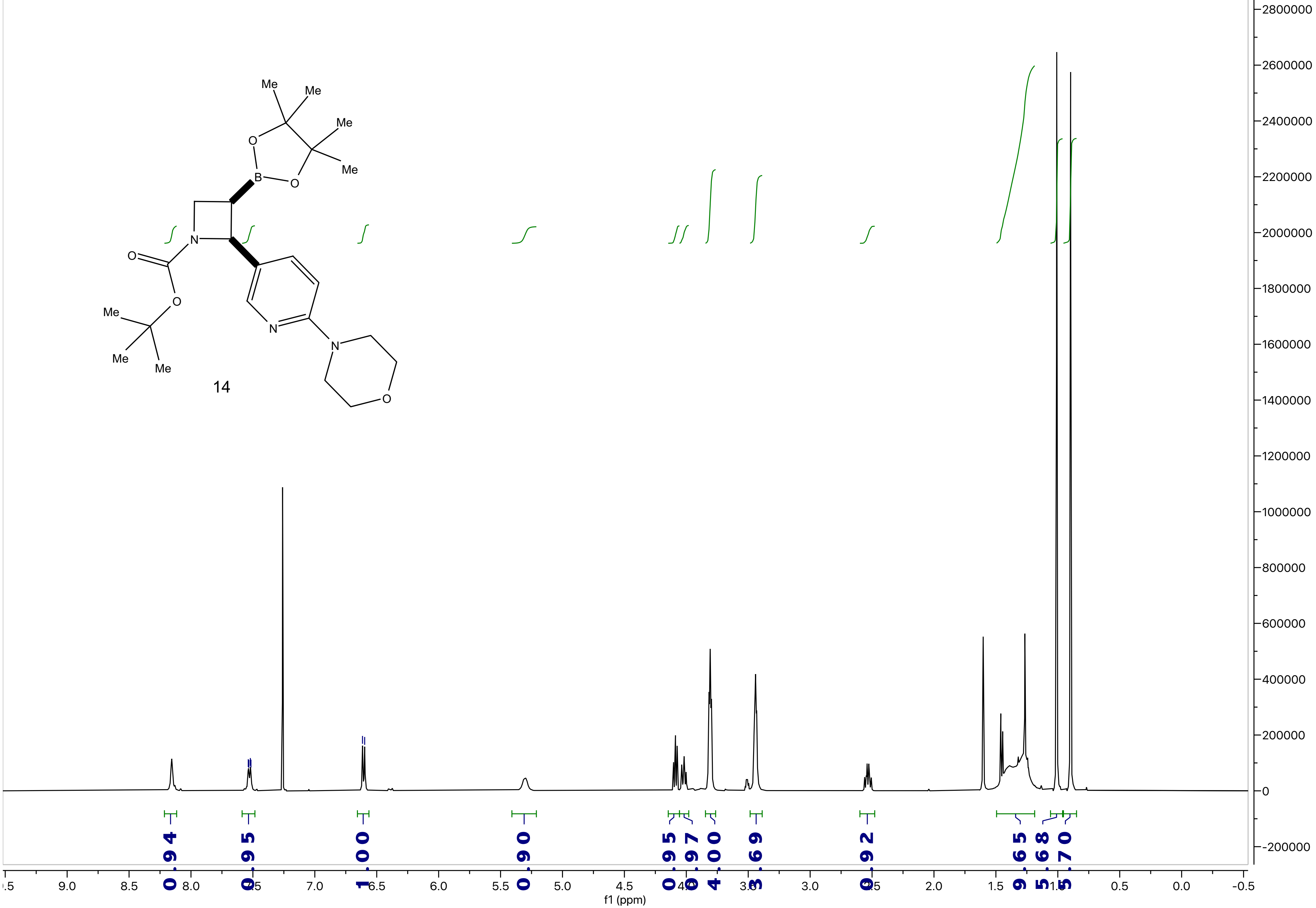

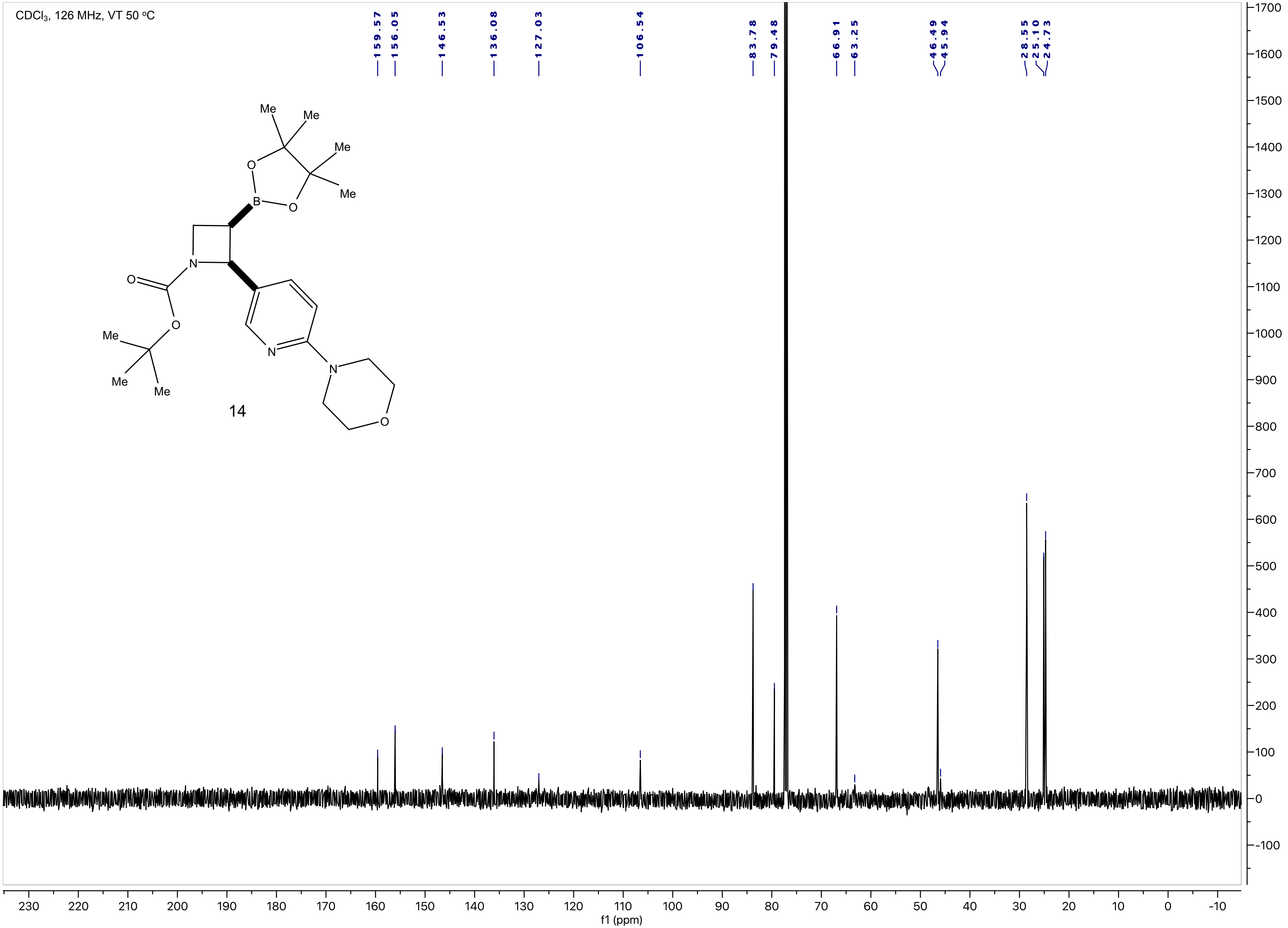

CDCl3, 126 MHz, VT 50 °C
159.57
156.05
146.53
136.08
127.03
106.54
83.78
79.48
66.91
63.25
46.49
45.94
28.55
25.10
24.73
Me
Me
Me
Me
O
B
O
N
O
O
Me
Me
Me
N
N
O
14
f1 (ppm)
230 220 210 200 190 180 170 160 150 140 130 120 110 100 90 80 70 60 50 40 30 20 10 0 -10
1700 1600 1500 1400 1300 1200 1100 1000 900 800 700 600 500 400 300 200 100 0 -100

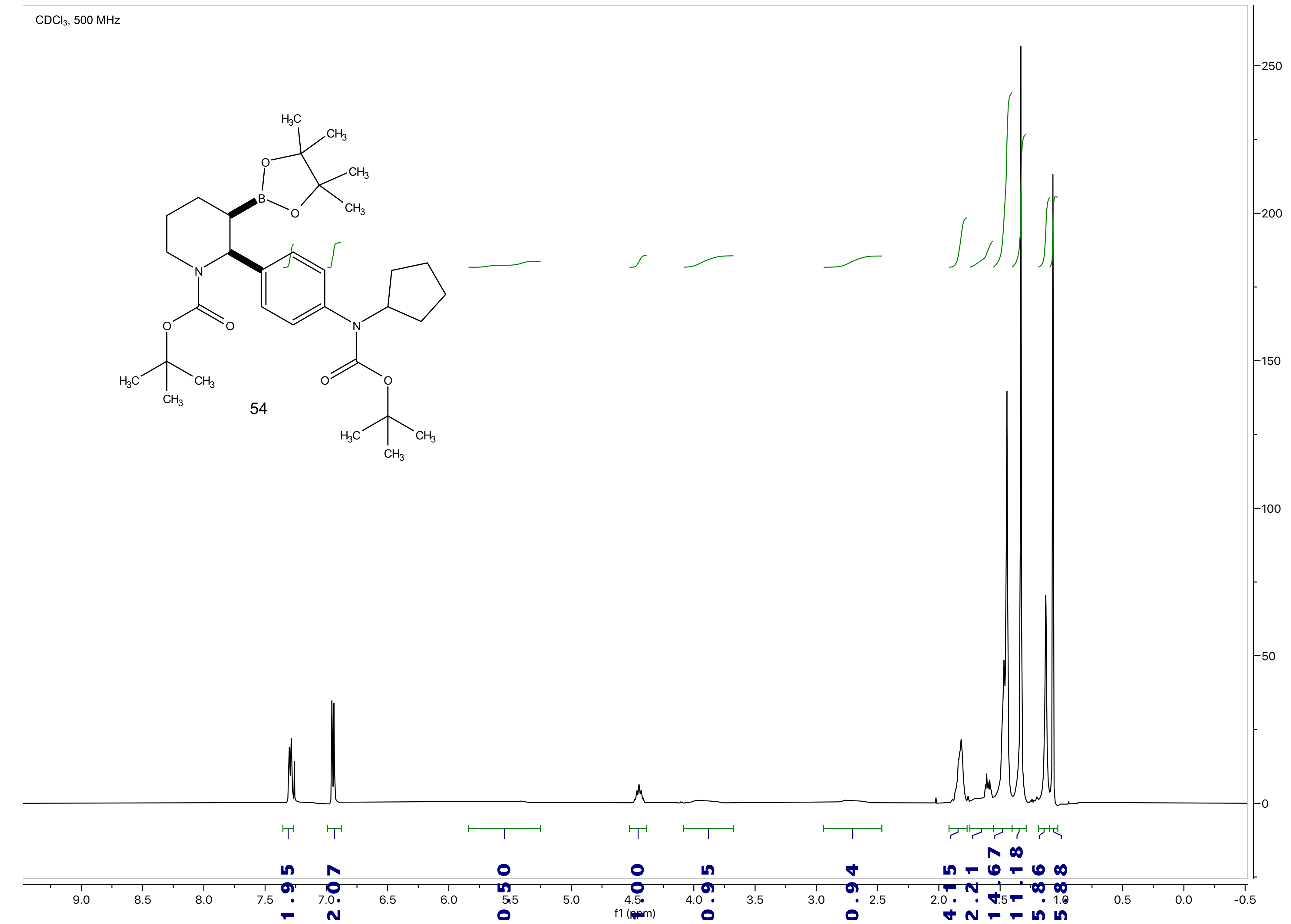
CDCl3, 500 MHz
54
1.95
2.07
0.50
1.00
0.95
0.94
4.15
2.21
14.67
11.18
5.86
5.88
9.0
8.5
8.0
7.5
7.0
6.5
6.0
5.5
5.0
4.5
4.0
3.5
3.0
2.5
2.0
1.5
1.0
0.5
0.0
-0.5
f1 (ppm)
250
200
150
100
50
0

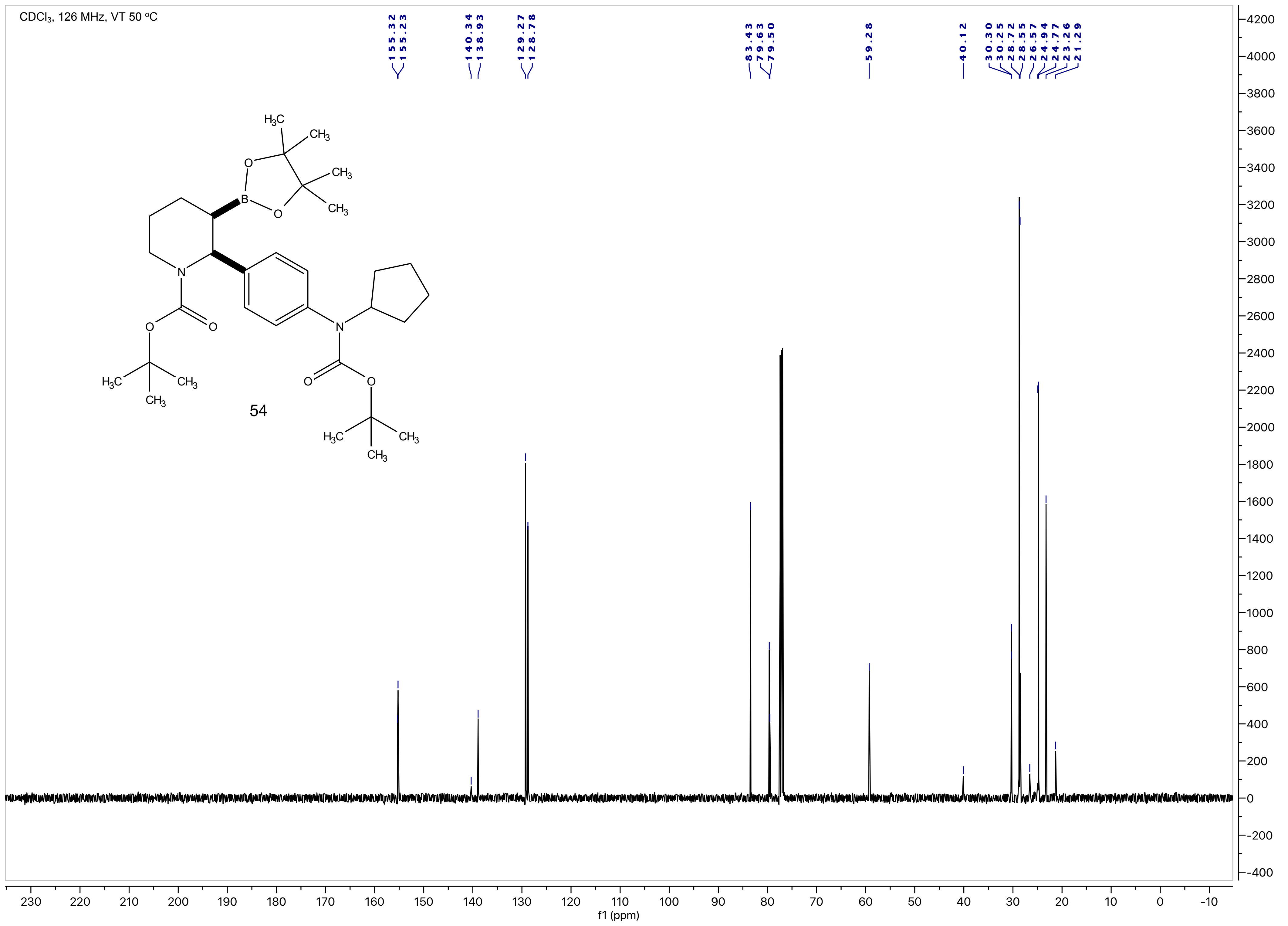

CDCl3, 126 MHz, VT 50 °C
155.32
155.23
140.34
138.93
129.27
128.78
83.43
79.63
79.50
59.28
40.12
30.30
30.25
28.72
28.55
26.57
24.94
24.77
23.26
21.29
54
f1 (ppm)

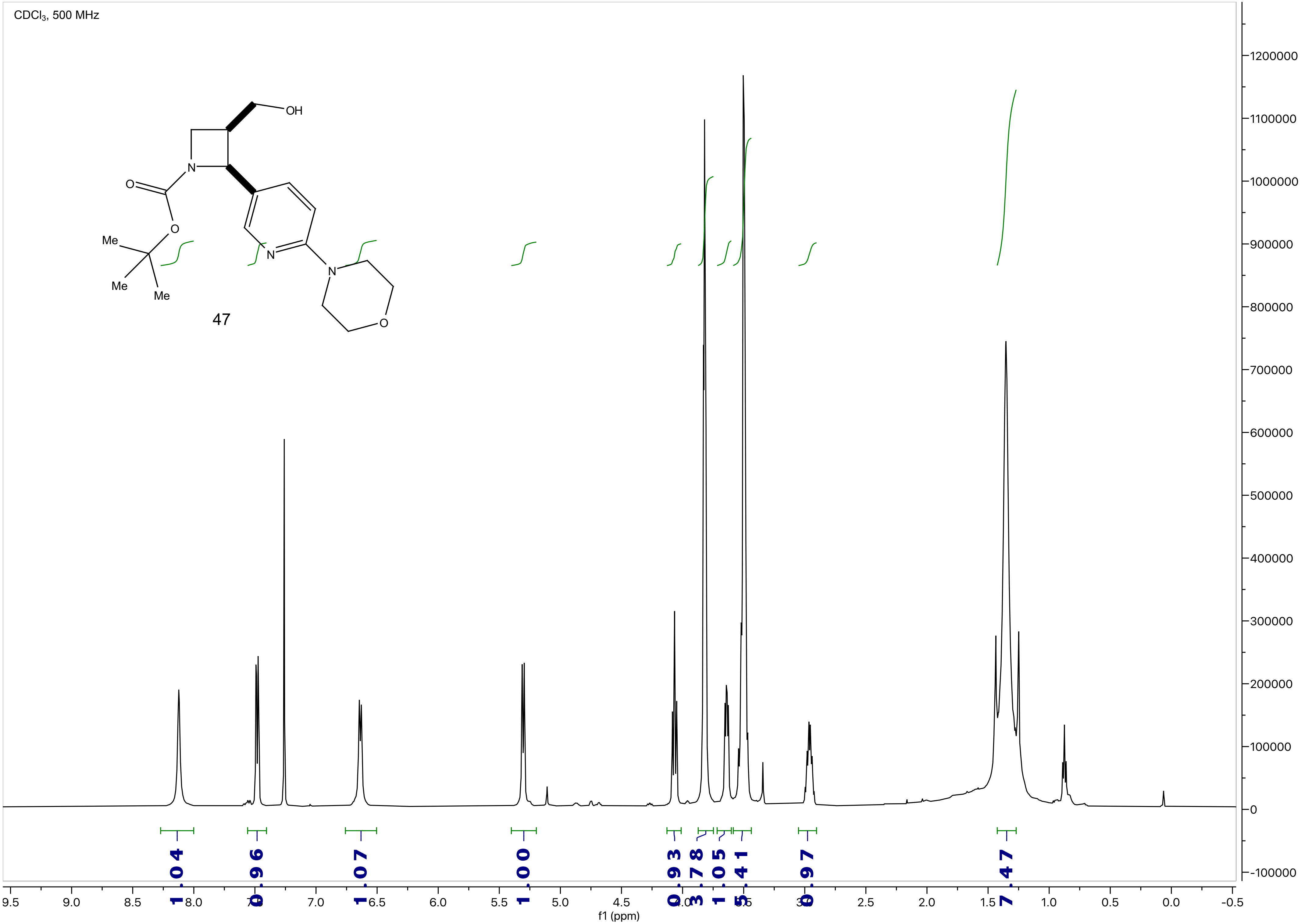

CDCl3, 500 MHz
OH
N
O
O
Me
Me
Me
N
N
O
47
1.04
0.96
1.07
1.00
0.93
3.78
1.05
5.41
0.97
7.47
9.5
9.0
8.5
8.0
7.0
6.5
6.0
5.5
5.0
4.5
2.5
2.0
1.5
1.0
0.5
0.0
-0.5
f1 (ppm)
1200000
1100000
1000000
900000
800000
700000
600000
500000
400000
300000
200000
100000
0
-100000

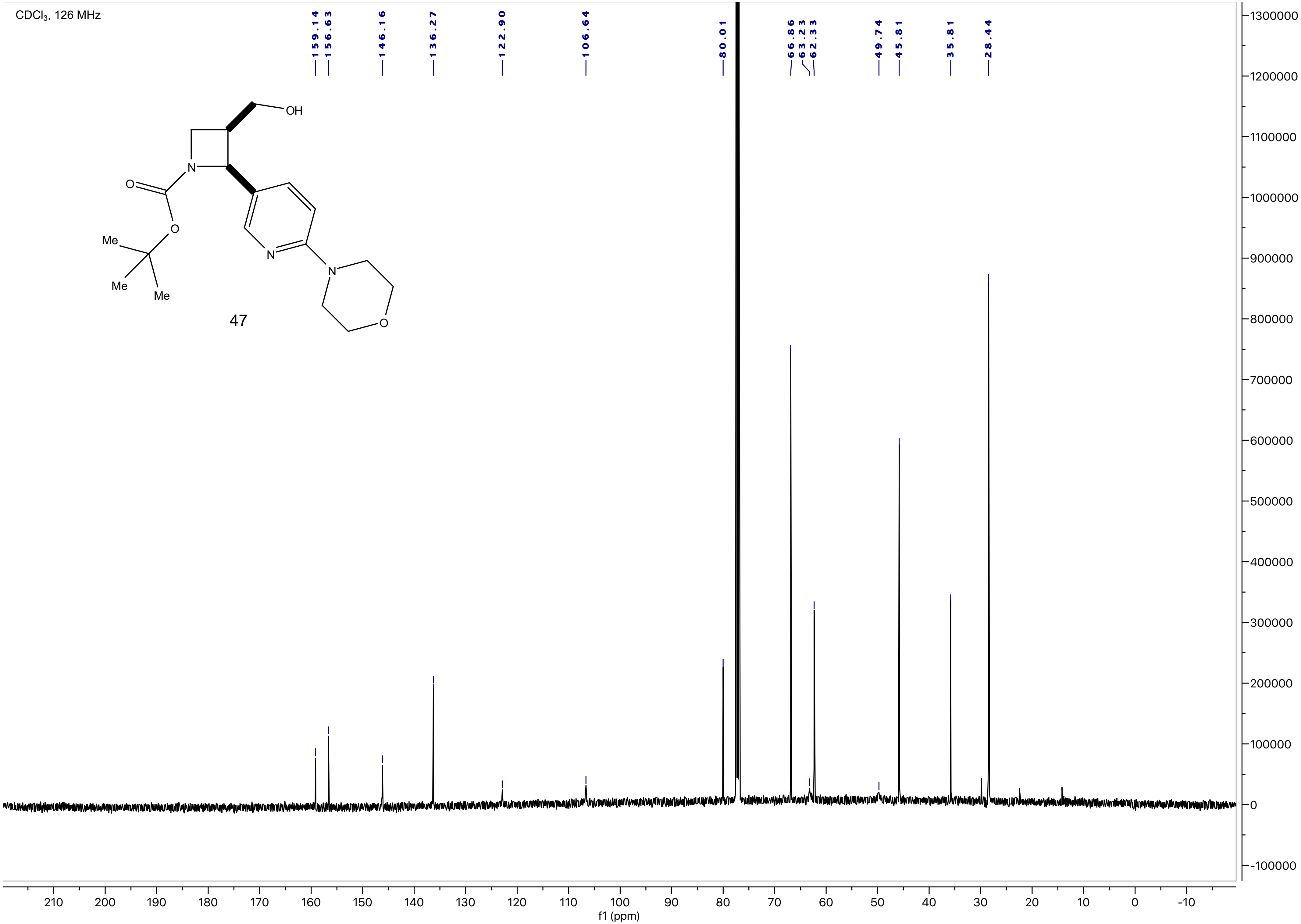
CDCl3, 126 MHz
159.14
156.63
146.16
136.27
122.90
106.64
80.01
66.86
63.23
62.33
49.74
45.81
35.81
28.44
OH
N
O
O
Me
Me
Me
N
N
O
47
f1 (ppm)

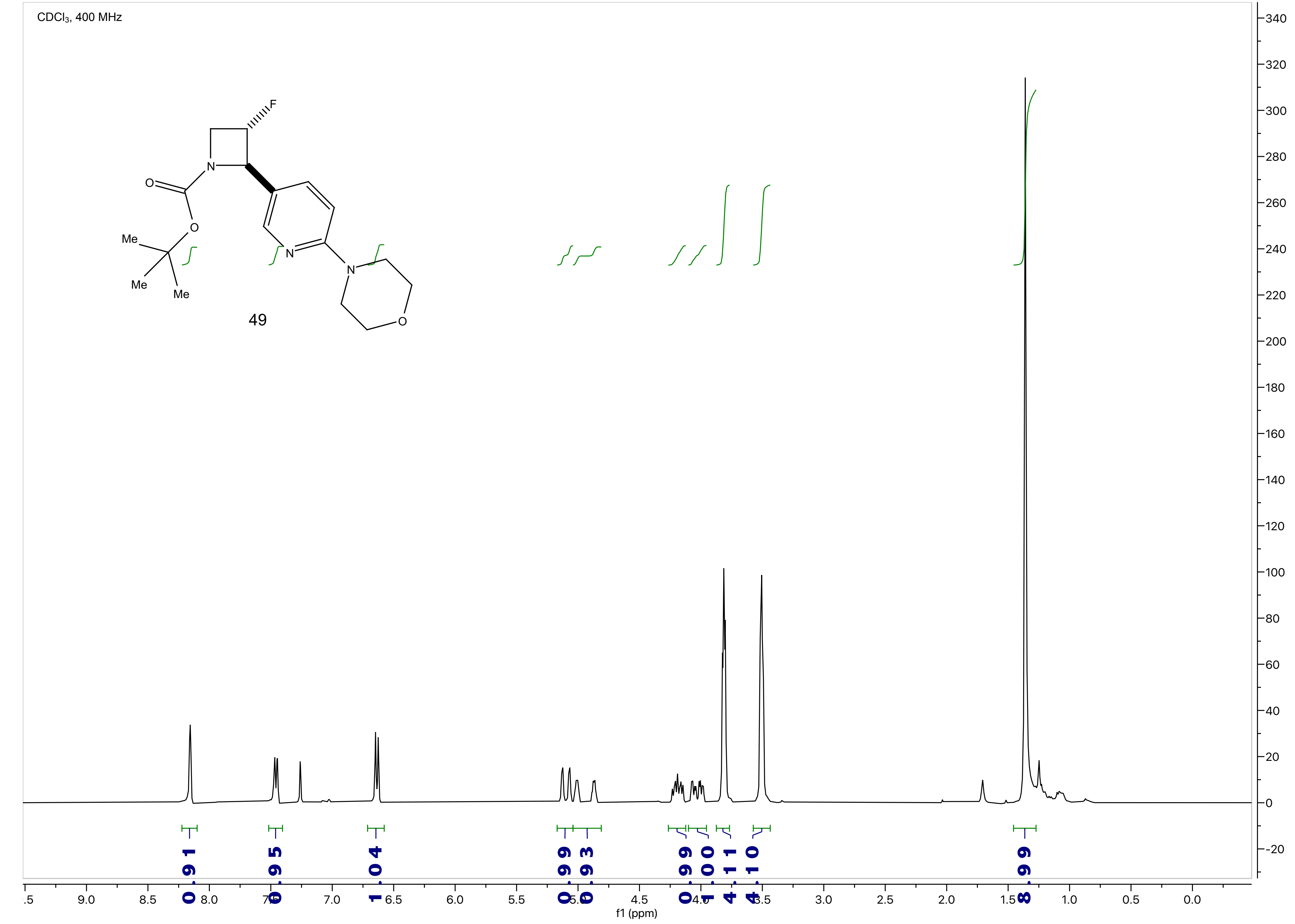
CDCl3, 400 MHz
F
N
O
O
Me
Me
Me
N
N
O
49
0.91
0.95
1.04
0.99
0.93
0.99
1.00
4.11
4.10
8.99
f1 (ppm)

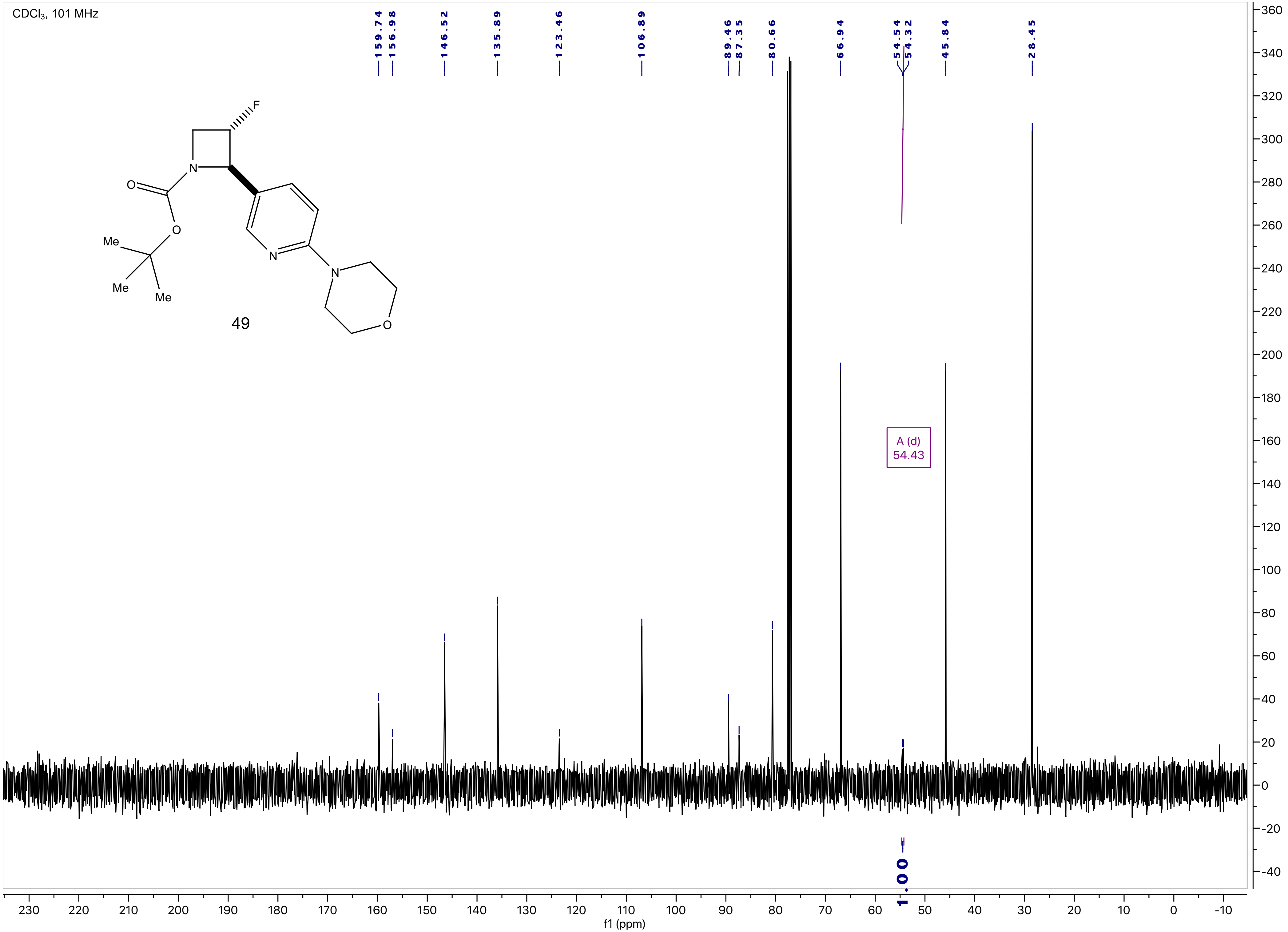
CDCl3, 101 MHz
159.74
156.98
146.52
135.89
123.46
106.89
89.46
87.35
80.66
66.94
54.54
54.32
45.84
28.45
F
N
O
O
Me
Me
Me
N
N
O
49
A (d)
54.43
1.00
f1 (ppm)

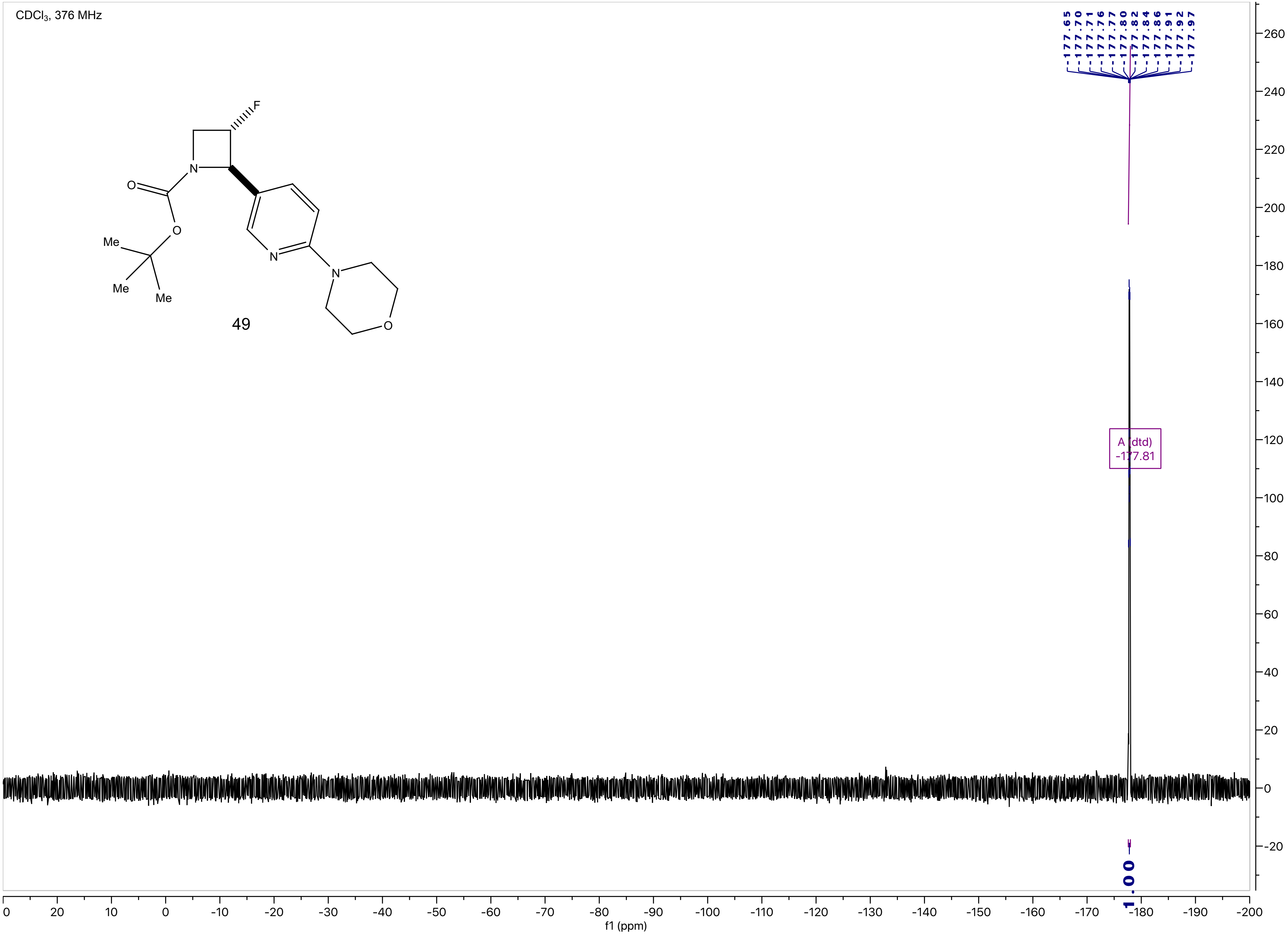
CDCl$_3$, 376 MHz
-177.65
-177.70
-177.71
-177.76
-177.77
-177.80
-177.82
-177.84
-177.86
-177.91
-177.92
-177.97
F
N
O
O
Me
Me
Me
N
N
O
49
A (dtd)
-177.81
1.00
f1 (ppm)

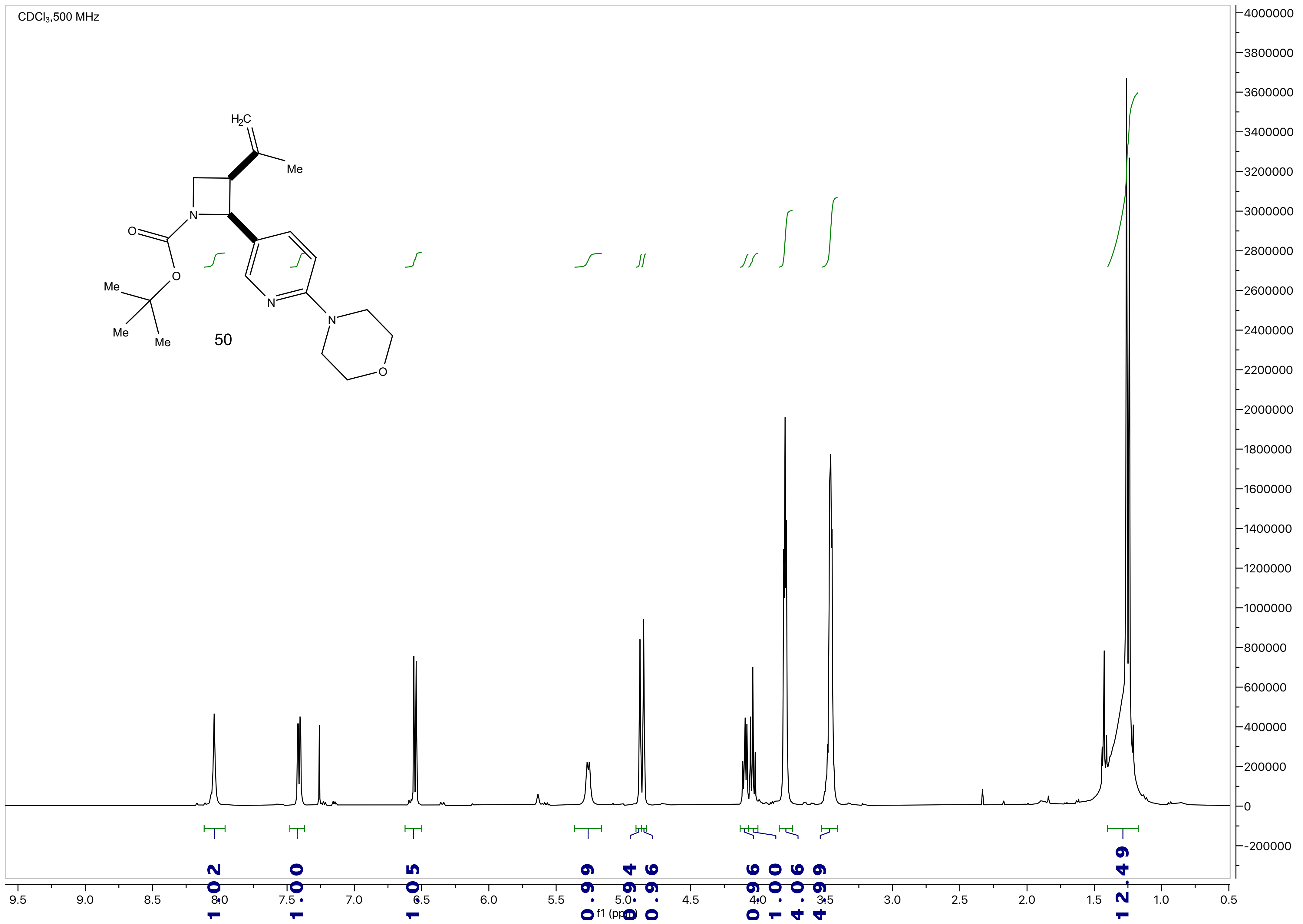
CDCl3,500 MHz
50
f1 (ppm)
1.02
1.00
1.05
0.99
0.94
0.96
0.96
1.00
4.06
4.99
12.49

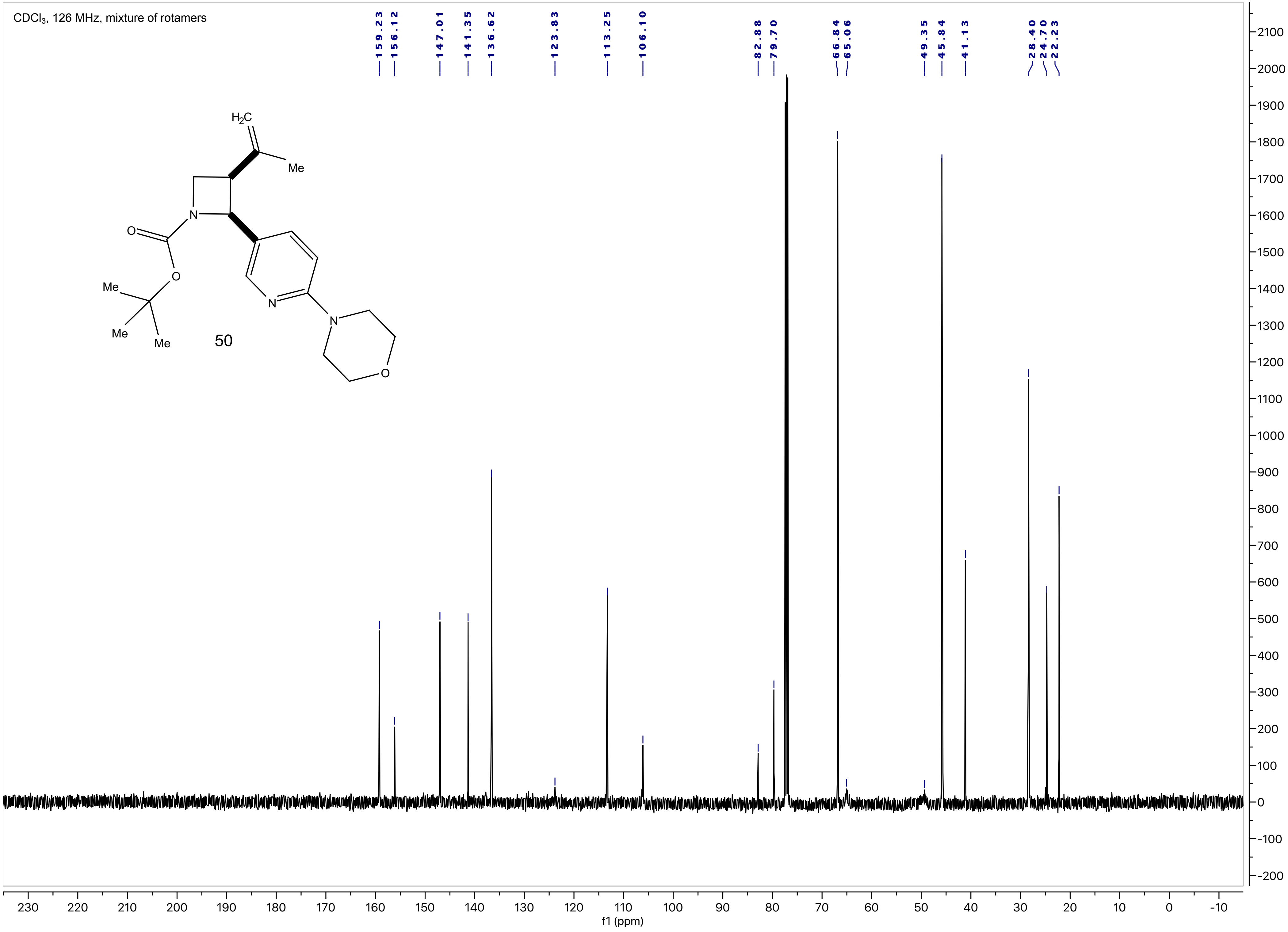
CDCl$_3$, 126 MHz, mixture of rotamers
159.23
156.12
147.01
141.35
136.62
123.83
113.25
106.10
82.88
79.70
66.84
65.06
49.35
45.84
41.13
28.40
24.70
22.23
H$_2$C
Me
N
O
O
Me
Me
Me
N
N
O
50
f1 (ppm)

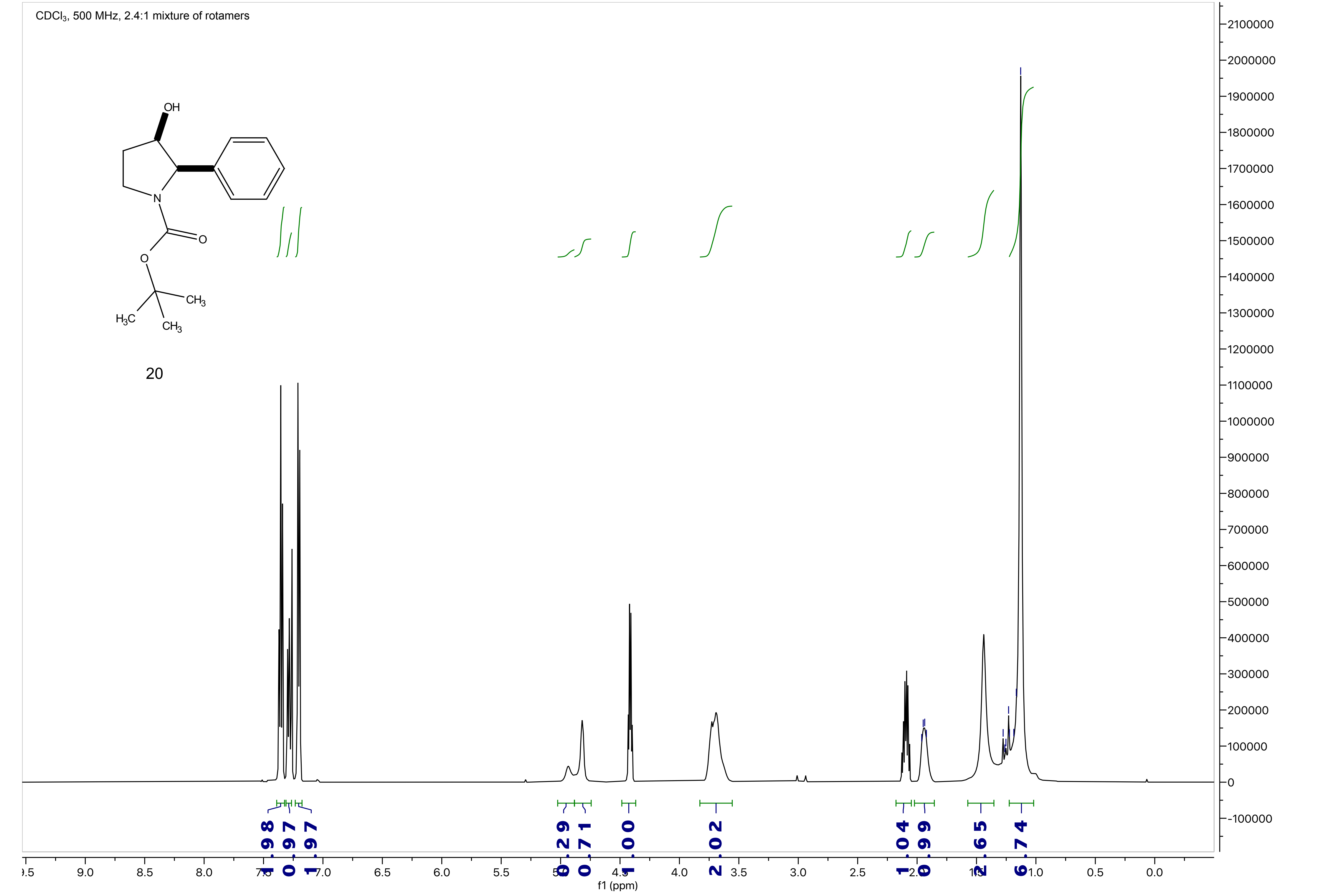
CDCl3, 500 MHz, 2.4:1 mixture of rotamers
OH
N
O
O
CH3
H3C
CH3
20
1.98
0.97
1.97
0.29
0.71
1.00
2.02
1.04
0.99
2.65
6.74
f1 (ppm)

CDCl$_3$, 126 MHz, mixture of rotamers

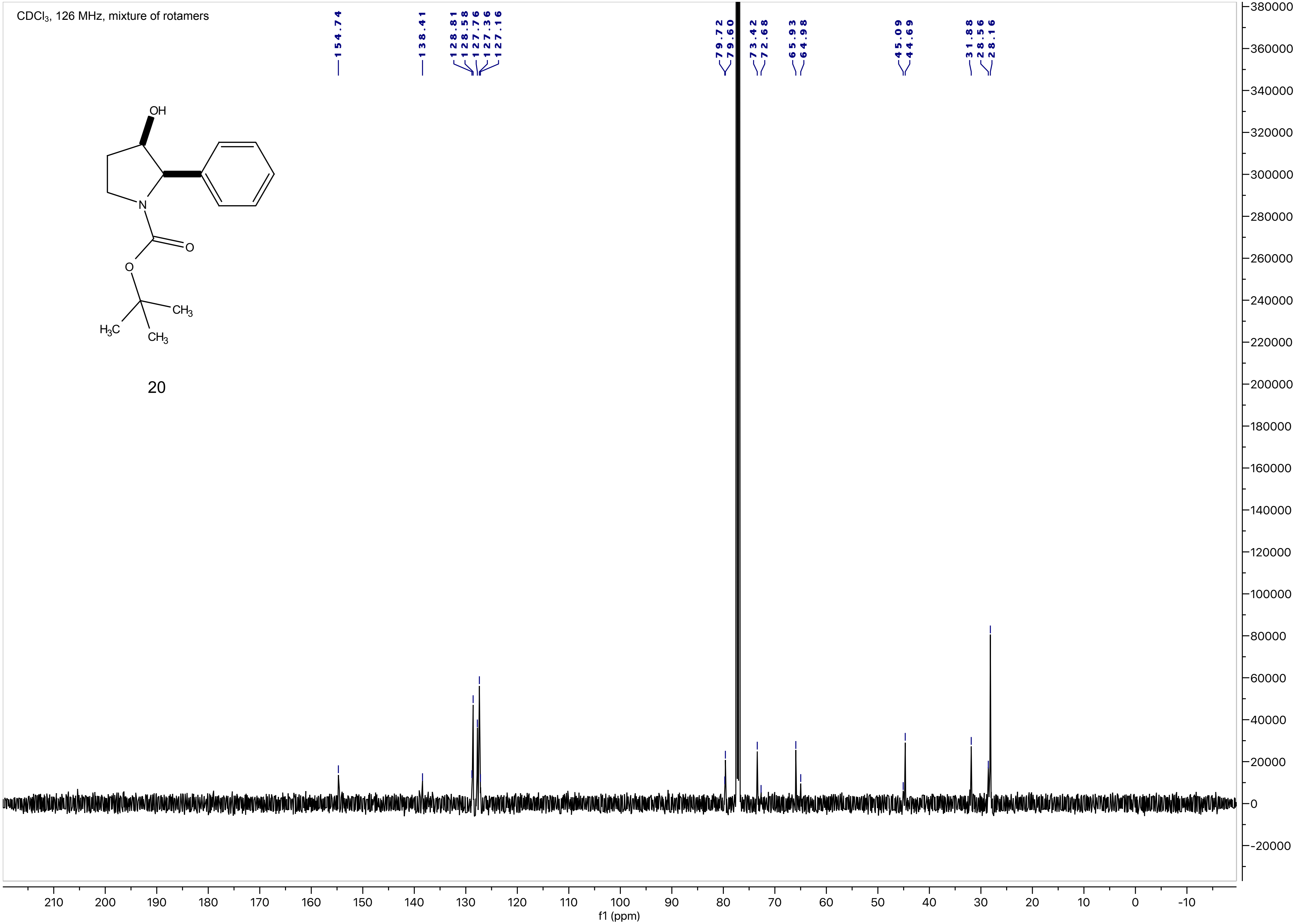

20

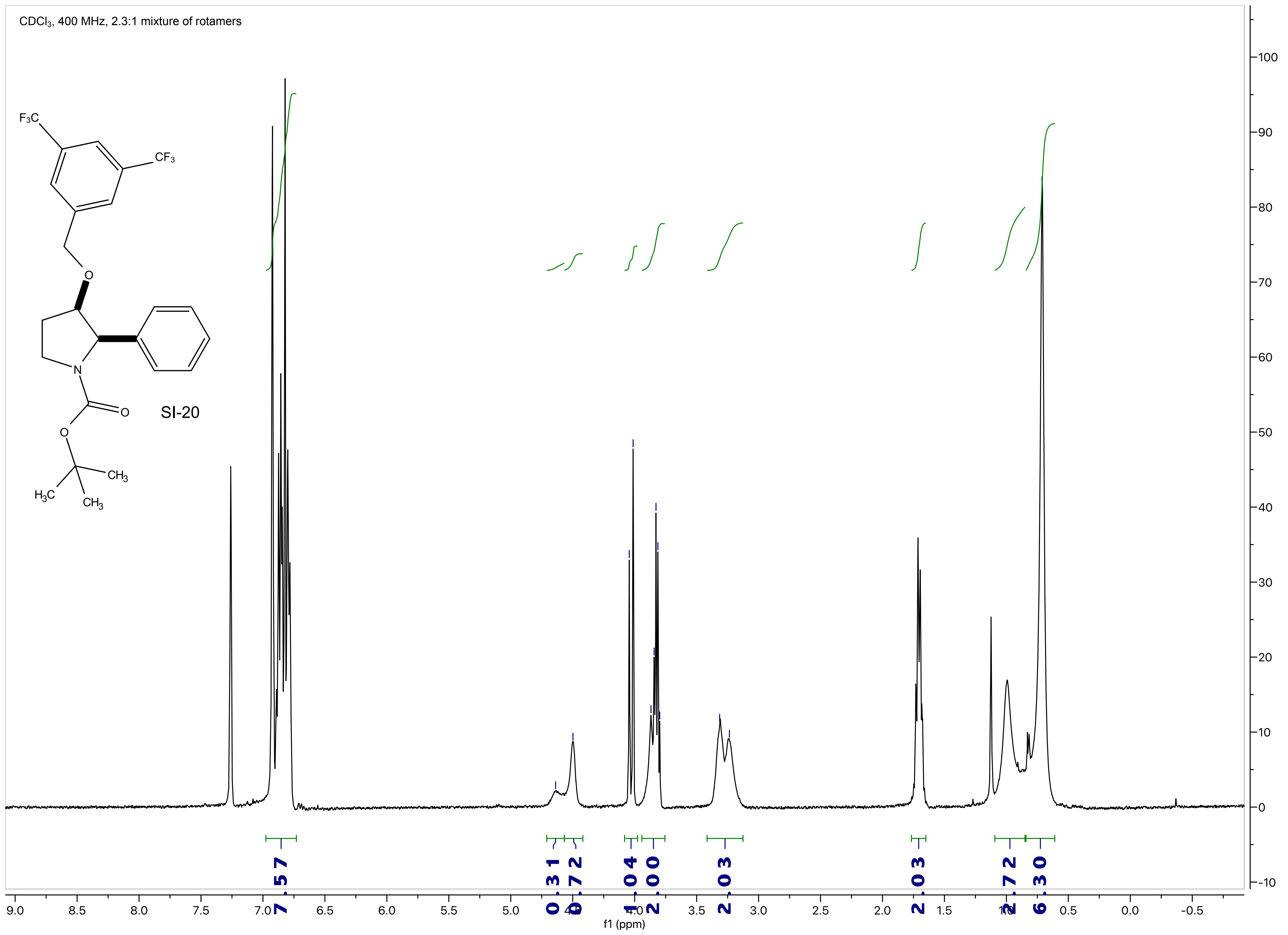

CDCl$_3$, 400 MHz, 2.3:1 mixture of rotamers
F$_3$C
CF$_3$
O
N
O
O
H$_3$C
CH$_3$
CH$_3$
SI-20
7.57
0.31
0.72
1.04
2.00
2.03
2.03
2.72
6.30
f1 (ppm)
9.0 8.5 8.0 7.5 7.0 6.5 6.0 5.5 5.0 4.5 4.0 3.5 3.0 2.5 2.0 1.5 1.0 0.5 0.0 -0.5
100 90 80 70 60 50 40 30 20 10 0 -10

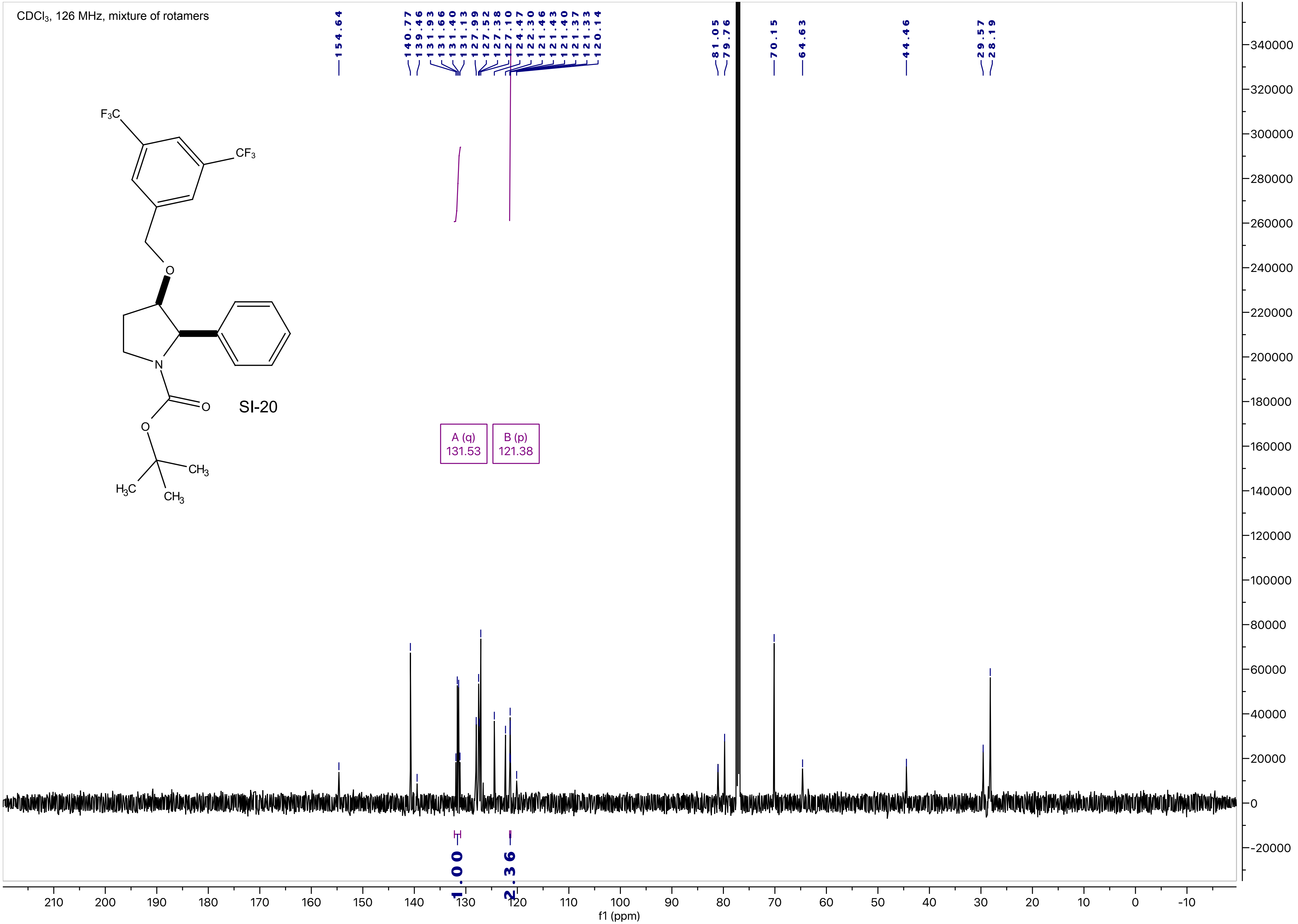
CDCl$_3$, 126 MHz, mixture of rotamers
154.64
140.77
139.46
131.93
131.66
131.40
131.13
127.99
127.52
127.38
127.10
124.47
122.30
121.46
121.43
121.40
121.37
121.33
120.14
81.05
79.76
70.15
64.63
44.46
29.57
28.19
F_3C
CF_3
O
N
O
O
CH_3
H_3C
CH_3
SI-20
A (q)
131.53
B (p)
121.38
1.00
2.36
f1 (ppm)

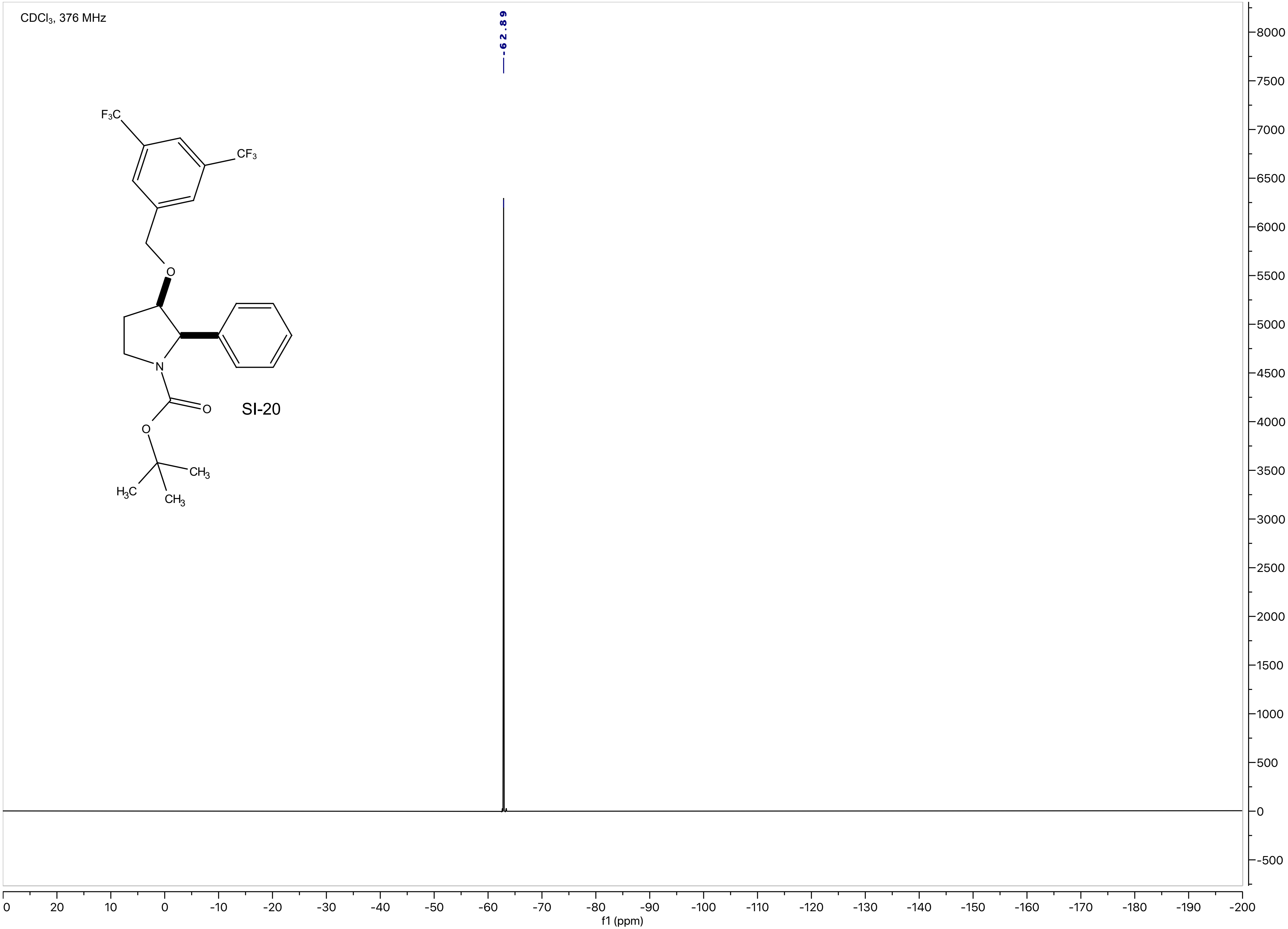

CDCl3, 376 MHz
-62.89
F3C
CF3
O
N
O
O
CH3
H3C
CH3
SI-20
f1 (ppm)

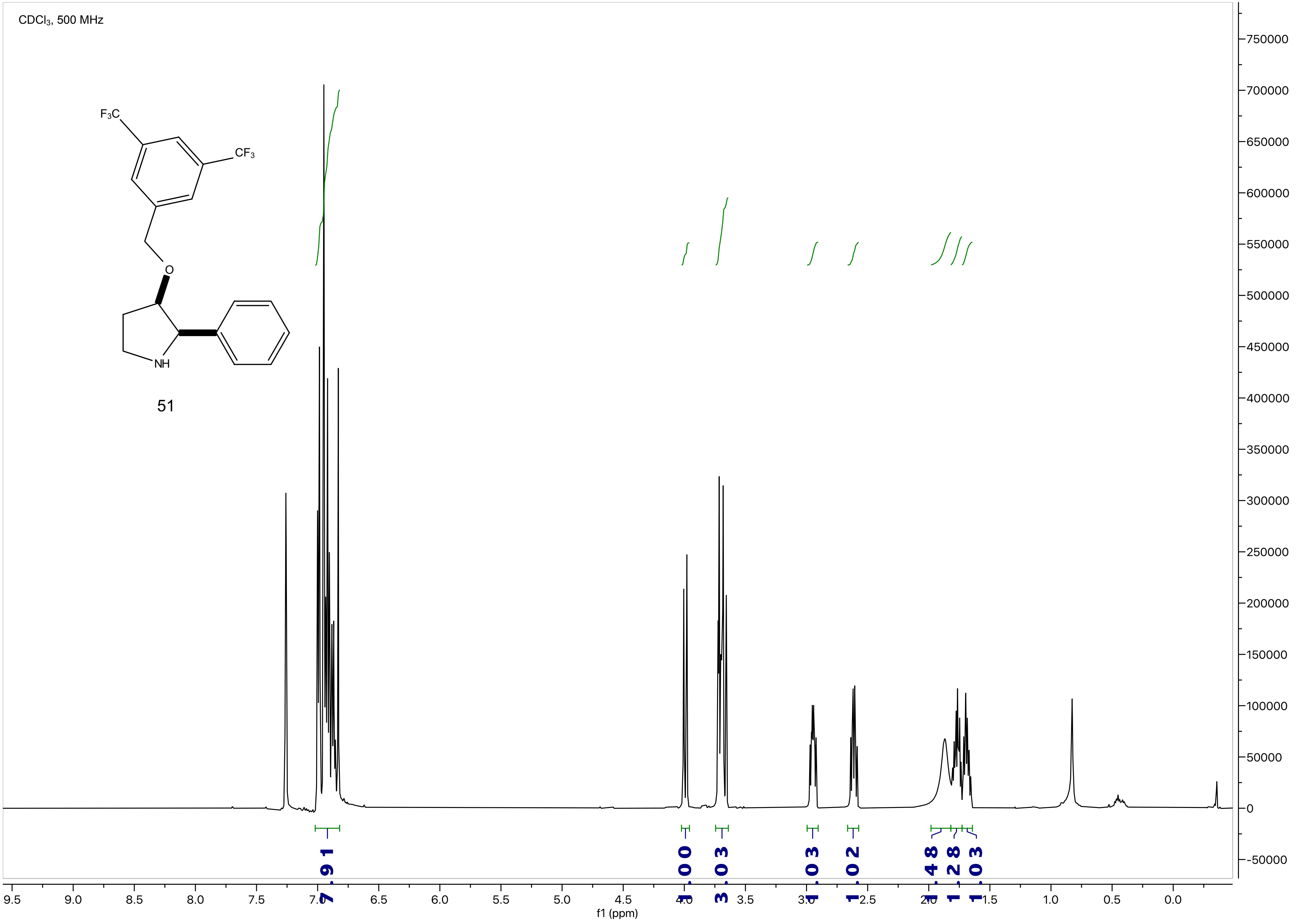
CDCl$_3$, 500 MHz
F$_3$C
CF$_3$
O
NH
51
7.91
1.00
3.03
1.03
1.02
1.48
1.28
1.03
f1 (ppm)

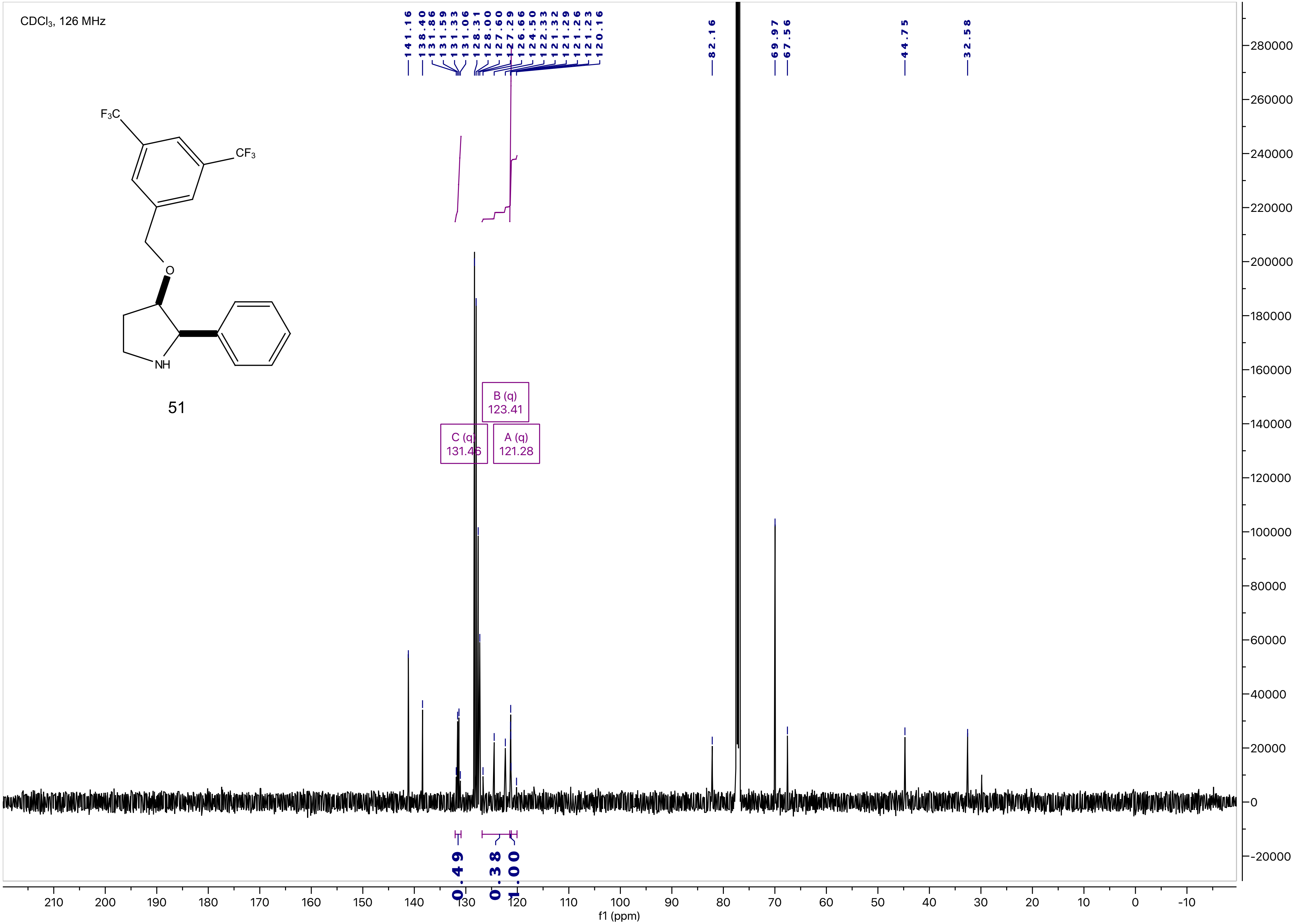

CDCl$_3$, 126 MHz
141.16
138.40
131.86
131.59
131.33
131.06
128.31
128.00
127.60
127.29
126.66
124.50
122.33
121.32
121.29
121.26
121.23
120.16
82.16
69.97
67.56
44.75
32.58
F$_3$C
CF$_3$
O
NH
51
B (q)
123.41
C (q)
131.46
A (q)
121.28
0.49
0.38
1.00
f1 (ppm)

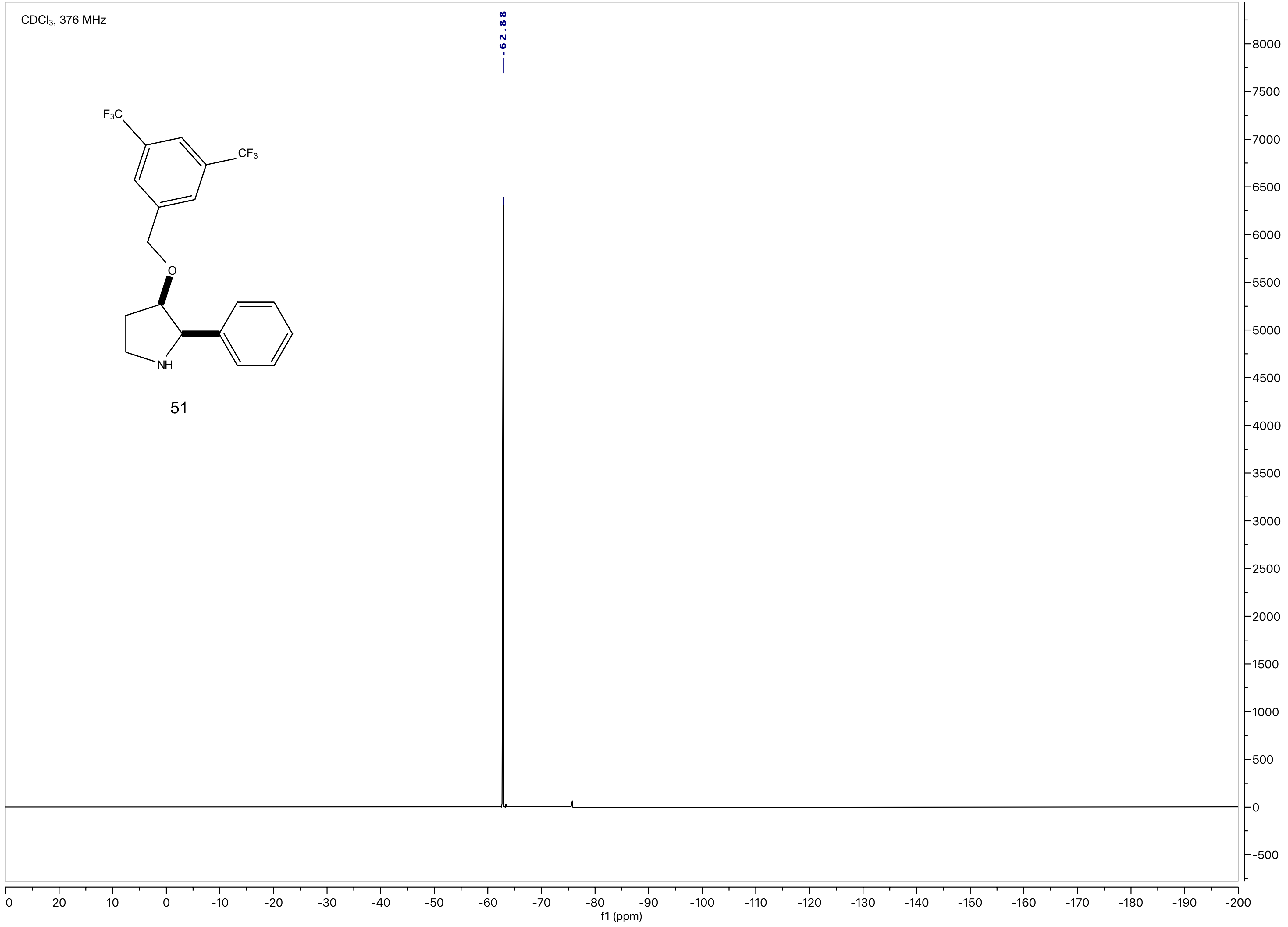
CDCl3, 376 MHz
-62.88
F3C
CF3
O
NH
51
f1 (ppm)

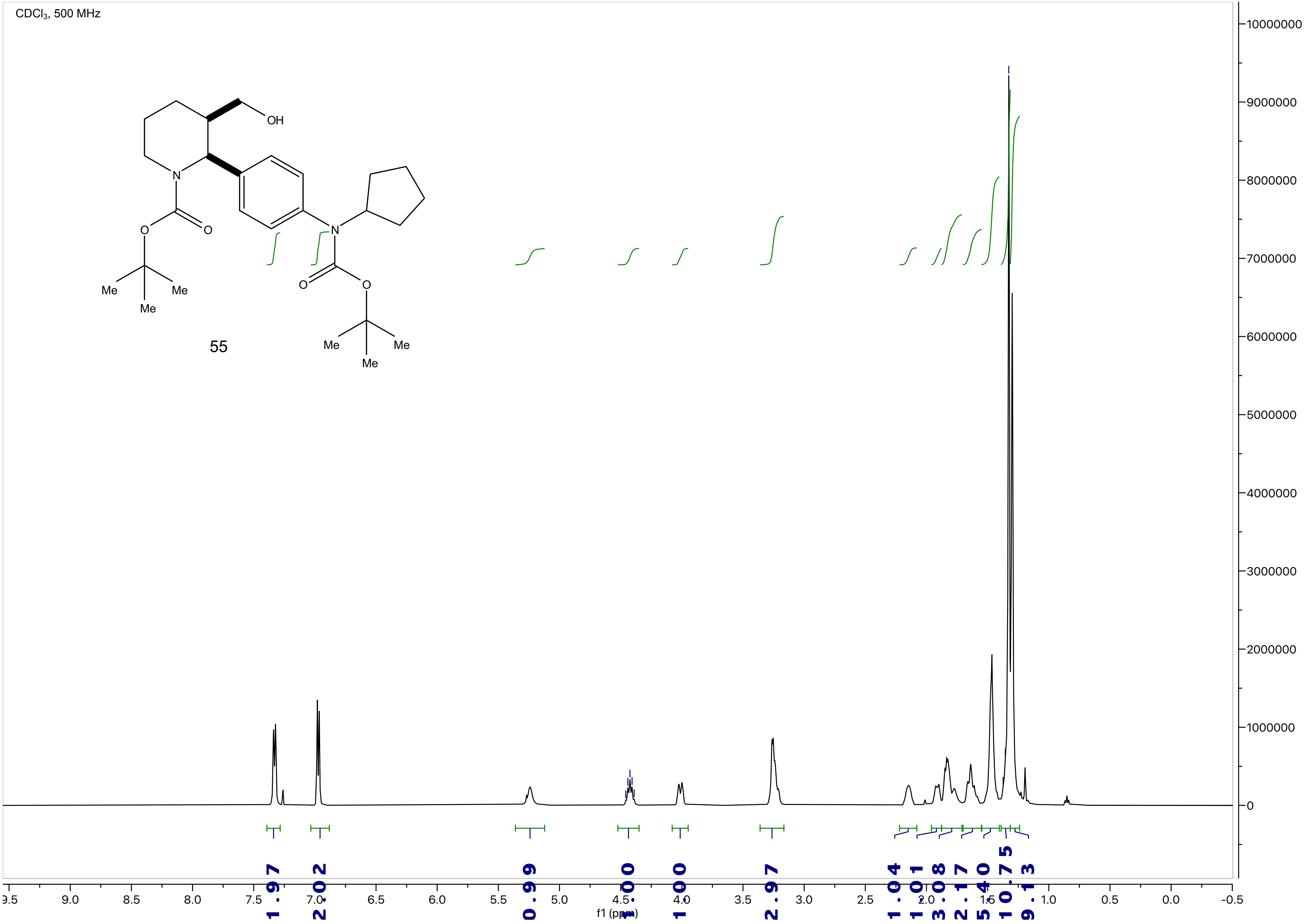
CDCl$_3$, 500 MHz
OH
N
O
O
Me
Me
Me
55
N
O
O
Me
Me
Me
10000000
9000000
8000000
7000000
6000000
5000000
4000000
3000000
2000000
1000000
0
1.97
2.02
0.99
1.00
1.00
2.97
1.04
1.01
3.08
2.17
5.40
10.75
9.13
9.5
9.0
8.5
8.0
7.5
7.0
6.5
6.0
5.5
5.0
4.5
4.0
3.5
3.0
2.5
2.0
1.5
1.0
0.5
0.0
-0.5
f1 (ppm)

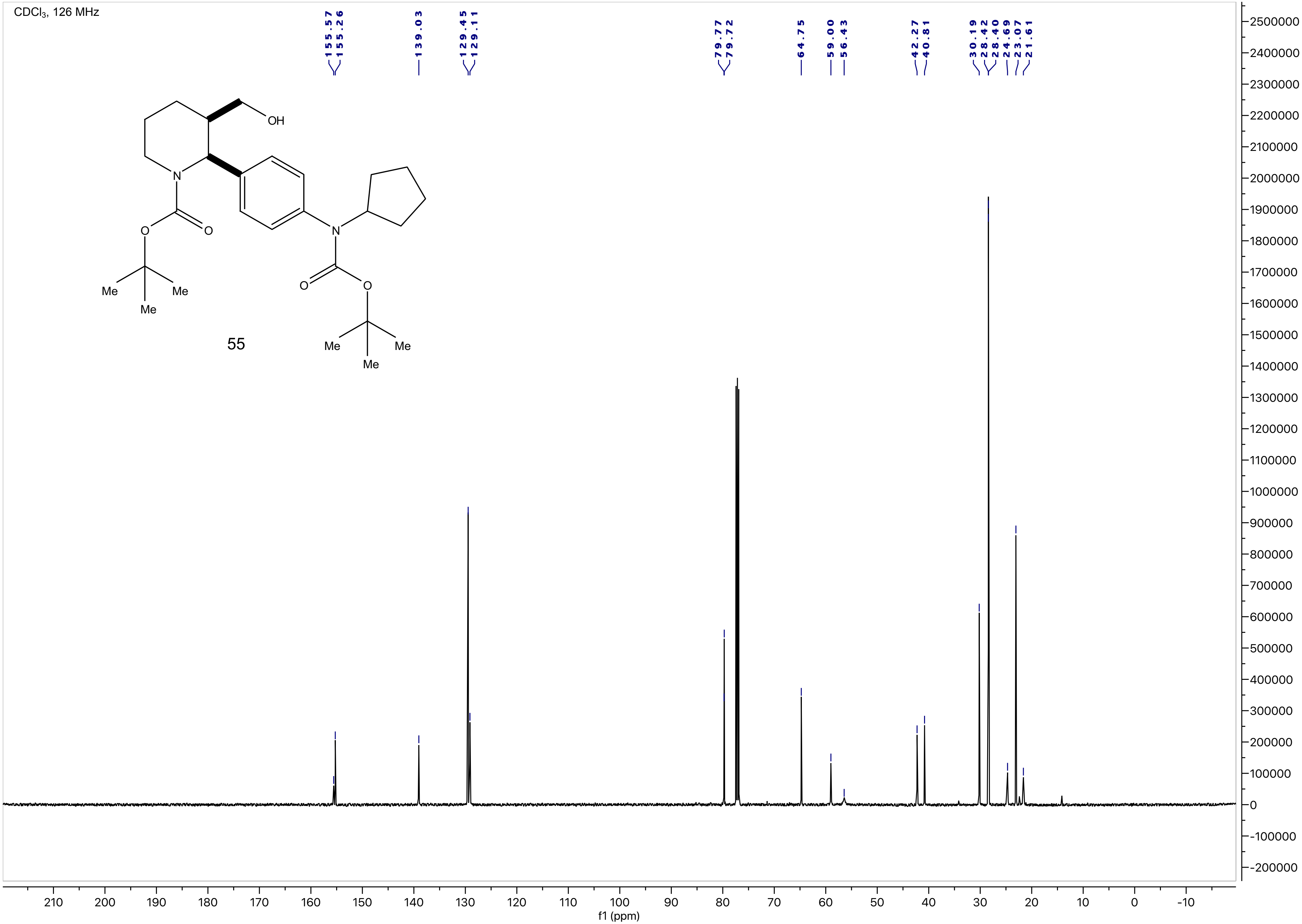
CDCl$_3$, 126 MHz
155.57
155.26
139.03
129.45
129.11
79.77
79.72
64.75
59.00
56.43
42.27
40.81
30.19
28.42
28.40
24.69
23.07
21.61
OH
N
O
O
Me
Me
Me
N
O
O
Me
Me
Me
55
f1 (ppm)

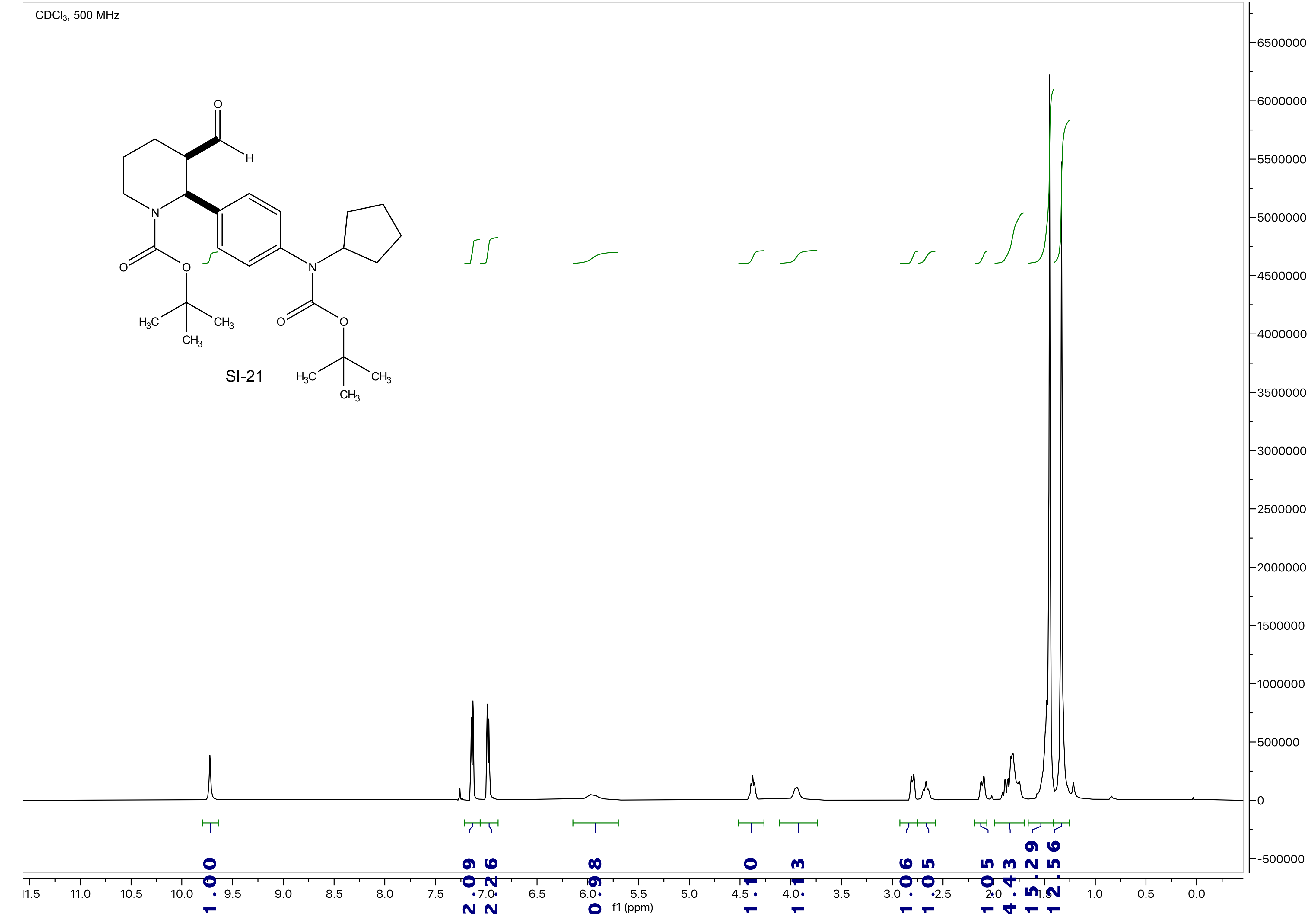
CDCl3, 500 MHz
SI-21
f1 (ppm)
1.00
2.09
2.26
0.98
1.10
1.13
1.06
1.05
1.05
4.43
15.29
12.56

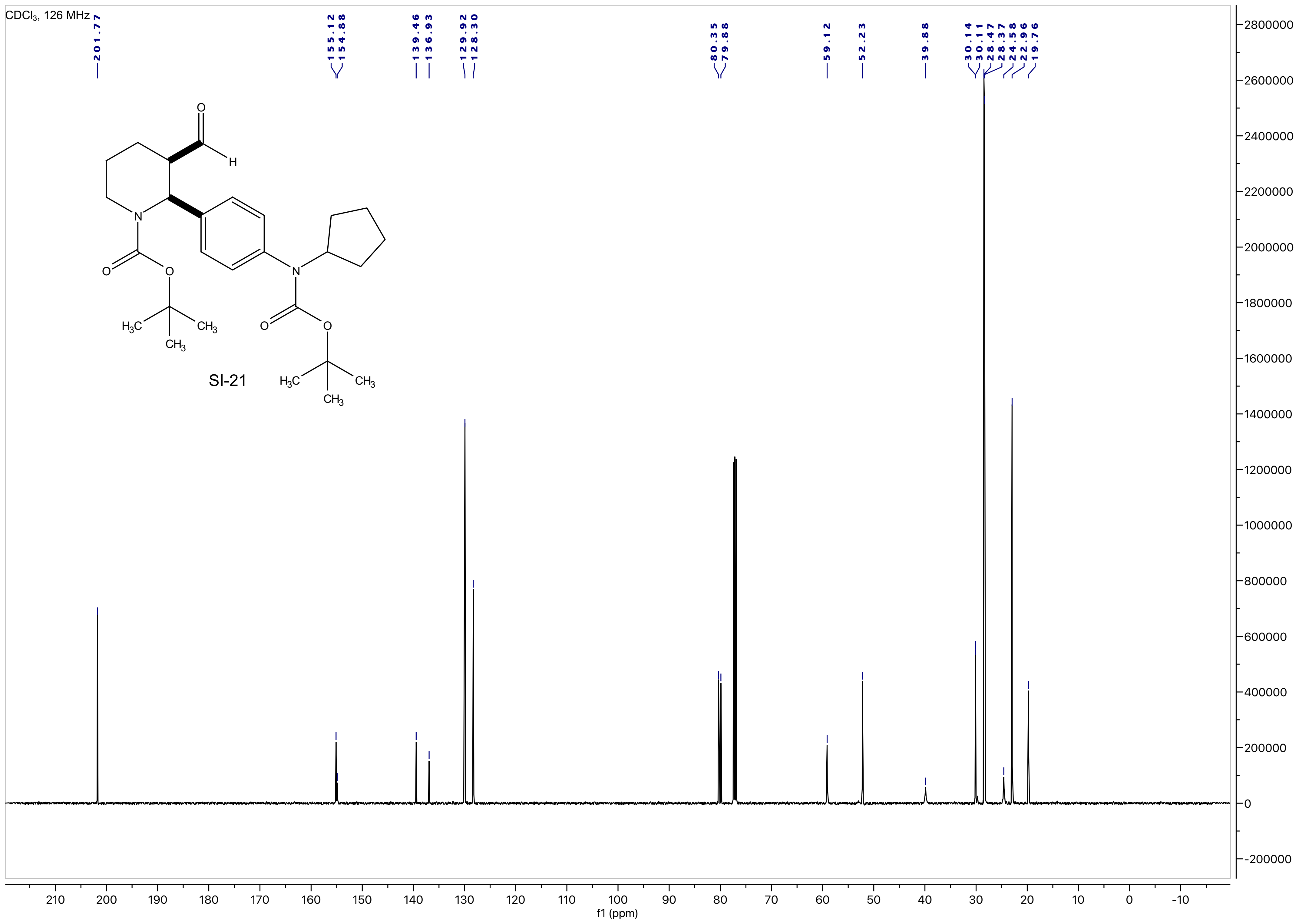

CDCl3, 126 MHz
201.77
155.12
154.88
139.46
136.93
129.92
128.30
80.35
79.88
59.12
52.23
39.88
30.14
30.11
28.47
28.37
24.58
22.96
19.76
SI-21
f1 (ppm)

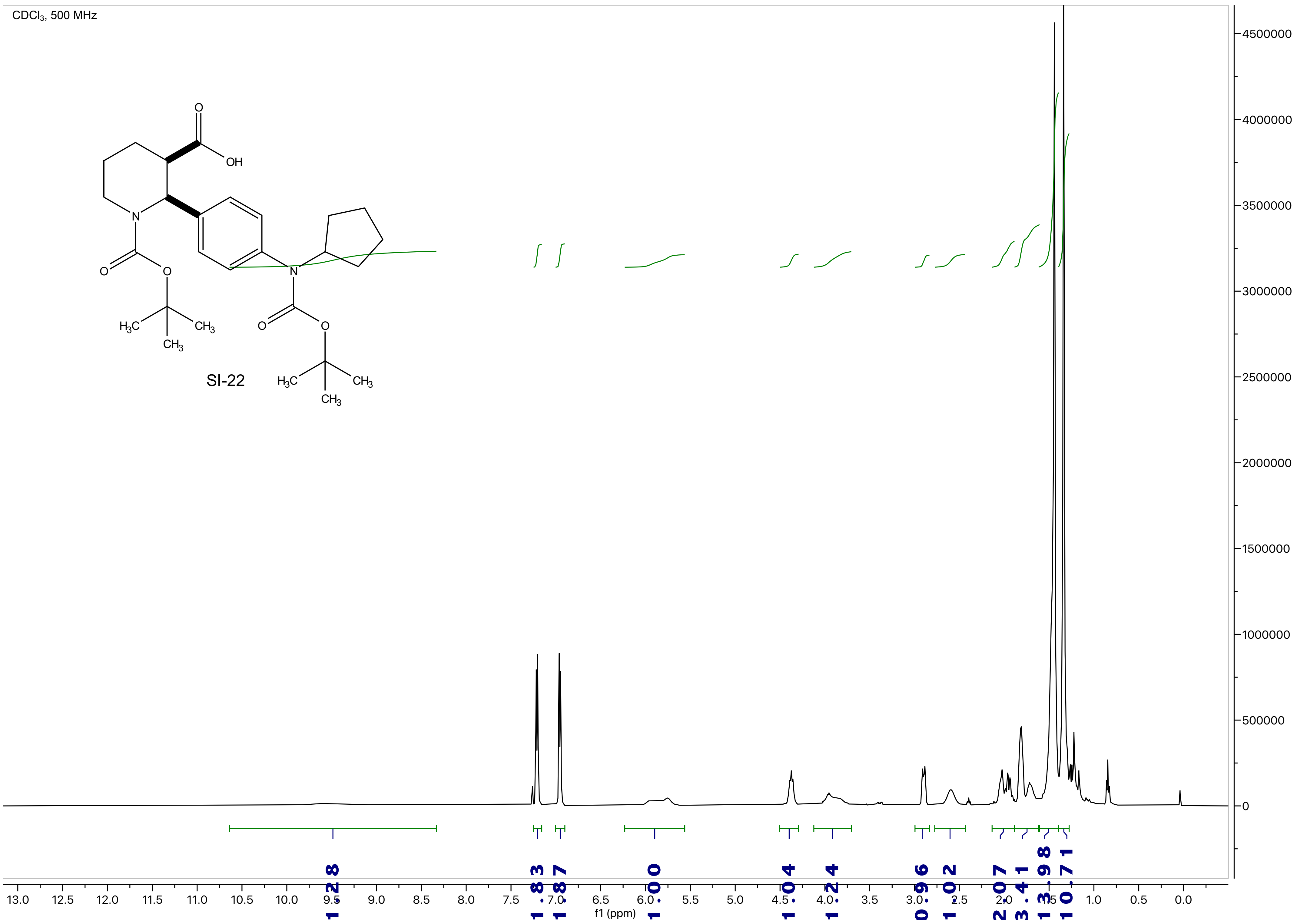

CDCl3, 500 MHz
SI-22
f1 (ppm)

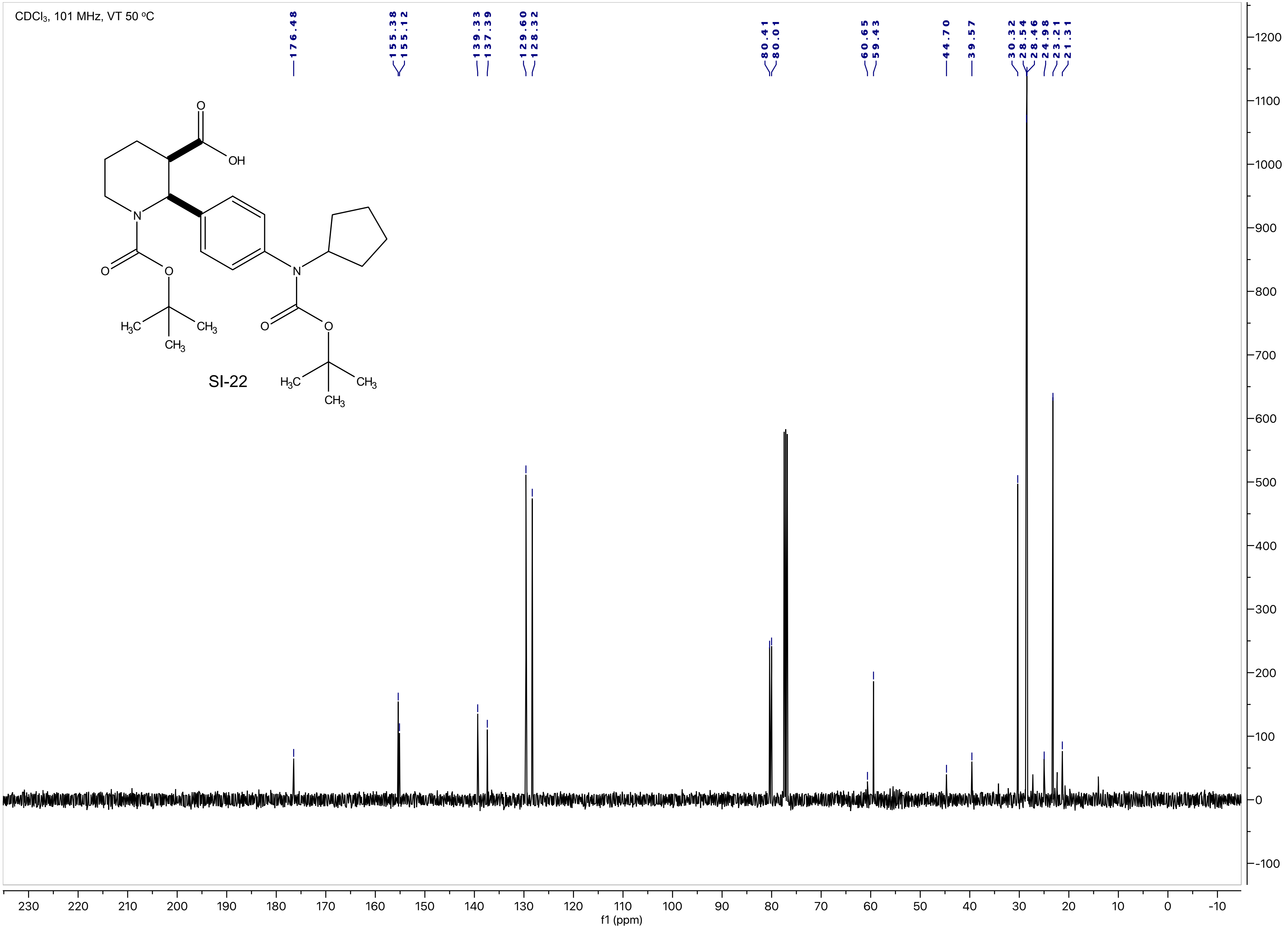

CDCl3, 101 MHz, VT 50 ºC
176.48
155.38
155.12
139.33
137.39
129.60
128.32
80.41
80.01
60.65
59.43
44.70
39.57
30.32
28.54
28.46
24.98
23.21
21.31
O
OH
N
O
O
H3C
CH3
CH3
N
O
O
H3C
CH3
CH3
SI-22
f1 (ppm)

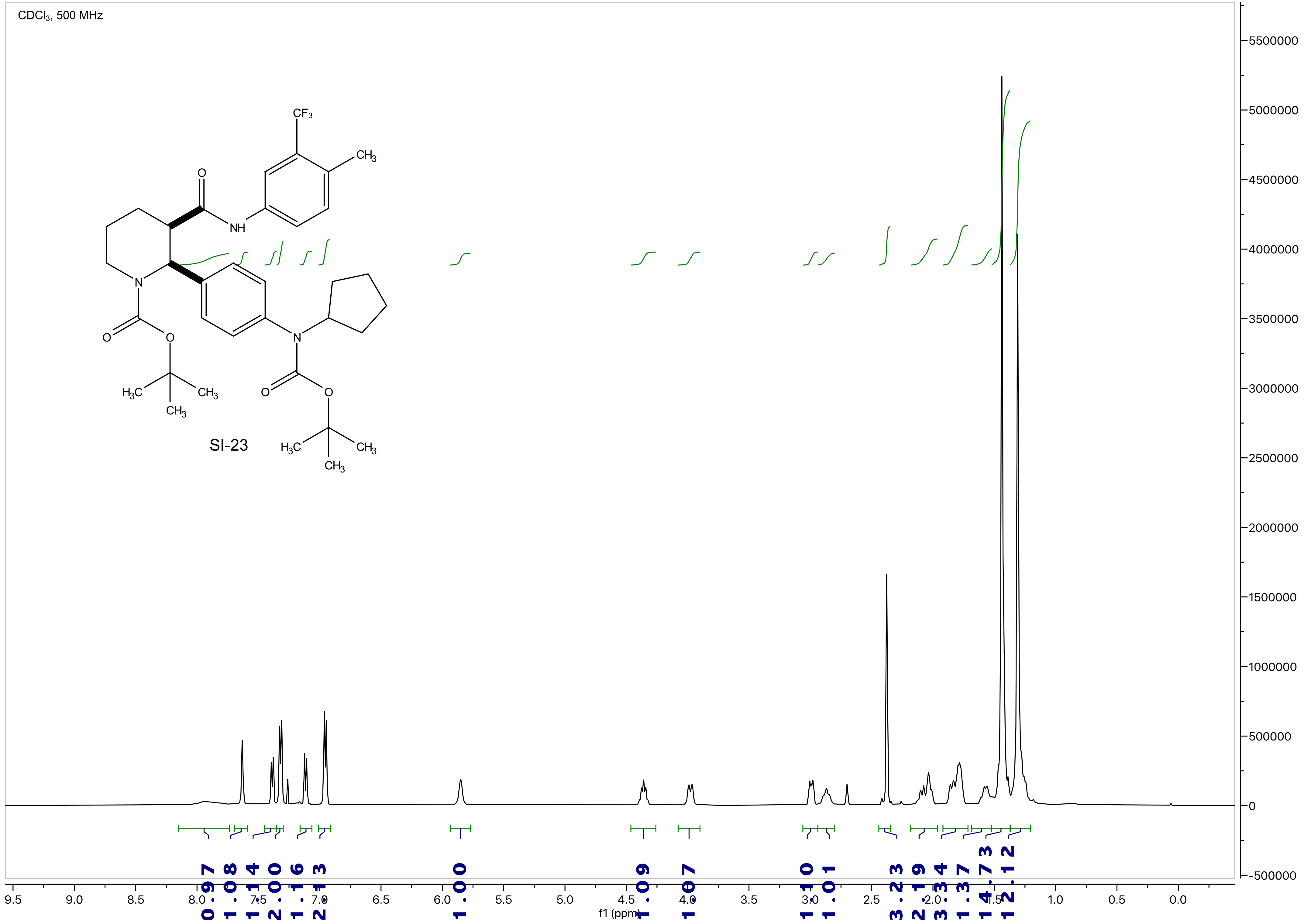
CDCl3, 500 MHz
SI-23
f1 (ppm)

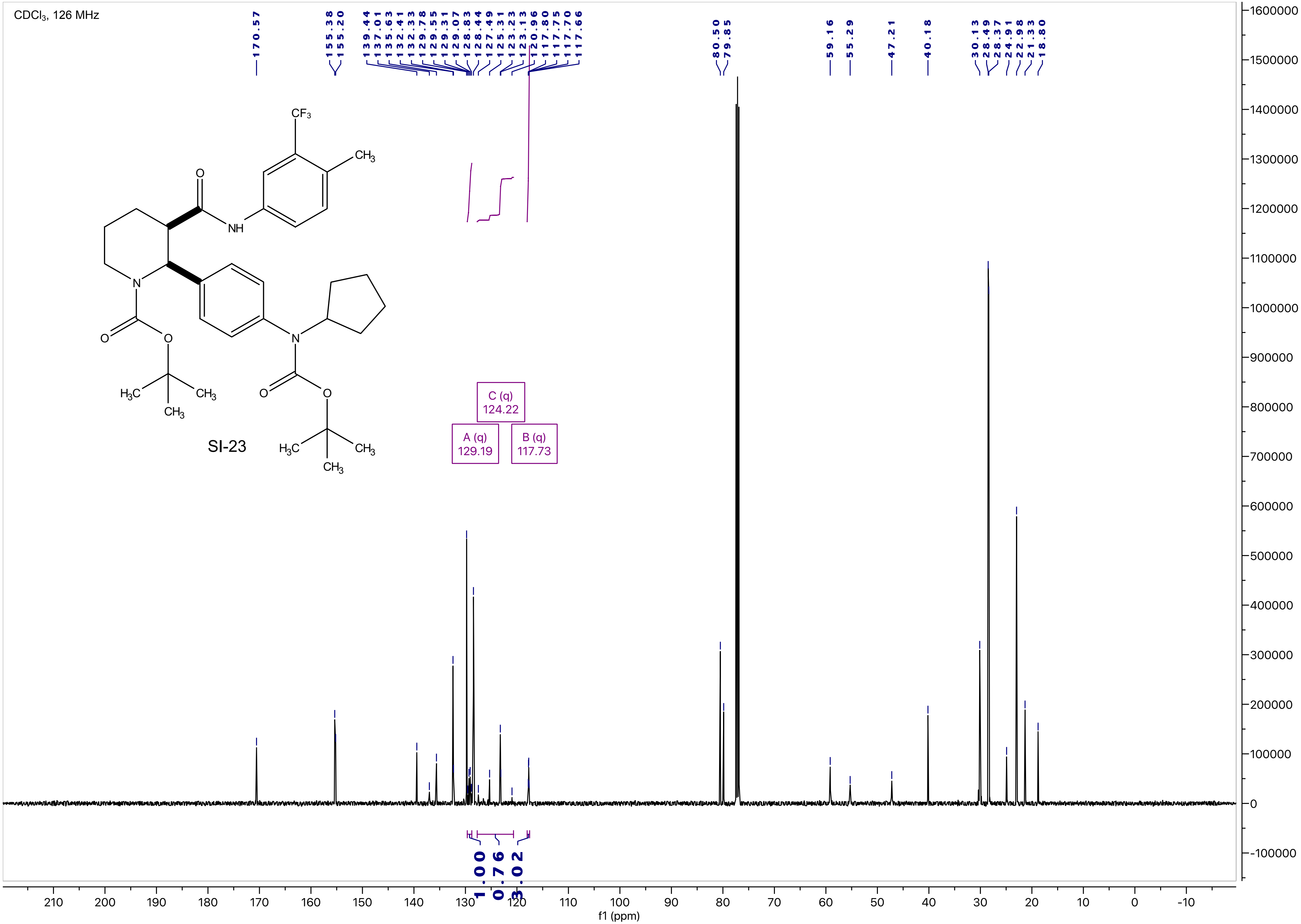
CDCl3, 126 MHz
170.57
155.38
155.20
139.44
137.01
135.63
132.41
132.33
129.78
129.55
129.31
129.07
128.83
128.44
127.49
125.31
123.23
123.13
120.96
117.80
117.75
117.70
117.66
80.50
79.85
59.16
55.29
47.21
40.18
30.13
28.49
28.37
24.91
22.98
21.33
18.80
C (q)
124.22
A (q)
129.19
B (q)
117.73
1.00
0.76
3.02
CF3
CH3
O
NH
N
O
O
H3C
CH3
CH3
N
O
O
H3C
CH3
CH3
SI-23
f1 (ppm)

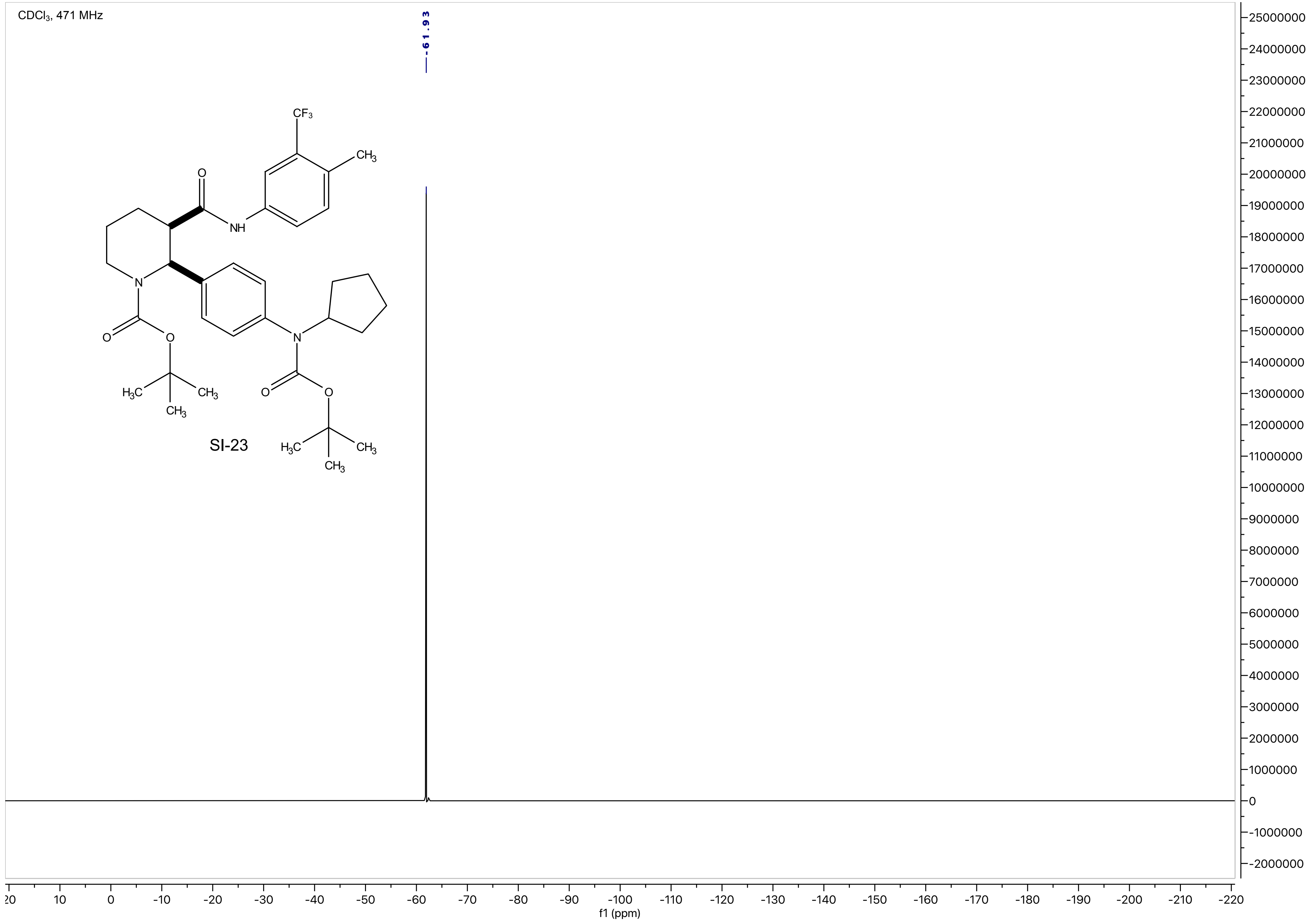

CDCl3, 471 MHz
-61.93
SI-23
f1 (ppm)

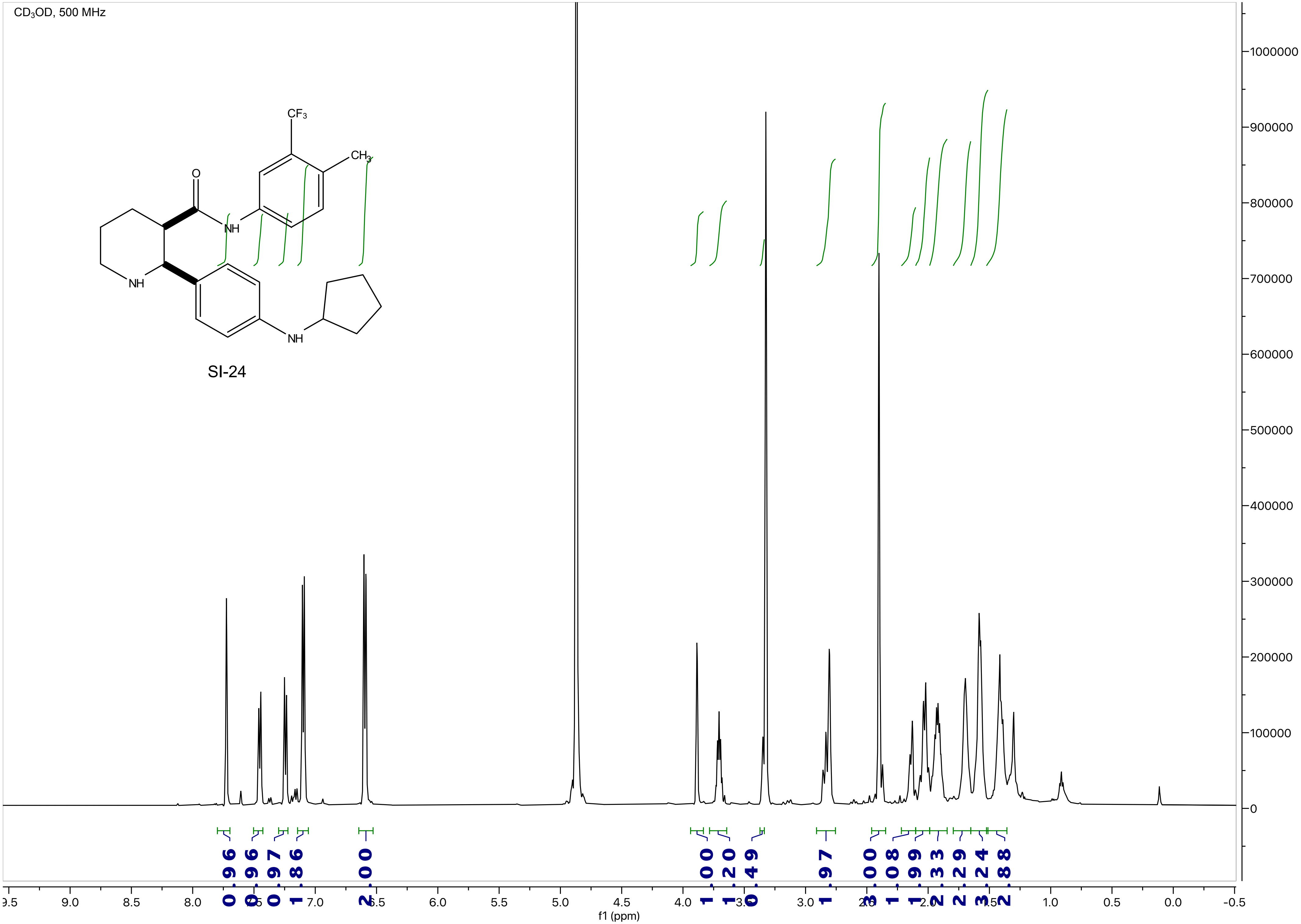

CD3OD, 500 MHz
CF3
CH3
O
NH
NH
NH
SI-24
0.96
0.96
0.97
1.86
2.00
1.00
1.20
0.49
1.97
3.00
1.08
1.99
2.33
2.29
3.24
2.88
f1 (ppm)

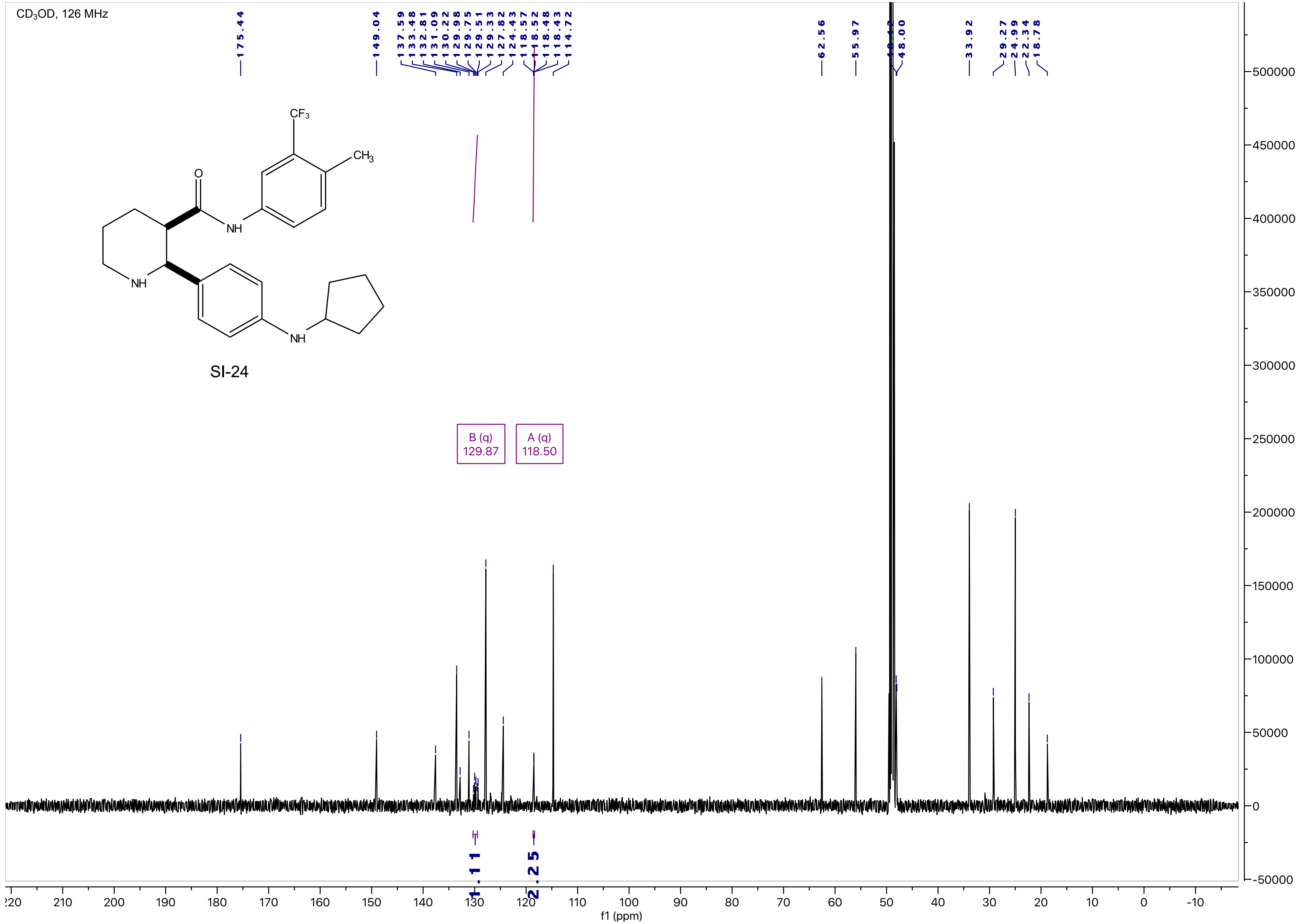
CD3OD, 126 MHz
175.44
149.04
137.59
133.48
132.81
131.09
130.22
129.98
129.75
129.51
129.33
127.82
124.43
118.57
118.52
118.48
118.43
114.72
62.56
55.97
48.00
33.92
29.27
24.99
22.34
18.78
CF3
CH3
O
NH
NH
NH
SI-24
B (q)
129.87
A (q)
118.50
1.11
2.25
f1 (ppm)

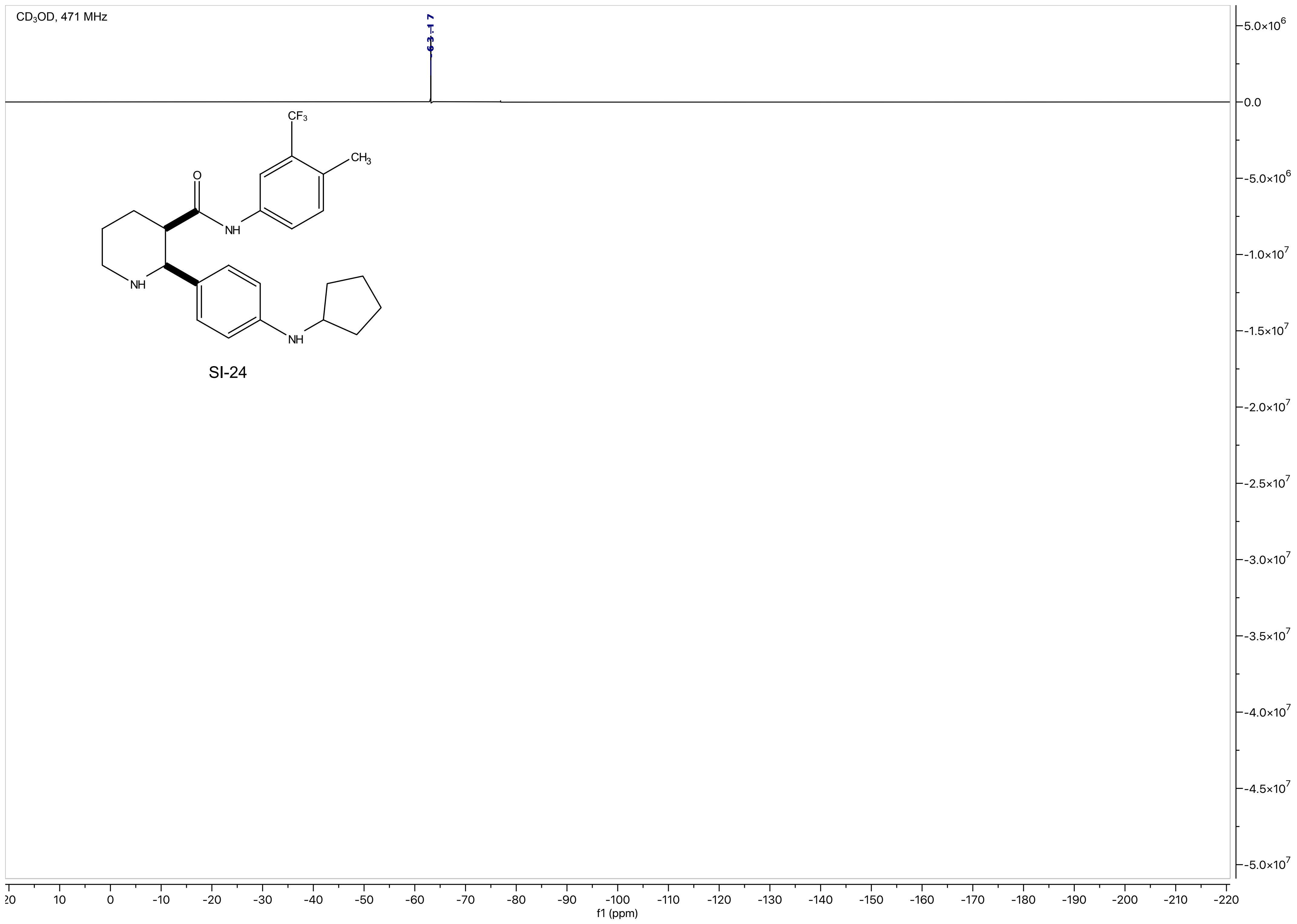

CD3OD, 471 MHz
-63.17
CF3
CH3
O
NH
NH
NH
SI-24
5.0×10^6
0.0
-5.0×10^6
-1.0×10^7
-1.5×10^7
-2.0×10^7
-2.5×10^7
-3.0×10^7
-3.5×10^7
-4.0×10^7
-4.5×10^7
-5.0×10^7
20 10 0 -10 -20 -30 -40 -50 -60 -70 -80 -90 -100 -110 -120 -130 -140 -150 -160 -170 -180 -190 -200 -210 -220
f1 (ppm)
